# Proceedings of the 24^th^ Paediatric Rheumatology European Society Congress: Part one: Athens, Greece. 14-17 September 2017

**DOI:** 10.1186/s12969-017-0185-x

**Published:** 2017-09-01

## Clinical aspects and treatment of systemic lupus erythematosus (SLE)

### O1 JSLE and the NLRP3 inflammasome – a novel therapeutic target

#### Jo Gamble^1^, Michael W. Beresford^2,3^

##### ^1^Child Health, Translational Medicine, University of Liverpool, Liverpool UK; ^2^Child Health, University of Liverpool, Liverpool, UK; ^3^Alder Hey Children's Hospital, Liverpool, UK

###### **Correspondence:** Jo Gamble

**Introduction:** Juvenile-onset Systemic Lupus Erythematosus (JSLE) is a severe autoimmune disease causing organ damage and long-term morbidity. Impaired clearance of nuclear debris from cells and upregulated circulating damage-associated molecular patterns (DAMPs) and cytokines are thought to trigger cell death and thus, further increase release of pro-inflammatory molecules following the cytosolic assembly of inflammasomes.

**Objectives:** To investigate the role of pyroptotic cell death in JSLE, which occurs following the assembly and activation of the NLRP3 inflammasome, which may be a promising target in the treatment of JSLE and other inflammatory diseases.

**Methods:** THP-1 cell line-derived macrophages (Mφs) were primed using lipopolysaccharide (LPS; 10 ng/ml) and interferon (IFN) γ (20 ng/ml) in complete media at 37 °C for 24 hours, and subsequently treated with 10 mM Adenosine triphosphate (ATP) for 30 min. Primed Mφs not treated with ATP were used as a negative control. Some Mφs were incubated with 10% JSLE patient sera or NETosis-derived material (10 ng/ml) from PMA-treated neutrophils. Primed Mφs were tested for cell surface markers, HLA-DR, CD282 (TLR2), and CD68 using flow cytometry. ATP-treated Mφs were collected and either assayed for pyroptosis marker, lactate dehydrogenase (LDH) activity, cleaved caspase-1 with immunofluorescence (IF), or lysed for cleaved caspase-1 using western blotting.

**Results:** Primed Mφs showed an M1 phenotype with geometric means ± SEM of HLA-DR, TLR2 and CD68 expression respectively of: 1177 ± 1.15, 613 ± 0.9, and 1549 ± 0.9 gMFI, respectively compared with un-primed Mφs of 597 ± 1.0, 122 ± 0.88, and 1225 ± 0.9 gMFI, respectively (n = 3; p < 0.05). ATP-treated Mφs showed increased LDH activity compared to controls (3.4x10^-3^ ± 0.12x10^-3^) compared to 0.00 milliunits/mL, respectively; n = 3; p < 0.05). Furthermore, a greater increase in LDH activity was observed in Mφs that were incubated with JSLE serum and NET material (3.7x10^-3^ ± 0.5x10^-3^ and 7.6x10^-3^ ± 0.5x10^-3^, respectively, compared to 0.00 milliunits/mL; n = 3-6; p < 0.05). IF was positive for cleaved caspase-1 in ATP treated Mφs; and this was confirmed in lysed cells, using western blotting.

**Conclusion:** Overall, the results indicate that Mφs undergo pyroptosis via the NLRP3 inflammasome when challenged with a two-signal approach of priming and ATP, and that cytokines, nuclear debris and DAMPs may not only trigger, but amplify the inflammatory response of this pathway. The NLRP3 inflammasome is thought to be an important mediator in the pathogenesis of certain inflammatory diseases, and flare episodes associated with JSLE. Further work is planned to investigate the role of the inflammasome in JSLE, using pharmacological interventions with specific known and novel inhibitors of the NLRP3 inflammasome. This work could prove the NLRP3 inflammasome to be a promising target for future therapy for JSLE.

**Disclosure of Interest:** None Declared

## Big data analytics

### O2 Persistence of CD4 memory pathogenic subsets in polyarticular juvenile idiopathic arthritis patients who relapse upon withdrawal of biologic therapy

#### Jing Yao Leong^1^, Joo Guan Yeo^1,2^, Phyllis Chen^1^, Liyun Lai^1^, Loshinidevi D/O Thana Bathi^1^, Justin Tan^2^, Thaschawee Arkachaisri^2, 3^, Daniel J. Lovell^4,5^, Salvatore Albani^1,3^

##### ^1^Singhealth Translational Immunology and Inflammation Centre (STIIC), Singapore Health Services Pte Ltd, Singhealth, Singapore, Singapore; ^2^KK Women's and Children's Hospital, Singapore, Singapore; ^3^Duke-NUS Graduate Medical School, Singapore, Singapore; ^4^Division of Rheumatology, Cincinnati Children's Hospital Medical Centre, Cincinnati, OH, United States; ^5^Department of Paediatrics, University of Cincinnati College of Medicine, Cincinnati, OH, United States

###### **Correspondence:** Jing Yao Leong

**Introduction:** Clinical management of polyarticular JIA with anti-TNF-alpha biologics has been met with significant success, with up to 80% of patients demonstrating clinically meaningful efficacy. Concerns about medium/long term drug toxicities and costs have driven the clinical need to find predictors for successful drug discontinuation. Emerging evidence from previous published data indicate that T cells play a crucial role in the disease progression. Defining the pathogenic subsets and mechanisms within the T cell immunome will likely help stratify patients in terms of therapeutic outcomes.

**Objectives:** We seek to distill this pathogenic signal hidden within the T cell compartment through the utilisation of a high dimensional platform, CyToF, that is capable of phenotyping up to 41 markers at a single cell resolution. JIA patients treated with anti-TNF-alpha biologics were recruited in the Understanding TNF-alpha trial and segregated into flare, active and inactive arms after drug discontinuation. The central aim of this project is to identify pathogenic immune mechanisms of clinical relapse and signatures capable of distinguishing clinical fates.

**Methods:** Patients treated with anti-TNF-alpha biologics were recruited into the study (Improved Understanding of the Biology and Use of TNF inhibition in Children with JIA Trial) with clinically inactive disease on treatment (Wallace criteria) and initiated with therapy discontinuation. The patients were followed and evaluated as flare, inactive and active based on 6 JIA core set parameters; number of joints with active arthritis and/or loss of motion, MD global assessment of current disease activity, patient/parent global assessment of overall disease severity in prior week, a validated measure of physical function and ESR. Healthy paediatric controls without any inflammatory diseases were recruited from the day surgery during intravenous plug setting pre-operatively after informed consent.

**Results:** PBMCs from n = 47 JIA patients (Flare n = 18, Active = 11, Inactive n = 18) and n = 10 healthy controls were stained with 37 markers/4 barcodes T cell CyToF Panel after 6 hours stimulation with PMA/Ionmycin. Dimensional reduction cluster analysis was performed with Marvis and unique nodes representing immune subsets enriched in flare or active disease was statistically filtered (Mann Whitney Test, p < 0.05) and manually verified through conventional bivariate gating (FlowJo). In patients destined to flare (vs inactive/healthy) prior to therapy withdrawal, the CD4 memory compartment was strongly dysregulated (p < 0.05), enriched particularly in (a) CD4 CD45RA^-^ TNF-alpha^+^, (b) CD4 CD45RA^-^ CXCR5^+^ (TfH), and were skewed towards (c) CD152^-^/PD1^-^.When contrasting against only healthy non-disease controls, patients destined to flare additionally were up-regulated for CD4 CD45RA^-^ TNF-alpha^+^ IL-6^+^, possibly representing as a sub-clinical disease subset. Intriguingly we noted a migratory subset, that was not evident in earlier flare cohort comparisons, CD4 CD45RA^-^ CXCR3^+^ CCR6^+^ that was present in patients destined to develop active disease (vs inactive/healthy). Upon flaring, the sub-clinical subset CD4 CD45RA^-^ TNF-alpha^+^ IL-6^+^ surfaces and CD4 memory subsets are now upregulating expression of CD152/PD1 (vs inactive) in response to ongoing inflammation, but when compared to healthy controls are still inadequate.

**Conclusion:** There exists a group of patients (flare) for which biologic therapy with anti-TNF-alpha is merely controlling the disease activity but not curative. The persistence of CD4 memory cells are likely to play a pivotal role in disease relapse, that may be partially explained by a weaker control through immune checkpoints (CD152/PD1) or interaction with B cells. These results suggest that clinical fate is immunologically predetermined and patients who will develop different clinical fates can be identified from prior biologic sampling.

**Disclosure of Interest:** None Declared.

### O3 A multi-dimensional immunomics approach reveals distinct regulatory and antigen presenting B cell changes associated with childhood onset systemic lupus erythematosus

#### Joo Guan Yeo^1,2^, Jingyao Leong^1^, Thaschawee Arkachaisri^2,3^, Angela Yun June Tan^2^, Loshinidevi D/O Thana Bathi^1^, Xuesi Sim^1^, Phyllis Zi Xuan Chen^1^, Liyun Lai^1^, Lena Das^2^, Justin Hung Tiong Tan^2^, Elene Seck Choon Lee^2^, Yun Xin Book^2^, Salvatore Albani^1,2,3^

##### ^1^Singhealth Translational Immunology and Inflammation Centre (STIIC), Singapore Health Services Pte Ltd, Singapore, Singapore; ^2^KK Women's and Children's Hospital, Singapore, Singapore; ^3^Duke-NUS Graduate Medical School, Singapore, Singapore

###### **Correspondence:** Joo Guan Yeo

**Introduction:** Systemic Lupus Erythematosus (SLE) is a multi-factorial autoimmune disease and the conventional oligo-dimensional investigative approach involving one or a few cell types or analyses at a time is inadequate for its study. We hypothesize that abnormalities within multiple components of the immune system contribute to lupus pathogenesis and hence, there is a need for a comprehensive interrogative analysis.

**Objectives:** We aim to employ a multi-dimensional holistic approach using mass cytometry to study the immunome of childhood onset SLE (cSLE) patients and unravel the immune derangements involved in its pathogenesis. This will address the critical unmet need for a simultaneous and holistic interrogation of the different immune cell subsets in cSLE.

**Methods:** Peripheral blood mononuclear cells (PBMC) from 14 cSLE patients and 14 healthy paediatric controls, were stained for 37 immune phenotypic markers after 72 hours of culturing with and without Class B CpG oligodeoxynucleotide stimulation followed by interrogation with mass cytometry. Blood from healthy paediatric subjects without any inflammatory diseases were obtained prior to their elective day surgical procedures during intravenous plug setting after informed consent. Subsequent analysis of the data was done using a machine learning approach with dimensional reductions followed by automated cell classification, clustering and visualization. Unique nodes representing immune subsets enriched in cSLE patients were statistically evaluated with reference to the healthy cohort and its clinical and mechanistic significance inferred from their phenotypes (Wilcoxon rank-sum test, *p* < 0.05).

**Results:** A statistically significant enrichment of a class-switched memory B cell subset (CD19^+^CD27^+^IgG^+^) with CD11c^+^CD25^+^HLA-DR^+^CD40^hi^CD86^hi^ was found in the SLE cohort, suggesting its pathologic mechanistic role in lupus. Intriguingly, CD25 (IL-2α chain) expression was found in this population, indicative of its potential responsiveness to IL-2 secreted T cells. This observation was coupled with a reciprocal increase in the transitional/naive B cell population that was negative for CD11c, CD25, CD40 and CD86 in the healthy cohort. Next, a significant enrichment of the memory regulatory T cell population (CD4^+^CD45RO^+^CD25^+^Foxp3^+^) was present in the diseased cohort with a small population of these cells expressing CXCR5. CXCR5 is a homing chemokine receptor to the lymph node germinal centre, an important lupus related microenvironment, and its presence may signify a potential regulatory role of these cells. In the healthy cohort, a reciprocal enrichment in the naive regulatory T cell population was found instead. Lastly, the B regulatory compartment of the PBMC was interrogated after CpG stimulation demonstrating an enrichment of IL10 secreting B regulatory cells with the naive and transitional B cell phenotypes (IgM^+^IgD^+/-^CD27^-^) in the cSLE patients. These populations of T and B regulatory cells may be involved in the amelioration of the lupus disease activity during different phases of the illness.

**Conclusion:** A holistic multi-dimensional approach was able to distill multiple derangements in the cSLE immunome with clinical and mechanistic significance. The identification of a B cell subset with phenotypic markers indicative of its ability for antigen presentation (HLA-DR, CD86) and T-B cell interaction (CD25, CD40) in the cSLE cohort highlights that the immunopathogenic role of B cell in SLE goes beyond antibodies production and immune regulation. Additionally, the concurrent demonstration of an increase in memory CD4 regulatory cells and B regulatory cells in the diseased cohort is consistent with our hypothesis that multiple immune abnormalities are involved in SLE pathogenesis. These findings have the translational potential to unravel the pathogenesis of lupus and identify the cellular subsets for further in-depth mechanistic and functional studies for the eventual goal of developing novel therapeutics.

**Disclosure of Interest:** None Declared.

### O4 Using blood transcriptomic data for disease etiology discovery in pediatric ANCA-associated vasculitis

#### Erin Gill^1^, Kelly Brown^2^, Kim Morishita^2^, David A. Cabral^2^, Robert E. W. Hancock^1^

##### ^1^University of British Columbia, Vancouver, Canada; ^2^BC Children's Hospital, Vancouver, Canada

###### **Correspondence:** Erin Gill

**Introduction:** Primary vasculitis encompasses a number of life threatening diseases. The different clinical manifestations and classification framework is partly determined by the size (small, medium, large) of the predominantly inflamed blood vessels. Among small vessel vasculitis (SVV) the Anti-Neutrophil Cytoplasmic Antibody (ANCA) -associated vasculitis (AAV) subtype includes, Microscopic Polyangiitis (MPA) and Granulomatosis with Polyangiitis (GPA) that are difficult for physicians to differentiate because of their many overlapping clinical features^1^. In the absence of biomarkers, classification algorithms (European League Against Rheumatism (EULAR^2^) and European Medicines Agency (EMA^3^) algorithms) exist to distinguish these diseases in adults and have been adapted for use in children. Although rare in childhood, the study of AAV in pediatric patients provides opportunity for biomarker discovery in individuals with a relatively naïve immune system and limited comorbidities.

**Objectives:** Our group has collected blood for RNA-Seq analysis from children diagnosed with AAV from centres around the world. Transcriptome analyses were conducted in an attempt to detect biomarkers for MPA and GPA that will help determine disease etiology and assist physicians in patient classification.

**Methods:** Briefly, blood was collected in Tempus tubes, RNA was extracted, enriched for mRNA and RNA-Seq libraries were prepared using standard methods. Sequencing was performed on an Illumina HiSeq 2500. fastq reads were mapped to the human genome using STAR^4^. DESeq2^5^ was used for differential expression analysis while pathway overrepresentation analysis was conducted via innateDB^6^ using the Reactome^7^ pathway annotation system. Samples from 32 patients at the point of diagnosis or flare-up at blood draw were included in the analysis. All 32 patients were classified using the EMA algorithm, although the EMA classification was not used in the analysis.

**Results:** Hierarchical clustering based on Euclidean distances between samples placed samples into three clusters. Cluster one is composed of only three samples, while clusters two and three are composed of 14 and 15 samples, respectively. Cluster two contains primarily samples from individuals who were classified (via the EMA algorithm) as having MPA or unclassifiable SVV (10/14 samples), while cluster three contains primarily samples from individuals who were classified as having GPA or unclassifiable SVV (14/15 samples). DE analysis between clusters two and three showed 3,198 differentially expressed genes. The MPA cluster is enriched for pathways including interferon signaling, the ISG15 antiviral mechanism, TCR signaling and adaptive immunity. Conversely, the GPA cluster is enriched for pathways including interleukin signaling, TLR4 signaling, and phagosomal maturation. These enriched pathways suggest a viral and adaptive immunity signature for MPA and a bacterial and innate immunity signature for GPA.

**Conclusion:** The identification of preliminary etiologies for GPA and MPA is the first step toward assisting clinicians in developing improved molecular diagnostics for disease classification, and suggest an opportunity for seeking different treatment paradigms for the management of these two diseases.

**Disclosure of Interest:** None Declared.

**References** 1. Barut, K. et al. 2016. Curr Opin Rheumatol 28:29.

2. Luqmani, R.A. et al. 2011. Clin Exp Immunol 164:11.

3. Abdulkader, R. et al. 2012. Ann Rheum Dis 72:1888.

4. Dobin, A. et al. 2013. Bioinformatics 29:15.

5. Love, M.I. et al. 2014. Genome Biol. 15:550.

6. Breuer, K. et al. 2013. Nucl Acids Res. 41:D1.

7. Fabregat, A. et al. 2016. Nucl Acids Res. 44:D481.

## Remission and outcome of systemic lupus erythematosus (SLE)

### O5 Impact of fatigue, depressive symptoms and skin symptoms in adults with childhood-onset sle and the relation with HRQoL – the CHILL-NL study

#### Ayse Kaynak^1^, Noortje Groot^1,2^, Santusja Ramnath^1^, Marc Bijl^3^, Radboud Dolhain^4^, Onno Teng^5^, Els Zirkzee^6^, Karina de Leeuw^7^, Ruth Fritsch-Stork^8^, Irene Bultink^9^, Sylvia Kamphuis^1^ and on behalf of the CHILL-NL study group

##### ^1^Department of Pediatric Rheumatology, Sophia Children's Hospital – Erasmus University Medical Centre, Rotterdam, Netherlands; ^2^Department of Pediatric Immunology, Wilhemina Children’s Hospital – University Medical Centre Utrecht, Utrecht, Netherlands; ^3^Department of Internal Medicine and Rheumatology, Martini Hospital, Groningen, Netherlands; ^4^Department of Rheumatology, Erasmus University Medical Centre, Rotterdam, Netherlands; ^5^Department of Rheumatology, Leiden University Medical Center, Leiden, Netherlands; ^6^Department of Rheumatology, Maasstad Hospital, Rotterdam, Netherlands; ^7^Department of Rheumatology and Clinical Immunology, University Medical Center Groningen, Groningen, Netherlands; ^8^Department of Rheumatology and Clinical Immunology, University Medical Center Utrecht, Utrecht, Netherlands; ^9^Amsterdam Rheumatology and Immunology Center, Location VUmc, Amsterdam, Netherlands

###### **Correspondence:** Ayse Kaynak

**Introduction:** Childhood onset SLE is a lifelong heterogeneous autoimmune disease that follows a more severe clinical course than adult-onset SLE. Previous studies have shown that HRQoL is impaired in cSLE, little is known which factors influence HRQoL. Fatigue, depressive symptoms and skin/mucosal symptoms (leading to changed physical appearance and potential lower self-esteem) may play an important role for a patient’s HRQoL.

**Objectives:** To determine the prevalence of fatigue, depressive symptoms and skin/mucosal symptoms in adults with cSLE and examine the relation with HRQoL

**Methods:** 106 adults with cSLE were included in the CHILL-NL (CHILdhood Lupus-NetherLands) study including a single study visit for structured history, physical examination and SLEDAI-2 k. HRQoL was assessed with SF-36. Fatigue was assessed with Fatigue Severity Scale (FSS) with scores range from 1 to 7. FSS score ≥4 defines abnormal fatigue. Beck Depression Inventory (BDI) was used for depressive symptoms. BDI score ≥14 is indicative for major depressive symptoms.

**Results:** 92% of cSLE patients were female and white (73%). The median age at study visit was 33 yrs, median disease duration was 20 years, 61% had developed damage (SDI ≥1). HRQoL of cSLE patients was impaired (7/8 domains SF36), remarkably mental health was similar between patients and Dutch norm data. Patients with damage (SDI ≥1) scored remarkably similar on all HRQoL domains, except for the domain ‘physical functioning’. 68% of cSLE patients were abnormal fatigued (FSS score ≥4). 15% were depressive (BDI-score ≥ 14). HRQoL (6/8 domains SF36) was lower in all patients with fatigue but also in those with major depressive symptoms. The median SLEDAI-2 k score was 4, 31% of cSLE patients had ≥ 1 skin/alopecia/mucosal ulcers and its presence was associated with lower HRQoL.

BDI: Exel E, Lupus 2013;22(14):1462-FSS::ad hoc committee on SLE response criteria, ArthrRheum2007;57(8):1348

**Conclusion:** HRQoL in this large cohort of adults with cSLE was not associated with disease damage, but self-reported fatigue- and major depressive symptoms were. Additionally physical appearance (skin/alopecia) and mucosal symptoms had a clear impact on HRQoL.

**Disclosure of Interest:** None Declared.

### O6 Clinical predictors of developing lupus nephritis in children

#### Eve M. Smith^1^, Peng Yin^2^, Andrea L. Jorgensen^2^, Michael W. Beresford^1^

##### ^1^Department of Women and Children’s Health, Institute of Translational Medicine, Liverpool, United Kingdom; ^2^Department of Biostatistics, Institute of Translational Medicine, University of Liverpool, Liverpool, United Kingdom

###### **Correspondence:** Eve M. Smith

**Introduction:** A proportion of patients with Juvenile-onset Systemic Lupus Erythematosus (JSLE) will have lupus nephritis (LN) as part of their initial presentation, but others will go on to develop this manifestation later [1, 2]. Early recognition and appropriate management of LN is important as early response to treatment is known to be associated with better renal outcomes [3]. Identifying those with or at risk of developing LN is important, so that clinicians can be extra vigilant in monitoring for LN development.

**Objectives:** (1) To characterise patients with LN at baseline, and how they differ from patients without LN in terms of clinical and demographic factors. (2) For those without LN at baseline, who develop it at a later stage, to determine (a) when they develop LN and (b) whether there are clinical and demographic predictors at baseline which can predict subsequent development of LN.

**Methods:** Participants of the UK JSLE Cohort Study, between 2006-2016, were included if they had active LN at the time of their initial presentation to paediatric rheumatology care (referred to as ‘baseline’ throughout), or subsequently developed LN. Univariate logistic regression modelling compared clinical/demographic factors of patients with/without active LN at baseline. Those without LN at baseline were followed longitudinally, monitoring for LN development. The association between outcome (time from baseline visit to the development of LN) and clinical/demographic variables at baseline was tested univariately using Cox Proportional hazard modelling. Covariates with a p < 0.2 on univariate analysis were included in a multiple regression model and backward stepwise model selection applied. HRs, 95% CIs and p-values were summarised for covariates present in the final model and the results were displayed graphically with Kaplan-Meier curves and risk tables.

**Results:** 331 JSLE patients were included in the study. A total of 121/331 (37%) of UK JSLE Cohort Study patients were found to have active LN at baseline. Of the 210/331 patients without LN at baseline, 13 only had a single study visit and were therefore excluded from further analyses. A further 34/197 (17%) patients were found to develop LN after a median of 2.04 years [IQR 0.8-3.7]. Six clinical/demographic factors were found to differ significantly between those with/without LN at baseline. These included the patient’s first ACR score (p = 3.6 x10-6), presence of severe hypertension (p = 0.0006), level of proteinuria (p = 5.7x10-12), serum creatinine (p = 0.00024), ESR (p = 0.00075) and C3 (p = 1.3x10-7). Within the multiple regression model, both ACR score (p = 0.014) and C3 levels (p = 0.0082) at baseline were also significant predictors for subsequent LN development. Patients with a higher ACR score and a lower C3 value at baseline were at greater risk of developing LN at any point in time (ACR score: HR 1.45, 95% CI 1.08-1.95 and C3: HR 0.27, 95% CI 0.10-0.68).

**Conclusion:** Using clinical data from the UK JSLE Cohort Study, this study has explored the ability of basic clinical and demographic factors at baseline for stratifying JSLE patients as ‘high or low risk’ for LN development at baseline or during their subsequent disease course. Consideration of such factors may help the clinician when considering the individual patients LN risk.

Disclosure of Interest: None Declared.

## Immunometabolomics

### O7 NRF2 enhances metabolic turnover in myeloid cells resulting in expansion of CD11b_+_Gr-1_+_MDSCs

#### Kim Ohl, Patricia Klemm, Klaus Tenbrock

##### Dept Of Pediatrics, RWTH University of Aachen, Aachen, Germany

###### Correspondence: Kim Ohl

**Introduction:** Reactive oxygen species (ROS) were primarily considered as harmful mediators of inflammation due to their ability to damage proteins, lipids, nucleic acids and matrix components. However, accumulating evidence point towards an immune-regulatory role of ROS with inflammation-limiting effects and the ability to prevent autoimmune diseases. Thus, a deeper analysis of ROS induced pathways in different immune cells is crucial to understand the ambivalent role of ROS molecules.

**Objectives:** The aim of our study was to analyse the effects of ROS and oxidative stress on the immune response by deciphering the role of Nrf2 – the key transcription factor of the oxidative stress response – in immune cells.

**Methods:** We generated mice with a constitutive activation of Nrf2 in immune cells. Emerging CD11b^+^Gr-1^+^ cells were analysed by flow cytometry, whole transcriptome analysis and metabolic assays and compared to wildtype and LPS induced MDSCs. Finally functionality of CD11b^+^Gr-1^+^ cells was tested in vivo by means of a transfer colitis model and in an acute lung injury model.

**Results:** Activation of Nrf2 in immune cells induced a massive expansion of splenic CD11b^+^Gr-1^+^ cells, which exhibited characteristics of myeloid derived suppressor cells (MDSCs) such as suppression of T cell proliferation *in vitro* and amelioration of T-cell mediated colitis *in vivo*. Whole transcriptome analysis revealed Nrf2 dependent activation of cell cycle and pentose phosphate pathway, which closely resembled pathways in MDSCs induced by the TLR4 ligand LPS resulting in higher metabolic turnover and cell proliferation. In addition constitutive activation of Nrf2 was protective in LPS induced acute lung injury.

**Conclusion:** Thus Nrf2 is a critical transcriptional regulator for a myeloid cell population that plays a major regulatory role in inflammation and infections. Modulation of MDSCs via targeting of Nrf2-dependent metabolism provides ample opportunities for therapeutic manipulation of MDSCs depending on clinical necessities.

**Disclosure of Interest:** None Declared

### O8 Novel serum broad-based proteomic discovery analysis identifies proteins and pathways dysregulated in juvenile dermatomyositis (JDM)

#### Hanna Kim^1^, Angélique Biancotto^2^, Foo Cheung^2^, Terrance O’Hanlon^3^, Ira Targoff ^4^, Yan Huang^5^, Frederick W. Miller^3^, Raphaela Goldbach-Mansky^5^, Lisa Rider^3^

##### ^1^NIAMS, National Institutes of Health, Bethesda, MD, United States; ^2^CHI, NHLBI; ^3^EAG, NIEHS, National Institutes of Health, Bethesda, MD, United States; ^4^Oklahoma University (OUHSC) and Oklahoma Medical Research Foundation (OMRF), Oklahoma City, OK, United States; ^5^TADS, NIAID, National Institutes of Health, Bethesda, MD, United States

###### **Correspondence:** Hanna Kim

**Introduction:** Juvenile dermatomyositis (JDM) is a complex heterogeneous autoimmune disease. Clinical markers are imperfect to correlate with disease activity. Broad proteomic analysis with high sensitivity and reproducibility may be used biomarker discovery. Novel biomarkers from peripheral blood may help characterize pathogenesis and improve disease monitoring and treatment.

**Objectives:** To define protein biomarkers dysregulated in JDM and better understand JDM pathogenesis using novel aptamer-based proteomic technology proteome in a well-characterized JDM cohort.

**Methods:** Unbiased internal discovery and validation analysis was done using broad proteomic analysis of 1306 protein targets using SOMAscan assay of slow off-rate modified aptamers (SomaLogic, CO) which generates simultaneous quantitative results with high sensitivity and reproducibility. In a discovery cohort, 27 JDM patient sera (prevalent cases on variable treatment, average physician global activity or PGA mean 3.9/10 visual analog scale, with 14 anti-p155/140 or TIF1, 6 NXP2, and 7MDA5 myositis specific autoantibodies or MSAs) was compared versus 19 age and gender-matched healthy controls or HC sera using Mann Whitney U FDR <0.10 cutoff. Resulting protein targets were subsequently analyzed in validation cohort sera (14 prevalent JDM cases with similar characteristics including MSA distribution vs 9 HC) using the same cutoff. Resulting proteins with expression ratio of >1.3 were analyzed using Ingeunity Analysis or IPA (Qiagen, CA). The top 10 pathways were then manually clustered to minimize protein overlap with at least 1 unique protein per pathway cluster. Exploratory analysis of the same set of significant upregulated proteins in JDM were analyzed for correlation with PGA by Spearman rank test.

**Results:** 311 protein targets met above criteria from the discovery cohort; 166 unique proteins met criteria after analysis in the validation cohort. 80 of these proteins were upregulated in JDM versus HC and 59 had expression ratio of >1.3. From IPA analysis, 28 proteins fit into 6 pathway clusters: type I IFN notably including IFN beta, granulocyte/agranulocyte adhesion and diapedesis, remodeling/damage, acute phase response, Th1 pathway, and adipokines. There were between 1-7 unique proteins per pathway cluster but many proteins fit into more than 1 pathway cluster. Among the 10 most highly expressed proteins, the most common associated pathway cluster is type I IFN with granulocyte/agranulocyte adhesion as the second most common. 13 proteins had significant moderate correlation with PGA in JDM from varied pathway clusters with Spearman r values of 0.32-0.41. Further analysis by MSA group is ongoing.

**Conclusion:** Broad quantitative proteomic analysis in a well-characterized JDM cohort identifies key differentiating pathway clusters as above in JDM versus HC including many novel proteins, 13 of which have moderate correlation with PGA. Top upregulated proteins are most commonly associated with type I IFN pathway cluster, with granulocyte-agranulocyte adhesion and diapedesis as the second most common pathway cluster. Further analysis by MSA group is ongoing. While in need of confirmation in other cohorts, these proteins identified through a high-throughput screen bring to light new pathways that may be important in JDM.

This research was supported by the Cure JM Foundation and the Intramural Research Program of the NIH, NIAMS, NIEHS, NHLBI, NIAID, and the CC.

**Disclosure of Interest**: H. Kim Grant/Research Support from: Cure JM Foundation, A. Biancotto: None Declared, F. Cheung: None Declared, T. O’Hanlon: None Declared, I. Targoff: None Declared, Y. Huang: None Declared, F. Miller: None Declared, R. Goldbach-Mansky: None Declared, L. Rider: None Declared.

## Autoinflammatory diseases in practice

### O9 A web-based collection of genotype-phenotype correlations in hereditary periodic fevers from the Eurofever registry

#### Riccardo Papa^1^, Matteo Doglio^1^, Helen J. Lachmann^2^, Seza Ozen^3^, Joost Frenkel^4^, Anna Simon^5^, Benedicte Neven^6^, Jasmin Kuemmerle-Deschner^7^, Huri Ozdogan^8^, Roberta Caorsi^1^, Silvia Federici^1^, Martina Finetti^1^, Nicolino Ruperto^1^, Isabella Ceccherini^9^, Marco Gattorno^1^ and the Paediatric Rheumatology International Trials Organisation (PRINTO) and the Eurofever Project

##### ^1^UO Pediatria II-Reumatologia, Istituto Giannina Gaslini, Genoa, Italy; ^2^National Amyloidosis Centre, Royal Free Campus, University College Medical School, London, United Kingdom; ^3^Department of Pediatric Nephrology and Rheumatology, Hacettepe University, Ankara, Turkey; ^4^Department of Paediatrics, University Medical Center Utrecht, Utrecht, Netherlands; ^5^Department of General Internal Medicine, Radboud University Nijmegen Medical Center, Nijmegen, Netherlands; ^6^Unité d'immuno-hématologie pédiatrique, Hôpital Necker Enfants Malades, Paris, France; ^7^Paediatric Rheumatology, University Hospital Tübingen, Tübingen, Germany; ^8^Ic Hastaliklari ABD, Romatoloji BD, Cerrahpasa Tip Fakultesi, Istanbul, Turkey; ^9^Laboratory of Molecular Genetics, Istituto Giannina Gaslini, Genoa, Italy

###### **Correspondence:** Riccardo Papa

**Introduction:** Because of the large number of common variants or polymorphisms in genes related to hereditary periodic fevers (HPF), genetic results often require an high clinical discrimination. The Infevers database is a large international registry of the different variants described for genes associated to autoinflammatory diseases. Due to the nature of this registry no genotype-phenotype correlation are provided, except for the clinical phenotype of the first patient(s) described for each mutation.

**Objectives:** Aim of this study was to elaborate a registry of genotype-phenotype correlations derived from the patients with HPF enrolled and validated in the Eurofever registry.

**Methods:** We created a table for each HPF describing the genotype-phenotype correlations observed in all the patients enrolled in the Eurofever registry. For autosomal dominant diseases (CAPS, TRAPS), all mutations were analyzed individually. For autosomal recessive diseases (FMF, MKD), homozygous and all combinations of compound heterozygous are described. For each mutation or combination, the following items are shown: number of patients, mean age of onset, disease course (recurrent or chronic), mean duration of fever episodes, clinical manifestations associated with fever episodes, atypical manifestations, complications and response to treatment.

**Results:** We analyzed the genotype-phenotype correlations of 718 patients (313 FMF, 133 CAPS, 114 MKD, 158 TRAPS) already reported in specific papers and validated by at least two experts for each disease. A total of 152 variants were described: 48 variants of *TNFRSF1A*, 30 *NLRP3,* 57 different combinations of *MVK* and 42 *MEFV* for compound heterozygous are available. For each HPF, a table with all the variables described has been established.

**Conclusion:** We provide a useful tool for all the physicians, creating a registry of genotype-phenotype correlation of HPF based on the patients enrolled in the Eurofever registry. This tool is complementary to the Infevers database and will be available at the Eurofever and Infevers websites.

**Disclosure of Interest:** None Declared.

## Genetic aspects of pediatric rheumatic diseases

### O10 IKZF1 mutation underlines the B cell landscape heterogeneity in mendelian lupus

#### Cécile Frachette^1^, Sulliman O. Omarjee^1^, Anne Laure Mathieu^1^, Thibault Andrieu^2^, Paul Mondier^3^, Gillian Rice^4^, Heloïse Reumaux^5^, David Launay^6^, Marc Lambert^7^, Guillaume Lefevre^8^, Nicole Fabien^9^, Christophe Malcus^10^, Isabelle Rouvet^11^, Emilie Chopin^11^, Anne-Sophie Michallet^3^, Thierry Defrance^3^, Thierry Walzer^1^, Yanick Crow^12^, Alexandre Belot^1^

##### ^1^Inserm U1111, Centre International de Recherche en Infectiologie- International Center for Infectiology Research, LYON, France; ^2^Inserm SFR Biosciences Gerland, LYON, France; ^3^Centre International de Recherche en Infectiologie- International Center for Infectiology Research, LYON, France; ^4^Division of Evolution and Genomic Sciences, Faculty of Biology, Medicine and Health, University of Manchester, Manchester, United Kingdom; ^5^Rhumatologie médecine interne pédiatrique, Urgences, Pédiatrie générale et maladies infectieuses Hôpital Salengro, Lille, France; ^6^Department of Internal medicine and Clinical Immunology, UMR 995 Inserm, University of Lille, Lille, France; ^7^Service de médecine interne, Hôpital Huriez, Lille, France; ^8^Lille Inflammation Research International center, Lille, France; ^9^Service d'immunologie, CHU de Lyon HCL-Groupement Hospitalier Sud, Lyon, France; ^10^Laboratoire d'immunologie cellulaire, Groupement hospitalier Edouard herriot, Lyon, France; ^11^Biotechnology Department, Hospices civils de Lyon, Lyon, France; ^12^Inserm UMR 1163, Laboratory of Neurogenetics and Neuroinflammation, Paris, France

###### **Correspondence:** Cécile Frachette

**Introduction:** Next generation sequencing (NGS) represents a revolution in the field of molecular medicine, and offers a new approach to deciphering the pathogenesis of complex diseases. Paediatric-onset SLE (pSLE) is a very rare and more severe phenotype than its adult-onset counterpart, and is possibly associated with a greater contribution of genetic aetiological factors. We have in the past described a B cell-related Mendelian form of lupus due to a deficiency of PKCδ. Here, we identified and characterized a new B cell-related Mendelian lupus secondary to *IKZF1* mutation and compared this novel monogenic disease with PKCδ deficiency.

**Objectives:** The aims of the study were to characterize the molecular impact of a new B cell related variant in *IKZF1* encoding the protein IKAROS, and to study the B cell landscape of two distinct monogenic forms of SLE using mass cytometry (Cytof®).

**Methods:** We designed an NGS panel comprising 200 genes including proven disease-associated as well as prospective candidate genes, and analysed 132 patients. We identified a family with three affected individuals carrying a previously unreported mutation in *IKZF1*. We set up functional assays including oligonucleotide pulldown, B cell phosphorylation and deep B cell immunophenotyping by mass cytometry.

**Results:** We identified a heterozygous missense mutation in *IKZF1* c.359A > T (p. D120V) in three affected patients in a single family. *IKZF1* encodes IKAROS, a key transcriptional factor in B cell development. Functional assays showed that protein stability was not impaired, but DNA-binding was partially impacted. We performed mass cytometry comparing SLE patients carrying a homozygous loss-of-function mutation in *PRKCD* and our newly identified *IKZF1* mutation. We identified B cell clusters, and unsupervised analysis underlined the wide differences between two monogenic diseases leading to SLE.

**Conclusion:** Ikaros and PKCδ dysfunction demonstrate that monogenic lupus can occur as a consequence of distinct anomalies of B cell development underlining the fact that SLE should be considered as a syndrome rather than a single homogenous disease.

**Disclosure of Interest:** None Declared.

## Remission in chronic arthritis

### O11 Development of New JADAS and cJADAS cut-offs for disease activity states in oligoarthritis and rf-negative polyarthritis from a large multinational cohort of children with juvenile idiopathic arthritis

#### Alessandro Consolaro^1,2^, E.H. Pieter van Dijkhuizen^3^, Graciela Espada^4^, Boriana Varbanova^4^, Sheila K. Oliveira^4^, Paivi Miettunen^4^, Gaelle Chédeville^4^, Michael Hofer^4^, Pavla Dolezalova^4^, Ivan Foeldvari^4^, Gerd Horneff^4^, Anne Estmann^4^, Chris Pruunsild^4^, Rosa Merino^4^, Inmaculada Calvo Penades^4^, Pablo Mesa-del-Castillo^4^, Pekka Lahdenne^4^, Maka Ioseliani^4^, Maria Trachana^4^, Olga Vougiouka^4^, Miroslav Harjacek^4^, Ilonka Orban^4^, Tamàs Constantin^4^, Nahid Shafaie^4^, Violeta Panaviene^4^, Marite Rygg^4^, Elzbieta Smolewska^4^, Jose Antonio Melo-Gomes^4^, Jelena Vojinovic^4^, Ekaterina Alekseeva^4^, Tadej Avcin^4^, Veronika Vargova^4^, Nuray Aktay Ayaz^4^, Ozgur Kasapcopur^4^, Yaryna Boyko^4^, Sarah Ringold^4^, Marco Garrone^1,4^, Nicolino Ruperto^1,4^, Angelo Ravelli^1,2^ on behalf of PRINTO and EPOCA Study Group

##### ^1^Istituto Giannina Gaslini, Genova, Italy; ^2^University of Genova, Genova, Italy; ^3^Wilhelmina Children's Hospital, Utrecht, Netherlands; ^4^PRINTO, Genova, Italy

###### **Correspondence:** Alessandro Consolaro

**Introduction:** The measurement of the level of disease activity plays a pivotal role in the care of patients with ju-venile idiopathic arthritis (JIA). To serve this purpose, the Juvenile Arthritis Disease Activity Score (JADAS) was developed in 2009. More recently, a version excluding the acute phase reactant was tested (cJADAS). Cutoff values for the state of remission, low disease activity (LDA), moderate disease activity (MDA) and high disease activity (HDA) were recently developed for the original JADAS score and for the clinical version. These cutoff values are ideally suited for pursuing tight disease control in a treat-to-target strategy, with treatment escalation if the desired JADAS score is not reached. However, although cut-offs were validated in a large and multinational cohort of patients, they were developed in a dataset of patients from a single pediatric rheumatology center an partly before the advent of the so-called biologic era.

**Objectives:** To develop the JADAS and cJADAS cut-off values of remission, LDA, MDA, and HDA for oligoarthritis and RF-negative polyarthritis in a large multinational cohort of JIA patients.

**Methods:** The EPidemiology, treatment and Outcome of Childhood Arthritis (EPOCA) study is aimed to obtain information on the frequency of JIA subtypes in different geographic areas, the therapeutic approaches adopted by pediatric rheumatologists practicing in diverse countries or continents, and the disease and health status of children with JIA currently followed worldwide. More than 9.000 patients with JIA from 118 pediatric rheumatology centres in 49 countries were collected so far. For the development of cut-offs, patients with oligoarthritis and polyarthritis followed in the 20 Centres with the highest frequency of these 2 subtypes were retained. In each centre, the 75th centile of JADAS and cJADAS distribution in patients who were subjectively rated by the attending physician as being in remission and LDA, and the 25th centile of JADAS and cJADAS in patients rated as in HDA, were calculated. The obtained values at each centre were then averaged to obtain the prelimary cut-offs for each disease activity state.

**Results:** The cut-offs validation cohorts were made of 930 patients with oligoarthritis from 20 pediatric rheumatology Centres and 1.004 patients with RF-neg polyarthritis from 20 pediatric rheumatology Centres. Preliminary cut offs values for each versions of JADAS and cJADAS are presented in Table 1.

**Table 1:** **(Abstract O11).** See text for description

	JADAS10	JADAS27	JADAS71	cJADAS10	cJADAS27	cJADAS71
Oligoarthritis						
Remission	1.5	1.5	1.5	1.2	1.2	1.2
LDA	3.9	3.7	3.9	3.4	3.3	3.4
HDA	16.4	16.2	16.4	14.3	14.1	14.3
Polyarthritis						
Remission	2.6	2.6	2.6	2.4	2.3	2.4
LDA	5.1	4.9	5.1	5.1	4.9	5.1
HDA	18.9	18.9	22.7	19.0	20.0	25.3

**Conclusion:** New JADAS and cJADAS temptative cut-offs for remission, LDA, and HDA were calculated. Obtained values will be tested in the validation analysis. The preliminary values are higher than currently available cut-offs.

**Disclosure of Interest:** None Declared.

## New insights into the pathogenesis of systemic lupus erythematosus (SLE)

### O12 Interferon-γ plays a role in the pathogenesis of pediatric systemic lupus erythematosus and its activity correlates with disease activity

#### Gian Marco Moneta^1^, Claudia Bracaglia^1^, Ivan Caiello^1^, Luisa Bracci-Laudiero^1,2^, Rita Carsetti^3^, Fabrizio De Benedetti^1^, Emiliano Marasco^1^

##### ^1^Division of Rheumatology, IRCCS Bambino Gesù Children’s Hospital, Rome, Italy; ^2^Institute of Translational Pharmacology, CNR, Rome, Italy; ^3^B Cell Physiopathology Unit, Immunology Research Area, IRCCS Bambino Gesù Children’s Hospital, Rome, Italy

###### **Correspondence:** Marco Moneta

**Introduction:** Pediatric systemic lupus erythematosus (pSLE) is a rare autoimmune disorder with onset before 18 years of age and characterized by heterogeneous clinical manifestations, unpredictable courses and a substantial risk of morbidity. There is no targeted treatment available for pSLE. In the last decade, several gene expression profiling and protein studies showed an up-regulation of genes typicallyinduced by type I interferons (IFNα/β) in the peripheral blood and tissue samples of pSLE patients. Although blood expression levels of IFNα/β-related genes were also correlated with disease activity of pSLE patients, the mechanism by which type I IFN contribute to pathogenesis has not yet been elucidated.

**Objectives:** In this study, we aim to investigate the role of type II IFN, IFNγ, in the pathogenesis of pSLE evaluating: 1) the expression levels of IFNγ-related genes in the peripheral blood of pSLE patients; 2) the relationship between type II and type I IFNs; 3) the correlations between the expression levels of IFNγ-related genes in peripheral blood and the clinical features of pSLE patients.

**Methods:** We analysed the expression levels of IFNα/β induced genes (IFI27, IFI44L, IFIT1, RSAD2, ISG15, SIGLEC1) and IFNγ induced genes (CXCL9, CXCL10, IDO1) in the peripheral blood of pSLE patients (n = 19) by real time PCR. For each patient, SLEDAI score was calculated. Human peripheral blood mononuclear cells (PBMCs) obtained by healthy donors (HD) (n = 3) were stimulated *in vitro* with recombinant human IFNγ and IFNα2b, expression levels of IFN-regulated genes were evaluated by real time PCR. We used non-parametric Mann-Whitney U test and Spearman *r* for statistical analysis.

**Results:** Expression levels of both IFNα/β-induced genes and IFNγ-induced genes were increased in the peripheral blood of pSLE patients with active disease (n = 9) compared to HD (n = 10) and pSLE patients with inactive disease (n = 10). We developed a type II IFN score similarly to the type I IFN score described by Crow et al. We calculated the type I IFN score and the type II IFN score for each pSLE patient. As previously reported, the type I IFN score was significantly correlated with the SLEDAI of pSLE patients (*r = 0.67, p < 0.01*). We found that the type II score was also significantly correlated with the SLEDAI score of pSLE patients (*r = 0.64, p < 0.01*).

As it is known that some IFN-induced genes can be upregulated by both types of interferons, we asked if IFNγ was able to affect, in human PBMCs, the expression of IFNα/β induced genes (IFI27, IFI44L, IFIT1, RSAD2, ISG15, SIGLEC1) assessed to calculate the type I score: we found that IFNγ treatment induced the expression of type I IFN-related genes in human PBMCs in dose-dependent manner. Interestingly, human PBMC stimulated with recombinant IFNα2b strongly up-regulated the expression of IFNγ.

**Conclusion:** Taken together, these data indicate a possible role of IFNγ in the pathogenesis of pSLE. Both types of IFNs potentiate each other effect on tissues by affecting their reciprocal biological activity. IFNγ correlates with disease activity in pSLE, IFNγ-induced gene expression may represents a promising biomarker in this autoimmune disease. The potential pathogenic role of IFNγ in SLE remains to be elucidated.

**Disclosure of Interest:** None Declared.

## Scleroderma and related disorders

### O13 Reliability and performance of the loscat clinical score for the assessment of activity and tissue damage in a large cohort of patients with juvenile localized scleroderma

#### Anna Agazzi, Gloria Fadanelli, Fabio Vittadello, Francesco Zulian, Giorgia Martini

##### Department of Woman and Child Health, University of Padua, Padova, Italy

###### **Correspondence:** Anna Agazzi

**Introduction:** One of the open issues for Juvenile Localized Scleroderma (JLS) is the assessment and monitoring over time of the extent of inflammation and tissue damage. The lack of reliable and standardized outcome measures has represented, over the years, a significant limitation for both clinical monitoring of the disease and development of therapeutic trials.

**Objectives:** We assessed the reliability of Localized Scleroderma Cutaneous Assessment Tool (LoSCAT) clinical score in comparison with contemporary thermographic analysis. Secondary aim was to evaluate and compare the sensitivity to change of LoSCAT and thermography over time.

**Methods:** A longitudinal observational analysis of patients with JLS, consecutively evaluated at our Paediatric Rheumatology Unit, has been performed by using the LoSCAT clinical score and infrared thermography.

LoSCAT score is composed of two indexes: the modified Localized Scleroderma Skin Severity Index (mLoSSI) which measures disease activity considering any new/enlarged lesion, erythema and thickening, and the Localized Scleroderma Skin Damage Index (LoSDI) which measures tissue damage as dermal atrophy, subcutaneous atrophy and depigmentation.

Infrared thermography is a non-invasive imaging technique detecting the thermal energy emitted from the skin providing a graphical representation of its distribution on the body surface.

Three examiners with different experience in Paediatric Rheumatology blindly evaluated all patients twice, at baseline and at least three months later. At each visit, thermographic analysis and LoSCAT score were performed by each examiner. The inter-rater reliability was assessed by the Intraclass Correlation Coefficient (ICC) and interpreted as follows: ICC values range 0.75-1 excellent reliability, 0.4-0.74 good reliability, <0.4 poor reliability. All statistical analyses, including the analysis of variance (ANOVA), were performed by using IBM SPSS (Vers. 18.0).

**Results:** Forty-seven patients (129 lesions) entered the study, and 26 (79 lesions) were reassessed by same examiners with the same modality after 4.5 (+1.5) months. As for LoSCAT, mLoSSI showed excellent inter-rater reliability expressed by ICC 0.895 (95% CI 0.846-0.931); the analysis of variance (ANOVA) confirmed that values indicated by the 3 examiners, were not different from each other (test F = 1.275 and p = 0.283). The inter-rater reliability for LoSDI was excellent too with ICC = 0.880 (95% CI 0.825-0.921), ANOVA test F = 3.030 (p = 0.052).

In the group of 79 lesions examined twice an improvement for all anatomic sites for both evaluations of the three examiners and thermographic detection was observed. Moreover, thermographic analysis showed statistically significant correlation in different anatomic sites with domains of erythema, dermal atrophy and subcutaneous atrophy of LoSCAT.

**Conclusion:** LoSCAT appears as a promising outcome measure in JLS, distinguishing the different aspects of activity and damage. LoSCAT is not influenced by the experience of the examiner. Infrared thermography confirms to be a very helpful tool for detecting disease activity and reliable in monitoring lesions over time.

**Disclosure of Interest:** None Declared.

### O14 Tocilizumab is a promising treatment option for therapy resistent juvenile localised scleroderma patients

#### Ivan Foeldvari^1^, Jordi Anton^2^, Mark Friswell^3^, Blanca Bica^4^, Jaime de Inocencio^5^, Angela Aquilani^6^, Nicola Helmus^1^

##### ^1^Hamburg Center for Pediatric and Adolescent Rheumatology, AM Schöen Klinik Eilbek, Hamburg, Germany; ^2^Sant Joan de Déu Hospital, Barcelona, Spain; ^3^Great North Children’s Hospital, Newcastle, United Kingdom; ^4^Universidade Federal do Rio de Janeiro, Rio de Janeiro, Brazil; ^5^University Hospital 12 de Octubre, Madrid, Spain; ^6^Ospedale Bambino Gesù, Rome, Italy

###### **Correspondence:** Ivan Foeldvari

**Introduction:** Juvenile localised scleroderma (jlSc) is an orphan disease. Most patient respond to treatment ot methotrexate or mycophenolate. In case of nonresponse or partial response, based on the promising tocilizumab (TOC) data of adult systemic sclerosis studies, TOC seems to be a promising option. There is no publication regarding the effectiveness of tocilizumab in jLS.

**Objectives:** To assess the effectivity of TOC in jlSC patients, who had nonresponse or partial response on conventional therapy.

**Methods:** Participants of the pediatric rheumatology email board were asked, if they follow patients with jlSc, who are treated with TOC. Clinical characteristics and the response to TOC was assessed.

**Results:** Six centers responded to the survey from the email board, with around 800 participants, and reported 11 patients. The mean age of the patients at disease onset was 5.5 years. Disease duration at time of the initiation of TOC was 53.5. months (range 9 to 109). 5 patients had linear subtype, 3 of them with facial involvement, 2 of them Parry Romberg and one of them coup de sabre. Three had generalized subtype, 2 mixed subtype and 1 limited subtype/morphea. Before starting TOC patients received 10/11 Methotrexate, 7/11 Mycophenolate, 1 abatacept and 1 anti-TNF therapy. Reason to start TOC was in 9 patients increase in Localised Scleroderma Activity Index[1] (mLoSSI). In two patients increased extracutaneous activity was the indication, in one increased activity of arthritis and in the other increased activity of the central nervous system involvement.

The mean duration of tocilizumab therapy was 14.75 months. 2 patients received s.c. according the poly JIA dosing and all other i.v.. There were different i.v doses applied, 5 of them 8 mg/kg every 4 weeks, one of them 8 mg/kg every three weeks, 1 every two weeks and 1 patients received 10 mg/kg every 3 weeks. 3/11 received TOC as monotherapy. 8/11 as combination therapy, 6 of them with Methotrexate and one of each with Mycophenolate or Tacrolimus. Therapy success was reflected by a decreased mLoSSI in 8/11 patients and in 6 patients by a decrease in the Localosed Scleroderma Skin Damage Index[1] (LoSDI). No new lesion occurred during the treatment and in the patients with Parry Romerg subtype(n = 2) no increase in the facial atrophy occurred. In 8/8 patients physician global (VAS 0-100) decreased and in 8/8 the patients global disease activity (VAS 0-100) decreased. In 3/3 patients, were it was applicable, the number of active joints decreased, in one patients the limb discrepancy decreased. The mean modified Rodnan skin score assessed in 8 patients decreased from the mean value of 9.6 to 5.5.

**Conclusion:** Conclusion:

In this small cohort of patients TOC seems to be a promising rescue medication in methotrexate/mycophenolate nonresponsive patients. A prospective controlled study would be important to prove the seen effect in a controlled way.

1. Arkachaisri T, Vilaiyuk S, Torok KS, Medsger TA, Jr.: Development and initial validation of the localized scleroderma skin damage index and physician global assessment of disease damage: a proof-of-concept study. *Rheumatology (Oxford)* 2010, 49(2):373-381.

**Disclosure of Interest:** None Declared.

## Pathogenesis of juvenile idiopathic arthritis (JIA)

### O15 P75NTR/PRONGF up regulation in synovial tissues activates inflammatory responses in chronic arthritis patients

#### gaetana minnone^1^, Melissa Noack^2^, Pierre Miossec^3^, Ivan Caiello^1^, Marzia Soligo^4^, Luigi Manni^4^, Antonio Manzo^5^, Fabrizio De Benedetti^1^, Luisa Bracci-Laudiero^4^

##### ^1^Division of Rheumatology and Immuno-Rheumatology Research Laboratories, Bambino Gesù Children’s Hospital, Rome, Italy; ^2^3 Immunogenomics and Inflammation Research Unit, EA 4130, Edouard Herriot Hospital, Hospices Civils de Lyon and University Claude Bernard Lyon 1, Lyon, France; ^3^Immunogenomics and Inflammation Research Unit, EA 4130, Edouard Herriot Hospital, Hospices Civils de Lyon and University Claude Bernard Lyon 1,, Lyon, France; ^4^Institute of Translational Pharmacology, CNR, Rome, Italy; ^5^Rheumatology and Translational Immunology Research Laboratories (LaRIT), Division of Rheumatology,, IRCCS Policlinico S. Matteo Foundation/University of Pavia, Pavia, Italy

###### **Correspondence:** gaetana minnone

**Introduction:** We have recently demonstrated that synovial fluids of JIA patients contain elevated levels of proNGF, the immature form of the Nerve Growth Factor (NGF), and not mature NGF as believed in the past. Moreover, mononuclear cells of JIA patients show a reduced expression of TrkA, the specific receptor of mature NGF, and an enhanced expression of p75NTR, the specific receptor for proNGF. This results in an inverted ratio of TrkA and p75NTR in JIA patients compared to healthy donors. How this altered proNGF/p75NTR axis can influence the inflammatory response is at present unknown.

**Objectives:** In this study, we focused on the involvement of p75NTR and its ligand proNGF in regulating inflammatory responses in the inflamed synovia and in synoviocytes of arthritis patients.

**Methods:** Fibroblast-like synoviocytes (FLS) were obtained by synovial tissue of rheumatoid arthritis patients (RA FLS) after enzymatic digestion. Osteoarthritis fibroblasts (OF) and skin fibroblasts (SF) were used as control. Using Realtime-PCR and western blot, we evaluated TrkA, p75NTR, sortilin, NGF mRNA expression and protein levels. Realtime-PCR and ELISA were used to evaluate cytokine production.

**Results:** Our preliminary data show that, similarly to JIA SFMC, p75NTR is significantly upregulated in FLS of RA patients, while TrkA is much less expressed than p75NTR. Instead, OF and SF showed a higher expression of TrkA than p75NTR. Moreover, FLS express NGF mRNA and release high amounts of proNGF, but not of mature NGF, in the conditioned media. Inflammatory stimuli, such as IL-1b, further upregulate p75NTR expression in FLS and induce the expression of p75NTR in control fibroblasts. *In vitro*, the addition of proNGF to FLS induces the expression of pro-inflammatory cytokines. This effect is abolished when p75NTR activity was inhibited using LM11A-31, a non-peptide ligand that blocks the binding site of p75NTR for proNGF.

**Conclusion:** These preliminary data suggest that the proNGF found in synovial fluids of chronic arthritis patients is produced and released principally by FLS. The accumulated proNGF binds to its specific receptor p75NTR, which is highly expressed by both FLS and SFMC, inducing pro-inflammatory cytokine expression. Inflammatory stimuli further enhances the expression of p75NTR in FLS, creating a vicious circle that amplify the inflammatory response. The use of p75NTR inhibitors might represent a new therapeutic approach for the treatment of JIA and RA.

**Disclosure of Interest:** None Declared.

## Oral Presentations 1

### O16 Three treatment strategies in recent onset dmard naive juvenile idiopathic arthritis: first results of clinical outcome after 24 months

#### Petra Hissink Muller^1,2^, Danielle Brinkman^1,3^, Dieneke Schonenberg^4^, Wytse van den Bosch^1^, Yvonne Koopman-Keemink^5^, Isabel Brederije^1^, Peter Bekkering^6^, Taco Kuijpers^4^, Marion van Rossum^7^, Lisette W. van Suijlekom-Smit^8^, J M. van den Berg^4^, Stefan Boehringer^9^, C F. Allaart^10^, Rebecca ten Cate^1^

##### ^1^Pediatric Rheumatology, LUMC, Leiden, Netherlands ^2^Pediatric Rheumatology, Erasmus MC Sophia, Rotterdam, ^3^Pediatrics, Alrijne Hospital, Leiderdorp, Netherlands ^4^Department of Pediatric Hematology, Immunology, Rheumatology and Infectious Diseases, Emma Children’s Hospital AMC, University of Amsterdam, Amsterdam, Netherlands ^5^Pediatrics, Juliana Children's Hospital, The Hague, Netherlands ^6^Pediatric Oncology, Princess Máxima Center of Pediatric Oncology, Utrecht, Netherlands ^7^Pediatric Rheumatology, Amsterdam Rheumatology and Immunology Center location Reade, Amsterdam, Netherlands ^8^Pediatric Rheumatology, Erasmus MC Sophia Children's Hospital, Rotterdam, Netherlands ^9^Medical Statistics and Bioinformatics, LUMC, Leiden, Netherlands ^10^Rheumatology, LUMC, Leiden, Netherlands

###### **Correspondence:** Petra Hissink Muller

**Introduction**

The BeSt treatment strategy for children with juvenile idiopathic arthritis (JIA) has not been determined as of today.

Objectives

The aim of the BeSt for Kids study was to investigate, which of three treatment strategies is most effective and safe, by comparing them directly. The therapeutic target in all arms was inactive disease by rapid reduction of disease activity and repeated monitoring and revision of therapy in case of insufficient response. We hypothesized that early treatment with etanercept and methotrexate (arm 3), compared to initial monotherapy (arm 1) or initial combination therapy with methotrexate and prednisone (arm 2), would lead to significantly earlier clinical inactive disease.

**Methods**

We conducted a randomized, single blinded, multicenter, treatment strategy study with 24 months of follow up. Disease modifying anti rheumatic drug (DMARD)-naive JIA patients were randomized to 1. sequential DMARD-monotherapy (sulfasalazine (SSZ) or methotrexate (MTX), 2. combination therapy: MTX and prednisolone-bridging, 3. Combination-therapy MTX with etanercept. For all arms, the treatment protocol described a number of subsequent treatment steps in case medication failed. After reaching remission for 3 (oligoarticular) or 6 months (polyarticular) medication was tapered and stopped as dictated per protocol and flare frequency was observed. Missing data were imputed. Primary outcome was time to inactive disease and time to flare after tapering and stopping DMARD therapy, both calculated using Kaplan Meier method with log rank test. Secondary outcomes are adjusted ACRPedi 30/50/70 scores, toxicity, functional ability and quality of life. Generalised Estimating Equations were used for longitudinal data analyses. In this abstract we share the first 24 months results.

**Results:** Ninety-four patients were randomised, 32 in arm 1, 32 in arm 2 and 30 in arm 3. Two patients received a different diagnosis during follow-up and were left out of all analysis. Two patient were lost to follow up but were analysed due to intention to treat principle. Overall baseline median (InterQuartileRange IQR) age was 9.1 (4.6-12.9) years. 37% were ANA positive, 11 patients had oligo-articular disease, 66 patients polyarticular JIA and 15 patients juvenile psoriatic (polyarticular) arthritis. Baseline median (IQR) ACRpedi-scores: VAS physician 50 (39-58) mm, VAS patient 54 (37-70) mm, ESR 6(2-14) mm/hr, active joint count 8 (5-12), limited joint count 2.5 (1-5), CHAQ score 0.9 (0.6-1.5). Inactive disease occurred overall after mean 9.8 months (8.5-11.1). Time to inactive disease was not significantly different in all three arms (log rank test p = 0.23). Outcome measures after 24 months of therapy are summarised in the Table 2.

**Table 2:** **(abstract O16).** See text for description

	Arm 1 n = 31	Arm 2 n = 32	Arm 3 n = 29	3 vs 1 p OR (CI)	2 vs 1 p OR (CI)	3 vs 2 p OR (CI)
ACRPEdi30(%)(CI)	92.2 (82,1-102.4)	84.4 (71.2-97.5)	96.6 (89.8-103.3)	0.1 1.7 (0.8-3.4)	0.8 0.9(0.5-1.7)	0.1 0.9-3.7
ACRPedi50 (%)(CI)	85.5 (72.4-98.6)	83.8 (70.1-97.4)	93.1 (83.7-102.4)	0.3 1.4(0.7-2.8)	0.9 1.1(0.6-2.0)	0.4 1.3(0.7-2.7)
ACRPedi70 (%)(CI)	69.0 (52.1-85.9)	68.8 (51.6-85.9)	82.8 (68.8-96.8)	0.07 1.8(1.0-3.3)	0.7 0.9(0.4-1.7)	0.03 2.1(1.1-4.0)
Inactive disease (%)	61.0 (39.7-82.3)	63.1 (43.6-82.7)	61.0 (40.9-81.2)	0.9 1.0(0.7-1.7)	0.1 0.7(0.4-1.1)	0.09 1.5(0.9-2.5)
JADAS-10 (CI)	2.6(1.4-3.8)	4.0(2.2-5.8)	3.0(1.6-4.4)	0.6 0.6(0.1-3.5)	0.2 3.1(0.5-20.3)	0.09 0.2(0.03-1.3)

**Conclusion:** Although our study did not meet its primary end point, treat-to-target treatment in this cohort of children with recent-onset JIA resulted in high frequencies of clinical inactive disease and adjusted ACRpedi30/50/70 scores in all three arms. In clinical trials inactive disease seems a feasible goal in juvenile idiopathic arthritis patients.

Trial registration identifying number: NTR1574

**Disclosure of Interest:** None Declared.

### O17 Predictive value of magnetic resonance imaging in patients with juvenile idiopathic arthritis in clinical remission

#### Marta Mazzoni^1^, Angela Pistorio^2^, Stefania Viola^3^, Alessia Urru^1^, Emanuela Sacco^3^, Eleonora Zaccheddu^3^, Francesca Magnaguagno^4^, Angelo Ravelli^1^, Alberto Martini^1^, Clara Malattia^1^

##### ^1^Università degli studi di Genova, Pediatria II-Reumatologia, Genoa, Italy; ^2^Epidemiologia, Biostatistica e Comitati, Genoa, Italy; ^3^Pediatria II-Reumatologia, Genoa, Italy; ^4^Radiologia, G. Gaslini, Genoa, Italy

###### **Correspondence:** Marta Mazzoni

**Introduction:** MRI studies on RA patients showed that subclinical synovitis is often present in patients in clinical remission and is responsible for progression of joint damage. A high frequency of MRI-detected inflammation in JIA patients with clinically inactive disease was also reported. Due to the lack of longitudinal studies in JIA, it is unclear whether this phenomenon entails a risk of progression of joint damage and whether it should affect the therapeutic decisions.

**Objectives:** To assess the prevalence of subclinical synovitis as detected by MRI in a cohort of JIA patients in clinical remission and to evaluate its association with disease flare and structural damage progression.

**Methods:** All JIA patients who met the Wallace criteria for clinical remission and underwent contrast-enhanced MRI at the Study Unit between 2007 and 2015 were included. MRIs were scored by two independent readers according to the Outcome Measure in Arthritis Clinical Trials (OMERACT) Rheumatoid Arthritis Scoring System (RAMRIS). Joint damage progression was assessed by conventional radiography (CR) according to the adapted versions of the Sharp/van der Heijde score and to the Childhood Arthritis Radiographic Score of the Hip. The concordance between the readers was assessed using kappa statistics. Categorical data were analyzed using chi-squared test and Fisher’s exact test. Comparison of quantitative variables was performed by the non-parametric Mann–Whitney U-test. A logistic regression model was applied to perform multivariate analysis of the radiographic damage risk factors.

**Results:** A total of 90 patients (15 M, 75 F; mean age 13.8 years; mean disease duration 8.5 years; mean follow-up duration 2.9 years) were included. Fourteen out of 90 patients (15.6%) were in remission off medication, while 76/90 patients (84.4%) were in remission on medication. Forty-five patients were assessed by MRI in the wrist, 30 in the hips, 13 in the ankle and 2 in the knee. Fifty-seven patients (63.3%) had evidence of subclinical synovitis on MRI. The inter-observer agreement for presence/absence of synovitis was good (k = 0.74; 95% CI: 0.5-0.9). Forty-three out of 57 patients (75,4%) with subclinical synovitis experienced a disease flare versus 11 out of 33 patients (33.3%) who hadn't any synovial inflammation (*P* < 0.0001). Radiographic damage progression was assessed in 54/90 patients for whom follow-up CRs were available and was detected in 19/54 patients (35.2%). A significant association between systemic JIA subtype and deterioration of joint damage was found (*P* = 0.027, Fisher’s exact test). MRI-detected bone marrow oedema (BMO) score and the baseline radiographic damage score were also significantly related to structural progression (*P* = 0.002, Mann–Whitney U-test). The multivariable logistic regression analysis showed that only baseline BMO score ≥3 independently contributed to explain radiographic damage progression (OR 4.82; 95% CI: 1.0-23.2; *P* = 0.035).

**Conclusion:** A sizeable proportion of patients in clinical remission had MRI evidence of persistent joint inflammation. Subclinical synovitis was significantly associated with disease flare, while BMO showed remarkable promise in predicting joint destruction. These findings support the utility of MRI for the assessment of JIA patients in clinical remission and may have important clinical implications for their management.

**Disclosure of Interest:** None Declared

### O18 Baseline predictors of functional disability eight years after disease onset in the nordic juvenile idiopathic arthritis population-based cohort

#### Veronika Rypdal^1^, Ellen D Arnstad^2^, Lillemor Berntson^3^, Marek Zak^4^, Kristiina Aalto^5^, Suvi Peltoniemi^5^, Susan Nielsen^4^, Mia Glerup^6^, Troels Herlin^6^, Anders Fasth^7^, Maria Ekelund^8^, Marite Rygg^2^, Ellen Nordal^1^ and Nordic Study Group of Pediatric Rheumatology (NoSPeR)

##### ^1^Dep of Pediatrics, University Hospital of North Norway, UiT The Arctic University of Norway, Tromsø; ^2^Dep of Laboratory Medicine, Children’s and Women’s Health, NTNU, Dep of Pediatrics, St.Olavs Hospital, Trondheim, Norway; ^3^Dep of Women’s and Children’s Health, Uppsala University, Uppsala, Sweden; ^4^Pediatric Clinic II, Rigshospitalet, Copenhagen, Denmark; ^5^Dep of Pediatrics, Helsinki University Hospital, Helsinki, Finland; ^6^Dep of Pediatrics, Århus University Hospital, Århus, Denmark; ^7^Dep of Pediatrics, University of Gothenburg, Gothenburg, Sweden; ^8^Dep of Pediatrics, Ryhov County Hospital, Dep of Women’s and Children’s Health, Uppsala University, Uppsala, Sweden

###### **Correspondence:** Veronika Rypdal

**Introduction:** Baseline clinical predictors on long-term outcome, not only regarding remission but also on functional ability, may enable assessment of prognosis and guide early treatment decisions in juvenile idiopathic arthritis (JIA).

**Objectives:** To evaluate potential baseline clinical predictors of functional ability assessed by the Childhood Health Assessment Questionnaire (CHAQ) eight years after disease onset.

**Methods:** Consecutive cases with newly diagnosed JIA from defined geographical areas in Denmark, Finland, Sweden and Norway were included and followed over eight years. Logistic regression was performed to assess potential baseline predictors of disability in terms of CHAQ (or HAQ for participants ≥18 years of age) score >0 at the final study visit.

**Results:** At follow-up 98 months (median, IQR 95-102) after disease onset, CHAQ/HAQ was available in 352 (80%) of the total 440 participants. A CHAQ/HAQ score of 0 was found in 239 (68%) of these. The following characteristics at the first study visit 7 months (median, IQR 6-8) predicted CHAQ/HAQ > 0 eight years after disease onset: higher cumulative joint count, symmetric arthritis in wrists, fingers, or ankles, higher score on physician and patient/parent global assessment of disease activity, pain, CHAQ and morning stiffness (Table 3).

**Table 3:** **(abstract O18).** Baseline characteristics analysed as predictors of disability eight years after disease onset

Clinical Characteristics	N	CHAQ/HAQ =0	CHAQ/HAQ >0	OR (95%CI)	p
Cumulative active joint count	352	3 (1-6)	5 (2-9)	1.1 (1.0-1.1)	0.001
Physician GA, VAS	210	1.0 (0.3-2.0)	2.0 (0.7-4.3)	2.5 (1.5-4.0)	< 0.001
Symmetric wrist arthritis, n (%)	351	28 (50.0)	28 (50.0)	2.5 (1.4-4.5)	0.002
Symmetric finger arthritis, n (%)*	351	20 (39.2)	31 (60.8)	4.2 (2.3-7.8)	< 0.001
Symmetric ankle arthritis, n (%)	351	50 (57.5)	37 (42.5)	1.9 (1.1-3.1)	0.02
CHAQ- score at baseline	239	0.1 (0.0-0.9)	0.6 (0.0-1.4)	2.0 (1.4-3.0)	0.001
Patient/parent GA, VAS	234	0.7 (0.0-2.3)	2.0 (1.0-4.0)	2.3 (1.5-3.6)	< 0.001
Pain VAS	231	0.7 (0.0-2.3)	2.7 (1.0-5.0)	3.2 (2.0-5.0)	< 0.001
Morning stiffness > 15 min, n (%)	276	41 (41.4)	58 (58.6)	5.2 (3.0-8.9)	<0.001

Values are the median (IQR) unless otherwise indicated; OR, odds ratio; CI, confidence interval. * Symmetric finger arthritis: bilateral arthritis in 1 or more of the 14 finger joints; GA, global assessment; VAS, visual analogue scale range 0-10

**Conclusion:** A higher cumulative joint count, and specifically symmetric arthritis in finger joints, morning stiffness and higher pain score, higher patient/parent and physician global VAS score at baseline, predicts functional disability eight years after disease onset in a population-based Nordic JIA cohort.

**Disclosure of Interest:** None Declared.

### O19 What is the outcome of juvenile idiopathic arthritis in adulthood? The monocentric experience of 414 patients followed in a transition tertiary clinic of rheumatology

#### Irene Pontikaki^1,2,3^, Lorenza Maria Argolini^4^, Tania Ubiali^4^, Maria Gerosa^2,3^, Carolina Artusi^4^, Marcello Truzzi^5^, Roberto Viganò^6^, Antonella Murgo^2,3^, Orazio De Lucia^2,3^, Pierluigi Meroni^2,3^

##### ^1^Center of Pediatric Rheumatology, Milan, Italy; ^2^Rheumatology, ASST Pini/CTO, Milan, Italy; ^3^Chair of Rheumatology, Milan, Italy; ^4^Rheumatology, University of Milan, Milan, Italy; ^5^Rheuma Surgery, Milan, Italy; ^6^Division of Rheumatoid Arthritis Surgery, ASST Pini/CTO, Milan, Italy

###### **Correspondence:** Irene Pontikaki

**Introduction:** Juvenile Idiopathic Arthritis (JIA) is a chronic inflammatory disease that affects children and adolescents and shows many differences in clinical manifestations, assessment and management compared to adult-onset arthritis. The transition from the child-centered to the adult-oriented care is a challenging multidimensional process that emphasizes a lot of aspects that need to be addressed.

**Objectives:** To describe the long-term outcome of JIA.

**Methods:** Four-hundred and fifteen patients affected by JIA and referred to a transition care rheumatology tertiary centre were considered between 1999 and 2016. The outcome assessment included disease activity, mean disease duration, medications, number of prosthesis implantation, pregnancies, mortality, social integration (mobility, employment status and educational level).

**Results:** A hundred and twenty (28.9%) males and 294 (71%) females were included; 58 (14%) patients were lost to follow up. The median age of the patients was 25 (18-57) years, the median age at onset was 9 years and the average disease duration was 17 years. Subtypes of JIA at disease onset included oligoarthritis 212 (51.2%), polyarthritis 98 (23.6%), systemic arthritis 51 (12.3%), psoriatic arthritis 11 (2.7%), enthesitis related arthritis 41 (9.9%) and undifferentiated arthritis 1 (0.2%). Seventy-one (17.1%) patients had persistent uveitis. Eighty-five implant prosthesis and 15 arthrodesis were recorded. Sixty-eight patients (16.4%) were also referred to the ultrasonography-guided infiltration clinic, to receive either intrarticular steroids or hyaluronic acid. At follow up 180 (43.5%) had low active disease activity, 84 (20.3%) had moderate disease activity, 14 (3.4%) had a high disease activity, 72 (17.4%) were on remission on medication and 64 (15.5%) off medication. Among the 350 patients still on medication, 75 (21.4%) were under treatment with oral steroids, 200 (57.1%) with sDMARDs and 225 (64.3%) with bDMARDs. Five deaths (1.2%) occurred in this cohort. A hundred and eighty-one (43.7%) subjects had a higher educational level (university), 294 (71%) had an employment, 243 (58.7%) obtained a driving license. Twenty-one (5.1%) pregnancies were registered. The transition age was considered after the age of sixteen years old. Particular attention was brought to the multidisciplinary approach towards each patient, that was realized with the collaboration of other specialists (ophthalmologist, orthopedic, dermatologist, gastroenterologist, obstetric and psychologist).

**Conclusion:** In the era of biologic therapy the long-term outcome of JIA underwent an outstanding improvement regarding a lot of variables. Two hundred and twenty-five (54.3%) patients were still on tight control, not only because of the continuation of the biological therapy, but also owing to the multidisciplinary care carried out even during remission. JIA often persists over the adulthood, therefore the long term follow-up and care of these patients needs to be conducted by a rheumatologist expertized in JIA in collaboration with other specialists.

**Disclosure of Interest:** None Declared.

### O20 Identification of novel antibodies predictive of the development of uveitis in jia using high-density nucleic acid programmable protein arrays

#### Madeleine Rooney^1^, David Gibson^2^, Ji Qui^3^, Sorcha Finnegan^1^, Joshua Labaer^3^

##### ^1^Center Experimental Medicine, Queens University Belfast, Belfast, United Kingdom; ^2^NI Centre of Stratified Medicine, Ulster University, Londonderry, United Kingdom; ^3^Center for Personalized Diagnostics, Arizona State University, Tempe, AZ United States

###### **Correspondence:** David Gibson

**Introduction:** Currently all children with oligo and polyarticular JIA have to be screened by ophthalmologists for years in order to ensure that this completely asymptomatic disease is not missed. If missed or inadequately treated, some 50% of these children can go functionally blind. The only useful biological marker currently available is ANA status. However this is neither sensitive nor specific enough to significantly alter regular clinical screening. It does however suggest that autoantibodies, as yet undiscovered, may be important in the pathophysiology of this disease.

We undertook this proof of concept study to see whether a novel technique capable of producing multiple proteins could be used to screen for autoantibodies that are associated with uveitis development in JIA patients. This could enable us to identify children, at the time of diagnosis of arthritis, who are highly likely to develop uveitis.

**Objectives:** To Identify novel antibodies that predict the development of uveitis in children with JIA.

**Methods:** Nucleic Acid Programmable Protein Arrays (NAPPA) enable the *in situ* production of multiple proteins from DNA templates which are immobilised on a solid phase. Methodology is described in detail elsewhere [1]. NAPPA slides with 2200 genes were produced. Pubmed search identified ~60 genes associated with uveitis pathology and ~30 genes associated with arthritis development. The remaining ~2100 genes were randomly identified from a ~12000 human gene collection (http://dnasu.asu.edu). The arrays were then probed using plasma from JIA patients with (n = 20) and without uveitis (n = 20) and from healthy age and sex matched controls (n = 20). Proteins with higher levels of antibody detected were confirmed by ELISA.

**Results:** NAPPA image analysis revealed distinct signals from plasma antibodies of JIA patients with Uveitis which had reacted with specific array proteins. Hierarchical clustering heat maps were used to visualize clusters of proteins with higher array reactivity for JIA or Uveitis samples, realtive to controls. ELISA's were developed for nine highly reactive proteins including BATF, FO5, PROSAPIP1, CCND1, NOUFV3 and SSB. Antibodies specific for single-stranded DNA-binding proteins (SSB) were confirmed to be at significantly higher concentrations in the plasma of JIA patients with uveitis, relative to JIA patients without eye disease.

**Conclusion:** These results indicate that the NAPPA technique is a sensitive tool for screening antigens including specific nuclear antigens which distinguish JIA patients with uveitis. We plan on developing a nuclear antigen array with over 2000 features to determine further uveitis patient reactivity. Prospective studies are also required to establish if these immunoglubulins are present at detectable levels in JIA patients prior to development of uveitis and to assess their predictive utility.

Reference

[1]: Bian, X., Wiktor, P., Kahn, P., Brunner, A., Khela, A., Karthikeyan, K., Barker, K., Yu, X., Magee, M., Wasserfall, C. H., Gibson, D., Rooney, M. E., Qiu, J., and LaBaer, J. (2015) Antiviral antibody profiling by high-density protein arrays. Proteomics 15, 2136–2145

**Disclosure of Interest:** None Declared.

### O21 New onset uveitis in patients with juvenile idiopathic arthritis under biological therapy

#### Irina Nikishina^1^, Olga Kostareva^1^, Maria Kaleda^1^, Svetlana Rodionovskaya^1^, Svetlana Arsenyeva^1^, Anna Shapovalenko^1^, Ekaterina Denisova^2^

##### ^1^Pediatric, V.A. Nasonova Research Institute of Rheumatology, Moscow, Russian Federation; ^2^Pediatric, Helmgoltz Moscow Research Institute of Eye Diseases, Moscow, Russian Federation

###### **Correspondence:** Irina Nikishina

**Introduction:** Biological agents (BA), especially TNF inhibitors, are high efficacy options for current therapy for patients (pts) with juvenile idiopathic arthritis (JIA). They are successfully used not only for the arthritis but also for JIA-associated uveitis, however, development of uveitis *de novo* in pts treated with BA is a well-established paradoxical adverse event.

**Objectives:** to evaluate the frequency of new onset (*no-*) uveitis, occurring under BA therapy in JIA pts, to establish clinical features, which may be associated with development of such phenomenone.

**Methods:** retrospective cohort study involved all JIA pts (760) who were treated with BA in our clinic from 2004 to 2017. All cases of *no-*uveitis were collected for the describing of their clinical features in disease onset and course, activity level, JIA category, exposure to Methotrexate (MTX) and BA, presence of ANA, HLA B27.

**Results:** among of 760 pts treated with different BA we identified 22 (2.9%) pts (12 female/10 male) with *no-*uveitis under BA, mostly during etanercept (ETA) therapy (22 cases from 302 ETA courses, 6.6%), 1/120 - in abatacept (ABA) and 1/249 - in adalimumab (ADA). There are no cases of *no-*uveitis under other BA Frequency of *no-*uveitis is much higher in ETA group (2.46 events per 100 patient-year (PY) vs 0.31 in ABA, and 0.15 in ADA. ETA exposure was 14.7 ± 9.7 months (mo).. A case of *no-*uveitis under ABA was observed after 5 mo of therapy in a girl previously treated with infliximab. 1 boy with early JIA onset (in age of 18 mo) developed *no-*uveitis after 71 mo of ADA 71 mo simultaneously with general response decreasing and successfully switched to ABA. JIA subtypes were as follows: RF-neg polyarthritis 6 (27%), persistent oligoarthritis 3 (14%), extended oligoarthritis 11 (50%), enthesitis-related arthritis (ERA) - 2 (9%). Average age at JIA onset was 4.5 ± 3.9 yrs. 19/22 patients had high laboratory activity (CRP 54 ± 23 mg/l; ESR 41 ± 19 mm/h) and severe arthritis before BA initiation. However most of pts (18/22) achieved 90-100% ACRpedi-response by the uveitis development. 14/22 pts were ANA-positive, 11/22 pts had HLAB27, including 3 pts who had the both features. Uveitis was occurred earlier in ANA plus HLAB27 positive pts (mean exposure - 10.7 mo) than in only ANA-positive or HLAB27-positive pts (27.4 mo and 21.6 mo accordingly). 19/22 (86%) of pts received methotrexate (MTX) in mean dosage 11.5 mg/m^2^/week. There are no differences in time of uveitis development depending of MTX. In all cases of *no-*uveitis BA was switched.

**Conclusion:** Our study suggested that new onset of uveitis is rare adverse event during BA therapy in JIA without any known predisposing risk factors.. Such paradoxical effects may reflect not so much therapy complications, but the delayed implication of natural character of disease. Development of *no-* uveitis is more often observed in pts receiving ETA, especially in pts with early age of JIA onset. High activity aggressive manifestations at the disease onset and good initial response to BA are typical features for all pts, who developed this paradoxical effect under BA therapy.

**Disclosure of Interest:** None Declared

### O22 Microparticles as potential biomarkers of disease activity in anti-neutrophil cytoplasmic antibody – associated vasculitis

#### Milena Kostić^1^, Fariborz Mobarrez^2^, Jelena Vojinović^1^, Iva Gunnarsson^2^, Aleksandra Antović^2^

##### ^1^Department of Pediatrics, Clinical Center, Nis, Serbia; ^2^Department of Medicine, Rheumatology Unit, Karolinska Institutet, Stockholm, Sweden

###### **Correspondence:** Milena Kostić

**Introduction:** Microparticles (MPs) are irregularly shaped submicron vesicles, which are released from plasma membrane upon cell activation and during the early phase of apoptosis. Increased levels of circulating MPs (mainly of endothelial cell origin, but also platelet derived) correlates with autoinflammatory disease activity, such as anti-neutrophil cytoplasm antibody (ANCA) – associated vasculitis (AAV).

**Objectives:** To evaluate levels of activity markers expressed on surface of MPs, during active disease and remission, compared to healthy control subjects.

**Methods:** Our study included 48 AAV patients (24 with active and 24 with inactive disease) and 23 healthy control subjects (age and gender matched). We analyzed the number and phenotype of MPs in plasma identified by flow cytometry, via labeling with following monoclonal antibodies: CD142 (tissue factor-TF), anti-H3cit (citrulinated H3 directed against neutrophil extracellular traps-NETs), anti-pentraxin3 (pentraxin3), HMGB1 (high mobility group box 1 protein-HMGB1), anti-TWEAK (tumor necrosis factor-like weak inducer of apoptosis-TWEAK), anti-plasminogen (plasminogen), anti-C3a (C3a) and anti-C5a (C5a). The assessment of vasculitis disease activity was performed using the Birmingham Vasculitis Activity Score (BVAS), while active disease was defined as BVAS ≥1 and inactive (remission) as BVAS = 0. Statistical analysis was performed using GraphPed software.

**Results:** Half of the patients group (24) had active vasculitis (mean BVAS 6,69 ± 8,76) and 24 had inactive disease. Plasma levels of MPs expressing TF, NETs, pentraxin3 and HMGB1 in active patients were significantly higher than in those in remission and healthy controls (p < 0.01, p < 0.0001, respectively). MPs expressing C5a and C3a were significantly higher in both patients groups compared to controls (p < 0.001). Additionally, levels of MPs expressing C5a strongly correlated with BVAS (r = 0.78, p < 0.0001), while there was no significant correlation between other explored markers and BVAS. Data presented in Table 4.

**Table 4:** **(Abstract O22).** Microparticles (MPs) levels in AAV patients with active or inactive disease and healthy controls

	Active AAV		Inactive AAV		Healthy controls		p-value (respectively)
	median	IQR	median	IQR	median	IQR	
Tissue factor	265	247	174	107	105	84,5	p < 0.01^*^ p < 0.001^**^
NETs	112	24	103	18	44	35	p < 0.05^*^ p < 0.0001^**^ p < 0.001^***^
Pentraxin3	1332	1810	347	294,5	184	146,5	p < 0.01^*^ p < 0.0001^**^ ns^***^
HMGB1	684	452,5	380	258	77	61,5	p < 0.01^*^ p < 0.001^**^ p < 0.001^***^
TWEAK	131	83	141	102,5	67	53	ns^*^ p < 0.0001^**^ p < 0.05^***^
Plasminogen	86	23	89	23	44	33,5	ns^*^ p < 0.001^**^ p < 0.01^***^
C3a	201	91	147	87	89	72	ns^*^ p < 0.001^**^ p < 0.05^***^
C5a	131	86,5	111	65	51	30	ns^*^ p < 0.001^**^ p < 0.01^***^

^*^between active and inactive, ^**^ between active and controls, ^***^between inactive and controls

**Conclusion:** Our results support recently postulated potential role of complement system in AAV pathogenesis and disease activity. Evaluated proteins expressed on MPs, especially C5a, could be used as potential biomarkers which might reflect inflammation and disease activity in AAV patients. Further investigations are necessary to confirm our preliminary results and to validate the most promising biomarker in AAV.

**Disclosure of Interest:** None Declared.

### O23 Plasmalemma vesicle-associated protein 1 (pv-1) as a marker of active disease in childhood vasculitis.

#### Andrea Taddio^1^, Sarah Abu Rumeileh^2^, Claudia Bracaglia^3^, Luigina De Leo^4^, Rebecca Nicolai^3^, Fabrizio De Benedetti^3^, Alberto Tommasini^4^, Samuele Naviglio^4^, Serena Pastore^4^, Alessandro Ventura^1^, Tarcisio Not^1^

##### ^1^Institute for Maternal and Child Health - IRCCS "Burlo Garofolo" - and University of Trieste, Trieste, Italy; ^2^University of Trieste, Trieste, Italy; ^3^Division of Rheumatology, IRCCS Ospedale Pediatrico Bambino Gesù, Rome, Italy; ^4^Institute for Maternal and Child Health - IRCCS "Burlo Garofolo" -, Trieste, Italy

###### **Correspondence:** Andrea Taddio

**Introduction:** The endothelial protein Plasmalemma Vesicle-associated protein 1 (PV-1) is the main component of stomatal and fenestral diaphragms of blood vessels. It regulates permeability, leukocyte migration and angiogenesis [1]. Considering the central role of the endothelium in vasculitis pathogenesis and the lack ofspecific biomarkersfor the clinical diagnosis and management of this group of complex disorders, we have investigated the levels of PV-1 among children with vasculitis and their association with disease activity or remission.

**Objectives:** To determine, for the first time, serum levels of endothelial protein PV-1 in a population of patients with vasculitis and healthy controls and possibly to assess the role of PV-1 to serve as a diagnostic biomarker in vasculitis disorders in childhood and/or to monitor disease activity and severity.

**Methods:** A case-control matched for age study was conducted including 62 healthy young people and 35 children with active vasculitis and other inflammatory diseases with vascular involvement: 18 patients with Pediatric Systemic Lupus Erythematosus (pSLE), 9 with Juvenile Dermatomyositis (JDM), 2 with Kawasaki Disease, 2 with leukocytoclastic vasculitis, 2 glomerulonephritis, 1 Central Nervous System Vasculitis, 1 Henoch-Schönlein Purpura (HSP). PV-1 serum levels were also measured in 9 children in clinical remission (2 SLE, 2 JDM, 3 HSP, 1 Kawasaki disease, 1 Hughes-Stovin Syndrome). Measurement of PV-1in blood serum (expressed in ng/ml) was performed using ELISA assay following the manufacturer’s (Biomatik) instructions.

**Results:** Patients with active disease showed significantly higher concentrations of PV-1 than healthy controls (p < 0.001) and patients with inactive disease (p < 0.001) (Table 5). Optimal serum PV-1 cut-off to identify patients with active vasculitis was found to be 0.78 ng/ml, which achieved on receiver operating characteristic (ROC) curve analyses an accuracy of 0.90, with 74% sensitivity and 92% specificity. Different diseases did not show significant differences regarding levels of PV-1.

**Table 5:** **(abstract O23).** See text for description

Patients	Controls N = 62	Acute Vasculitis N = 35	Remission vasculitis N = 9
PV – 1 (ng/ml) Median (interquartile range)	0.221 (0.082 – 0.436)	1.818 (0.746– 3.970)	0.316 (0.203 – 0.606)
p value		< 0.001	< 0.001

**Conclusion:** PV-1 could represent a specific marker of disease activity in vasculitis disorders in childhood.

1. Guo L, Zhang H, Hou Y, et al. Plasmalemma vesicle–associated protein: A crucial component of vascular homeostasis (Review). Exp Ther Med 2016; **12**:1639–44.

**Disclosure of Interest:** None Declared.

### O24 Takayasu arteritis in childhood: retrospective experience from a tertiary referral center in South India

#### Sathish Kumar^1^, Ruchika Goel^2^, Debashish Danda^2^

##### ^1^Pediatric Rheumatology, Department of Pediatrics, Christian Medical College, Vellore, India; ^2^Department of Clinical Immunology and Rheumatology, Christian Medical College, Vellore, India

###### **Correspondence:** Sathish Kumar

**Introduction:** Takayasu arteritis (TAK) is an idiopathic large-vessel vasculitis affecting the aorta and its major branches. Although the disease rarely affects children, it does occur, even in infants. The untreated inflammation often leads to narrowing or ectasias of aorta and its direct branches with or without pulmonary and coronary involvement. It’s a rare medical condition but we in our tertiary care center have collected the second largest single center series of patients over the past 16 years.

**Objectives:** To evaluate the clinical features, disease activity, treatment and outcome of childhood TAK in a tertiary center in South India

**Methods:** We analyzed a retrospective case series of children fulfilling the TAK classification criteria of the European League against Rheumatism and the Paediatric Rheumatology European Society. Data has been collected in a retrospective manner from our institute’s electronic medical records (EMR) for childhood onset TAK. Data regarding demographics, clinical features, treatments, and outcomes were recorded. Baseline data including age, gender, disease duration, angiographic type, the extent of clinical disease using DEI.Tak, CRP, and ESR were collected. Disease activity was assessed by a composite clinical score namely ITAS-A (CRP). At each visit, active disease was defined as

1. **Clinical activity** by the presence of any one of the following:

1a. ITAS ≥ 2 (not attributable by in-stent restenosis) or

1b. ITAS-A (CRP) ≥ 3, but at least one point should be contributed by clinical criteria as in ITAS proforma

2. **Activity by imaging** by presence of de Novo lesion on follow-up angiography or stenosis of the same vessel extending beyond stent margins

3. **Laboratory criteria** of activity were defined by persistently raised CRP as well as ESR on 2 consecutive visits without any alternative explanation like infection

**Results:** 100 children with juvenile-onset TAK were included in this study over the past 16 years. Out of 100 children with TAK, 30 were male and 70 were female. Median age at onset was 14 years and mean duration of delay in diagnosis was 7 months (4-24 months). The most common clinical features at presentation were pulse abnormality (67%) followed by arterial hypertension (62%), claudication (40%) and systemic symptoms like fever and fatigue (36%).

Past, present history of tuberculosis or tuberculosis after treatment was seen in 6 patients while 2 patients had been treated empirically for TB. At presentation, median ITAS 2010 score was 9 (4-15), DEI. Tak was 9 (6-13) and TADS was 7 (4-11).

The full angiographic profile was performed for 97 patients. Rest of the patients had limited area imaging. Type 5 was the commonest followed by type 4 and type 1. Aneurysms or ectasias with or without stenosis seen in 7 patients

Treatment included corticosteroids (85%), combined with MMF in most cases (51), azathioprine(15), methotrexate (9) and tocilizumab (5) were reserved for severe and/or refractory cases. Mean follow-up duration was 33 months (12-61). Delta TADS calculated in 75 patients was 0 (0-1) and last visit ITAS and ITAS-A CRP was 0 (0-1). Disease course was persistently stable in 39, relapsing in 16, persistent active in 5 children. 1 child expired during follow-up.

**Conclusion:** Improved awareness of TAK is essential to secure a timely diagnosis. The initial symptoms and signs are non-specific, and a high index of suspicion is needed if the diagnosis is to be made. The investigation and management of Takayasu’s disease can prove difficult.

**Disclosure of Interest:** None Declared.

## Oral Presentations 2

### O25 Single cell RNA-sequencing of bone marrow macrophages identifies a distinct subpopulation in systemic JIA with features of interferon response, endocytic vesicles and phagocytosis

#### Grant Schulert^1^, Nathan Salomonis^2^, Sherry Thornton^1^, Alexei Grom^1^

##### ^1^Pediatric Rheumatology, Cincinnati Children's Hospital Medical Center, Cincinnati, OH, United States; ^2^Biomedical Informatics, Cincinnati Children's Hospital Medical Center, Cincinnati, OH, United States

###### **Correspondence:** Grant Schulert

**Introduction:** Macrophage activation syndrome (MAS) is a life-threatening complication of systemic juvenile idiopathic arthritis (SJIA), characterized by activation and expansion of cytolytic lymphocytes and macrophages with hemophagocytic properties. Recent work by us and others has shown that emergence of MAS is associated with a surge in IFN-gamma and IFN-induced chemokines; however, previous gene expression studies failed to demonstrate this IFN-induced signature in peripheral blood cells. However, prior studies in SJIA and MAS have been limited by a failure to examine key myeloid effector cells, specifically activated macrophages or histiocytes which traffic to tissue including bone marrow during MAS.

**Objectives:** Utilize single-cell RNA-sequencing to identify specific gene expression signatures of bone marrow macrophage populations in SJIA and MAS.

**Methods:** Cell sources include unused portions of bone marrow aspirates obtained as part of a diagnostic workup and interpreted as normal were obtained from the Cincinnati Children’s Hospital Immunopathology lab, as well as cells obtained from a bone marrow biopsy from a newly diagnosed SJIA patient. Macrophage single cell suspensions were obtained using cell sorting for populations expressing the monocyte and macrophage surface markers CD14 and CD163 while excluding cells expression the granulocyte/monocyte marker CD15, prior to loading onto the Fluidigm C1 Single-Cell Auto Prep System. Extracted RNA was converted into cDNA and sequenced as a pooled library, and aligned to the human Ensembl transcriptome using Kallisto through AltAnalyze version 2.1.1.

**Results:** Three independent control samples yielded 180 single cells which passed quality-control filtering. While there was substantial inter-individual variability, using principle component analysis and Iterative Clustering and Guide-gene Selection, a core set of approximately 1400 genes were identified that contributed to the heterogeneity of normal bone marrow macrophage population. Control macrophages formed at least three primary cellular clusters, which were distinguished based on expression of genes associated with inflammatory responses, GM-CSF signaling, and aurora B signaling. 61 single bone marrow macrophages were captured from a patient with newly diagnosed SJIA with active systemic features, arthritis, marked anemia and relative thrombocytopenia, but lacking other overt signs of MAS, who underwent bone marrow biopsy as part of the diagnostic evaluation. Expression profiles were broadly similar to control macrophages by PCA, and all macrophage clusters were represented. However, a distinct subpopulation of bone marrow macrophages from the SJIA patient was identified that exhibited markedly altered transcriptional profiles. Compared to other macrophages within the cluster, this SJIA macrophage population showed alterations in gene pathways including cellular response to interferon gamma (adjusted p = 1.35e-14), endocytic vesicle membranes (p = 8.44E-14), phagosome (p = 2.98e-9) and vesicle-mediated transport (p = 1.05E-07). These cells showed a proinflammatory gene expression signature, including significant enrichment for genes regulated by NF-kB and STAT1. This included S100A12, which is highly expressed in patients with SJIA and MAS and was markedly elevated in serum from this patient.

**Conclusion:** We identify a distinct subpopulation of bone marrow macrophages in an SJIA patient with features associated with emergence of MAS, including interferon response, phagocytosis and vesicular transport. This demonstrates the importance of studying these effector cells at the sites of inflammation, and suggests that tissue macrophages may be a key source of IFN-induced products during MAS. Together, these data show that single cell gene expression profiling of bone marrow macrophages can identify reproducible cellular clusters as well as potential biologically relevant subpopulations and pathways perturbed during inflammatory disorders.

**Disclosure of Interest**: G. Schulert Consultant for: Novartis, N. Salomonis: None Declared, S. Thornton: None Declared, A. Grom Grant/Research Support from: NovImmune, AB2Bio, Consultant for: Novartis, Juno.

### O26 Adjudication of infections from the pharmacovigilance in juvenile idiopathic arthritis patients (pharmachild) treated with biologic agents and/or methotrexate with a focus on opportunistic infections

#### Gabriella Giancane, Joost Swart, Elio Castagnola, Andreas Groll, Gerd Horneff, Hans-Iko Huppertz, Dan Lovell, Tom Wolfs, Michael Hofer, Ekaterina Alexeeva, Violeta Panaviene, Susan Nielsen, Jordi Anton, Florence Uettwiller, Valda Stanevicha, Maria Trachana, Fabrizio De Benedetti, Constantin Ailioaie, Elena Tsitsami, Sylvia Kamphuis, Troels Herlin, Pavla Dolezalova, Gordana Susic, Berit Flato, Flavio Sztajnbok, Elena Fueri, Francesca Bovis, Francesca Bagnasco, Angela Pistorio, Alberto Martini, Nico Wulffraat, Nicolino Ruperto

##### Pediatria II, Reumatologia; PRINTO, Istituto Giannina Gaslini, GENOA, Italy

###### **Correspondence:** Gabriella Giancane

**Introduction:** Pharmachild is a pharmacovigilance registry on children with JIA treated mainly with biologics ± methotrexate (MTX). Little evidence exists in literature about the role of JIA or its immunosuppressive therapy in determining infections, especially caused by opportunistic pathogens.

**Objectives:** To provide an update on opportunistic infections (OI) revised by an independent Safety Adjudication Committee (SAC) (3 pediatric rheumatologists and 2 pediatric infectious disease specialists).

**Methods:** The participating centres were asked to report all infections encountered by their JIA patients. PRINTO and the medical monitor (MM) classified events based on MedDRA dictionary. Moderate/serious/severe/very severe infections were then revised blindly by the SAC, who were asked to answer 6 questions. The events with consensus of at least 3/5 experts on the first 3 questions (‘Is this an infection?’, ‘Is it common?’, ’Is it opportunistic?’) were retained for the analysis. With referral to the recommendations by Withrop *et al.* ^1^, for the first time a list of opportunistic infections in children with JIA on immunosuppressive therapy was elaborated and approved by consensus, through three Delphi steps.

**Results:** A total of 772 safety events related to 634 patients were submitted to the Safety Adjudication Committee. 689 (89.2%) events received consensus among the experts on the 3 questions and, of these, 682 (99.0%) were considered as infections, corresponding to 53 High Level Term (HLT) including 153 different Preferred Terms (PT), according to MedDRA dictionary. Among the 682 infections, 603 (88.4%) were defined by the experts as common and 119 (17.4%) as opportunistic. For 92 (60%) of the 153 PT, the MM and SAC used the same PT, while the remaining 40% was adjudicated by a third examiner, who analyzed again the case reports and assigned the PT which was the most appropriate taking into account the experts’ opinion. A final number of 52 HLT emerged and, among them, herpes viral infections, tract respiratory infections and EBV were the most frequent (Table 6). Analyzing the infections by PT, 151 different PT resulted. Of them, the experts adjudicated: 22 as OI, 117 as not OI, 8 discordant and 4 not evaluable. Comparing the experts’ adjudication with the approved list of OI by PT, there was full agreement for the 22 PT classified as OI, while 26/117 (22.2%) PT resulted in the list, but were not classified as OI by the experts.

**Table 6:** **(Abstract O26).** The most frequent HLT for the 682 infections with agreement of at least 3/5 experts on the first 3 questions. (N: number of infections)

HLT	N	%
Herpes viral infections	265	38.9
Lower and upper respiratory tract and lung infections	93	13.6
Epstein-Barr viral infections	38	5.6
Abdominal and gastrointestinal infections	32	4.7
Tuberculous infections	29	4.3
Candida infections	17	2.5
Influenza viral infections	14	2.1
Streptococcal infections	14	2.1
Salmonella infections	9	1.3

**Conclusion:** Our preliminary analysis showed a significant number of opportunistic infections in JIA patients on immunosuppressive therapy, which was mostly confirmed in the list of opportunistic infections approved by the experts. Further analysis on the correlation with medications is ongoing.

**Disclosure of Interest**: G. Giancane: None Declared, J. Swart: None Declared, E. Castagnola: None Declared, A. Groll: None Declared, G. Horneff: None Declared, H.-I. Huppertz: None Declared, D. Lovell: None Declared, T. Wolfs: None Declared, M. Hofer: None Declared, E. Alexeeva: None Declared, V. Panaviene: None Declared, S. Nielsen: None Declared, J. Anton: None Declared, F. Uettwiller: None Declared, V. Stanevicha: None Declared, M. Trachana: None Declared, F. De Benedetti: None Declared, C. Ailioaie: None Declared, E. Tsitsami: None Declared, S. Kamphuis: None Declared, T. Herlin: None Declared, P. Dolezalova: None Declared, G. Susic: None Declared, B. Flato: None Declared, F. Sztajnbok: None Declared, E. Fueri: None Declared, F. Bovis: None Declared, F. Bagnasco: None Declared, A. Pistorio: None Declared, A. Martini Grant/Research Support from: Starting from 1 march 2016 I became the Scientific Director of the G. Gaslini Hospital, therefore my role does not allow me to render private consultancies resulting in personal income. I perform consultancy activities on behalf of the Gaslini Institute for the following companies: Abbvie, Boehringer, Novartis, R-Pharm The money received for these activities are directly transferred to the Gaslini Institute's bank account., N. Wulffraat: None Declared, N. Ruperto Grant/Research Support from: The G. Gaslini Hospital, which is the public Hospital where I work as full time public employee, has received contributions from BMS, Hoffman-La Roche, Janssen, Novartis, Pfizer and Sobi for the coordination activity of the PRINTO network. This money has been reinvested for the research activities of the hospital in fully independent manners besides any commitment with third parties., Speaker Bureau of: Abbvie, Ablynx, AstraZeneca, Baxalta Biosimilars, Biogen Idec, Boehringer, Bristol Myers Squibb,, Eli-Lilly, EMD Serono, Gilead Sciences, Janssen, Medimmune, Novartis, Pfizer, Rpharm, Roche, Sanofi.

### O27 Use of whole-body magnetic resonance to identify potential diagnostic clues in children with fever of unknown origin (FUO)

#### Sara Signa^1^, Giorgio Stagnaro^2^, Roberta Caorsi^1^, Francesca Minoia^1^, Paolo Picco^1^, Angelo Ravelli^1^, Gian Michele Magnano^2^, Maria Beatrice Damasio^2^, Marco Gattorno^1^

##### ^1^U.O. Pediatria II, Instituto Giannina Gaslini, Genova, Italy; ^2^U.O. Radiologia, Instituto Giannina Gaslini, Genova, Italy

###### **Correspondence:** Sara Signa

**Introduction:** Whole-body magnetic resonance imaging (WBMRI) is a fast and accurate method to detect diseases throughout the entire body without exposure to ionizing radiation. Possible emerging applications for this technique include rheumatologic field and evaluation of fever of unknown origin (FUO).

**Objectives:** To evaluate the ability of WBMRI to identify significant potential diagnostic clue(PDC) in patients presenting a non specific inflammatory clinical picture.

**Methods:** We retrospectively collected cases of pediatric patients followed in a single pediatric rheumatology center who underwent WBMRI between January 2010 and December 2015 for the following indications: i) FUO(temperature greater than 38.3 °C for more than three weeks or failure to reach diagnosis after one week of investigations), iii) recurrent fever, iii) persistent low grade fever with evidence of elevation of acute phase reactants and/or other clinical symptoms.WBMRI studies were acquired with coronal and sagittal planes (slice thickness 5 mm) with acquisition of several image sets with automatic direct image realignment after acquisition creating a whole-body scan.Sequences include short τ inversion recovery (STIR) and T1-weighted. All studies have been evaluated twice, the second time according to a predefined checklist, defined by an experienced radiologist, considering systematically single/multifocal bone lesion, bone marrow, joint effusion, soft tissues, adenopathies, parenchymal and vessels looking for PDC.

**Results:** We collected 105 patients who underwent WBMRI; 24 (23%) of them presenting FUO, 29 (28%) presenting recurrent fever and 52 (49%) presenting persistent low grade fever.The mean age of onset symptoms was 7 years and seven months (range: 2 weeks old- 17 years and 6 months). The mean age of execution of WBMRI was 9 years (range: 5 months old- 19 years and one month). After the whole diagnostic work-out a final diagnosis was achieved in 34 patients (32%).

PDCs were identified at the first evaluation in 72/105 cases (68.5%) and in 29/105 cases (39%)the identified PDC was useful to the achievement of the diagnosis (7 JIA, 8 systemic infections, 4 monogenic inflammatory diseases, 3 ALPS, 2 Goldbloom’s Syndrome,2 Vasculitis,1 eosinophilic fasciitis, 1 hystiocytosis, 1 neuroblastoma).

Blind re-evaluation of WBMRI allowed the identification of additional PDCs in 51 patients (14 of them previously negative).In 8 cases the PDC found after re-evaluation were consistent with the final diagnosis (2 JIA, 2 infectious diseases,,1 neuroblastoma, 1 ALPS, 1 monogenic inflammatory disease, 1Takayasu arteritis).

**Conclusion:** WBMRI can be a powerful diagnostic tool in patients with FUO. A predefined checklist increase sensitivity of WBMRI in the identification of PDC.

**Disclosure of Interest:** None Declared.

### O28 Usefulness of magnetic resonance imaging in early assesment of low back pain with possible inflammatory cause in children

#### Lovro Lamot^1,2^, Mandica Vidovic^1^, Matej Mustapic^1^, Mirta Lamot^1^, Ivana Rados^1^, Karla Rubelj^1^, Miroslav Harjacek^1,2^

##### ^1^Clinical Hospital center Sestre Milosrdnice, Zagreb, Croatia; ^2^Univeristy of Zagreb School of Medicine, Zagreb, Croatia

###### **Correspondence:** Lovro Lamot

**Introduction:** Low back pain (LBP) is a common complaint in adults that often begins in childhood. Despite the increasing frequency, it is estimated only 24% of children with LBP visit a doctor. Nevertheless, those patients are often seen in pediatric rheumatology clinic. Although most of the LBP cases after exclusion of trauma are caused by benign musculoskeletal disease, a history of sacroiliac (SI) joint tenderness and/or inflammatory lumbosacral (LS) pain is one of the ILAR classification criteria for enthesitis related arthritis (ErA), a form of juvenile idiopathic arthritis (JIA) that includes most of the patients with juvenile spondyloarthritis (jSpA). Recognition of jSpA particularly early in the course of the disease represents a unique set of challenges and therefore all of the patients with inflammatory back pain (IBP) and arthritis or enthesitis should be suspected for jSpA with possible involvement of SI and other vertebral joints. Since magnetic resonance imaging (MRI) is the preferred method of assessment both for axial inflammation and other possible musculoskeletal, infectious and malignant causes of LBP in children, it might be advisable to use it in the initial assessment of suspected IBP in children.

**Objectives:** To evaluate the usefulness of early MRI in discovering inflammation of spinal joints and other possible causes of LBP in children with suspected IBP not fulfilling ILAR classification criteria for ErA at the time of investigation.

**Methods:** Thirty five children referred to our pediatric rheumatology clinic due to LBP lasting for more than three months, who meet ASAS criteria for IBP and had SI joint tenderness and positive FABRE test on physical examination, participated in the study. Their average age was 14.2 years (6-18) and 11 (31.4%) of them were boys. Five study participants (14.3%) had arthritis and 19 (54.3%) had enthesitis confirmed by physical examination and ultrasound, but none of them meet ILAR criteria for ErA at the time. Twelve (out of 15) were HLA-B27 positive and 13 (37.1%) had a history of SpA related disease in a first degree relative. One of the patients had a diagnosis of ulcerative colitis (UC), and one of psoriasis. All of the participants had normal CBC and CRP values, with negative ANA and RF. None had neurological symptoms. Contrast enhanced MRI of SI joints and thoracolumbar spine was performed according to recommended protocols for the assesment of inflammatory changes within one week after initial visit on a 1.5 T machine and interpreted by experienced musculoskeletal radiologist.

**Results:** Nineteen (54,3%) patients had various pathological findings detected by MRI. Four (11,4%) had signs of inflammation with one having an active sacroiliitis according to ASAS criteria. Schmorl nodes were discovered in six patients and three of them, including one with the signs of inflammation, had Scheuermann disease. Two patients had stress reaction in LS region. Five patients had incipient degeneration of intervertebral discs. Three patients had disc protrusion without and one with radial nerve compression. After three months of follow up, 19 patients (54.3%) meet ILAR criteria for ErA, one for psoriatic arthritis and one, who also had UC, meet criteria for undifferentiated arthritis.

**Conclusion:** Differential diagnosis of LBP in children is very wide and it is difficult to distinguish inflammatory and other causes upon the first encounter with pediatric rheumatologist based on history and physical examination alone. In our study, all of the patients with LBP had some characteristics of IBP and ErA, while definite diagnosis of jSpA was subsequently established in almost 60% of the patients. Interestingly, 20% of them already had inflammatory changes of LS and SI joints discovered by MRI, while many others had signs of other LBP causes. Therefore, MRI performed early after referral to pediatric rheumatologist, in all children with suspected IBP, can be very useful in elucidating the cause of LBP and differentiation of those who need only symptomatic relief, orthopedic consultation or further follow up in pediatric rheumatology clinic. High cost of this approach can be justified with the benefit of early therapeutic intervention in those with established axial inflammation and the avoidance of unnecessary treatment in those with other causes.

**Disclosure of Interest:** None Declared.

### O29 Efficacy and safety of Canakinumab in patients with HIDS/MKD: results from the pivotal phase 3 cluster trial

#### Joost Frenkel^1^, Jordi Anton^2^, Philip Hashkes^3^, Marco Cattalini^4^, Tamas Constantin^5^, Avi Livneh^6^, Bernard Lauwerys^7^, Susanne Bensler^8^, Paivi Miettunen^9^, Tillman Kallinich^10^, Jasmin Kummerle-Deschner^11^, Yankun Gong^12^, Eleni Vritzali^13^, Guido Junge^13^, Fabrizio De Benedetti^14^, Anna Simon^15^

##### ^1^University Medical Center Utrecht, Utrect, Netherlands; ^2^Hospital Sant Joan de Déu, Barcelona, Spain; ^3^Shaare Zedek Medical Center, Jerusalem, Israel; ^4^Pediatric Clinic, University of Brescia and Spedali Civili di Brescia, Brescia, Italy; ^5^Department of Pediatrics, Semmelweis Egyetem, Budapest, Hungary; ^6^Department of Medicine, Sheba Medical Centre, Tel-Hashomer, Ramat-Gan, Israel; ^7^Cliniques Universitaires Saint-Luc and Université Catholique de Louvain, Brussels, Belgium; ^8^Department of Paediatrics, Alberta Children's Hospital, University of Calgary, Alberta, Canada; ^9^Alberta Children’s Hospital Research Institute Calgary, Calgary, Canada; ^10^Charité, Humboldt University, Berlin, Germany; ^11^University Hospital Tuebingen, Tuebingen, Germany; ^12^Novartis Pharma, Shanghai, China; ^13^Novartis Pharma AG, Basel, Switzerland; ^14^IRCCS Ospedale Pediatrico Bambino Gesú, Rome, Italy; ^15^Radboud University Medical Centre, Nijmegen, Netherlands

###### **Correspondence:** Joost Frenkel

**Introduction:** Canakinumab (CAN), a fully human, specific, anti-interleukin-1β monoclonal antibody, has been shown to be efficacious in reducing the frequency of flares and improving clinical symptoms in patients (pts) with hyper immunoglobulin D syndrome/mevalonate kinase deficiency (HIDS/MKD)^1^.

**Objectives:** Primary objective was to demonstrate that CAN 150 mg every 4 weeks (q4w) was superior to placebo (PBO) in resolving flare by Day 15 with no new flares over 16 weeks (wks). Secondary objectives included: proportion of pts who maintained optimal control of disease activity (absence of new flares) between Wk 16 and Wk 40 after dose prolongation; evaluation of pharmacokinetics (PK), pharmacodynamics (PD) and safety of CAN in the HIDS/MKD pts.

**Methods:** The study comprised 4 epochs (E1-4): The study design has been reported earlier^2^. In E2, HIDS/MKD pts were randomised (1:1) to CAN 150 mg every 4 wks (q4w) or PBO. Pts who did not flare in E2 in the CAN group were rerandomised to CAN 150 mg q8w or PBO/CAN withdrawal in E3. In E3, dose could be escalated up to 300 mg q4w for pts with a flare. For PK/PD assessments, CAN concentrations and total IL1β at baseline (Day 1), and trough concentrations at Wk 16 were assessed. Safety assessments included adverse events (AEs) and serious AEs (SAEs).

**Results:** Of the 72 HIDS/MKD pts randomised to CAN 150 mg q4w (n = 37) or PBO (n = 35) in E2, 3 discontinued the study (2 PBO, 1 CAN). The proportion of responders at Wk 16 was significantly higher with CAN vs PBO (`). In E3, 13 (CAN E2 responders) were re-randomised to CAN 150 mg q8w or PBO while 53 pts completing E2 received open-label CAN treatment. In E3, the proportion of pts who did not present new flares was numerically higher in the CAN vs PBO group (Table 7). 22.7% of the HIDS/MKD pts, including pts treated in open-label maintained disease control with a prolonged dosing interval (q8w) till Wk 40. 22.7% required uptitration back to original dose 150 mg q4w while 28.8% pts required uptitration to 300 mg q4w. No new safety findings or deaths were reported in CAN treated pts through E3 (Table 7). The serum clearance and steady state volume of distribution of CAN (liquid in vial) varied according to body weight, and were estimated to be 0.14 ± 0.04 L/day and 4.96 ± 1.35 L, respectively. The estimated half-life of CAN was 25 ± 6.4 days. Total IL-1β serum concentrations showed dose proportional increase.

**Table 7:** **(Abstract O29).** Efficacy results and summary of safety

	PBO N = 35	CAN 150 mg q4w N = 37	p-value
Proportion of responders at Wk 16 (E2), n (%)	2 (5.7)	13 (35.1)	<0.002*
Proportion of patients with no new flare at Wk 40 (E3), n (%)	PBO N = 7	CAN 150 mg q8w N = 6	p-value
	1 (14.3)	3 (50.0)	0.2168
Safety			
	PBO N = 35	Any CAN*, E2 N = 68	Any CAN*, E2 + E3 N = 71
Exposure to CAN, pyr	3.2	19.1	51.0
Number of AEs (AE rate/100 pyr)	59 (1818.5)	251 (1313.6)	613 (1201.2)
Number of SAEs (SAE rate/100 pyr)	4 (123.3)	11 (57.6)	20 (39.2)

*Indicates statistical significance (one-sided) at the 0.025 level. ^#^Any patient who received a dose of CAN during E2 or E3

n = number of patients who responded; N = number of patients evaluated for response. AE, adverse event; CAN, canakinumab; E, epoch; PBO, placebo; q4w, every 4 weeks pyr, patient-years; SAE, serious AE ; Wk, week

**Conclusion:** CAN (150 mg q4w) was efficacious in resolving flare by Day 15 and preventing new flares in HIDS/MKD patients over 16 weeks. Approximately 23% pts did not flare with a prolonged dose interval (q8w) at the end of E3 compared to 14% pts who did not flare when CAN was completely withdrawn. No new safety issues were reported over 40 weeks of treatment; the safety profile was not distinct from previous controlled studies. The PK/PD results observed with the liquid-in-vial form of CAN were similar to those observed in CAPS and SJIA.

1. Arostegui JI, et al *Arthritis Rheumatol*. 2015;67(S10). 2. De Benedetti F, et al. *Ann Rheum Dis.* 2016;75(Suppl2):615.

Trial registration identifying number: NCT02059291

**Disclosure of Interest**: J. Frenkel Grant/Research Support from: Novartis and SOBI, J. Anton: None Declared, P. Hashkes Grant/Research Support from: Novartis, M. Cattalini: None Declared, T. Constantin: None Declared, A. Livneh: None Declared, B. Lauwerys: None Declared, S. Bensler Consultant for: Novartis, SOBI, AbbVie, P. Miettunen: None Declared, T. Kallinich: None Declared, J. Kummerle-Deschner Grant/Research Support from: Novartis, Consultant for: Novartis, SOBI, Baxalta, Y. Gong Employee of: Novartis, E. Vritzali Employee of: Novartis, G. Junge Employee of: Novartis, F. De Benedetti Grant/Research Support from: Pfizer, AbbVie, Roche, Novartis, Novimmune and BMS, A. Simon Grant/Research Support from: Novartis, Xoma/Servier, CSL Behring, Consultant for: Novartis, Takeda, SOBI, Xoma.

### O30 Efficacy, safety, pharmacokinetics and pharmacodynamics of Canakinumab in patients with traps: results from the pivotal phase 3 cluster trial

#### Marco Gattorno^1^, Ryoki Hara^2^, Anna Shcherbina^3^, Laura Obici^4^, Segundo Bujan^5^, Gerd Hoerneff^6^, Anette Jansson^7^, Riva Brik^8^, Itzhak Rosner^9^, Alberto Tomassini^10^, Yankun Gong^11^, Eleni Vritzali^12^, Guido Junge^12^, Fabrizio De Benedetti^13^, Helen Lachmann^14^

##### ^1^Pediatric Rheumatology, G. Gaslini Institute, Genoa, Italy; ^2^Yokohama City University, Yokohama, Japan; ^3^Clinical immunology Department, Center of Children's Hematology n.a. D. Rogachev, Moscow, Russian Federation; ^4^Amyloidosis Research and Treatment Centre, Biotechnology Research Laboratories, Fondazione IRCCS Policlinico San Matteo, Pavia, Italy; ^5^Vall d'Hebron Hospital, Barcelona, Spain; ^6^Asklepios Clinic, Sankt Augustin, Munich, Germany; ^7^Department of Rheumatology & Immunology, Dr. von Hauner Childrens Hospital, Ludwig-Maximilians-University, Munich, Germany; ^8^Meyer Children's Hospital, Rambam Medical Center, Haifa, Israel; ^9^Rheumatology, Bnai-Zion Medical Center, Rappaport Faculty of Medicine. Technion, Haifa, Israel; ^10^Institute for Maternal and Child Health- IRCCS “Burlo Garofolo”, Trieste, Italy; ^11^Novartis Pharma, Shanghai, China; ^12^Novartis Pharma AG, Basel, Switzerland; ^13^IRCCS Ospedale Pediatrico Bambino Gesú, Rome, Italy; ^14^UK National Amyloidosis Centre, University College London, London, United Kingdom

###### **Correspondence:** Marco Gattorno

**Introduction:** Interleukin (IL)–1β is known to be a key cytokine in the pathogenesis of tumour necrosis factor receptor associated periodic syndrome (TRAPS).^1^ Canakinumab (CAN) is a fully human monoclonal antibody that specifically neutralises the activity of IL–1β.

**Objectives:** The primary objective was to demonstrate that canakinumab (CAN)150 mg every 4 weeks (q4w) is superior to placebo (PBO) in achieving a clinically meaningful response [resolution of flare at Day 15 and no new disease flares over 16 weeks (wks) of treatment] in TRAPS patients (pts). Secondary objectives included: the proportion of pts who maintained optimal control of disease activity (absence of new flares) between Wk 16 and Wk 40 after dose reduction; evaluation of pharmacokinetics (PK), pharmacodynamics (PD) and safety of CAN in TRAPS pts.

**Methods:** The study comprised 4 epochs: a 12-wk screening epoch (E1), a 16-wk randomised treatment epoch (E2), a 24-wk randomised withdrawal epoch (E3) and a 72-wk open-label treatment epoch (E4). TRAPS pts with a flare during E1 were randomised in E2 to receive CAN 150 mg q4w or PBO. Pts who did not flare in E2 in the CAN group were re-randomised to CAN 150 mg q8w or PBO/CAN withdrawal in E3. In E3, for pts with a flare, dose could be escalated up to 300 mg q4w. For PK/PD assessments, CAN concentrations and total IL-1β at baseline (Day 1), and trough concentrations at Wk 16 were assessed. Safety assessments included adverse events (AEs) and serious AEs (SAEs).

**Results:** Of the 46 TRAPS pts randomised to CAN 150 mg q4w (n = 22) or PBO (n = 24) in E2, 2 pts in the PBO group discontinued the study. The proportion of responders at Wk 16 was statistically higher with CAN vs. PBO (Table 8). In E3, 9 (CAN E2 responders) were re-randomised to CAN 150 mg q8w or PBO while remaining pts completing E2 received open-label treatment. In E3, the proportion of pts with no new flare was numerically higher in the CAN vs PBO group (Table 8). 53.3% of the TRAPS pts, including pts treated in open-label maintained disease control with a prolonged dosing interval (q8w) till Wk 40. While 8.3% pts were up-titrated to 300 mg q4w. No new safety findings or death were reported in CAN treated pts through E3 (Table 8). The serum clearance and steady-state volume of distribution of CAN varied according to body weight, and were estimated to be 0.14 ± 0.04 L/day and 4.96 ± 1.35 L, respectively. The estimated half-life of CAN was 25 ± 6.4 days. Total IL-1β serum concentrations showed dose proportional increase.

**Table 8:** **(Abstract O30).** Efficacy and summary of safety

	CAN 150 mg q4w N = 22	PBO N = 24	p-value
Proportion of responders at Wk 16 (E2), n(%)	10 (45.5)	2 (8.3)	0.005*
Proportion of patients with no new flare at Wk 40 (E3), n(%)	CAN 150 mg q8w N = 4	PBO N = 5	p-value
	3 (75.0)	2 (40.0)	0.3571
Safety			
	PBO (N = 24)	Any CAN*, E2 (N = 43)	Any CAN*, E2 + E3 (N = 61)
Exposure to CAN, pyr	2.0	12.1	39.2
Number of AEs (AE rate/100 pyr)	34 (1698.8)	112 (925.7)	265 (676.2)
Number of SAEs (SAE rate/100 pyr)	1 (50.0)	3 (24.8)	5 (12.8)

*Indicates statistical significance (one-sided) at the 0.025 level. ^#^Any patient who received a dose of CAN during E2 or 3.

n = number of patients who responded; N = number of patients evaluated for response. AE, adverse event; CAN, canakinumab; E, epoch; PBO, placebo; q4w, every 4 weeks pyr, patient-years; SAE, serious adverse event; W, week

**Conclusion:** CAN (150 mg q4w) was efficacious in resolving flare by Day 15 and preventing new flares in TRAPS patients over 16 weeks. The prolonged dose interval (150 mg q8w) was sufficient to maintain disease control in approximately 50% TRAPS patients at the end of epoch 3. No new safety issues were reported over 40 weeks of treatment; the safety profile was not distinct from previous controlled studies. The PK/PD results observed with the liquid-in-vial form of CAN were similar to those observed in CAPS and SJIA.

1.Gattorno M, et al. *Ann Rheum Dis*. 2016;76(1):1738

Trial registration identifying number: NCT02059291

**Disclosure of Interest**: M. Gattorno Grant/Research Support from: Novartis and SOBI, Consultant for: Novartis and SOBI, R. Hara: None Declared, A. Shcherbina: None Declared, L. Obici: None Declared, S. Bujan: None Declared, G. Hoerneff Grant/Research Support from: AbbVie, Chugai, Novartis, Pfizer, Roche, A. Jansson: None Declared, R. Brik: None Declared, I. Rosner: None Declared, A. Tomassini: None Declared, Y. Gong Employee of: Novartis, E. Vritzali Employee of: Novartis, G. Junge Employee of: Novartis, F. De Benedetti Grant/Research Support from: Pfizer, AbbVie, Roche, Novartis, Novimmune and BMS, H. Lachmann Consultant for: Novartis, SOBI, Takeda, GSK, Speaker Bureau of: Novartis, SOBI.

### O31 Evidence based recommendations for corticosteroid tapering/discontinuation in new onset juvenile dermatomyositis patients from the PRINTO trial

#### Gabriella Giancane, Claudio Lavarello, Angela Pistorio, Francesco Zulian, Bo Magnusson, Tadej Avcin, Fabrizia Corona, Valeria Gerloni, Serena Pastore, Roberto Marini, Silvana Martino, Anne Pagnier, Michel Rodiere, Christine Soler, Valda Stanevicha, Rebecca Ten Cate, Yosef Uziel, Jelena Vojinovic, Elena Fueri, Angelo Ravelli, Alberto Martini, Nicolino Ruperto

##### Pediatria II, Reumatologia; PRINTO, Istituto Giannina Gaslini, GENOA, Italy

###### **Correspondence:** Gabriella Giancane

**Introduction:** At present no clear evidence based guidelines exist to standardize the tapering and discontinuation of corticosteroids (CS) in juvenile dermatomyositis (JDM).

**Objectives:** To provide evidence-based recommendations for CS tapering/discontinuation through the analysis of the patients in the PRINTO new onset JDM trial. Secondary objective of the study was to identify predictors of clinical remission and CS discontinuation.

**Methods:** New onset JDM children were randomized to receive either prednisone (PDN) alone or in combination with MTX or CSA. All children were given initially intravenous methylprednisolone, and then PDN starting with 2 mg/kg/day. Gradual tapering according to a specific protocol could lead to the safe dose of 0.2 mg/kg/day by month 6, then discontinued at month 24. Major therapeutic changes (MTC) were defined as the addition or major increase in the dose of MTX/CSA/other drugs or any other reasons for which patients were dropped from the trial. Patients were divided according to clinical remission (CMAS = 52 and MD global = 0 for 6 continuous months) into two major groups. Group 1 included those on clinical remission, who could discontinue PDN, with no MTC, and represented the reference standard for the best clinical outcome. Group 1 was compared with those who did not achieve clinical remission, without or with MTC (group 2 and 3, respectively). JDM core set measures (CSM) were compared in the 3 groups. We also calculated the gold standard group (group 1) median change in the CSM in the first 6 and over 24 months and applied a logistic regression model to identify the predictors of clinical remission with PDN discontinuation.

**Results:** 139 children were enrolled in the trial: 47 on PDN, 46 on PDN + CSA and 46 on PDN + MTX. We identified 30 (21.6%) patients for group 1, 43 (30.9%) for group 2 and 66 (47.5%) for group 3. At baseline all 3 groups had a high level of disease activity with no differences in the CSM. Already in the first 2 months a clear differential trend in disease activity measures, according to clinical remission status and PDN discontinuation, could be identified. From the observation of the median change in the CSM of group 1 in the first 6 months, the following recommendations could be extrapolated: decrease corticosteroids from 2 to 1 mg/kg/day in 2 months if the MD-global, parent-global, CHAQ, DAS, CMAS, MMT or Phs measures have a change of at least 50%; from 1 to 0.5 mg/kg/day in the following 2 months if the MD-global, CHAQ, DAS, CMAS have a change of at least 20%; in the following 2 months (month 4-6) corticosteroids can be tapered up to the safe dose of 0.2 mg/kg/day, if the disease activity measures remain at low/normal values. We finally ran a logistic regression model that showed that the achievement of PRINTO criteria 50-70-90 at 2 months from disease onset, an age at onset >9 years and the combination therapy PDN + MTX, increase the probability of clinical remission from 4 to 7 times. (Table 9).

**Table 9:** **(Abstract O31).** Logistic regression model for the outcome: achievement of remission (n/tot: 28/130; 21.5%)

	OR (95% CI)	P^#^
Responder at 2 months: Printo-50 (*vs.* not responder/Printo-20)	5.41 (1.37 - 21.32)	0.0076
Printo-70 (*vs.* not responder/Printo-20)	6.90 (1.91 - 24.99)	
Printo-90 (*vs.* not responder/Printo-20)	4.46 (1.08 - 18.38)	
Age at onset > 8.53 years (£ 8.53 years)	4.64 (1.69 - 12.71)	0.0017
Treatment group: PDN + MTX (*vs.* PDN/PDN + CSA)	3.63 (1.30 - 10.09)	0.0116
AUC of the model: 0.80		

OR: Odds Ratio; 95% CI: 95% Confidence Interval; P^#^: Likelihood Ratio test

**Conclusion:** We propose evidence based specific cut-offs for corticosteroid tapering/discontinuation based on the change in JDM CSM of disease activity, and to identify the best predictors for clinical remission and corticosteroid discontinuation.

Trial registration identifying number: NCT00323960

**Disclosure of Interest**: G. Giancane: None Declared, C. Lavarello: None Declared, A. Pistorio: None Declared, F. Zulian: None Declared, B. Magnusson: None Declared, T. Avcin: None Declared, F. Corona: None Declared, V. Gerloni: None Declared, S. Pastore: None Declared, R. Marini: None Declared, S. Martino: None Declared, A. Pagnier: None Declared, M. Rodiere: None Declared, C. Soler: None Declared, V. Stanevicha: None Declared, R. Ten Cate: None Declared, Y. Uziel: None Declared, J. Vojinovic: None Declared, E. Fueri: None Declared, A. Ravelli Grant/Research Support from: BMS, Hoffman-La Roche, Janssen, Novartis, Pfizer, Sobi, Speaker Bureau of: AbbVie, BMS, Pfizer, Hoffman LaRoche, Novartis, Centocor., A. Martini Grant/Research Support from: Starting from 1 march 2016 I became the Scientific Director of the G. Gaslini Hospital, therefore my role does not allow me to render private consultancies resulting in personal income. I perform consultancy activities on behalf of the Gaslini Institute for the following companies: Abbvie, Boehringer, Novartis, R-Pharm The money received for these activities are directly transferred to the Gaslini Institute's bank account., N. Ruperto Grant/Research Support from: The G. Gaslini Hospital, which is the public Hospital where I work as full time public employee, has received contributions from BMS, Hoffman-La Roche, Janssen, Novartis, Pfizer and Sobi for the coordination activity of the PRINTO network. This money has been reinvested for the research activities of the hospital in fully independent manners besides any commitment with third parties., Speaker Bureau of: Abbvie, Ablynx, AstraZeneca, Baxalta Biosimilars, Biogen Idec, Boehringer, Bristol Myers Squibb,, Eli-Lilly, EMD Serono, Gilead Sciences, Janssen, Medimmune, Novartis, Pfizer, Rpharm, Roche, Sanofi.

### O32 Evaluation of varicella zoster immune status in children with rheumatic diseases treated with biologic agents

#### Elena Moraitis^1,2^, Lucy Backhouse^1^, Ian Macdonald^1^, Jennifer Crooks^1^, Muthana Al-Obaidi^1^

##### ^1^Rheumatology, Great Ormond Street Hospital NHS Foundation Trust, London, United Kingdom; ^2^Infection, Inflammation, Rheumatology, UCL GOS Institute of child Health, London, United Kingdom

###### **Correspondence:** Elena Moraitis

**Introduction:** In immunocompromised patients, varicella zoster virus (VZV) infection can have a very severe clinical course. There is scarce evidence that in adult and paediatric population with rheumatoid or juvenile idiopathic arthritis and other rheumatic diseases the treatment with biologic agents may impair the antibody response to vaccines. However, there is no evidence on whether the use of biologic agents affects in children the VZV IgG levels acquired after natural infection.

**Objectives:** The aim of the study is to investigate whether children with a rheumatic condition treated with biologic agents have a change in their VZV immune status.

**Methods:** We performed retrospective case notes review of children presenting with any rheumatic diagnosis requiring treatment with a biologic agent to the Paediatric Rheumatology services at Great Ormond Street Hospital NHS Foundation Trust between 2009 and 2017, identified from the biologic database. We included in the study the children with positive VZV IgG prior to the treatment with a biologic agent and with a minimum of one repeat VZV IgG measurement post initiation of the treatment. A sensitive VaccZyme

VZV glycoprotein IgG Low Level Enzyme Immunoassay Kit (Binding Site) was used to measure VZV IgG.

**Results:** Sixty four patients treated with biologic agents and with two measurements of VZV IgG prior and post biologic were identified. Forty three patients had positive VZV IgG acquired prior to treatment and were included in the study. None had received the VZV vaccine, which is not part of the routine childhood vaccination schedule in the United Kingdom. After initiation of treatment with biologics, 10/43 (23%, 2 males and 8 females) had a change in VZV IgG, of which 5/43 (11.6%) had negative VZV IgG, and 5/43 (11.6%) equivocal. Four had a diagnosis of JIA, 2 systemic vasculitides, and 4 other rheumatic conditions. Seven patients were treated with anti-TNF agents, 2 patients with Tocilizumab and 1 received Abatacept. Nine patients received concomitant treatment with other disease-modifying anti-rheumatic drugs (7/9 Methotrexate, 2/9 Azathioprine). For the 10 patients, the median age at the measurement of a positive VZV IgG pre-biologic was 7 years (range 1-12), median time between the two assessments was 40.5 months (range 9-105), and the median time between the initiation of the biologic agent and the post-biologic measurement of VZV IgG was 24 months (range 11-68).

**Conclusion:** We describe the changes in the VZV immune status post treatment with biologics in the first cohort of paediatric patients with initial seroconversion following natural infection. Although limited by the size of the study population, observational nature and other concomitant medications, our results indicate that biologic agents can affect the VZV immune status of patients with rheumatic conditions, making them susceptible to re-infection. Therefore we suggest that all patients on biologic agents should have the VZV IgG levels re-tested regularly to identify whether the VZV IgG levels are protective and give prophylaxis as required in the event of varicella contact.

**Disclosure of Interest:** None Declared.

### O33 Prolonged reduced aerobic fitness in adolescents and young adults with juvenile idiopathic arthritis

#### Philomine Van Pelt^1,2^, Tim Takken^3^, Marco V. Brussel^3^, Radboud Dolhain^4^, Johannes Bijlsma^5^, Aike Kruize^5^, Nico Wulffraat^1^

##### ^1^rheumatology and pediatric rheumatology, Wilhelmina Children’s Hospital, University Medical Center Utrecht, Utrecht, Netherlands; ^2^rheumatology and pediatric rheumatology, ErasmusMC University Rotterdam, Rotterdam, Netherlands; ^3^Child Development and Exercise Center, Wilhelmina Children’s Hospital, University Medical Center Utrecht, Utrecht, Netherlands; ^4^rheumatology, ErasmusMC University Rotterdam, Rotterdam, Netherlands; ^5^rheumatology and immunology, University Medical Center Utrecht, Utrecht, Netherlands

###### **Correspondence:** Philomine Van Pelt

**Introduction:** Aerobic fitness may serve as an important health-related outcome measure in Juvenile Idiopathic Arthritis (JIA). Reduced aerobic fitness is associated with cardiovascular morbidity, mortality and osteoporosis in adult patients with chronic diseases. However, in adolescents and young adults, long-term outcome of aerobic fitness is unknown. Reduced aerobic fitness was described previously in cross-sectional studies in children and adolescents with JIA and was more impaired in active disease.

**Objectives:** Our objectives are to describe the course of aerobic fitness in a longitudinal cohort of adolescents and young adult JIA-patients who are intensively treated and to identify the association of clinical variables.

**Methods:** In a longitudinal cohort, all consecutive patients aged 10-24 years were included after informed consent. Annual examinations were obtained from demographic and disease-related items. At baseline and at study-end, aerobic fitness (VO_2_peak) was assessed using a graded cardiopulmonary exercise test (CPET) to volitional exhaustion performed on a cycle ergometer. Absolute and relative VO_2_peak values were measured and related to reference values of healthy controls (Z-scores), using one-sample T-tests. Non-parametric tests were used to evaluate results.

**Results:** Paired Z-scores were available from 35 patients. 40% were male, median age at baseline was 13,0 yrs (IQR 4,1), disease duration 7,8 yrs (6,4), JADAS27 3,7 (6,4), DAS28 1,8 (1,0). 79% of the patients were in DAS28-remission. 17% had systemic JIA, 6% persistent oligoarticular and 74% had a polyarticular course. Baseline and end Z-scores were reduced compared to healthy controls (ZAbs_base -0,68, IQR2,3 p = 0,01; Zrel_base -1,33, IQR 2,0, p < 0,01; Zabs_end -1,33, IQR 1,7, p < 0,01; Zrel_end -0,70, IQR 1,8, p = 0,01) and did not change significantly over time (change Zabs_change 0,65, p = 0,34; Zrel_change 0,63, p = 0,31). At baseline, a higher DAS28 (p = 0,01), higher JADAS27 (p < 0.01), ESR (p < 0,01), higher thrombocytes (p < 0.01) and the use of MTX (p = 0.01) were associated with a worse outcome of aerobic fitness. The greatest improvement of aerobic fitness over time was seen in male patients (p < 0.01) at baseline. Multivariate analysis showed that a higher DAS28 and male gender were the most important variables for worse aerobic fitness at baseline, a higher ESR at baseline was the most important predictor for improving aerobic fitness over time.

**Conclusion:** Aerobic fitness is significantly reduced in adolescents and young adults with JIA and does not improve over time, despite intensive treatment. Be aware of a reduced health outcome into adulthood due to a persistent reduced aerobic fitness during disease course of JIA, despite low disease activity.

**Disclosure of Interest:** None Declared.

## Autoinflammatory diseases

### P1 PFAPA syndrome in large pediatric population: a single center experience

#### Esra Pehllivan, Amra Adrovic, Sezgin Sahin, Kenan Barut, Ovgu Kul, Ozgur Kasapcopur

##### Pediatric Rheumatology, Istanbul University, Cerrahpasa Medical School, Istanbul, Turkey

###### **Correspondence:** Amra Adrovic

**Introduction:** Periodic fever, aphthosis, pharyngitis, and adenitis (PFAPA) syndrome is an auto-inflammatory condition of unknown etiology. It is the second most common auto-inflammatory disease in our country, following familial Mediterranean fever. The strong familial clustering suggest a potential genetic origin of the syndrome but none of single genetic variants seem to be relevant for the disease etiology. Previous studies showed that tonsillectomy represents efficient treatment options.

**Objectives:** Our aim was to explore the main clinical features, response to tonsillectomy and long–term outcome of PFAPA pediatric patients in a single cohort. We assessed association of MEFV gene mutation with disease characteristics and treatment response.

**Methods:** We reviewed medical records of patients who were diagnosed with PFAPA syndrome between the January 2010 and June 2016. All of the recorded 562 patients were called by the telephone and 359 (64%) of them were reached. Demographic, clinical and therapeutically features were taken from the patients’ medical records. Data on clinical course and the disease outcome were collected by using a structured questionnaire, which was fulfilled during the phone conversation between investigator and patient’s parents.

**Results:** A total of 359 patients with PFAPA were examined: 155 (43%) of them were female. The mean age at disease onset, at diagnosis and at the investigation was 22.79 ± 18.8, 41.7 ± 21.7 and 77.14 ± 31.35 months, respectively. The most common disease feature at the disease onset was: recurrent fever in 359 (100%), cryptic tonsillitis in 359 (100%) and aphtous stomatitis in 317 (88%). Sixty-three (17%) patients met the criteria for both PFAPA and FMF. MEFV gene mutation analysis was performed in 93 (25%) patients and 51 of them (54%) had a heterozygous mutation in exon 10. Surgical treatment was performed in 158 (43%) patients. Complete clinical remission was achieved in 127 (80.3%) patients. Six (3%) showed no response to surgical treatment while 25 (15.8%) patients had a partial response. In patients with partial clinical response, frequency of fever attacks decreased significantly from 17.5 to 7.3 attack per year (p < 0.05). Among patients who did not respond to tonsillectomy, 11 (52.4%) were carrier of *MEFV* heterozygous mutation in exon 10. There was a statistically significant difference between patients with and without coexistence of FMF features, according to surgical treatment response (p < 0.05). The mean age of resolution of PFAPA symptoms in patients who underwent tonsillectomy was 52 ± 22.4 months and in patients without tonsillectomy 66 ± 22.6 months (Table 10).

**Table 10:** **(Abstract P1).** Clinical characteristics of patients according to coexistence of FMF

	Patients with coexistence of FMF (n = 63)	Patients without coexistence of FMF(n = 296)	p
Mean age at disease onset (months ± SD)	27 ± 23	22 ± 16.6	0.562
Mean age at diagnosis (months ± SD)	44 ± 23	43 ± 21	0.274
Male/Female ratio	36/27	168/128	1.0
Mean time interval between fever episodes (weeks ± SD)	3 ± 0.9	3.2 ± 1.4	0.61
Mean duration of fever (days ± SD)	3.6 ± 1.4	3.9 ± 3.6	0.94
Cryptic tonsillitis	63 (100%)	296 (100%)	1.0
Abdominal pain	20 (31.7%)	83 (28%)	0.54
Response to colchicum treatment	18/27 (66%)	6/18 (33%)	0.03
Clinical response to surgical treatment	18/36 (50%)	113/146 (77.4%)	0.002

**Conclusion:** Although PFAPA symptoms usually resolve before age of eight, some patients’ complaints persist. FMF should be considered in tonsilloadenoidectomy unresponsive PFAPA patients, especially in endemic regions like Turkey. Tonsilloadenoidectomy seems to be an effective treatment option for pediatric PFAPA patients.

References:

1. Marshall GS, Edwards KM, Butler J, Lawton AR (1987) Syndrome of periodic fever, pharyngitis, and aphthous stomatitis. J Pediatr 110:43–46

2. Vanoni F, Theodoropoulou K, Hofer M. PFAPA syndrome: a review on treatment and outcome. Pediatr Rheumatol Online J 2016;14:38.

3. Batu ED, Kara Eroğlu F, Tsoukas P, et al. Periodic Fever, Aphthosis, Pharyngitis, and Adenitis Syndrome: Analysis of Patients From Two Geographic Areas. Arthritis Care Res (Hoboken) 2016;68:1859-1865.

4. Celiksoy MH, Ogur G, Yaman E, et al. Could familial Mediterranean fever gene mutations be related to PFAPA syndrome? Pediatr Allergy Immunol. 2016;27:78-82.

**Disclosure of Interest:** None Declared.

### P2 through new classification criteria for inherited periodic fevers and PFAPA. An integrated approach among clinicians and geneticists.

#### Silvia Federici^1^, Federica Vanoni^2^, Francesca Bovis^3^, Maria Pia Sormani^3^, Nicola Ruperto^4^, Marco Gattorno^4^ and on behalf of the Expert Committee for the Classification Criteria in periodic fever

##### ^1^2° Division of Pediatric, Gaslini Institute, Genova, Italy; ^2^Pediatric Rheumatology Unit, CHUV, University of Lausanne, Lausanne, Switzerland; ^3^Biostatistics Unit, Department of Health Sciences, University of Genoa, Genoa, Italy; ^4^2° Division of Pediatric, Gaslini Institute, Genoa, Italy

###### **Correspondence:** Silvia Federici

**Introduction:** Provisional Eurofever evidence-based classification criteria for inherited periodic fever (TRAPS, FMF, MKD and CAPS) have been recently developed and other diagnostic criteria are available for FMF, CAPS and PFAPA. However, no consensus on how to combine clinical criteria and results of molecular analysis has been reached so far. We have recently identified, through a process based on the Delphi techniques (2 subsequent Delphi surveys), those variables the expert, clinicians and geneticists, consider as important for the diagnosis of each monogenic periodic fever syndrome and PFAPA. We also obtained, trough a web-based evaluation a consensus >80% among clinicians and geneticists on the diagnosis for 269 over 360 patients with monogenic periodic fever, PFAPA and undefined periodic fever (UPF) randomly selected from the Eurofever Registry.

**Objectives:** To identify candidate set of classification criteria for monogenic periodic fever and PFAPA and to test their sensibility and specificity on the subset of patients having reached a consensus among experts. To select the best sets of criteria for each disease to be discussed in a Consensus Conference

**Methods:** We selected for each disease the clinical variables corresponding to the 3rd quartile considering the total score obtained after the second Delphi survey. Performing univariate statistical analysis, we subsequently assess the ability of these variables to classify individual patients as having or not FMF, MKD, TRAPS, CAPS, PFAPA or UPF according to consensus classification of experts. These variables could be associated both in a positive or negative way to each disease. At the same time we assigned to each genotype presented by our patients a score from 5 to 1 on the basis of the evaluation done by the experts to be able to include the results of genetic testing in the statistical analysis. In a second step, multivariate statistical analysis has been performed to obtain different sets of criteria including only positively associated or positively and negatively associated clinical variables with and without genetic data. For each criteria the sensitivity, specificity, positive predictive value (PPV), negative predictive value (NPV) and area under the curve (AUC) were calculated. The best performing criteria for each disease have been selected and presented to the expert for voting in a consensus Conference held in Genova in march 2017.

**Results:** A total of 98 clinical variables derived from the second Delphi survey has been tested. The univariate analysis identified 15 variables significantly associated with FMF (6 positively and 9 negatively), 20 with CAPS (12 positively and 8 negatively), 9 with TRAPS (6 positively and 3 negatively), 13 with MKD (11 positively and 2 negatively) and 15 with PFAPA (5 positively and 10 negatively). 193 criteria derived from the statistical analysis have been selected (45 for FMF, 50 for CAPS, 44 for TRAPS, 32 for MKD and 22 for PFAPA) to be discussed and voted at the consensus. In addition criteria already published in the literature and criteria directly proposed by experts were included for discussion.

**Conclusion:** Our process led to the identification of the best variables to be included in the multivariate analysis for the identification of the candidate criteria for monogenic periodic fever and PFAPA. Next, the ability of candidate criteria to classify individual patients as having FMF, MKD, TRAPS CAPS or PFAPA have been assessed by evaluating the agreement between the classification yielded using the criteria and the consensus classification of the experts. The best classification criteria for each disease in term of sensibility and specificity have been shown and voted by a panel of Experts during the Consensus Conference held in Genoa. For CAPS, FMF and MKD a consensus >80% has been reached for the best genetic/clinical criteria and for the best clinical criteria while for TRAPS the consensus has been reached only for the best clinical/genetic criteria. A new round of web-based evaluation is now ongoing to try to reach a consensus on the clinical criteria for TRAPS as well. Moreover a consensus has been reached for PFAPA clinical criteria.

**Disclosure of Interest:** None Declared.

### P3 Chronic recurrent multifocal osteomyelitis: case report

#### Maria Francesca Gicchino^1^, Mario Diplomatico^1^, Daniela Capalbo^1^, Carmela Granato^1^, Alma Nunzia Olivieri^1^

##### ^1^Department of Women, Child and General and Specialistic Surgery, Università della Campania Luigi Vanvitelli, Naples, Italy

###### **Correspondence:** Maria Francesca Gicchino

**Introduction:** Chronic recurrent multifocal osteomyelitis (CRMO), also known as chronic nonbacterial osteomyelitis, is a rare, noninfectious inflammatory disorder that causes multifocal lytic bone lesions with swelling and pain characterized by periodic exacerbations and remissions of unclear/unknown pathogenesis.

**Objectives:** To describe a case of CRMO in a 10 years old girl.

**Methods:** A 10 years old girl came to our department because of pain in her right shoulder since the age of 9 years. She did not recall a precipitating event or a trauma, reported no fever or weakness. There was no relevant personal or family history. Before coming to our observation an X-ray of her right shoulder revealing an osteolytic lesion and a PET-CT showing the presence of a pathological high-uptake of the lesion were performed, and a biopsy of the lesion of the right shoulder was done to exclude a neoplastic lesion, a bone infection, or a Langerhans cells histiocytosis (LCH). Patient’s bone biopsy showed infiltration by lymphocytes and neutrophils. No neoplastic cells were identified. All cultures were negative. LCH was excluded since immunohistochemical evaluation of bone marrow for CD1a and S100 expression was negative. When she was admitted to our department she had pain in her right arm and shoulder. Laboratory results showed a modest increase of inflammation parameters (ESR 34 mm/h, CRP 0,88 mg/dL) with normal complete blood count, liver and renal function. The abdominal ultrasound and the chest X-ray were normal so we performed an MRI. On MRI the lesion was hypointense in T1 and hyperintense in T2 and STIR, suggesting an inflammatory process.Suspecting a CRMO, anti-inflammatory treatment with naproxen was prescribed and the symptomatology disappeared. Five months later the patient came back to our department because of pain and swelling in her right clavicle. The physical examination showed a painful swelling of the sternal end of the right clavicle. Laboratory results indicated a modest increase of inflammation parameters (ESR 29 mm/h, CRP 0,98 mg/dL). Clavicle X-ray showed an osteolytic lesion, ultrasound of sternoclavicular joint revealed articular effusion. On MRI this lesion was hyperintense in T2 and STIR.

**Results:** Clinical history, physical signs, instrumental investigations and histopathological pattern were highly suggestive of CRMO. Anti-inflammatory treatment with naproxen was started again and the symptoms disappeared. After six months was performed a whole-body MRI to evaluate the status of disease. The MRI did not show new lesions and previous bone lesions disappeared.

**Conclusion:** In a patient with recurrent bone pain CRMO should be considered. The diagnosis is of exclusion and it is based on the clinical and radiological data. Biopsy is needed to exclude infectious osteomyelitis, malignancy, LCH. Some authors suggest that biopsy is not necessary if a patient has classical radiological findings of CRMO, or comorbidities, such as Crohn's disease. Skeletal manifestations are unifocal or multifocal, and the involvement of clavicle, sternum or mandible suggest a CRMO diagnosis. Extra-articular manifestation are gastrointestinal and skin involvement (acne, pustolsis, psoriasis).The SAPHO syndrome seems to be an advanced state of CRMO. The treatment of CRMO has been mostly empiric; NSAIDs are the first choice for CRMO treatment. When disease activity is high or there are complications therapy with bisphosphonates or biologic drugs such as TNF antagonists (etanercept) or inhibitors of IL-1 (anakinra) should be considered.

**Disclosure of Interest:** None Declared.

### P4 Pluripotent stem cell models of Blau syndrome reveal an IFN-γ-dependent inflammatory response in macrophages

#### Yuri Kawasaki^1^, Sanami Takada^2^, Naotomo Kambe^3^, Ryuta Nishikomori^4^, Tatsutoshi Nakahata^1^, Megumu K. Saito^1^

##### ^1^Department of Clinical Application, Center for iPS Cell Research and Application, Kyoto University, Kyoto, Japan; ^2^Department of Dermatology, Chiba University Graduate School of Medicine, Chiba, Japan; ^3^Department of Dermatology, Kansai Medical University, Hirakata, Japan ^4^Department of Pediatrics, Kyoto University Graduate School of Medicine, Kyoto, Japan

###### **Correspondence:** Yuri Kawasaki

**Introduction:** Blau syndrome, or early-onset sarcoidosis, is a juvenile-onset systemic granulomatosis associated with a mutation in *nucleotide-binding oligomerization domain 2* (*NOD2*). The underlying mechanisms of Blau syndrome leading to autoinflammation are still unclear, and there is currently no effective specific treatment for Blau syndrome.

**Objectives:** To elucidate the mechanisms of autoinflammation in Blau syndrome, we sought to clarify the relation between disease associated-mutant NOD2 and the inflammatory response in human samples.

**Methods:** Blau syndrome-specific induced pluripotent stem cells (iPSCs) lines were established. To precisely evaluate the *in vitro* phenotype of iPSC-derived cells, the disease-associated *NOD2* mutation of iPSCs was corrected using a CRISPR-Cas9 system. We also introduced the same *NOD2* mutation into a control iPSC line. These isogenic iPSCs were then differentiated into monocytic cell lineages, and the status of NF-κB pathway and proinflammatory cytokine secretion were investigated.

Results: IFN-γ acted as a priming signal through the up-regulation of NOD2. In iPSC-derived macrophages with mutant NOD2, IFN-γ treatment induced ligand-independent, NF-κB activation and proinflammatory cytokine production. RNA-seq analysis revealed distinct transcriptional profiles of mutant macrophages both before and after IFN-γ treatment. Patient-derived macrophages demonstrated a similar IFN-γ-dependent inflammatory response.

**Conclusion:** Our data support the significance of ligand-independent autoinflammation in the pathophysiology of Blau syndrome. Our comprehensive isogenic disease-specific iPSC panel provides a useful platform for probing therapeutic and diagnostic clues for the treatment of Blau syndrome patients.

**Disclosure of Interest:** None Declared.

### P5 Transcriptional regulatory gene variations could influence expression patterns and inflammatory propagation in clavicular cortical hyperostosis

#### Lovro Lamot^1,2^, Kristina Gotovac Jercic^2^, Antonela Blazekovic^2^, Mandica Vidovic^1^, Mirta Lamot^1^, Fran Borovecki^2^, MIroslav Harjacek^1,2^

##### ^1^Clinical Hospital Center Sestre Milosrdnice, Zagreb, Croatia; ^2^University of Zagreb School of Medicine, Zagreb, Croatia

###### **Correspondence:** Lovro Lamot

**Introduction:** Clavicular cortical hyperostosis (CCH) is a rare inflammatory bone disorder of unknown aetiology represented by pain and swelling of the clavicle. Although similar to chronic nonbacetril/recurrent multifocal osteomyelitis (CNO/CRMO), due to lack of recurrence and additional inflammatory sites, it can be considered separate disease in the spectrum. In our previous study microarray gene expression profiling of CCH patients revealed 974 differentially expressed genes involved in various inflammatory processes (1). Subsequent qRT-PCR in additional number of patients confirmed significantly lower expression of ERBB2 gene and higher expression of TRPM3 and TPRM7 transient receptor potential (TRP) channel genes. Additionally, immunofluorescence microscopy showed high signal of TRPM3 in blood cells of one CCH patient. Based on described findings and other studies which showed TRP channels are involved in inflammasome activation, while ERBB activation promotes protective cellular outcomes during inflammation, we hypothesized CCH could be autoinflammatory disease, but the mechanisms responsible for observed expression alterations remained unclear.

**Objectives:** Identify possible disease causing gene variants in CCH patients using whole exome sequencing (WES).

**Methods:** Genomic DNA was extracted from peripheral whole blood samples of three CCH patients. Targeted exome sequencing was performed using the Nextera Rapid Capture Exome Kit (Illumina). The sequence library was constructed with 50 ng of genomic DNA and 100-bp paired-end reads were sequenced on HiSeq 2000 next-generation sequencer (Illumina). The extracted variants were annotated and filtered using the Variant Studio software (Illumina) and Variant Interpreter (Illumina).

**Results:** WES analysis identified 428 shared identical variants among affected individuals. Thirty of these variants were not associated with a gene, 121 were in ZNF717 gene and 277 were distributed in 63 other genes. One heterozygous variant in CTBP2 gene, one in HYDIN gene and six in ZNF717 were classified as likely pathogenic (Table 11).

**Table 11:** **(Abstract P5).** Shared identical variants classified as likely pathogenic

Gene	Variant	Coordinate	Transcript	Consequence	dbSNP ID
CTBP2	T > T/A	126727602			rs76555439
HYDIN	GA > GA/G	70896015	NM_032821.2	frameshift_variant, feature_truncation	rs77276171
ZNF717	A > A/T	75786827	NM_001128223.1	stop_gained	rs79635065
ZNF717	C > C/A	75786916	NM_001128223.1	stop_gained	rs78906544
ZNF717	G > G/T	75787127	NM_001128223.1	stop_gained	rs74740396, rs148624740
ZNF717	T > T/TA	75787352	NM_001128223.1	frameshift_variant, feature_elongation	
ZNF717	GAA > GAA/G	75787645	NM_001128223.1	frameshift_variant, feature_truncation	rs67117547, rs141065192
ZNF717	G > G/C	75788028	NM_001128223.1	stop_gained	rs77971486, rs150689808

**Conclusion:** In this comprehensive study “bottom-up” approach was used in order to elucidate the molecular disease mechanisms of what we believe could be a new disease entity. Previously performed diligent transcriptome analysis and proof-of-concept experiment which confirmed the results on a protein level indicated CCH could be induced by sternoclavicular joint overuse, TRP channel overexpression, inflammasome activation and reduced protection during inflammation. Interestingly, WES analysis identified majority of likely pathogenic variants in ZNF717 gene. This gene encodes a Kruppel-associated box (KRAB) zinc-finger protein, which belongs to a large group of transcriptional regulators and is therefore involved in the regulation of crucial physiological and pathological processes (2). Many aspects of these proteins are still essentially unknown, as is their role in the inflammation, yet it is speculated they increase DNA accessibility and inflammatory gene transcription (3). Together with possible influence on observed expression patterns, all of these processes could contribute to CCH evolution.

References

1. Lamot L et al. TRP channels overexpression contributes to inflammasome activation in clavicular cortical hyperostosis? Ann Rheum Dis 2015;74(Suppl2):146.

2. Lupo A et al. KRAB-Zinc Finger Proteins: A Repressor Family Displaying Multiple Biological Functions. Current Genomics 2013;14(4):268–278.

3. Chinenov Y et al. Glucocorticoid Receptor Coordinates Transcription Factor-Dominated Regulatory Network in Macrophages. BMC Genomics 2014;15(1):656.

**Disclosure of Interest:** None Declared.

### P6 A novel phenotype associated with ADAM17 mutations

#### Sonia Melo Gomes^1,2^, Ebun Omoyinmi^1^, Ying Hong^1^, Glenn Anderson^3^, William Mufzud^3^, William Moore^4^, Clive Edelsten^4^, Louise Wilson^5^, Paul Brogan^2^

##### ^1^Rheumatology, GOSH UCL Institute of Child Health, London, United Kingdom; ^2^Rheumatology, Great Ormond Street Hospital, London, United Kingdom; ^3^Pathology, Great Ormond Street Hospital, London, United Kingdom; ^4^Ophthalmology, Great Ormond Street Hospital, London, United Kingdom; ^5^Genetics, Great Ormond Street Hospital, London, United Kingdom

###### **Correspondence:** Sonia Melo Gomes

**Introduction:** ADAM17 is an important shedding enzyme that regulates the response to tissue injury and inflammation. Only 2 cases of ADAM17 mutations in humans have been described thus far: siblings with a homozygous ADAM17 deletion resulting in a severely truncated protein causing autosomal recessive neonatal inflammatory bowel and skin disease.

**Objectives:** The aim of this study was to discover the genetic cause affecting a child with a novel inflammatory phenotype.

**Methods:** Whole Exome sequencing was performed using DNA extracted from peripheral blood for a trio analysis. The Nextera Exome Capture and Hiseq sequencing platforms were used in this process.

**Results:** A four-year- old girl presented with extreme photophobia, bloody diarrhoea, anal fissures from the first weeks of life, and intermittent fevers. Examination revealed blepharitis, perioral rash and gingival inflammation with hyperthrophy. Feeding difficulties attributed to gastro-oesophageal reflux led to oral aversion and poor weight gain. Inflammatory markers were moderately elevated (mean CRP-22 mg/L and ESR-32 mm/h) in between fevers, and higher (CRP > 150 mg/L) during fevers. Upper gastrointestinal endoscopy at 10 months of age showed gastritis and duodenitis. Brain MRI showed cerebellar hypoplasia; corneal biopsy revealed an abnormal cornea and conjunctival epithelium. Extensive neuro-metabolic investigations performed were normal. Whole exome sequencing revealed 2 rare heterozygous missense mutations in the ADAM17 gene (p.I50V and p.R215V). Sanger sequencing confirmed these results, and carrier status in the parents. Both these mutations were absent from our in-house exome database of 120 exomes; were not seen in homozygous state in population databases; and were predicted damaging in silico. Biopsies of oral mucosa, tonsillar and skin tissue were then assessed by electron microscopy showing patchy structural changes in hemidesmosomes.

**Conclusion:** We describe the third case of human disease associated to ADAM17, but with a novel phenotype, similar to conditional knock-out mouse models with severely reduced ADAM17 sheddase activity, which develop opaque eyes, skin and heart defects as well as increased susceptibility to dextran sulfate induced colitis. This is in-keeping with the mutations found in our proband, since they are unlikely to result in the complete absence of the protein described in the other human cases.

Ongoing functional work will provide further information on how these mutations affect ADAM17 activity and impact on hemidesmosome structure.

**Disclosure of Interest:** None Declared.

### P7 The impact of gastrointestinal clinical manifestations in autoinflammatory diseases (AID): lessons from the international Eurofever registry

#### Alessia Omenetti^1^, Francesca Bovis^2^, Joost Frenkel^3^, Helen J. Lachmann^4^, Seza Ozen^5^, Nicolino Ruperto^6^, Marco Gattorno^6^ on behalf of PRINTO and Eurofever Registry

##### ^1^DINOGMI, Genoa, Italy; ^2^Biostatistics Unit, Department of Health Sciences,, University of Genoa, Genoa, Italy; ^3^University Medical Center Utrecht, Utrecht, Netherlands; ^4^Division of Medicine, University College London, London, United Kingdom; ^5^Department of Paediatric Rheumatology, Hacettepe University, Ankara, Turkey; ^6^Pediatria II, Gaslini IRCCS, Genoa, Italy

###### **Correspondence:** Alessia Omenetti

**Introduction:** The spectrum of autoinflammatory diseases (AID) is continuosly expanding and significant delay in diagnosis may occur if those rare entities are not promptly identified.

**Objectives:** To assess the impact and significance of gastrointestinal (GI) clinical manifestations in a broad cohort of AID patients.

**Methods:** The web-based international Eurofever registry containing data retrospectively collected about patients enrolled by 108 partecipating centres from November 2009 to June 2016 was evaluated. When present, each GI manifestation was ranked as occasionally or persistently present during flares. Development of severe GI complications was also assessed. To then identify subsets of patients featured by homogeneous patterns of clinical features, the unsupervised Latent Class (LC) analysis was applied to the cohort featured by GI manifestations (GI cohort) in the attempt to assign mutually exclusive class membership based on latent patterns among the observed variables.

**Results:** The cohort included 1655 patients with confirmed diagnosis of AID. At least 1 GI manifestation was reported in 52.32%. Interestingly, the latter had earlier onset compared to cohort that did not show GI involvement (5.18 ± 8.18 vs 9.11 ± 10.25, mean years ± SD). As expected, the majority of FMF, MVK and TRAPS were affected by GI symptoms whereas CAPS and CRMO were mainly represented in the subset lacking GI involvement. In the GI cohort (Table 12), during flares LC1 (13%) displayed persistent abdominal pain together with prolonged diarrhea, and it mainly included majority of MVK. LC2 (7%) was affected by occasional abdominal pain together with occasional vomiting, diarrhea and hepatosplenomegaly. LC3 represented the majority of GI cohort (72%) and presented only occasional abdominal pain while LC4 (8%) was characterized by persistent abdominal pain occasionally associated with vomiting, diarrhea, and sometime developing complications such as septic peritonitis and peritoneal adhesions. As expected, the latter was mostly represented by FMF.

**Table 12:** **(abstract P7).** See text for description

n=	LC1	LC2	LC3	LC4	Tot GI Cohort	Total not GI Cohort	Total AID
Total	87	52	666	61	866	789	1655
CAPS	4	7	31	2	44	134	179
CRMO	0	0	28	1	29	323	352
FMF	17	15	274	43	349	28	378
MVK	39	15	56	8	118	4	122
PFAPA	11	2	99	0	112	158	271
TRAPS	7	7	101	5	120	37	158
Undefined	9	5	57	1	72	63	135
Others	0	1	20	2	23	38	60

**Conclusion:** These data unveiled the unforeseen significance of GI involvement in AID with implications in the daily clinical management. GI specialists should be aware of these rare diseases in order to avoid delayed diagnosis and joined work-up protocols should be developed in order to monitor these signs and prevent potential complications. A prospective analysis is needed in order to delineate the disease history and potential evolutions (if any) into more defined GI diseases in the contest of autoinflammation.

**Disclosure of Interest:** None Declared.

### P8 Next generation sequencing-based panel screening in patients with undifferentiated autoinflammatory diseases: friend or foe?

Abstract withdrawn

### P9 Monogenic diseases masquerading as Behçet’s disease in the young

#### Charalampia Papadopoulou^1^, Ebun Omoyinmi^1^, Ariane Standing^1^, Jessica Macwilliam^2^, Clare Pain^2^, Despina Eleftheriou^1,3^, Paul Brogan^1^

##### ^1^Infection, Inflammation and Rheumatology Section, UCL Great Ormond Street Institute of Child Health, London, United Kingdom; ^2^Department of Paediatric Rheumatology, Alder Hey Children's NHS Foundation Trust, Liverpool, United Kingdom; ^3^ Centre for Adolescent Rheumatology, Arthritis Research UK, London, United Kingdom

###### **Correspondence:** Charalampia Papadopoulou

**Introduction:** Monogenic autoinflammatory disorders (AID) and some primary immunodeficiencies can present early in life with Behçet-like symptoms, and may be mistaken for Behçet’s disease (BD). Most of these monogenic conditions require alternative treatments than typically required for BD, and are associated with high morbidity and mortality if diagnosis and appropriate treatment are delayed. Next generation sequencing (NGS) undoubtedly offers the opportunity to screen for these diseases more efficiently, and greatly facilitates diagnosis in this context.

**Objectives:** To retrospectively describe the clinical and laboratory features, and molecular diagnoses of 11 paediatric cases referred for suspected BD.

**Methods:** Retrospective, two-centre, case series. A combination of NGS or conventional candidate gene screening approaches were utilised to ascertain genetic diagnoses, facilitated in some cases by functional immunological screening assays. NGS approaches utilised included targeted exome sequencing (the “Vasculitis and Inflammation Panel”, VIP); or whole exome sequencing (WES).

**Results:** Eleven children referred with suspected BD underwent further molecular testing because of incomplete or atypical BD features, and presentation under the age of 5 years. Seven/11 patients (58%) were Caucasian; the remaining 4/11 were Asian; 64% were female. Two cases were siblings from a consanguineous kindred with “familial” BD; and another had a maternal history of BD. The median age of disease onset was 1.0 (range 0.5-3.0) years. All had systemic inflammation and oral ulceration; 4/11 had genital ulceration; 4/11 had ocular involvement; and 8/11 had cutaneous manifestations. Nine out of 11 were considered to have a monogenic cause: the final genetic diagnoses were: A20 haploinsufficiency (n = 1); Hyper IgE syndrome (n = 1); LYN associated AID (n = 1); TRAPS (n = 1), Chronic Granulomatous Disease (n = 2), Periodic Fever Immunodeficiency and thrombocytopenia Syndrome (PFIT; n = 2), and pustular psoriasis caused by mutation in AP1S3 (n = 1). The remaining two cases had a suspected atypical AID caused by a novel variant in MEFV (n = 1); and a STAT1 variant, as the suspected but as yet unproven monogenic cause (n = 1).

**Conclusion:** Rare monogenic disorders can mimic BD, particularly when the presentation is under the age of 5 years. These data are now informing a strategy to begin to explore screening for genetic mimics of BD in a UK cohort of children and adults to better understand the proportion of UK BD patients who may in fact have an underlying genetic diagnosis.

**Disclosure of Interest:** None Declared.

### P10 Proteomic profile of patients with different autoinflammatory diseases: an approach to identify new biomarkers

#### Federica Penco^1^, Andrea Petretto^2^, Chiara Lavarello^2^, Elvira Inglese^2^, Ilaria Gueli^3^, Alessia Omenetti^3^, Martina Finetti^3^, Federico Tomasi^4^, Annalisa Barla^4^, Arinna Bertoni^1^, Claudia Pastorino^1^, Marco Gattorno^3^

##### ^1^Laboratorio di Immunologia delle Malattie Reumatiche Pediatria II, Università degli Studi di Genova, Genova, Italy; ^2^Core Facilities, Università degli Studi di Genova, Genova, Italy; ^3^U.O. Pediatria II, Instituto Giannina Gaslini, Università degli Studi di Genova, Genova, Italy; ^4^Dibris, Università degli Studi di Genova, Genova, Italy

###### **Correspondence:** Federica Penco

**Introduction:** Autoinflammatory diseases are a group of inherited diseases characterized by early onset and systemic inflammation, often manifesting with unexplained fevers. These pathologies are usually caused by mutations in genes involved in the regulation of innate immune response with consequent inflammatory phenotype. The mechanism that lead to the development of this diseases are not still clear and part are however genetically undefined.

**Objectives:** Our aim is to evaluate the differences in the expression of proteins or pathway in monocytes, and plasma metabolites of patients with some of the best-known autoinflammatory diseases (CAPS, TRAPS, FMF and MKD) and healthy subjects. The purpose is clusterize the different disorders to better characterize each pathology and try to distinguish the genetically undefined pathologies.

**Methods:** Monocytes (purified form peripheral blood and incubated for 4 hours with or without LPS) and plasma were collected from patients and healthy donors. The samples were processed and proteins or metabolites expression evaluated by an High Resolution/Mass Accuracy Liquid Chromatography Tandem Mass Spectrometry (HR/MA LC MS/MS). PCA analysis and Person’s correlation were used as quality control of experimental design, while the statistical analysis was performed with the Perseus software.

**Results:** We analyze the monocytes and plasma of patients with CAPS, TRAPS, FMF and MKD. We identified about 4000 proteins of each 3500 are quantified by LFQ approach. PCA analysis and Person’s correlation show a good reproducibility of data and a good separation between the different groups. In synchronous way using a cluster analysis and heatmap, based on a machine learning protein selection, we observed a protein signature specific for each group of pathology and for each condition of monocytes treatment. Furthermore we used a supervised multivariate analysis to try to identify a more specific list of proteins for each pathology: at the moment we obtained a list of about 40 protein per disease, significantly modulated if compared with healthy controls and comparing each pathology with the other.

**Conclusion:** Here, we addressed how an high resolution proteomics approach could be used to better understand the biology of autoinflammatory diseases. The characterization of a broad spectrum of proteins and metabolites and their interaction network will allow us to identify new biomarkers for the different pathologies and better comprehend and recognize the genetically undefined disorders.

**Disclosure of Interest:** None Declared.

### P11 Through new classification criteria for inherited periodic fevers and PFAPA syndrome: the patient validation phase

#### Federica Vanoni^1,2^, Silvia Federici^3^, Francesca Bovis^4^, Mariapia Sormani^5^, Nicola Ruperto^3^, Marco Gattorno^3^, Michaël Hofer^2^ and on behalf of the Expert Committee for the Classification Criteria in periodic fever

##### ^1^Departement of Pediatric of Southern Switzerland, Ospedale San Giovanni, Bellinzona, Switzerland; ^2^Pediatric Rheumatology Unit, CHUV, Lausanne, Switzerland; ^3^Pediatria II - Reumatologia, Istituto G. Gaslini, University of Genoa, Genoa, Italy; ^4^Biostatistics Unit, Department of Health Sciences, University of Genoa, Genoa, Italy; ^5^Biostatistics Unit, Department of Health Sciences, University of Genoa, Genoa, Italy

###### **Correspondence:** Federica Vanoni

**Introduction:** Provisional evidence-based classification criteria for inherited periodic fever (TRAPS, FMF, MKD and CAPS) have been recently developed. However, no consensus on how to combine clinical criteria, laboratory test and results of molecular analysis has been reached so far. Moreover, no validated evidence based set of classification criteria for PFAPA has been established so far. We conducted a multistep process, based on a combination of expert consensus and analysis of real patient, to develop new criteria for inherited periodic fevers and PFAPA syndrome.

**Objectives:** To obtain a consensus > 80% among experts on the diagnosis of a group of patients with inherited periodic fevers, PFAPA and undefined periodic fevers.

**Methods:** A panel of experts (25 clinicians and 8 geneticists) was asked to evaluate, via a web-based system on the basis of clinical and laboratory data, 360 real patients affected by AIDs (60 FMF, 60 TRAPS, 60 MKD, 60 CAPS, 60 PFAPA and 60 undefined periodic fever) randomly selected from the EUROFEVER Registry. PFAPA patient were evaluated only by clinicians. Experts were asked to classify each patient as having FMF, TRAPS, MKD, CAPS, PFAPA or undefined periodic fever syndrome. The criterion for agreement used was 80% consensus among raters.

**Results:** After four round of evaluation, 281 over 360 patients (78%) were classified with a consensus > 80% for a specific disease: 36 as FMF, 32 as CAPS, 56 as MKD, 39 as TRAPS, 37 as PFAPA and 81 as undefined periodic fever. In 50 cases the diagnosis changed respect to the diagnosis of the enrolling center. 25 over 36 FMF patients were homozygous or compound heterozygous for high penetrance variants while 4 over 36 patients were compound heterozygous with one high penetrance mutation associated with E148Q variant. 6 over 36 were heterozygous for high penetrance mutations and 1over 36 patients carried only the E148Q variant. Over the 32 CAPS patients, 28 had a genotype consistent with the disease while 4 patients carried a variant of unknown significance (V198M). None of the patients showing the Q703K variant or with negative genetic test was classified as CAPS by the experts. Regarding TRAPS only 1 over 15 patients carrying a low penetrance variants (R92Q, P46L) reached the consensus; none of the 2 patients with negative genetic analysis reached the consensus. Among MKD patients, 4 carried the V377I variant in heterozygous date and among these 2 reached the consensus.

**Conclusion:** Our process led to a consensus on the diagnosis for 281 patients affected by inherited periodic fevers and PFAPA. In the subsequent phase we had performed statistical analysis in which variables coming from the EUROFEVER Delphi survey will be tested on these patients, to identify the best sets of classification criteria (in term of sensitivity and specificity) for inherited periodic fever and PFAPA that have been discussed in the consensus conference held in Genoa in March 2017.

**Disclosure of Interest:** None Declared.

### P12 A preliminary study regarding to the levels of vitamin B12 and folate during colchicine treatment in children with FMF

#### Özge Kaba^1^, Mustafa Çakan^2^, Şerife Gül Karadağ^2^, Betül Sözeri^3^, Gonca Keskindemirci^1^, Nuray Aktay Ayaz^2^

##### ^1^Pediatrics, Kanuni Sultan Süleyman Research And Training Hospital, İstanbul, Turkey; ^2^Pediatric Rheumatology, Kanuni Sultan Süleyman Research And Training Hospital, İstanbul, Turkey; ^3^Pediatric Rheumatology, Ümraniye Research And Training Hospital, İstanbul, Turkey

###### **Correspondence:** Nuray Aktay Ayaz

**Introduction:** Familial Mediterranean fever (FMF) is an autosomal recessive autoinflammatory disease characterized by irregular periodic attacks of serosal inflammation. The gold standard in treatment is colchicine and it is very potent both in preventing attacks and subsiding inflammation and by that way avoiding emergence of amyloidosis.

**Objectives:** There is a hypothesis pointing out to the negative influence of colchicine on the intestinal absorption of vitamin B12. As colchicine treatment should be used lifelong, vitamin B12 deficiency due to malabsorption may ensue during the course of treatment. Although there is an adult study, there is no data for children with FMF under colchicine treatment.

**Methods:** Forty-six children with newly diagnosed FMF were enrolled to this prospective study. They were all started colchicine according to their age (0-5 years:0.5 mg, 5-10 years:1 mg, >10 years: 1.5 mg - maximum 2 mg). Vitamin B12 and folic acid levels were obtained at the first day of colchicine treatment, 3rd month and 6th month of the treatment. Mutation analysis of the patients and their clinical findings were also reported.

**Results:** Of 46 children involved to the study 18 (39.1%) were females and 28 (60.9%) were males. The mean age of beginning of the symptoms was 65.3 ± 45.5 months, the mean age of diagnosis was 97.13 ± 50.2 months. There was family history of FMF in 25 patients (54.3%). Fever was present in 40 (86.9%), peritonitis was in 35 (76%), pleuritis was in 11 (23.9%), arthritis was in 6 (13%) patients. The mean vitamin B12 values and folate values were significantly decreased during colchicine treatment. Vitamin B12 levels were 436.28 ± 174.43, 411.63 ± 196.70, 361.67 ± 142.58 at the beginning, on the 3^rd^ and 6^th^ months of the colchicine treatment respectively. Folate levels were 10.91 ± 3.45, 9.29 ± 4.37, 9.76 ± 5.64 at the beginning, on the 3^rd^ and 6^th^ months respectively.

**Conclusion:** There was significant vitamin B12 and folate level decrease on the 6th month of colchicine therapy in the children with newly diagnosed FMF. This may be related to ileal malabsorption of vitamin B12 and jejunal malabsorption of folate. Although the study was performed among a small number of children with FMF and a limited time period was reported, prolonged periods may show more significant decrements in the levels of vitamin B12 and folate.

**Disclosure of Interest:** None Declared

### P13 Longitudinal assessment of iron homeostasis in patients with newly diagnosed familial Mediterranean fever

#### Özge Kaba^1^, Nuray Aktay Ayaz^2^, Şerife Gül Karadağ^2^, Mustafa Çakan^2^

##### ^1^Pediatrics, Kanuni Sultan Süleyman Research and Training Hospital, İstanbul, Turkey; ^2^Pediatric Rheumatology, Kanuni Sultan Süleyman Research and Training Hospital, İstanbul, Turkey

###### **Correspondence:** Nuray Aktay Ayaz

**Introduction:** Familial Mediterranean Fever (FMF) is an autosomal recessive autoinflammatory disease, characterized by recurrent fever and self-limited paroxysmal attacks of serosal inflammation.

**Objectives:** The aim of this study was to evaluate whether iron homeostasis parameters in patients with AAA was improved by treatment with colchicine or not.

**Methods:** Forty-six pediatric patients with newly diagnosed FMF were included into the study. All patients were assessed in the attack-free periods for hemoglobin, hematocrit, platelet, serum amyloid A, iron, total iron binding capacity, and ferritin before the colchicine treatment, and on the 3^rd^ and 5^th^ months of colchicine treatment.

**Results:** We have found that there was a significant increase in mean hemoglobin values when compared the results of hemoglobin levels before the treatment and on the 5^th^ month of the treatment (p = 0,048; <0,05). Also, we have observed the same increase in iron levels (p═0,004). We have seen that there was a significant decrease in ferritin and SAA levels and platelet counts, reflecting the control of ongoing inflammation. However, no significant difference was found between mean total iron binding capacity value, mean hematocrit values (Table 13).

**Table 13:** **(abstract P13).** See text for description

	Before treatment	3th month of treatment	5th month of treatment	p*
HGB	12,11 ± 1,16 (12,08)	12,20 ± 1,03 (12,05)	12,38 ± 1,02 (12,31)	0,027^*^
HCT	38,05 ± 3,19 (38,02)	38,63 ± 3,45 (38,30)	38,82 ± 2,81 (38,27)	0,135
PLT	350.932 ± 110.096 (334.600)	310.671 ± 85.475 (309.850)	303.202 ± 81.570 (296.700)	<0,001^*^
SAA	192,19 ± 295,14 (38,95)	20,26 ± 46,58 (3,85)	9,68 ± 25,81 (3,30)	<0,001^*^
IRON	53,47 ± 29,56 (50,85)	66,76 ± 35,61 (56,50)	67,41 ± 31,15 (64,00)	<0,001^*^
TIBC	361,09 ± 52,93 (362,50)	364,78 ± 55,12 (365,00)	372,91 ± 48,92 (366,00)	0,163
FERRITIN	56,63 ± 42,46 (45,00)	34,69 ± 21,34 (30,55)	32,74 ± 23,80 (25,55)	0,002^*^

**Conclusion:** In conclusion, we think that colchicine may be effective in balancing the iron homeostasis that is disturbed by chronic inflammation in FMF.

**Disclosure of Interest:** None Declared

### P14 Population based study of frequency of carrying FMF mutation among Armenian females

#### Artashes Tadevosyan^1^, Tigran Avagyan^1^, Gayane Amaryan^2,3,4^, Hasmik Hayrapetyan^5^, Anush Budumyan^1^

##### ^1^Public Health, YEREVAN STATE MEDICAL UNIVERSITY, Yerevan, Armenia; ^2^Pediatrics, YEREVAN STATE MEDICAL UNIVERSITY, Yerevan, Armenia; ^3^National Pediatric Centre for Familial Mediterranean fever, Yerevan, Armenia; ^4^Arabkir” Joint Medical Centre - Institute of Child and Adolescent Health, Yerevan, Armenia; ^5^Centre of Medical Genetics - Primary Health Care, Yerevan, Armenia,

###### **Correspondence:** Tigran Avagyan

**Introduction:** The Familial Mediterranean fever (FMF; OMIM 249100) is the most common autoinflammatory syndrome in the group of hereditary periodic fever syndromes, widespread in Armenia. The existing information on frequency of carrying the mutations of FMF gene is hospital based data with subsequent recalculations. To our best knowledge this is first epidemiological population based study in Armenia.

**Objectives:** The objective is revealing the frequency of MEFV gene mutations and genotypes in Armenian female population in Republic of Armenia.

**Methods:** The proportionate random sampling technique was performed to select 173 females aged 18-19 years from Yerevan and all marzes (regions) of Armenia. Absence of history or clinical manifestation of FMF or any autoimmune inflammatory disease/syndrome was the premium inclusion criteria. Venous blood samples were taken in EDTA containing vials. Genomic DNA was isolated by means of “Puregene kit” (Gentra System, USA). Molecular-genetic testing performed in the laboratory of auto inflammation diseases of the Center of Medical Genetics and Primary Health Care that has an international certificate and is the best in the region. The most common for Armenian ethnicity 12 mutation were analyzed: M694V, M694I, M680I (G/C), M680I (G/A), V726A, E148Q, K695R, F479L, R761H, A744S, P369S, 1692del.

**Results:** 53 (30.6%) of females from total 173 carry at least one mutation of FMF gene. From tested 12 mutations only 9 were detected: M694V, M680I, V726A, E148Q, K695R, F479L, R761H, A744S, and P369S. The most frequent finding was M694V: 19 cases (38.8%), followed by V726A: 11cases (22.4%) and E148Q: 9 cases (18.4%). These three mutations cumulatively comprised almost 80% of all. Out of 53 carriers 49 were heterozygous and four carries two mutations. No homozygous were observed. E148Q mutation was presented in all four compound heterozygote carriers, two in combination with V726A and two with P369S.

**Conclusion:** The frequency of carrying of FMF gene mutation in Armenian non-symptomatic female population is 30.6%. The most common mutations are M694V, V726A, and E148Q. The M694V reported as the most frequent mutation in hospital settings as well, causing the most sever course of disease. Asymptomatic carrier of compound heterozygous genotype is rare, 2.3% only.

**Disclosure of Interest:** None Declared

### P15 Is routine periodic laboratory work-up necessary for paediatric MEFV (mediterranean fever) carriers

#### Balahan Balahan, Nesrin Gülez

##### Behçet Uz Children s Hospital, Izmir, Turkey

###### **Correspondence:** Balahan Balahan

**Introduction:** Familial Mediterranean fever (FMF) is the most common hereditary auto-inflammatory disease in the world. Although FMF is inherited autosomal recessively, some heterozygotes may express disease phenotype and require therapy. On the other hand, some of the patients presenting manifestations evocative of FMF might happen to be heterozygotes coincidentally due to the high frequency of MEFV variants in at-risk populations.To date, there is no study in the literature about how to follow-up MEFV heterozygotes who do not fulfil FMF criteria.

**Objectives:** To share a single-center experience of the long-term clinical and laboratory follow-up of paediatric MEFV carriers.

**Methods:** The hospital charts of 240 children who were heterozygous for MEFV variants were retrospectively reviewed. Among them, 119 heterozygous patients fulfilled paediatric FMF diagnostic criteria and were started colchicine in the first 6 months of follow-up. Ten patients who fulfilled PFAPA syndrome with one MEFV mutation were excluded. 42 patients did not continue to be followed-up at our unit. So, the rest 69 heterozygotes were included in this study. Families were advised to record an FMF diary; including presence of fever, abdominal pain, chest pain, arthritis, arthralgia, myalgia, and erysipelas like erythema and also were asked to record the duration of these symptoms. Patients were asked to admit to hospital in case of any advocative FMF symptom lasting at least 6 hours in order to check acute phase reactants. The children were also followed-up with their routine analysis and serum amyloid A (SAA) levels every 6 months. The data of the visits in which an infection was reported were not included. The averages of all laboratory parameters were then calculated.

**Results:** 39 children had pathogenic mutations and 30 children had MEFV variants of unknown significance. The most common MEFV variants were M694V (n = 24) and E148Q (n = 16). The mean age at admission was 7.3 ± 3 years. The mean follow-up was 3.2 ± 1.6 years (min:2 max:6 years).

The median of average SAA level was 3.5 (3-38) mg/L and the median of average CRP level was 0.1 (0.01-1.8) mg/dl and mean of average ESR was 11.3 ± 5.4 (5-27) mm/hour. The children with pathogenic mutations had significantly higher mean SAA levels than the children with variants of unknown significance (p = 0.018), however; the mean CRP and ESR were similar. Besides, the children with pathogenic mutations had significantly more fever episodes than the children with variants of unknown significance (p = 0.04). None of the children had persistent proteinuria in the follow-up. We started colchicine in only 2 patients who were M694V heterozygous because of initiation of typical disease symptoms accompanied by at least one increased acute phase protein during an attack. Both patients had family history for FMF and fulfilled the disease criteria after 2 years of follow-up. Neither of these patients had persistently elevated acute phase reactants in their routine follow-up.

**Conclusion:** The results of this study suggested that routine clinical follow-up is useful; however, routine periodic laboratory work-up is not necessary among MEFV carriers. To the best of our knowledge, this is the first study about the long-term periodic clinical and laboratory follow-up of MEFV carriers in the literature.

**Disclosure of Interest:** None Declared

### P16 Increased psoriasis frequency in patients with familial Mediterranean fever

#### Ezgi D. Batu^1^, Abdulsamet Erden^2^, Emrah Seyhoğlu^3^, Alper Sarı^2^, Hafize E. Sönmez^1^, Berkan Armagan^2^, Selcan Demir^1^, Emre Bilgin^3^, Levent Kılıç^2^, Ömer Karadağ^2^, Ali Akdoğan^2^, Yelda Bilginer^1^, Ihsan Ertenli^2^, Sedat Kiraz^2^, Sule A. Bilgen^2^, Umut Kalyoncu^2^, Seza Özen^1^

##### ^1^Division of Rheumatology, Department of Pediatrics, HACETTEPE UNIVERSITY FACULTY OF MEDICINE, Ankara, Turkey; ^2^Division of Rheumatology, Department of Internal Medicine, HACETTEPE UNIVERSITY FACULTY OF MEDICINE, Ankara, Turkey; ^3^Department of Internal Medicine, HACETTEPE UNIVERSITY FACULTY OF MEDICINE, Ankara, Turkey

###### **Correspondence:** Ezgi D. Batu

**Introduction:** Familial Mediterranean fever (FMF) is a periodic fever syndrome caused by *MEFV* mutations. FMF may be associated with psoriasis in some cases. The prevalence of psoriasis in normal Turkish population is 0.40%.

**Objectives:** We aimed to investigate the prevalence of psoriasis among FMF patients and their relatives.

**Methods:** FMF patients followed at Hacettepe University Adult and Pediatric Rheumatology Departments between January and August 2016 were included. FMF patients/their relatives were accepted to have psoriasis if the diagnosis was made by a dermatologist.

**Results:** 351 FMF patients (177 adults; 174 children) were included. The median (min-max) age of adult and pediatric patients was 35 (19-63) and 10 (2-18) years, respectively. Thirteen (3.7%) FMF patients (11 adults, 2 children) had psoriasis. Psoriasis was more common in adult than pediatric patients (p = 0.02). Psoriasis was present in 22 (12.4%) of adult and 9 (5.2%) of pediatric patients’ relatives (p = 0.023). The frequency of psoriasis in ≥1 relatives of FMF patients was found to be 8.8%. Abdominal pain and fever were significantly higher and arthralgia, arthritis, pleural chest pain, pericarditis, and erysipelas-like erythema were significantly less frequent in the pediatric group than adults (p < 0.05).

**Conclusion:** In our study, psoriasis was more common in FMF patients than the normal population. Thus, FMF patients should be questioned and carefully examined for psoriasis lesions and psoriasis family history. Prospective multicenter studies may be important to find the incidence of psoriasis in FMF.

**Disclosure of Interest:** None Declared

### P17 Periodic fever, aphtous stomatitis, pharyngitis and cervical adentitis (PFAPA) syndrome. A qualitative study of parent's experiences and strategies

#### Carina Sparud-Lundin^1^, Stefan Berg^2^, Anders Fasth^3^, Anna Karlsson^4^, Per Wekell^5^

##### ^1^Institute of Health and Care Sciences, Sahgrenska Academy, Goteborg, Sweden; ^2^Pediatric Immunology and Rheumatology, The Queen Silvia Children's Hospital, Goteborg, Sweden; ^3^Dept of Pediatrics, Institute of Clinical Sciences, Sahlgrenska Academy, Goteborg, Sweden; ^4^Dept of Rheumatology and Inflammation Research, Sahlgrenska Academy, Instutute of Medicine, Goteborg, Sweden; ^5^NU-Hospital Organisation, Dept of Pediatrics, Uddevalla, Sweden

###### **Correspondence:** Stefan Berg

**Introduction:** Periodic fever, aphthous stomatitis, pharyngitis and cervical adenitis (PFAPA) syndrome is a fairly common autoinflammatory disease. Still, knowledge of the effects of the recurrent fever episodes on the child and its family situation are limited.

**Objectives:** To examine the experiences of parents regarding the impact of the disease on the child's general well-being, the family's situation and how the family handles the associated challenges.

**Methods:** A qualitative approach was used, applying a modified version of Grounded theory for design, data collection and analysis. Data was collected from two different sources:

· Face-to face interviews with one of the parents of children diagnosed with PFAPA (n = 10, 6 mothers and 4 fathers)

· Communication between parents of children with PFAPA in a closed Facebook group

**Results:** The child´s well-being was highly affected by the symptoms during episodes. Parents experienced increased stress with constant fatigue, social constraints of family life and restricted career opportunities. Hope of a recovery was constantly present. Parents described a lengthy process, depicted in the following Grounded Theory core category: **From uncertainty to gradually managing and awaiting recovery.**

Subcategories (below) describes illness trajectory, illness representation and the experiences/impacts of the periodic condition:

**Uncertainty** (Harmlessness - severity)

·Worries · Fear · Frustration

**Assurance** (Establishment of diagnosis)

·Relief · Reassuring · Explaining · Guilt reducing

**Gradually managing** (Regularity - unpredictability)

·Suffering · Exhaustion · Isolation · Work-related limitations

Recovery

·Outgrowing PFAPA · Surgery (tonsillectomy)

**Conclusion:** Getting a diagnosis becomes very important for parents since this reduces uncertainty and feelings of guilt

**Disclosure of Interest:** None Declared

### P18 Aicardi-Goutières syndrome with a novel mutation in the SAMHD1 gene

#### Sorina Boiu^1^, Adrianos Nezos^2^, Isabelle Melki^3^, Argirios Dinopoulos^4^, Erato Atsali^1^, Clio Mavragani^2^, Vana Papaevangelou^5^, Dimitrios Boumpas^6^

##### ^1^Pediatric Rheumatology Unit, Third Department of Pediatrics, "Attikon" University Hospital, National and Kapodistrian University of Athens, Athens, Greece; ^2^Department of Physiology, School of Medicine, National and Kapodistrian University of Athens, Athens, Greece; ^3^Laboratory of Neurogenetics and Neuroinflammation, INSERM UMR1163, Institut Imagine, Paris, France; ^4^Pediatric Neurology Unit, Third Department of Pediatrics, "Attikon" University Hospital, National and Kapodistrian University of Athens, Athens, Greece; ^5^Third Department of Pediatrics, "Attikon" University Hospital, National and Kapodistrian University of Athens, Athens, Greece; ^6^Joint Rheumatology Program, Fourth Department of Medicine, “Attikon” University Hospital, National and Kapodistrian University of Athens, Athens, Greece

###### **Correspondence:** Sorina Boiu

**Introduction:** Type I interferonopathies are a clinically heterogeneous group of Mendelian disorders characterized by constitutive upregulation of type I interferon activity. Aicardi-Goutières syndrome (AGS) manifests as an early-onset encephalopathy and sometimes mimics a congenital viral infection. Over time, as many as 40% of patients develop chilblain skin lesions on the fingers, toes, and ears. Classical AGS phenotype was associated with mutations in seven genes encoding proteins involved in nucleotide metabolism and/or sensing: *TREX1* (AGS1), *RNASEH2A* (AGS2), *RNASEH2B* (AGS3), *RNASEH2C* (AGS4), *SAMHD1* (AGS5), *ADAR1* (AGS6), and *IFIH1* (AGS7). Mutations in these genes result in the induction of type I interferon production and upregulation of interferon stimulated genes. However, the true extent of the phenotype associated with pathogenic variants in the AGS-related genes is not yet known.

**Objectives:** To identify the genetic cause for a phenotype associating severe encephalopathy and chilblain skin lesions in a twenty years old male.

**Methods:** A twenty years old male presented since early infancy with violaceous, scaling lesions of the fingers and toes, resorption of distal phalanges, dystrophic nail changes and, violaceous lesions on the nose that worsened during the cold season. He was diagnosed at the age of 7 years with Cornelia de Lange syndrome (CdLS) based on the phenotype associating severe somatic and psychomotor retardation, microcephaly, hypertrichosis, synophrys, arched palate, strabismus, hearing loss, cryptorchidism and unilateral vesicoureteral reflux with kidney scarring. Laboratory investigations showed mild thrombocytopenia and positive anti-thyroid antibodies. No cerebral imaging was ever performed. The patient consulted in our Clinic because of his severe peripheral vasculopathy. The interferon type I signature was determined in the peripheral blood of the patient and four age/sex matched healthy controls by quantitative PCR for 3 interferon-stimulated genes (ISGs) (MX-1, IFIT-1 and IFI44); a type I interferon score was calculated as previously described (Nezos et al. J. Autoimmunity, 2015). For disease gene identification, a next-generation sequencing (NGS) panel followed by Sanger sequencing were performed.

**Results:** The type I interferon score was markedly higher (98 times higher) in our patient compared to healthy controls. Using a next-generation sequencing panel, followed by Sanger confirmation, the patient was found to be homozygous for a novel c.66delC/p.SerGlnfs*43 variant in *SAMHD1*. Although not seen before, considering that this lesion is predicted to act as a biallelic nonsense mutation, and in light of the clinical features, this result confirmed the diagnosis of an Aicardi-Goutières syndrome. A cerebral MRI will be performed to evaluate the cerebrovascular involvement in this patient, considering the risk of intracerebral large vessel involvement with catastrophic cerebral vascular accidents associated with mutations in *SAMHD1*.

**Conclusion:** We describe a novel mutation in *SAMHD1* in a patient with AGS*,* previously diagnosed with CdLS. It is important to consider the diagnosis of AGS in patients with neurologic symptoms in the presence of chilblain lesions.

**Disclosure of Interest:** None Declared

### P19 The challenges to diagnose and treat a rare patient with candle syndrome

#### Martin Boyadzhiev^1^, Veselin Boyadzhiev^1^, Lachezar Marinov^1^, Violeta Iotova^1^, Sophie Hambleton^2^, Ivona Aksentijevich^3^

##### ^1^Dept. of Pediatrics, Medical University, Varna, Bulgaria; ^2^Instituste of Cellular Medicine, Newcastle University, Newcastle Upon Tyne, United Kingdom; ^3^National Human Genome Research Institute, National Institutes of Health, Washington D.C., United States

###### **Correspondence:** Martin Boyadzhiev

**Introduction:** Chronic Atypical Neutrophilic Dermatosis with Lipodystrophy and Elevated temperature (CANDLE) is a rare recessively inherited autoinflammatory syndrome, characterized by early-onset recurrent fevers, skin lesions, periorbital swelling, lipodistrophy, arthritis, myositis, short stature and hepatomegaly. It is caused by defects in assembly of the proteasome complex. Thus far, mutations have been identified in 5 genes although most commonly they are found in the *PSMB8* gene.

**Objectives:** We present a 4 year old boy with a genetically confirmed diagnosis of CANDLE after presenting to our clinic with the typical clinical and laboratory features of the syndrome.

**Methods:** The patient was born at full-term and after uneventful pregnancy, to unrelated parents. At 20 days postnatal age a maculopapular rash appeared, initially confined to the extremities, and accompanied by fever. Over the next two years the rash spread to involve the face and body. He developed periorbital swelling, splenomegaly, leucocytosis, iron-deficiency anemia and thrombocytopenia. Fever, reaching up to 40 °C, was a consistent features of disease flares. Three skin biopsies were performed and showed evidence for perivascular and periadnexial infiltrations, consisting mainly of neutrophils. Bone marrow biopsy and immunologic investigations were unremarkable. Treatments with Hydroxychloroquine, Methotrexate and short courses of corticosteroid were tried with little to no effect.

At the age of 2 years and 10 months, the boy presented to our Clinic. Lipodystrophy, short stature (-4 SDS), typical facial features, longer IV-th and V-th fingers of both hands and hepatomegaly were noted in addition to the above features and a clinical diagnosis of CANDLE syndrome was made. Blood samples from the child and both parents were sent to NIH for genetic testing, revealing compound heterozygous mutations in the patient’s PSMB8 gene. The two mutations, p.A92V and p.K105Q, were previously associated with CANDLE. Each parent is heterozygous for one mutation.

**Results:** The patient has since been on treatment with oral Methylprednisolone that proved to be effective in controlling his skin rash and systemic inflammation at the cost of systemic side effects. Regular follow-up during the next two years showed nephrocalcinosis, which resolved with symptomatic therapy, and arterial hypertension, controlled by dual antihypertensive therapy. Preliminary reports in the literature showed that JAK-inhibitors might be very effective in controlling the disease activity and we hope to obtain this therapy for our patient.

**Conclusion:** CANDLE should be considered in patients who present with early-onset severe inflammation, typical skin rash, and lipodystrophy. Genetic testing is already available and although therapy is currently challenging, treatment with JAK-inhibitors holds promise for the future.

**Disclosure of Interest:** None Declared

### P20 Complement endorse the pathogenesis in autoinflammation

#### Juergen Brunner^1^, Wilfried Posch^2^, Evelyn Rabensteiner^1^, Doris Wilflingseder^2^

##### ^1^Pediatrics, Medical University Innsbruck, Innsbruck, Austria; ^2^Division of Hygiene and Medical Microbiology, Medical University Innsbruck, Innsbruck, Austria

###### **Correspondence:** Juergen Brunner

**Introduction:** The complement system represents a major part of the innate immune system, consisting of more than 30 different proteins in plasma and on cell surfaces and can be activated through three different pathways. Inflammasomes are also part of the innate immune system. A group of disorders in inflammasomes have been associated with autoinflammatory diseases (AIDs). Familial cold autoinflammatory syndrome (FCAS), Muckle-Wells syndrome (MWS) and chronic infantile neurological, cutaneous and articular syndrome/neonatal onset multisystem inflammatory disease (CINCA/NOMID) were originally described as three distinct diseases. After the identification of their common genetic origin, i.e. mutations in the NLRP3 gene on chromosome 1q44, they are perceived as a continuum of one disease entity and labelled cryopyrin-associated periodic syndromes (CAPS). Aim of this preliminary study in a patient with MWS was to find a correlation between the complement system and a disorder of autoinflammation.

**Objectives:** Aim of this preliminary study in a patient with MWS was to find a correlation between the complement system and a disorder of autoinflammation.

**Methods:** PBMCs (peripheral blood mononuclear cells) were isolated from blood of a healthy donor and of an individual suffering from MWS by density gradient centrifugation using a Ficoll Paque Premium (GE Healthcare). After washing, PBMCs were incubated with anti-human CD14 Magnetic Beads (BD) to obtain CD14+ monocytes. These were stimulated by addition of cytokines (IL-4 and GM-CSF) for five days to generate immature moDCs (iDCs), which were used for cytokine ELISAs and flow cytometric analyses. IL-6 and IL-1β cytokine ELISAs were performed according to the manufacturer (Biolegend) following stimulation of cells using either LPS or differentially complement opsonized HIV-1. Phenotypical characterization of pathogen-exposed DCs was performed by analyzing characteristic surface markers (CD11c, DC-SIGN (C-type lectin receptor on DCs, characteristic marker for iDCs), CD86) by multi-color flow cytometry.

**Results:** IL-1β production of iDCs is higher in the patients cells than in the cells of the healthy donor. However, the most significant difference was shown in complement opsonized iDCs. DC-SIGN is higher expressed in complement opsonized iDCs in patient cells compared to cells of a healthy donor (37,12% v28,64%). DC-SIGN is also higher expressed in the iDCs of the MWS patient after stimulation with LPS.

**Conclusion:** The complement system may play an important role in the development of an proinflammatory milieu in patients with disorders of autoinflammation. The phenomenon shown in a patient with MWS has to be reproduced in more MWS patients a well as in patients with other disorder of autoinflammation.

**Disclosure of Interest:** None Declared

### P21 Recurrent pericarditis: clinical course and treatment in pediatric patients

#### Camilla Celani^1^, Diala Khraiche^2^, Pierre Quartier^3^, Damien Bonnet^2^, Brigitte Bader-Meunier^1^

##### ^1^Unité d'Immunologie-Hématologie et Rhumatologie Pédiatriques, Hopital Necker, Paris, France^2^Cardiologie pediatrique, Hopital Necker, Paris, France^3^Unitè d'Immunologie-Hématologie et Rhumatologie Pédiatriques, Hopital Necker, Paris, France

###### **Correspondence:** Camilla Celani

**Introduction:** Recurrent pericarditis is a common complication of acute pericarditis and affects 15-30% of patients after an initial attack. The etiology is poorly understood and about 80% of these recurrent pericarditis are "idiopathic". However, some data suggest an autoimmune or autoinflammatory mechanism. Colchicine associated with nonsteroidal anti-inflammatory drugs (NSAIDs) is the treatment of choice and the use of other treatments remains exceptional.

**Objectives:** To analyze the clinical findings, course and treatment of pediatric patients with recurrent pericarditis.

**Methods:** Retrospective monocentric study. Inclusion Criteria :1) patients with at least twice recurrence of recurrent pericarditis (RP) ; 2) followed at Necker hospital between 2006 and 2016. Exclusion Criteria : 1)Known history of autoimmune or autoinflammatory disease.

**Results:** Thirteen patients (10 F and 3 M) with recurrent pericarditis were included. The median age at disease onset (first episode of pericarditis) was 11.3 years (range 8 - 17). Two groups of patients could be identified: In group 1 (6 patients), the recurrent pericarditis occurred after surgical correction of cardiac malformations; in group 2 (7 patients), there was no history of cardiac disease. During the episodes of pericarditis, all patients showed fever, chest pain, electrocardiographic changes and increased white blood cell counts and C reactive protein (CRP median 181 mg/l, polymorphonuclear cells 13619 g/l,) without differences between the two groups. A family history of recurrent pericarditis was noted in 2 patients from the first group. Six patients had pleuritis and/or pneumonia with concomitant pleural effusion (3 patients in each group). Patients in group 1 had more recurrences than in group 2 (median 2.8 vs 1.5) during the same follow-up period (one year). ANA > 1/640 (Anti-nuclear autoantibodies) and anti-DNA were present in 2 patients of each groups. The pericardial biopsy was performed in 5 patients and displayed fibrosis and showed[an inflammatory tissue with predominance of neutrophils. Initial treatments was aspirin with anti-inflammatory dose, alone (5 patients) or in combination with Colchicine (4 patients), colchicine alone (1 patients) or in combination with ibuprofen (1 patient) and antibiotic therapy (2 patients). To prevent recurrence, six of patients were treated with NSAIDs associated to colchicine or indomethacin alone. Corticotherapy was required in 2 patients in group 2 . Anakinra was introduced in one of them because of corticodependence. Clinical remission was obtained in all patients after discontinuation of the treatment (10 patients) or under treatment (3patients). A heterozygous mutation of R92Q for the TRAPS gene was found in 1 of the 6 patients studied. No mutation in the MEFV gene was found.

**Conclusion:** Recurrent pericarditis can develop after cardiac surgery or can be isolated. The presence of fever, inflammatory syndrome, pericardial neutrophilic infiltrate, and the efficacy of anti-IL1 in some patients suggests that some recurrent pericarditis could be considered as auto-inflammatory disease. Furthermore the existence of familial cases in two patients suggests a genetic susceptibility. In conclusion early anti inflammatory treatment should be considered in recurrent pericarditis associated with an inflammatory syndrome, after rulling out an infection.

**Disclosure of Interest:** None Declared

### P22 Sinogenic subdural empyema in two girls treated with Adalimumab for chronic recurrent multifocal osteomyelitis (CRMO)

#### Anne Estmann Christensen, Peter Toftedal

##### Hans Christian Andersen Children's Hospital, Odense University Hospital, Odense, Denmark

###### **Correspondence:** Anne Estmann Christensen

**Introduction:** Sinusitis is a rare cause of intracranial infection in children. The morbidity and mortality remains high in reported series.

**Objectives:** We report two cases of sinusitis complicated with intracranial empyema in patients treated with tumor necrosis factor-alfa monoclonal antibody (Adalimumab) for CRMO.

**Methods:** Case report

**Results:** The two girls were 11 years old when the infectious complication developed.

Patient 1 had been treated with adalimumab for 15 months, as she developed fever, acute pansinusitis and orbital cellulitis and abscesses during vacation. She was treated with intravenous antibiotics and drainage. The treatment was continued after returning home, and clinical improvement and normalisation of C-reactive protein (CRP) was achieved. Mild and unspecific symptoms of headache, double vision and swelling of the forehead remained and was initially thought of as sequela after surgery. Upon further investigation papilloedema was recognised and a CT scan of the brain showed subdural empyema, intra cerebral oedema located in the frontal lope, abscesses intra orbital and in the forehead, continued sinusitis and subperiosteal abscess and osteomyelitis (Potts puffy tumor). An aggressive rhino- and neuro-surgical approach combined with long-term broad-spectrum antibiotic treatment was initiated. Despite this, osteomyelitis in the frontal bone reoccurred several times, latest 1½ year after the initial infection. Immunosuppressive drugs have been discontinued at the beginning of the infectious period.

Patient 2 was treated with methotrexate for a year and received adalimumab for a period of 3 months. In these months she developed chronic rhinitis and allergy was suspected but not confirmed. When psoriasis appeared adalimumab was stopped, but the chronic sinusitis persisted. Two months later she was admitted with high fever and convulsions. She had discrete increased white cell count in the spinal fluid and a CT scan showed pansinusitis but no ostitis or intra cerebral changes. Broad-spectrum antibiotic treatment was started together with drainage of the sinuses. She improved clinically but CRP remained elevated. A MRI of the head was planned, but before it was performed another convulsion occurred. MRI showed pansinusitis, osteitis and subdural empyema. Functional endoscopic sinus surgery (FESS) was performed and the girl is now improving on long-term antibiotic treatment. Methotrexate treatment was stopped at the initial symptoms of meningitis.

**Conclusion:** Tumor necrosis factor-alfa monoclonal antibody is an efficient anti-inflammatory drug used in treatment of several inflammatory diseases. The increased risk of infections is well known. Special attention should be drawn towards symptoms of chronic rhinitis/sinusitis, as severe intra cerebral complications, such as subdural empyema, may develop. Children with sinusitis and any neurologic finding, signs of complicated sinusitis such as Pott's puffy tumor or orbital cellulitis, or persistent headache, fever, or nausea and vomiting should have additional evaluation for sinogenic subdural empyema.

Written consent for publication has been obtained from the parents.

**Disclosure of Interest:** None Declared

### P23 Spectrum of chronic recurrent multifocal osteomyelitis in a Belgian cohort of 25 children: clinical presentation, imaging, treatment and outcome

#### Lien De Somer^1^, Sara Kaut^2^, Ine Van den Wyngaert^2^, Davy Christiaens^3^, Carine Wouters^1^, Steven Pans^3^

##### ^1^Department of Pediatric Rheumatology, UZ Leuven, Leuven, Belgium; ^2^Pediatrics, UZ Leuven, Leuven, Belgium; ^3^Radiology, UZ Leuven, Leuven, Belgium

###### **Correspondence:** Lien De Somer

**Introduction:** Chronic Recurrent Multifocal Osteomyelitis (CRMO) is a rare auto-inflammatory disorder, characterized by recurrent episodes of bone pain. Diagnosis can be challenging and is based on exclusion. Laboratory investigations, radiology and histology are necessary to make a differential diagnosis with malignancy, infectious osteomyelitis and juvenile idiopathic arthritis.

**Objectives:** To document clinical characteristics of pediatric patients diagnosed with CRMO. To collect data on outcome and management of the disease.

**Methods:** We reviewed clinical characteristics, radiological data and treatment in pediatric CRMO patients, followed at the pediatric rheumatology department of the University Hospital of Leuven.

**Results:** Twenty-five patients were enrolled, with a mean age of 10.1 years at onset of the disease. The mean age at diagnosis was 11.2 years, with a mean diagnostic delay of 14 months. Bone pain was the leading symptom (24/25 patients). On imaging, 148 lesions were identified with an average of 5,9 lesions per patient. The most common sites of involvement were the vertebrae (37%) and lower limbs (31%), followed by the pelvis (10%) and clavicle (9%). In almost half of patients (12/25) monotherapy with NSAIDs was sufficient to obtain remission. The remaining 13 patients received bisphosphonates as 2nd-line therapy. Methotrexate was prescribed in 5 patients. Two patients needed further therapy with biologicals: etanercept (n = 2) and tocilizumab (n = 1). Disease was in remission in 15/25 after a mean time of 22.7 months. The prognosis was worse in patients with spinal involvement, resulting in more long-term sequelae

**Conclusion:** We present a unique pediatric cohort of 25 CRMO patients. A typical pattern of bone involvement could be found on MRI. Lesions presented as areas of bone marrow edema, with an abnormal hyperintensity on STIR images and/or abnormal hypointensity on T1-weighted images and/or areas of contrast enhancement. NSAIDs were administered as first-line treatment. Second-line strategies included bisphosphonates, glucocorticosteroids, methotrexate, etanercept and tocilizumab

**Disclosure of Interest:** None Declared

### P24 Clinical characteristics of familial Mediterranean fever patients according to their MEFV mutation

#### Pelin Esmeray^1^, Zehra S. Arıcı^2^, Hafize E. Sönmez^2^, Yelda Bilginer^2^, Seza Özen^2^

##### ^1^Department of Pediatrics, Hacettepe University Faculty of Medicine, Ankara, Turkey; ^2^Department of Pediatrics, Division of Rheumatology, Hacettepe University Faculty of Medicine, Ankara, Turkey

###### **Correspondence:** Pelin Esmeray

**Introduction:** Familial Mediterranean fever (FMF) is the most common autoinflammatory disease in the world. It is an autosomal recessive disease caused by mutations in *MEFV*.

**Objectives:** The aim of this study is to evaluate the clinical characteristics of FMF patients in relation with their *MEFV* mutations.

**Methods:** 378 patients (0-18 years), who were followed up at the Department of Pediatric Rheumatology in Hacettepe University, Ankara, Turkey, between January 2005 and October 2016, were included in the study. Mutations in *MEFV* were evaluated in all of the patients. The clinical characteristics, disease activity, need for additional treatment and acute phase reactants were compared according to the mutations of the patients. We have also compared after dividing patients into two groups as patients with high-risk mutations (M694V/M694V, M680I/M680I, M694V/M680I) and others.

**Results:** 36.7% of the patients were homozygous for M694V. 14.3% M694V/M680I, 10% M694V/V726A, 9% M694V/-, 6% M694V/E148Q, 3.7% M680I/V726A, and 2.9% were M680I/M680I. The patients with M69V/M694V were significantly younger at disease onset than other patients (3 years vs 4 years, respectively; p = 0,021). In addition, the disease duration, disease activity, acute phase reactants, and AIDAI(auto-ınflammatory disease activity index) values were higher and need for additional therapy was more frequent among patients with M694V/M694V compared to others.The disease activity, acute phase reactants were higher and need for additional therapy was more frequent in patients with high-risk mutations than others (Table 14).

**Table 14:** **(abstract P24).** Clinical characteristics and laboratory findings of M694V homozygote patients

	Others (n = 240)	M694V-Homozygote (n = 138)	p
Age of diagnosis (years),	5 (0-17)	4 (1-15)	0,098^*^
Disease duration (years),	6 (0-17)	7 (1-17)	0,004^*^
Attack status in last 6 months, n (%)	72 (30,0)	65 (47,1)	0,001^**^
Attacks frequency in last 6 months	2 (1-19)	2 (1-15)	0,722^*^
Disease activity score	0 (0-5)	1 (0-6)	<0,001^*^
AIDAI,	0 (0-14)	1 (0-9)	<0,001^*^
Regular drug use (n = 192), n (%)	86 (64,7)	38 (64,4)	0,973^**^
Need for addditional treatment (n = 192), n (%)	7 (5,3)	13 (22,0)	<0,001^**^

n: number of patient; %: Column percentage; *Mann-Whitney U Test; **Pearson chi-Square Test

**Conclusion:** The disease phenotype is more severe in patients with high-risk mutations. Therefore, close follow-up is critical for these patients.

**Disclosure of Interest:** None Declared

### P25 Infection status in children with familial Mediterranean fever (FMF) according to MEFV

#### Pelin Esmeray^1^, Ezgi D. Batu^2^, Zehra S. Arıcı^2^, Hafize E. Sönmez^2^, Yelda Bilginer^2^, Seza Özen^2^

##### ^1^Department of Pediatrics, Hacettepe University Faculty of Medicine, Ankara, Turkey; ^2^Department of Pediatrics, Division of Rheumatology, Hacettepe University Faculty of Medicine, Ankara, Turkey

###### **Correspondence:** Pelin Esmeray

**Introduction:** Familial Mediterranean fever (FMF) is the most common auto-inflammatory disease which is characterized by recurrent, self-limited flares of fever associated with polyserositis. The association with infectious disease is not clear except for the association with helicobacter pylori infection.

**Objectives:** In this study, the aim was to assess whether there was a tendency to infections.

**Methods:** 197 children with FMF who were followed at the Department of Pediatric Rheumatology in Hacettepe University, Ankara, Turkey between May 2016 and October 2016 were included in the study. In immunodeficiencies, the tendency for infections are defined as acute otitis media, sinusitis and pneumonia frequencies in a year, intravenous antibiotic requirement, presence of abscess, presence of recurrent urinary tract infections and acute gastroenteritis. We have assessed these frequencies in our patient groups according to mutations and attack frequencies.

**Results:** 30.4% of the patients were in the M694V homozygote group and 42.1% were in high-risk mutation(M694V/M694V, M680I/M680I, M694V/M680I) group. When examining M694V homozygous patients; 3% of patients had more than 4 acute otitis media per year, 6.7% had sinusitis more than 4 per year, and 1.7 had more than 4 per month of upper respiratory tract infection. All of the patients had less than 2 pneumonia per year. Only 6.7% of patients needed intravenous antibiotics. Recurrent urinary tract infection was present in 3.3% and abscess was 1.7% of patients. 13.4% of the patients had a history of acute gastroenteritis.

When examining high-risk mutations patients; 3.6% of patients had more than 4 acute otitis media per year, 4.8% had sinusitis more than 4 per year, and 2.4% had more than 4 per month of upper respiratory tract infection. All of the patients had less than 2 pneumonia per year. 4.8% of patients needed intravenous antibiotics. Recurrent urinary tract infection was present in 3.6% and abscess was 1.2% of patients. 14.4% of the patients had a history of acute gastroenteritis.

There was no statistically significant difference in the incidence of infection between mutation groups.

When the patients who had an attack in the last 6 months were examined, there was no significantly difference in infection status.

When the patients, who had increase in any infection frequency, were evaluated according to the mutation groups and attack status in the last 6 months, there was no significantly difference between the groups.

**Conclusion:** FMF patients are not inclined to develop infections as in immunodeficiencies. It may be speculated that the abnormality in the innate immune system in these patients does not significantly inpair host defense. More extensive work on this issue may be needed.

**Disclosure of Interest:** None Declared

### P26 Clinical characteristics of FMF patients according to age groups

#### Pelin Esmeray^1^, Ezgi D. Batu^2^, Hafize E. Sönmez^2^, Selcan Demir^2^, Yelda Bilginer^2^, Seza Özen^2^

##### ^1^Department of Pediatrics, Hacettepe University Faculty of Medicine, Ankara, Turkey; ^2^Department of Pediatrics, Division of Rheumatology, Hacettepe University Faculty of Medicine, Ankara, Turkey

###### **Correspondence:** Pelin Esmeray

**Introduction:** Familial Mediterranean fever (FMF) is the most common autoinflammatory disease in the world. The disease course could be different in different age groups.

**Objectives:** In this study, we aimed to study the disease characteristics, treatment compliance and need for additional treatment according to age groups of FMF patients.

**Methods:** 378 FMF patients (0-18 years) followed at the Department of Pediatric Rheumatology in Hacettepe University, Ankara, Turkey, between January 2005 and October 2016 were included in the study. The patients were evaluated in three groups according to their age at disease onset; 0-5, 6-11, 12-18 years. The treatment compliance was evaluated in 199 out of 378 patients according to their present ages.

**Results:** The disease onset was ≤5 years in 69%, 6-11 years in 26%, and ≥12 years in 5% of patients. Fever symptom was significantly less frequent in the ≥12 years group than the other age groups. The characteristics of patients according to age groups (at symptom onset) are presented in Table 15. The disease onset was significantly younger in patients homozygous for M694V mutation than patients who were negative for M694V (p = 0.021). 64,6% of 199 patients were compliant to colchicine treatment. When we grouped these patients according to their present age, ≤5 years group was the most compliant and the adolescent patients (≥12 years of age) was the less compliant to treatment (p < 0.05).

**Table 15:** **(abstract P26).** See text for description

		Onset age		p
	≤5 yaş (n = 261)	6-11 yaş (n = 98)	≥12 yaş (n = 19)	
Age (year), median (min-max)	10 (2-18)	13 (7-18)	17 (14-18)	<0,001^*^
Sex, n (%) Male Girl	143 (54,8) 118 (45,2)	56 (57,1) 42 (42,9)	10 (52,6) 9 (47,4)	0,897^**^
Attack Status in Last 6 Months n (%)	100 (38,3)	29 (29,6)	8 (42,1)	0,237^**^
Attack frequency in the last 6 months median (min-max)	2 (1-19)	2 (1-6)	1,5 (1-3)	0,226^*^
ISSF, median (min-max)	1 (0-6)	1 (0-5)	1 (0-4)	0,103^*^
Treatment compliance (n = 192), n (%)	84 (66,7)	31 (57,4)	9 (75,0)	0,363**
Need for additional treatment (n = 192), n (%)	12 (9,5)	7 (13,0)	1 (8,3)	0,764**

**Conclusion:** The disease course may be different in different age groups in FMF. FMF may present with less typical phenotype when the symptoms start at an older age (less fever symptom in adolescent. Treatment compliance is very critical in FMF patients and our results have emphasized that poor compliance is an important problem in especially adolescent patients.

**Disclosure of Interest:** None Declared

### P27 Oral findings in children with familial Mediterranean fever

#### Pelin Esmeray^1^, Ezgi D. Batu^2^, Zehra S. Arıcı^2^, Tülin I. Keçeli^3^, Yelda Bilginer^2^, Meryem Tekçiçek^3^, Seza Özen^2^

##### ^1^Department of Pediatrics, Hacettepe University Faculty of Medicine, Ankara, Turkey; ^2^Department of Pediatrics, Division of Rheumatology, Hacettepe University Faculty of Dentistry, Ankara, Turkey; ^3^Deparment of Pediatrics Pedodontology, Hacettepe University Faculty of Dentistry, Ankara, Turkey

###### **Correspondence:** Pelin Esmeray

**Introduction:** Familial Mediterranean fever (FMF) is the most common auto-inflammatory disease which is characterized by recurrent, self-limited flares of fever associated with polyserositis. Oral apthosis and periodontitis may be seen in the course of the disease. However we lack studies on the dental care in pediatric FMF patients.

**Objectives:** In this study, the aim was to report the oral findings and to check the effects of tooth decay on disease course in children with FMF.

**Methods:** 199 children with FMF who were followed at the Department of Pediatric Rheumatology in Hacettepe University, Ankara, Turkey between May 2016 and October 2016 were included in the study.

**Results:** The characteristics of patients are presented in Table 16. The mean age of patients was 11.24 ± 4.04 years. Tooth decay was observed in 182 (91.5%) patients. Tongue abnormalities in 65 (32.8%), lip abnormalities in 13 (6.6%), buccal abnormalities in 6 (3%). Oral aphthosis occured in 21 (10.6%), and recurrent oral aphthosis in 67 (33.8%) patients. The median (min-max) dental plaque index was 1.26 (0-2.25), and the median gingival index was 0.87 (0-1.81). Erythrocyte sedimentation rate and the attack frequency at the last six months were both higher in the patients with tooth decay than patients who did not have decay. Tooth decay was more common away M694V homozygous patients (98.3% vs 88.6%, p = 0.025; respectively).

**Table 16:** **(Abstract P27).** The demographic and laboratory features of the patients

	All patients (n = 199)	Patients with tooth decay (n = 182)	Patients who did not have tooth decay (n = 17)
	median (minimum-maximum)		
The status of attack at the last six months, n (%)	115 (57.7)	75 (97.4)	2 (2.6)
The frequency of attacks at the last six months (n = 115)	0.96 ± 2.08/0 (0-19)	2 (1-19)	2.5 (2-3)
	n (%)		
Deciduous tooth decay	166 (83,4)		
Permanent tooth decay	104 (52,3)		
Tooth decay	182 (91,5)		
Dental plaque index	1.26/198 (0-2.25)		
Gingival index	0.87/198 (0-1.81		

**Conclusion:** The frequency of the tooth decay was higher in our patient group compared to previously reported Turkish figures(91.5% vs 69.8-73.1%; respectively). The attack frequency at the last six months was higher away patients with tooth decay. Our results may suggest that dental problems may be a trigger for inflammatory attack in FMF. On the other hand, the chronic inflammation itself may be a risk factor for tooth decay in FMF.

**Disclosure of Interest:** None Declared

### P28 Successful use of tocilizumab, interleukin-6 (IL-6) inhibitor, in a child with TRAPS

Abstract withdrawn

### P29 The Eurofever project: an update on the longitudinal stage

#### Martina Finetti, Silvia Federici, Joost Frenkel, Seza Ozen, Helen Lachmann, Fabrizio De Benedetti, Joost Swart, Luca Cantarini, Romina Gallizzi, Marco Cattalini, Rolando Cimaz, Donato Rigante, Jordi Anton, Maria Alessio, Alma Nunzia Olivieri, Pavla Dolezalova, Annette Jansson, Giovanna Fabio, Judith Sanchez Manubens, Eric Hachulla, Rita Consolini, Karoline Krause, Zelal Ekinci, Jurgen Brunner, Isabelle Koné-Paut, Giovanni Filocamo, Maria del Carmen Pinedo, Efimia Papadopoulou-Alataki, Liliana Bezrodnik, Alberto Martini, Nicola Ruperto, Marco Gattorno and on behalf of Eurofever Project

##### Pediatria II - Reumatologia, Istituto Giannina Gaslini on behalf of Eurofever Project, Genoa, Italy

###### **Correspondence:** Martina Finetti

**Introduction:** In 2008 the Paediatric Rheumathology European Society (PReS) promoted an International Project (EAHC, Project No2007332) for the study of Autoinflammatory Diseases (AIDs) named Eurofever, whose main purpose is to create a web-based registry for the collection of clinical, laboratory, instrumental and response to treatment information in patients with AIDs.

**Objectives:** To implement the Registry with the new recently described AIDs and to increase the collection of longitudinal data, particularly regarding treatment and safety.

**Methods:** The data analysed in the study were extracted from the Eurofever registry, which is hosted in the PRINTO website (http://www.printo.it). From February 2015 we started the longitudinal collection of follow-up data for the patients already included in the Registry with particular focus on treatment, modification of the clinical picture, onset of complication/sequelae/adverse events.

**Results:** Up to date 3648 patients (M:F = 1799:1849) have been enrolled in the Registry from 60 different countries (all of them with available baseline demographic information). In 3183 pts (87%) complete clinical information and response to treatment data from disease onset to diagnosis are also available. For each disease the number of enrolled patients is: FMF 1012 pts (877 with complete clinical data); CAPS 289 pts (268 with complete clinical data); TRAPS 272 pts (254 with complete clinical data); MKD 203 pts (188 with complete clinical data); Blau’s disease 50 pts (26 with complete clinical data); PAPA 35 pts (all of them with complete clinical data); NLRP-12 mediated periodic fever 13 pts (10 with complete clinical data); DADA2 10 pts (6 with complete clinical data); SAVI 3 pts (all of them with complete clinical data); DIRA 3 pts (all of them with complete clinical data); Majeed 3 pts (all of them with complete clinical data); CANDLE 1 pt (with complete clinical data). Among multifactorial autoinflammatory diseases: PFAPA 653 pts (530 with complete clinical data); CRMO 535 pts (513 with complete clinical data); 340 pts with undefined periodic fever (269 with complete clinical data); Bechet disease 215 pts (186 with complete clinical data) and Schnitzler syndrome 11 pts (all of them with complete clinical data).

Longitudinal data are available for 393 pts (11%, M:F = 190:203). For each disease, the number of patients with at least one follow-up visit is: FMF 72 pts, CAPS 36 pts, TRAPS 32 pts, MKD 29 pts, PAPA 6 pts, DADA2 3 pts, SAVI 1 pt, DIRA 1 pt. Among multifactorial diseases: CRMO 99 pts, Bechet disease 67 pts, undefined periodic fever 29 pts, PFAPA 14 pts, Schnitzler syndrome 4 pts.

**Conclusion:** The enrolment in Eurofever Registry is still ongoing. The longitudinal collection and analysis of data coming from the Registry will improve our knowledge both on the natural history of the single disease and on the efficacy/safety of treatment commonly used in the clinical practice.

**Disclosure of Interest**: M. Finetti: None Declared, S. Federici: None Declared, J. Frenkel: None Declared, S. Ozen: None Declared, H. Lachmann: None Declared, F. De Benedetti: None Declared, J. Swart: None Declared, L. Cantarini: None Declared, R. Gallizzi: None Declared, M. Cattalini: None Declared, R. Cimaz: None Declared, D. Rigante: None Declared, J. Anton: None Declared, M. Alessio: None Declared, A. N. Olivieri: None Declared, P. Dolezalova: None Declared, A. Jansson: None Declared, G. Fabio: None Declared, J. Sanchez Manubens: None Declared, E. Hachulla: None Declared, R. Consolini: None Declared, K. Krause: None Declared, Z. Ekinci: None Declared, J. Brunner: None Declared, I. Koné-Paut: None Declared, G. Filocamo: None Declared, M. D. C. Pinedo: None Declared, E. Papadopoulou-Alataki : None Declared, L. Bezrodnik: None Declared, A. Martini Grant/Research Support from: Starting from 1 march 2016 I became the Scientific Director of the G. Gaslini Hospital, therefore my role does not allow me to render private consultancies resulting in personal income. I perform consultancy activities on behalf of the Gaslini Institute for the following companies: Abbvie, Boehringer, Novartis, R-Pharm. The money received for these activities are directly transferred to the Gaslini Institute's bank account., N. Ruperto Grant/Research Support from: The G. Gaslini Hospital, which is the public Hospital where I work as full time public employee, has received contributions from BMS, Hoffman-La Roche, Janssen, Novartis, Pfizer and Sobi for the coordination activity of the PRINTO network. This money has been reinvested for the research activities of the hospital in fully independent manners besides any commitment with third parties., Speaker Bureau of: Abbvie, Ablynx, AstraZeneca, Baxalta Biosimilars, Biogen Idec, Boehringer, Bristol Myers Squibb,, Eli-Lilly, EMD Serono, Gilead Sciences, Janssen, Medimmune, Novartis, Pfizer, Rpharm, Roche, Sanofi., M. Gattorno Grant/Research Support from: Unrestricted grant from SOBI and NOVARTIS

### P30 Mevalonate-kinase deficiency in the Czech Republic

Abstract withdrawn

### P31 Eye manifestations of patients with Muckle-Wells syndrome

#### sukru cekic^1^, ozgur yalcinbayır^2^, Sara Sebnem Kilic^1^

##### ^1^Pediatric Rheumatology, Uludag University Medical Faculty, Bursa, Turkey; ^2^ophthalmatology, Uludag University Medical Faculty, Bursa, Turkey

###### **Correspondence:** Sara Sebnem Kilic

**Introduction:** CAPS is a rare autoinflammatory disease associated with mutations in the *CIAS1* gene, encoding for NLRP3 that result in overactivation of the inflammasome and systemic inflammation. Muckle-Wells syndrome (MWS) is a rare autosomal dominant disease which causes episodic fever attacks, sensorineural deafness, recurrent hives, arthritis and eye involvement.

**Objectives:** Here we present the findings of eye involvement in a family whose 11 members have MWS.

**Methods:** Clinical data was collected during the course of ongoing patient care.

**Results:** We evaluated the clinical features of 11 patients who were referred to a tertiary care center. The median age of the patients was 25 years (range: 9-65). The ratio of females/males was 1.2 (6/5). All patients had arthritis with exacerbation on exposure to cold and recurrent episodes of pink eye. The median age of onset of ocular involvement was 8 years (2-45). We observed severe eye involvement in 27% of our cases, including band keratopathy, severe damage of corneal stroma and neovascularization. Corneal involvement and clouding was detected in four patient. Two of those had the diagnosis of keratoconus as well. Patients with keratoconus had corneal scarring due to corneal hydrops verified with corneal topography. The other two patients with corneal clouding had bant keratopathy. One of those patient was a 17 year old girl who had recurrent uveitis with hypopyon which necessiated the use of intravitreal dexamethasone implant. She also had posterior synechia of the iris to the lens. The other eye of that patient had signs of phthisis bulbi. The other patient with bant keraopathy was 46 years old male who had optic atrophy and tractional fibrovascular membranes at the posterior pole of the eye. Anakinra was used for treatment of 5 cases, and canakinumab of 3 cases. It was observed that the frequency of conjunctivitis decreased after anti IL-1 therapy. There was no mutation detected in the study of MEFV (all exons), TNFRSF1A (exons 2 to 7), MVK (all exons), NLRP3 (all exons), NOD2 (exons 4, 8 and 9) and PSTPIP1 (exons 10 and 11) genes.

**Conclusion:** In this study, it has been shown that eye findings related to MWS can vary from conjunctivitis to severe uveitis. We want to emphasize that ocular involvement in MWS should be carefully assessed, since it can lead to visual impairment.

Trial registration identifying number:

**Disclosure of Interest:** None Declared

### P32 Clinical comparison of patients with unifocal and multifocal chronic recurrent multifocal osteomyelitis in an Irish cohort

#### Daire O'Leary^1^, Anthony Gerard Wilson^2^, Emma-Jane MacDermott^3^, Orla Killeen^3^

##### ^1^Paediatric Department, RCSI, Dublin, Ireland; ^2^School of Medicine, UCD, Dublin, Ireland; ^3^National Centre for Paediatric Rheumatology, Our Lady's Children's Hospital, Dublin, Ireland

###### **Correspondence:** Orla Killeen

**Introduction:** Chronic recurrent multifocal osteomyelitis (CRMO) is an auto-inflammatory condition primarily affecting children with an estimated prevalence of 1 per 10^6^. The umbrella term CRMO includes patients unifocal and multifocal disease that meet the diagnostic criteria.

**Objectives:** To describe clinical phenotype of an Irish cohort of patients with CRMO and compare those with unifocal and multifocal disease.

**Methods:** This study was a retrospective chart review of a prospectively gathered cohort of patients attending a single paediatric rheumatology centre.

**Results:** Since 2006, 42 patients have been diagnosed with CRMO at the National Centre for Paediatric Rheumatology (NCPR) and 5 of these have unifocal disease. Patients with unifocal disease had a single site involved throughout the disease. Three patients with unifocal disease had a single symptomatic episode. All patients met the diagnostic criteria proposed by Jansson et al and are ethnically Irish. Overall the mean age at diagnosis was 10.4 years, diagnostic delay was 0.78 years and number of physicians seen prior to referral to paediatric rheumatology was 3.5. All patients with unifocal disease and 68% of patients with multifocal disease were female (overall F:M = :1). The distribution of lesions was similar in both groups. In patients with unifocal disease, all underwent biopsy to confirm the diagnosis, none had systemic symptoms at presentation, synovitis at any stage or a personal history/first-degree relative with an associated autoimmune disease. In patients with multifocal disease, 81% underwent biopsy, 24% had systemic symptoms at presentation, 21% had synovitis and 38% had a personal history/first-degree relative with autoimmune disease. 1 patient (20%) with unifocal disease was refractory to NSAID treatment and received methotrexate. 14 patients (38%) with multifocal disease were refractory to NSAID treatment and received methotrexate, corticosteroids, anti-TNF, pamidronate or a combination of these.

**Conclusion:** The clinical phenotype of patients with unifocal CRMO appears to be different to those with multifocal disease. It may be less frequently associated with other autoimmune or autoinflammatory conditions and more frequently responsive to NSAID treatment alone. However, the number of patients with unifocal disease in this cohort was low.

**Disclosure of Interest:** None Declared

## Bone in rheumatic diseases

### P33 Magnetic resonance imaging bone marrow oedema in children: insight to comprehension and clinical significance

#### Federico Caldonazzi^1^, Sara Pieropan^2^, Maddalena Maschio^3^, Gloria Dallagiacoma^2^, Daniela Degani^3^, Giorgio Piacentini^3^, Diego A. Ramaroli^3^, Domenico Biasi^2^, Maurizio Rossini^2^

##### ^1^UOC di Pediatria, Universita' degli Studi di Verona, Verona, Italy; ^2^UOC di Reumatologia, Università degli Studi di Verona, Verona, Italy; ^3^UOC di Pediatria, Università degli Studi di Verona, Verona, Italy

###### **Correspondence:** Federico Caldonazzi

**Introduction:** Bone marrow oedema in a child is a rare and uncommon condition associated with joint and bone pain exacerbated by weight bearing.

**Objectives:** We re-evaluated in the period of 2015-2016 seven pediatric patients referred to our Rheumatology Clinic for persistent foot pain with MRI oedema of the feet bones (interpreted commonly as algodystrophic syndrome manifestation); they had all been misdiagnosed in other institutions as affected by algodystrophy of the ankle/feet or complex regional pain syndrome (CRPS).

**Methods:** All the patients were re-evaluated with clinical examination, blood samples of bone turnover and vitamin D levels.

**Results:** Mean age of the 7 patients (F:M 6:1) was 11 years. 3 of the patients (43%) had a previous diagnosis of oligoarticular juvenile idiopathic arthritis ANA+ with the disease in complete remission at the moment of the clinical evaluation; 4 patients did not suffer from any chronic disease.

Physical examination did not reveal any skin color change (rubor) but intense pain limited to the skeletal sites of feet and ankles; only two patients (28%) had a slight swelling of the feet.

6 of the patients suffered from joint hypermobility with recall of minor ankle strain prior to the symptoms onset as precipitating event after meticulous history; 1 was a football player and reported recurrent feet microtrauma.

1 patient had been treated in another institution with bisphosphonates without any improvement. Bone turnover markers were normal in the 6 patients not treated previously with bisphosphonates; vitamin D was below the lower limits in all patients (72% < 20 ng/mL).

All Patients were treated with an adequate daily vitamin D intake and symptomatic pain relief was achieved with low dose NSAIDs (ibuprofen) or a short course of steroid treatment (prednisone); rest and physical therapy were recommended; vitamin C was added when diet intake was too low according to minimal daily diet recommendations. Clinical improvement appeared within a 2 to 3 month treatment period in all patients; MRI performed in the 3 patients with over 6 month of treatment documented complete regression of bone marrow oedema.

**Conclusion:** Symptoms and disability out of proportion to the clinical findings is a clinical key feature of MRI bone oedema of painful skeletal sites in children; differential diagnosis with the multifacet pediatric CRPS is possible when bone oedema is associated with limited clinical findings; it may be secondary to underestimated articular microtraumatisms in a bone turnover milieu of vitamin D deficiency; we underline the importance of supplementing vitamin D until the age of 18 as daily drops, in order to avoid its deficiency, which, in combination with other environmental and predisposing factors, could be the cause of invalidating clinical complaints.

**Disclosure of Interest:** None Declared

### P34 Performance of bone scan compared to whole body-MRI in evaluation of chronic non-bacterial osteomyelitis

#### Pei Ling Ooi^1^, Gemma Buckley^2^, Lorenzo Biassoni^2^, Marina Easty^2^, Sandrine Compeyrot-Lacassagne^1^

##### ^1^Department of Rheumatology, Great Ormond Street Hospital for Children, London, United Kingdom**;**^2^Department of Radiology, Great Ormond Street Hospital for Children, London, United Kingdom

###### **Correspondence:** Pei Ling Ooi

**Introduction:** Chronic non-bacterial osteomyelitis (CNO) is a rare non-infectious inflammatory bone disorder that affects young children and adolescents. It usually affects multiple sites and most commonly the metaphyseal regions of long bones in the lower limbs, pelvis and clavicles.

There is presently no approved guideline for evaluation of bone inflammation in chronic non-bacterial osteomyelitis (CNO) or chronic recurrent multifocal osteomyelitis (CRMO). Bone scan (BS) has been the conventional imaging modality used to evaluate this rare inflammatory bone disease. While recent literature suggests whole body-magnetic resonance imaging (WB-MRI) as the imaging modality of choice, limited resources and high costs are often prohibitive.

**Objectives:** We aimed to compare the diagnostic yield of bone scan to WB-MRI in CRMO in relation to clinical symptoms and location of lesions on imaging.

**Methods:** A retrospective review of clinical notes of children with CNO/CRMO was performed. 7 patients with paired BS and WB-MRI performed less than 9 weeks apart between September 2009 and October 2015 were analysed. Patients were excluded if both scans were done more than 9 weeks apart or if there was treatment change between scans.

**Results:** Four sets of scans were performed at diagnosis and 3 sets after initiation of treatment. BS and WB-MRI detected a total of 29 and 40 lesions respectively. 6 out of 7 patients had asymptomatic lesions. 17/29 (58.6%) and 27/40 (67.5%) were found on BS and WB-MRI respectively.

Symmetrical lesions were detected in 4/7 patients with no significant difference between both modalities (BS 43% vs MRI 57%). All symmetrical lesions occurred in the pelvis and lower limbs. BS was superior in detecting lesions in the spine (85.7% vs 57.4%) and feet (42.8% vs 14.29%), whereas WB-MRI was better for upper limb (0% vs 14.29%) and lower limb (57.1% vs 71.4%) lesions. Both performed equally in detecting lesions in the pelvis and clavicles, with an incidence of 85.7% and 28.6% respectively.

None of the differences in results reached statistical significance but our cohort of patients was small.

**Conclusion:** Whole body bone scan performs comparably to MRI in detecting inflammatory bone lesions in CRMO, both symptomatic and asymptomatic. Bone scan remains an appropriate and accessible imaging modality in the evaluation of CRMO, particularly in institutions where resources are limited.

**Disclosure of Interest:** None Declared

### P35 The status of bone metabolism in children with juvenile idiopathic arthritis

#### Yury M. Spivakovskiy^1^, Anna Spivakovskaja^1^, Youri Chernenkov^1^, Natalya Zacharova^2^

##### ^1^Department of Hospitality Pediatrics, SARATOV STATE MEDICAL UNIVERSITY, Saratov, Russian Federation; ^2^SARATOV STATE MEDICAL UNIVERSITY, Saratov, Russian Federation

###### **Correspondence:** Yury M. Spivakovskiy

**Introduction:** The decrease in mineral bone density (BMD) is one of the main manifestations of juvenile idiopathic arthritis (JIA).

**Objectives:** To evaluate indicators of bone metabolism in patients with articular JIA.

**Methods:** The study included 66 patients with articular variant of JIA (36 oligoarticular variant of the disease, 30 - with polyarticular), whose average age of 9.74 + 3.56 years, boys – 28, girls - 38. All patients by ELISA in the serum was assessed level: beta crosslaps c-terminal telopeptide (CL), osteoprotegerin (OPG), a membrane protein RANKLand 25-hydroxycholecalciferol (25(Oh)D), carried out ultrasonic densitometry. The obtained data were compared with the results defined in the group of children without signs of inflammatory diseases (comparison group).

**Results:** The results of densitometry showed a reduction in BMD in the range Z = -1,1 -2,6 SD up to 40% of cases with oligoarticular the form of JIA and in 57,7% of cases, polyarticular JIA variant. Deficiency of 25(Oh)D in blood serum of examined children with oligoarthritis and polyarthritis detected at 23.3% and 50%, respectively, and the failure is at 76,7% and 50%, respectively. In the control group, these figures were 10% and 75%, respectively. The ratio of RANKL/OPG in children with oligoarthritis were 0.17 and polyarthritis - 0,22 in the control group - 0,07. Indicators of CL, indicating bone resorption in the group of oligoarthritis was increased at 93.3%, in the group of polyarthritis in 100%.

**Conclusion:** Thus, in patients with JIA is a decrease in BMD, with changes in biochemical parameters outstrip the capabilities of the method of instrumental diagnosis (densitometry) and can be recommended for research on early stages of osteopenia providing for timely correction of bone metabolism.

**Disclosure of Interest:** None Declared

### P36 Tocilizumab (ANTI-IL6R MOAB) achieves complete and sustained remission in a patient with refractory CRMO

Abstract withdrawn

## Disease outcome

### P37 Intraarticular corticosteroid injections (IACI) of temporo-mandibular joints (TMJ) in juvenile idiopathic arthritis (JIA): the patients’ perspective

#### Alessia Arduini^1^, Denise Pires-Marafon^2^, Rebecca Nicolai^2^, Aurora Pucacco^2^, Fabrizio De Benedetti^2^, Silvia Magni-Manzoni^2^

##### ^1^Pediatrics, Policlinico Umberto I, “La Sapienza” University, Rome, Italy ^2^Rheumatology, IRCCS Bambino Gesù Hospital, Rome, Italy

###### **Correspondence:** Alessia Arduini

**Introduction:** Several studies investigated the accuracy and efficacy of IACI for the treatment of TMJ involvement in JIA. However, the impact on patients’ daily activities and patients’ acceptance of TMJ IACI has been scarcely studied.

**Objectives:** To detect variations in daily activities involving TMJs in patients with JIA, before and after TMJ IACI, through a Juvenile Arthritis TMJ self-reported Assessment (JATA) questionnaire, developed for the purpose. Second aim: to assess the patients’ acceptance of the TMJ IACI procedure at the study center.

**Methods:** Patients with JIA and clinical involvement of TMJ consecutively seen at the study center in September-December 2016, with at least 8 years of age, were asked to identify the daily activities most affected by TMJ arthritis. From the collected items, we asked them to select the most relevant and representative of “TMJ well-being/worsening” in their daily life. We included in the JATA the items most frequently selected and a patient’s global assessment (GA) of their TMJs (VAS 0-10; 0 = worst; 10 = best). Then, a subgroup of patients, who underwent to intraarticular corticosteroid injection (IACI) of one or both TMJ, was asked to complete the questionnaire (VAS 0-10; 0 = totally unable, 10 = fully able) and TMJ-GA, before and after the therapeutic intervention. Further, we asked the patients to provide a global assessment of the TMJ IACI procedure performed at the study center (VAS 0-10; 0 = worst; 10 = best). Statistical analysis was performed using GraphPad Prism 5 software. Scores of each item and JATA-score were compared between pre- and post-TMJ IACI (Wilcoxon matched pairs test, statistical significance: p <0.05).

**Results:** Twenty (77%) out of the 26 eligible JIA patients identified daily activities most relevantly affected by TMJ involvement. Thereafter, the items most frequently selected were included in the JATA: 1.ability to bite a sandwich/an apple/a “bombolone”; 2.ability to eat a pizza or a steak; and 3.ability to accurately wash the teeth. Eight patients (100% female, 62.5% with persistent oligoarthritis and 12.5% with extended oligoarthritis, polyarticular RF-negative, and polyarticular RF-positive, respectively) filled the JATA questionnaire, reporting their abilities and global assessment of TMJ before and after TMJ IACI. Each questionnaire was completed in about 3-5 minutes. We observed a statistically significant improvement in the scores, both of the single items and the TMJ-GA, prior and after TMJ IACI (Table 17). The procedure was evaluated with a median score of 8 (IQR: 7.8-10).

**Table 17:** **(Abstract P37).** Comparison between JATA single-item scores and JATA TMJ-GA before and after TMJ intraarticular corticosteroid injection (IACI); VAS 0-10 (0 = worst;10 = best). Values are expressed as median (IQ). Wilcoxon test; statistical significance: p < 0.05.

	Pre-TMJ IACI	Post-TMJ IACI	p
Ability to bite a sandwich/an apple/a “bombolone”	5,0 (4,8-6,0)	9,0 (8,0-10,0)	0,022
Ability to eat a pizza or a steak	5,0 (4,0-7,0)	9,0 (8,5-10,0)	0,036
Ability to accurately wash the teeth	4,0 (4,0-7,3)	10,0 (9,5-10,0)	0,035
TMJ global assessment	5,0 (3,0-5,3)	9,0 (8,5-9,5)	0,022

**Conclusion:** The JATA questionnaire demonstrated to be easily applicable and showed significant improvement of each item, after TMJ IACI. TMJ IACI procedure at the study center was well accepted. Further studies are planned to confirm these results and investigate the applicability of JATA in clinical practice and research.

**Disclosure of Interest:** None Declared

### P38 Early pain report in oligoarticular juvenile idiopathic arthritis is related to long-term outcome

#### Ellen D. Arnstad^1,2^, Veronika G. Rypdal^3,4^, Suvi Peltoniemi^5^, Ellen Nordal^3,4^, Lillemor Berntson^6^, Kristiina Aalto^5^, Anders Fasth^7^, Troels Herlin^8^, Susan Nielsen^9^, Marek Zak^9^, Pål R. Romundstad^10^, Marite Rygg^2,11^ and Nordic Study Group of Pediatric Rheumatology (NoSPeR)

##### ^1^Department of Pediatrics, Levanger Hospital, Levanger, Norway**;**^2^Department of Laboratory Medicine, Children’s and Women’s Health, NTNU - Norwegian University of Science and Technology, Trondheim, Norway**;**^3^Department of Pediatrics, University Hospital of North Norway, Tromsø, Norway**;**^4^Department of Clinical Medicine, UIT The Arctic University of Norway, Tromsø, Norway**;**^5^Department of Pediatrics, Children’s Hospital, Helsinki University Hospital, Helsinki, Finland**;**^6^Department of Women`s and Children`s Health, Uppsala University, Uppsala, Sweden**;**^7^Department of Pediatrics, University of Gothenburg, Gothenburg, Sweden**;**^8^Department of Pediatrics, Århus University Hospital, Århus, Denmark**;**^9^Pediatric Rheumatology Department, Pediatric Clinic II, Copenhagen University Hospital, Rigshospitalet, Copenhagen, Denmark**;**^10^Department of Public Health and General Practice, NTNU - Norwegian University of Science and Technology**;**^11^Department of Pediatrics, St. Olavs Hospital, Trondheim, Norway

###### **Correspondence:** Ellen D. Arnstad

**Introduction:** Persistent course oligoarticular juvenile idiopathic arthritis (JIA) has the best prognosis of all JIA categories. One third of children with oligoarticular onset will, however, develop oligoarticular extended JIA or other JIA categories during the course of the disease with outcome similar to polyarticular disease (1). Therefore it is important to look for early predictive factors to define long-term outcome for these children. Pain is a frequent complaint, and knowledge of pain and its relevance for disease outcome is limited.

**Objectives:** To study if self-reported pain early in disease course can predict long-term disease outcome among children with oligoarticular JIA.

**Methods:** Consecutive cases of JIA from defined areas of Norway, Denmark, Sweden and Finland with onset in 1997 to 2000 were prospectively included in a population-based cohort study (1). Self-reported pain was measured on a 10 cm visual analogue scale (VAS_pain_). The Finnish participants were excluded due to lack of pain scores. Remission at 8 years follow-up was defined according to Wallace (2). We used binomial regression in STATA for multivariable analyses.

**Results:** The final study population consisted of 243 children, and of these 120 (49%) had oligoarticular disease 6 months after disease onset. Median age at onset in the oligoarticular group was 5.0 years, 73% were girls and 67% reported VAS_pain_ >0 six months after disease onset. At 8 years follow-up 43% had developed oligoarticular extended or other JIA categories, 49% reported VAS_pain_ >0 and 53% were not in remission off medication.

In the oligoarticular onset group, we analyzed associations between early pain reports and outcome at 8 years follow-up. Children reporting pain 6 months after disease onset developed oligoarticular extended or other JIA categories more often compared to those reporting no pain at 6 months (percent point difference 19%, 95% CI [1-37%]). Among those reporting pain at 6 months, a higher proportion reported pain also at 8 years, compared to children with no pain at 6 months (percent point difference 35%, 95% CI [16-53%]. Pain at 6 months was associated with not being in remission off medication at 8 years compared to no pain at 6 months (percent point difference 36%, 95% CI [19-54%]. After adjustments for age and sex, the results were similar.

**Conclusion:** Early pain report among children with oligoarticular JIA seems to predict development into prognostically more unfavourable JIA categories, more pain persistence and more severe long-term disease outcome.

References:

1. Nordal E, et al. Arthritis Rheum 2011;63:2809-18

2. Wallace CA, et al. J Rheumatol 2004;31:2290-4

Trial registration identifying number:

**Disclosure of Interest:** None Declared

### P39 HLA II class alleles in juvenile idiopathic arthritis patients with and without chronic arthritis signs in temporomandibular joints evaluated with contrast enhanced MRI

#### Zane Davidsone^1,2^, Elena Eglite^3^, Aleksandrs Kolesovs^4^, Ruta Santere^1^, Valda Stanevica^1^

##### ^1^Rheumatology, Childrens university hospital, Riga, Latvia; ^2^Pediatric, Riga`s Stradins University, Riga, Latvia; ^3^Laboratory of clinical immunology and immunogenetics, Riga Stradins University, Riga, Latvia; ^4^University of Latvia, Riga, Latvia

###### **Correspondence:** Zane Davidsone

**Introduction:** Temporomandibular joint (TMJ) involvement with both active and chronic signs of arthritis is seen very often (17-87%) in children with juvenile idiopathic arthritis. Contrast enhanced MRI is the golden standart for diagnosis of TMJ arthritis. There are data that specific HLA II class alleles can be asociated with more severe disease course and radiologic progression in patients with rheumatoid arthritis.

**Objectives:** To identify HLA II class alleles of risk and protection in JIA patients with chronic arthritis signs in temporomandibular joints evaluated with contrast enhanced MRI.

**Methods:** We performed retrospective study with 72 JIA patients treated at Childrens university hospital in whom TMJ MRI was performed and acute or chronic or both signs of arthritis were detected. Patients were genotyped for HLA- DRB1; DQB1; DQA1- using RT-PCR with sequence-specific primers. Associations of DRB1; DQB1; DQA1 alleles in patients were examined individually using the Chi-square test and Cochran-Mantel-Haenszel test.

**Results:** 72 JIA patients with mean age of 13.9 years (range 6.0-17.9 yr); 48 (77%) girls and 14 (23%) boys. The mean duration of the disease from the time of diagnosis till performing TMJ MRI was 1.5 years (range 0.2-8.0 yr). JIA subtypes were as follows: seronegative polyarthritis 60%, seropositive polyarthritis 8%, peristant olygoarthritis 2%, arthritis with enthesitis 9%, undifferentiated 3% and 2%for systemic arthritis.

Two groups were separated on the basis of presence of chronic arthritis signs (n = 62) or absence of them (n = 10). Alleles DRB1*14 (OR = 0.19, 95% CI from 0.04 to 0.92, p = 0.039) and DQA1*05:01 (OR = 0.29, 95% CI from 0.11 to 0.80, p = 0.016) were less often observed in the group with chronic arthritis signs than in the group without them. There were no longer duration of disease in the group with chronic arthritis signs (1,47 ± 1,98) compared with 2nd group (2,71 ± 3,30) (p = 0,173).

**Conclusion:** Alleles DRB1*14 and DQA1*05:01 are possibly protective for development of structural damage in temporomandibular joints in JIA patients and is not dependent on duration of the disease.

**Disclosure of Interest:** None Declared

### P40 The role of antinuclear antibodies positivity in predicting clinical remission in juvenile idiopathic arthritis

#### Raquel M. Ferreira, Francisca Aguiar, Mariana Rodrigues, Iva Brito

##### São João Hospital Centre, Porto, Portugal

###### **Correspondence:** Raquel M. Ferreira

**Introduction:** Juvenile idiopathic arthritis (JIA) consists of an auto-immune condition with several subgroups with distint clinical, laboratory features and therapy response. Although antinuclear antibody (ANA) positivity can be found in all subtypes, a higher prevalence in the oligoarticular form is well known. Recently, its association with the remission rate has been studied, but few data are available.

**Objectives:** To evaluate the clinical response based on ANA status in JIA patients.

**Methods:** We performed an observational retrospective study with all children diagnosed with JIA followed in our Rheumatology Pediatric Unit until april 2017. Demographic and clinical data were colleted from medical records. JIA subtypes were categorized according to the classification of the International League of Associations for Rheumatology 2001. ANA were considered positive when at least 2 titers were ≥ 1/100. Remission status at follow-up was defined using the Wallace preliminary criteria. Parametric and non-parametric test were used for statistic analysis (SPSS 20.0). P-values <0.05 were considered statistically significant.

**Results:** A total of ninety-four patients were enrolled. Sixty-three were female (67%). The mean age at time of diagnosis was 7.2 years (SD 4.4) and the mean follow-up was 8.9 years (SD 6.6). The most common subgroup was oligoarticular persistent JIA (38.3%), followed by polyarticular (25.5%), systemic (13.8%), psoriatic (10.6%), enthesitis-related arthritis (7.4%) and oligoarticular extended JIA (4.3%). Extra-articular manifestation as uveitis was seen in 14 patients. Both rheumatoid factor and anti-citrullinated peptide antibody were positive in 37.5% of the polyarticular JIA. ANA positivity was presented in 41.5% of all patients, in which was found a significant predominance of the oligoarticular form, female gender, earlier onset of disease and increased risk of developing chronic anterior uveitis during the course of the disease (p = 0.001, p = 0.025, p = 0.001, p = 0.015, respectively). The mean joint count at diagnosis was 3.9 (SD 5.0). At the last follow-up evaluation the mean joint count was 0.32 (SD 0.7), almost 59% of the patients were in remission (25.5% on medication for at least 6 months and 33% off medication for ≥ 12 months) and 41.5% had active disease. 6.4% of the patients were treated with biologic monotherapy, 37.2% were receiving classic DMARDs while 14.9% were under combination therapy. In the group of patients in remission on medication >6 months, 8.3% and 45.8% of the patients were treated with biologic and classic DMARDs respectively, and combination therapy was observed in 37.5% of them. In the group with active disease, biologic monotherapy, classic DMARDs or combination therapy use was seen in 10.3%, 61.5% and 12.8% of the patients, respectively. No significant diferences were found in what concerns clinical outcome when comparing ANA-positive and ANA-negative patients (p = 0.091).

**Conclusion:** Our study demonstrates once again the association between the ANA positivity and the oligoarticular subtype, female predominance and the presence of uveitis. It also supports the late literature that didn’t find a relationship between the remission rate and ANA status.

**Disclosure of Interest:** None Declared

### P41 Evaluation of knee joint Q angle and balance in juvenile idiopathic arthritis patients with pesplanovalgus deformity

#### Eylul Pinar Kisa^1^, Serap Inal^2^, Ela Tarakci^3^, Nilay Arman^3^, Ozgur Kasapcopur^4^

##### ^1^Division of Physiotherapy and Rehabilitation, Faculty of Health Science, Yeditepe University, Istanbul, Turkey; ^2^Division of Physiotherapy and Rehabilitation, Faculty of Health Science, Bahcesehir University, Istanbul, Turkey; ^3^Division of Physiotherapy and Rehabilitation, Faculty of Health Science, Istanbul, Turkey ^4^Department of Pediatric Rheumatology, Medical Faculty of Cerrahpasa, Istanbul University, Istanbul, Turkey

###### **Correspondence:** Eylul Pinar Kisa

**Introduction:** Juvenile idiopathic arthritis (JIA) is the childhood disability from a musculoskeletal disorder. It generally affects large joints such as the knee and the ankle. JIA combine with muscle weakness, poor flexibility, atrophy, pain, and decreased proprioception of the affected joints, abnormal biomechanics, articular cartilage damage.

**Objectives:** We aimed to observe the effects of knee Q deformity on postural stability of children with pes planovalgus having JIA.

**Methods:** Twenty participants as age range 4-16 years (4 female,16 male) were enrolled this study. New York Posture Rating Scale were used to evaluate the posture of participants. Hand held dynamometry was used to assess the strength of the lower extremity muscles. Their static balance evaluated with Flamingo balance test. Their dynamic balance evaluated with Prokin balance system. Q angle was calculated by Universal desktop ruler. Universal Desktop Ruler allows you to measure not only a straight-line distance but any curved distance on the screen. For statistical analysis SPSS Version 21.0 program was used.

**Results:** For our statistical evaluations, abduction, adduction, knee flexion and extension negatively, knee flexion angle and addiction angle positively may affect static balance(p < 0,05). In addition to that, dynamic balance related to power of plantar flexion positively, knee extension, inversion, abduction angle positively. The results of this study showed that knee flexion angle and plantar flexion angle may affect Q angle negatively. There is statistically significant effect between Q angle and posture deformity (p < 0.05).

**Conclusion:** In this study, power of hip flexion structure and function of the foot and ankle, and there is preliminary evidence that foot problems impair balance. Balance has often been used as a measure of lower extremity function and is defined as the process of maintaining the center of gravity within the body's base of support. These results are supported by evaluating more children with juvenile idiopathic arthritis with pesplanovalgus.

**Disclosure of Interest:** None Declared

### P42 Development of a unique platform for pediatric immuno-rheumatologic diseases (JIRCOHORTE): inclusion of 3266 patients.

#### M. Mejbri^1^, R. Carlomagno^1^, F. Hofer^1^, V. Hentgen^2^, B. Bader-Meunier^3^, B. Fonjallaz^4^, S. Georgin- Lavialle^5^, Y. Guex-Crosier^6^, P. Scolozzi^7^, A. Woerner^8^, E. Cannizzaro^9^, D. Kaiser^10^, G. Berthet^11^, W. Baer^12^, F. Vanoni^1^, K Theodoropoulou^1^, A. Belot^13^, M. Hofer^1^

##### ^1^Consultation Romande d’immuno-rhumatologie pediatrique CHUV, HUG, Lausanne, Geneva, Switzerland, ^2^Centre Hospitalier de Versailles, Versailles, France ^3^Hôpital Universitaire Necker, Paris, France, ^4^Ligue Genevoise contre le Rhumatisme, Geneva, Switzerland, ^5^Hôpital Tenon, Paris, France, ^6^Hôpital Ophtalmique Jules-Gonin, Lausanne, Switzerland ^7^HUG, Geneva, Switzerland ^8^Kinderspital Beider, Basel, Switzerland ^9^Kinderspital, Zürich, Switzerland ^10^Kinderklinik, LUCERNE, Switzerland ^11^Kinderklinik, Aarau, Switzerland ^12^Kantonspital, Graubunden, Switzerland, ^13^Hôpital Femme Mère Enfant, Lyon, France

###### **Correspondence:** M. Mejbri

**Introduction:** Pediatric immune-rheumatologic diseases are rare, characterized by chronic course and significant impact on patient’s life. Recent developments have significantly improved the prognosis of these diseases, but a close follow-up of patients’ cohorts is essential to evaluate the long- term outcome. The JIRcohorte is an international platform developed to follow pediatric immune- rheumatologic diseases, and evaluate the long-term tolerance and efficacy of immunosuppressive and biological therapies. The challenge was to develop a tool with items both common for all patients and specific for each disease.

**Objectives:** Describe the multi-module tool implemented in the JIRcohorte platform and the collective of patients included in the different modules.

**Methods:** For each of the eCRF, an expert group has defined the items to be collected for prospective follow-up of patients with a specific disease. A first comparison was done to highlight the identical items from the different eCRF and the items specific to each one. For all the items reported in more than one module in a similar but not identical way, a negotiation between the experts made it possible either to find a common item or to clearly define the difference between them. We describe the patients of 45 centers (in Belgium, France, Maroc and Switzerland) included in the JIRcohorte between 2014 and 2017.

**Results:** Thanks to the development of a multi-module tool, we were able to reduce the number of items to insert in the JIRcohorte from 3860 to 2188, by keeping the same level of information. A total of 3266 patients and 10733 visits were collected. The number of patients and visits per module are as follows: Juvenile Idiopathic Arthritis (1371 patients, 4782 visits), Temporomandibular Arthritis (64, 156), Juvenile Dermatomyositis (18, 61), Juvenile Systemic Lupus (121, 369), Juvenile Periodic Fever Syndrome (450, 803), Still Disease (54, 176), Uveitis (142, 1409) and Vaccination (2251, 6924).

**Conclusion:** JIRcohorte collects follow-up data on pediatric patients with different immuno- rheumatologic pathologies. Thanks to its structure, with both common and specific items in each module, it can be used as a valuable tool to compare pediatric patients with different inflammatory rheumatic diseases.

**Disclosure of Interest:** None Declared

## Genetics and environment

### P43 Progressive pseudorheumatoid dysplasia resolved by whole exome sequencing: a novel mutation in WISP3 and review of the literature

#### Aviva Eliyahu^1,2^, ben pode-shakked^3,4,5^, Asaf Vivante^5,6^, Ortal Barel^7^, Shai Padeh^8,9^, Dina Marel-Yagel^9,10^, Alvit Veber^11^, Shachar Abudi^12^, Irit Tirosh^2,13^, Shiri Shpilman^2,14^, Shirlee Shril^15^, gideon rechavi^7,16,17^, Friedhelm Hildebrandt^18^, Mordechai Shohat^7,19^, yair anikster^3,20,21^

##### ^1^ Metabolic Disease Unit, Edmond and Lily Safra Children's Hospital, Sheba Medical Center, tel hashomer, Israel; ^2^Sackler Faculty of Medicine, Tel-Aviv University, Tel Aviv, Israel; ^3^Metabolic Disease Unit, Edmond and Lily Safra Children's Hospital, Sheba Medical Center, Tel Hashomer, Israel; ^4^Sackler Faculty of Medicine, Tel-Aviv University, Tel Aviv, Israel; ^5^The Dr. Pinchas Borenstein Talpiot Medical Leadership Program, Sheba Medical Center, Tel-Hashomer, Israel; ^6^Division of Nephrology, Department of Medicine, Boston Children's Hospital, Harvard Medical School, Boston, MA, United States; ^7^Sheba Cancer Research Center, Sheba Medical Center, Tel Hashomer, Israel; ^8^Pediatric Rheumatology Unit and Department of Pediatrics, Edmond and Lily Safra Children's Hospital, Sheba Medical Center, Tel-Hashomer, Israel; ^9^Sackler Faculty of Medicine, Tel-Aviv University, Tel-Aviv, Israel; ^10^Metabolic Disease Unit Israel, Edmond and Lily Safra Children's Hospital, Sheba Medical Center, Tel-Hashomer, Israel; ^11^Metabolic Disease Unit, Edmond and Lily Safra Children's Hospital, Sheba Medical Center, Tel-Hashomer, Israel; ^12^Metabolic Disease Unit, Edmond and Lily Safra Children's Hospital, Sheba Medical Center, Tel-Hashomer, Israel; ^13^Pediatric Rheumatology Unit and Department of Pediatrics, Edmond and Lily Safra Children's Hospital, Sheba Medical Center, Tel Hashomer, Israel; ^14^Pediatric Rheumatology Unit and Department of Pediatrics, Edmond and Lily Safra Children's Hospital, Tel Hashomer, Israel; ^15^Division of Nephrology, Department of Medicine, Boston Children's Hospital, Harvard Medical School, Boston, MA, United States; ^16^Sackler Faculty of Medicine, Tel Aviv University, Tel Aviv, Israel; ^17^The Wohl Institute for Translational Medicine, Sheba Medical Center, Tel Hashomer, Israel; ^18^ Division of Nephrology, Department of Medicine, Boston Children's Hospital, Harvard Medical School, Boston MA, United States; ^19^Sackler Faculty of Medicine, Tel-Aviv University, Tel-Aviv, Israel; ^20^Sackler Faculty of Medicine, Tel-Aviv University, Tel-Aviv, Israel; ^21^The Wohl Institute for Translational Medicine, Sheba Medical Center, Tel-Hashomer, Israel

###### **Correspondence:** Aviva Eliyahu

**Introduction:** Progressive pseudorheumatoid dysplasia (PPRD) is a rare autosomal-recessive, non-inflammatory arthropathy, shown to be caused by mutations in the WNT1-inducible signaling pathway protein 3 (*WISP3*) gene. Although several hundred cases were reported worldwide, the diagnosis remains challenging. Subsequently, the syndrome is often unrecognized and misdiagnosed, leading to unnecessary procedures and treatments.

**Objectives:** We present here a multiply affected consanguineous family of Iraqi-Jewish descent with PPRD. The proband, a 6.5 years old girl, presented with bilateral symmetric bony enlargements of the 1^st^ interphalangeal joints of the hands, without signs of synovitis.

**Methods:** Molecular analysis by Whole Exome Sequencing and homozygosity mapping was performed on the proband and other affected and non affected relatives.

**Results:** Molecular analysis by Whole Exome Sequencing and homozygosity mapping identified a novel homozygous missense mutation (c.257G > T, p.C86F) in the *WISP3* gene. Following this diagnosis, an additional 53 years old affected family member was found to harbor the mutation. Two other individuals in the family were reported to have had similar involvement however both had died of unrelated causes.

**Conclusion:** The reported family underscores the importance of recognition of this unique skeletal dysplasia by clinicians, and especially by pediatric rheumatologists and orthopedic surgeons.

**Disclosure of Interest:** None Declared

### P44 Cytokines genes and the severity of juvenile idiopathic arthritis

#### Liliia S. Nazarova^1^, Kseniia V. Danilko^1^, Tatiana V. Viktorova^1,2^, Viktor A. Malievsky^1^

##### ^1^Bashkir State Medical University, Ufa, Russian Federation; ^2^Institute of Biochemistry and Genetics, Ufa, Russian Federation

###### **Correspondence:** Viktor A. Malievsky

**Introduction:** Juvenile idiopathic arthritis (JIA) is the most common chronic rheumatic disease in children and an important cause of short-term and long-term disability [1]. It is essential to know the prognosis for the individual patient early in the course of disease and preferentially at the time of diagnosis in order to start the right treatment immediately [2].

**Objectives:** The goal of the study was to determine whether the *TNFA* gene -308A > G, *IL1B* gene -511C > T and *IL6* gene -174G > C single nucleotide polymorphisms (SNPs) are associated with the disease severity in patients with JIA.

**Methods:** The study included 255 patients with JIA who were divided into 4 subgroups according to the severity of the disease course: mild (N = 33), moderate (N = 58), severe (N = 68) and very severe (N = 96). The grouping was performed depending on the number of affected joints, the presence or absence of high laboratory activity, systemic features, uveitis, early radiologic progression, functional disability and the need for early "aggressive" therapy. Genotyping was performed using real-time PCR method. Statistical analysis was performed using two-tailed Fisher's exact test (p), odds ratio (OR) and 95% confidence interval (CI).

**Results:** The *IL6* -174G allele was significantly more frequent in patients with a very severe disease course, than in other patients (p = 0.040, OR = 1.498, 95%CI 1.033-2.167). At the same time, there was a significantly higher proportion of the *IL1B* -511CT genotype in a mild disease course subgroup in comparison to other subgroups (p = 0.024, OR = 2.427, 95%CI 1.109-5.160). Then the analysis was performed separately for girls and boys. The association mentioned above for the *IL6* -174G allele was confirmed only for boys (p = 0.026, OR = 2.108, 95%CI 1.131-3.883) and for the *IL1B* -511CT genotype – only for girls (p = 0.0004, OR = 10.079, 95% CI 2.656-44.857). In addition, it was found that the *IL1B* -511 T allele marks a very severe JIA course in boys (p = 0.019, OR = 2.231, 95% CI 1.135-4.215) and that the *TNFA* -308A allele marks a mild JIA course in boys (p = 0.007, OR = 4.778, 95% CI 1.731-14.256).

**Conclusion:** In this study we revealed gender-specific associations of the *IL6* gene -174G > C, *IL1B* gene -511C > T and *TNFA* gene -308A > G SNPs with an altered risk of a very severe or mild JIA course.

**References.** [1] Ravelli A, Martini A. Juvenile idiopathic arthritis. Lancet. 2007 Mar 3;369(9563):767-78.

[2] van Dijkhuizen EH, Wulffraat NM. Early predictors of prognosis in juvenile idiopathic arthritis: a systematic literature review. Ann Rheum Dis. 2015 Nov;74(11):1996-2005.

**Disclosure of Interest:** None Declared

### P45 Single nucleotide polymorphisms in survivin gene are associated to response to methotrexate in juvenile idiopathic arthritis

#### Mojca Zajc Avramovič^1^, Vita Dolzan^2^, Natasa Toplak^1^, Tadej Avcin^1^

##### ^1^Department of Allergology, Rheumatology and Clinical Immunology, Children’s Hospital, University Medical Centre Ljubljana, Slovenia, Ljubljana, Slovenia ^2^Institute of Biochemistry, Medical faculty, Ljubljana, Slovenia

###### **Correspondence:** Mojca Zajc Avramovič

**Introduction:** Survivin is an anti-apoptotic protein and its circulating levels were associated with joint destruction and erosions in rheumatoid arthritis (RA). Recently it was suggested to be a marker of severe disease course in adult patients with RA as well as juvenile idiopathic arthritis (JIA). Three single nucleotide polymorphisms (SNPs) can affect the normal protein and its concentration. Methotrexate (MTX) is an important drug in treating JIA, but markers to predict its efficacy are needed.

**Objectives:** To evaluate the effect of SNPs in survivin gene on methotrexate efficacy in JIA.

**Methods:** The data of 119 consecutive patients with JIA treated with MTX at the University Children’s Hospital Ljubljana from June 2011 to January 2015 have been retrospectively reviewed. The disease activity was measured using JADAS 71 score. Non-responders were defined as patients not reaching 30% improvement in JADAS 71 score 6 months after the beginning of treatment with MTX. Genotyping of SNPs in the genes for survivin was performed using real time PCR methods. The following SNPs were analyzed: BIRC5 G692C rs8073069, BIRC5 T241C rs17878465 and BIRC5 G692C rs8073069. Two-tailed Fisher exact test was used for statistical analysis.

**Results:** A total of 119 patients were included in the analysis, 91 (76.5%) girls and 28 (23.5%) boys. Ten (8.4%) patients had systemic arthritis, 41 (34.5%) RF negative polyarthritis, 5 (4.2%) RF positive polyarthritis, 24 (20.2%) persistent oligoarthritis, 24 (20.2%) extended oligoarthritis, 11 (9.2%) juvenile psoriatic arthritis, 2 (1.7%) patients suffered from enthesitis related arthritis and 2 (1.7%) had undifferentiated arthritis. Mean time of disease duration before MTX was started was 13 months in the whole cohort and 6.9 months in the subgroup of patients with polyarticular disease (RF positive and RF negative). Mean starting dose of MTX was 10.3 mg/m^2^ and mean dose at 6 months was 12.0 mg/m^2^. Forty-two (35%) patients were switched to subcutaneous MTX to achieve higher efficacy. At 6 months 30/116 (25.8%) of patients were defined as non-responders. BIRC5 rs17878465 (p < 0.0001) and BIRC5 rs9904341 (p = 0.0272) were associated with achieving 30% improvement in JADAS 71 after 6 months. BIRC5 rs9904341 was also associated with 70% improvement in JADAS 71 score (p < 0.0001) after 6 months of treatment.

**Conclusion:** Our results suggest that SNPs in survivin gene, BIRC5 rs17878465 and BIRC rs9904341 could be markers of MTX response in JIA.

**Disclosure of Interest: **None Declared

## Imaging - Vasculitides

### P46 Henoch schonlein purpura nephitis: initial risk factor and outcomes in a tertiary center of Latin America

#### Izabel M. Buscatti, Beatriz B. Casella, Nadia E. Aikawa, Andrea Watanabe, Sylvia C. Fahrat, Lucia M. Campos, Clovis A. Silva

##### Pediatric Rheumatology Division, CHILDREN’S INSTITUTE, HOSPITAL DAS CLINICAS HCFMUSP, FACULDADE DE MEDICINA, UNIVERSIDADE DE SAO PAULO, São Paulo, Brazil

###### **Correspondence:** Izabel M. Buscatti

**Introduction:** European League Against Rheumatism/Paediatric Rheumatology International Trials Organisation/Paediatric Rheumatology European Society (EULAR/PRINTO/PRES) proposed validated classification criteria for Henoch-Schönlein purpura (HSP). However, there are rare studies reporting initial risk factors to HSP nephritis (HSPN) and outcomes using these new criteria. In addition, these studies generally included small samples, and none of them were evaluated in Latin American population.

**Objectives:** To evaluate risk factors associated with HSPN and outcomes in children and adolescents of a tertiary center in Latin America.

**Methods:** Two hundred ninety six patients with HSP were retrospectively assessed by demographic data, and initial clinical manifestations, laboratory exams and treatments evaluating in the first three months of disease. All of them fulfilled validated EULAR/PRINTO/PRES criteria for HSP. They were also divided in two groups: with and without nephritis. Additional, persistent non-nephrotic proteinuria, nephrotic proteinuria and renal insufficiency were also evaluated at 1, 5, 10 and 15 years after diagnosis.

**Results:** Nephritis was detected in 139/296 (47%) at first 3 months. The median of age at diagnosis was significantly higher in HSPN patients compared to those without this complication [6.6(1.5-17.7) vs. 5.7(0.9-13.5) years, p = 0.022]. The frequencies of persistent purpura (31% vs. 10%, p < 0.0001), recurrent abdominal pain (16% vs. 7%, p = 0.011), gastrointestinal bleeding (25% vs. 10%, p < 0.0001) and corticosteroid use (54% vs. 41%, p = 0.023) were significantly higher in the former group. In the multivariate analysis, logistic regression demonstrated that the independent variables that predicted HSNP were persistent purpura (OR = 3.601; 95%CI 1.605-8.079; p = 0.002) and gastrointestinal bleeding (OR = 2.991; 95%CI 1.245-7.183; p = 0.014). Of 139 HSPN, further analysis revealed that persistent non-nephrotic proteinuria, nephrotic proteinuria and renal insufficiency occurred during follow-up: at 1 year [46/88 (52%), 1/88 (1%) and 2/88 (2%)], at 5 year [25/47 (53%), 1/47 (2%) and 1/47 (2%)], at 10 year [9/20 (45%), 1/20 (5%) and 1/20 (5%)] and at 15 years [1/6 (17%), 0/6 (0%) and 0/6 (0%)]. In addition, in patients without HSPN at disease onset: 29/118 (25%) had persistent non-nephrotic proteinuria and/or hematuria only at 1 year of follow-up, 19/61 (31%) at 5 years, 6/17 (35%) at 10 years and 4/6 (67%) at 15 years of follow-up.

**Conclusion:** Persistent purpura and gastrointestinal bleeding were initial predictors for HSPN. Persistent non-nephrotic proteinuria and/or hematuria may occur during the disease follow-up, even in patients without previous HSPN, and rigorous monitoring of renal involvement should be performed.

**Disclosure of Interest**: I. Buscatti: None Declared, B. Casella: None Declared, N. Aikawa: None Declared, A. Watanabe: None Declared, S. Fahrat: None Declared, L. Campos: None Declared, C. Silva Grant/Research Support from: Conselho Nacional de Desenvolvimento Científico e Tecnológico (CNPq 472155/2012-1)

### P47 Toward the development of a new radiographic score for diagnosis and monitoring of temporomandibular joint disease in children with juvenile idiopathic arthritis

#### Gabriella Giancane, Giacomo Chiappe, Fiammetta Sertorio, Veronica Incarbone, Alessandro Consolaro, Nicola Laffi, Gian Michele Magnano, Angelo Ravelli

##### Istituto Giannina Gaslini, Genoa, Italy

###### **Correspondence:** Gabriella Giancane

**Introduction:** Arthritis of the temporo-mandibular joint (TMJ) is often responsible for severe osteo-articular damage in patients with juvenile idiopathic arthritis (JIA). Early diagnosis of TMJ involvement remains difficult, due to the lack of reliable clinical or imaging parameters. However, magnetic resonance imaging (MRI) is regarded as the most sensitive tool for the detection of TMJ involvement in JIA.

**Objectives:** To develop and validate a MRI score for early detection of TMJ disease activity and damage in children with JIA.

**Methods:** After a review of the most recent literature on MRI of the TMJ in JIA and based on their experience, three specialists in different fields (a rheumatologist, a dentist and a radiologist) devised the score for the assessment of activity and damage of TMJ shown in Table 18. The score consists of 3 parameters of arthritis activity (joint effusion, contrast enhancement (CE) and bone edema), and 4 parameters of joint damage (erosion/irregularities of the mandibular condyle, disc abnormalities, flattening of the condyle, mandibular asymmetry). The activity score ranges from 0 to 6 and the damage score from 0 to 9. After a series of training sessions aimed to reach consensus on the definition of the score features, each specialist assigned independently the MRI score to an unselected sample of TMJ MRIs of JIA patients followed at our center. The inter- and intra-observer reliability were calculated through the weighted kappa. The CE parameter was also compared with a recently validated score calculated on a specific region of interest (ROI) of the TMJ MRI in JIA patients^1^. Agreement was considered acceptable for a k value > 0.60.

**Table 18:** **(Abstract P47).** MRI score (CE: contrast enhancement)

TMJ ACTIVITY PARAMETERS			
	Right	Left	Total score
Joint effusion	YES □ NO □	YES □ NO □	0-2
Contrast enhancement (CE)	YES □ NO □	YES □ NO □	0-2
Bone edema/bone marrow CE	YES □ NO □	YES □ NO □	0-2
Total activity score	0-3	0-3	0-6
TMJ DAMAGE PARAMETERS			
Erosions/irregularities of the condyle	YES □ NO □	YES □ NO □	0-2
Disc abnormalities	YES □ NO □	YES □ NO □	0-2
Flattening of condyle	MODERATE/SEVERE □ MILD□ NO□	MODERATE/SEVERE □ MILD□ NO□	0-4
Mandibular asimmetry	YES □ NO □ IF YES: RIGHT < □ LEFT < □		0-1
Total damage score	0-5		0-9

**Results:** A total of 35 TMJ MRIs performed between December 2013 and December 2016 were evaluated. The analysis of the absolute agreement in score assignment among specialists revealed substantial agreement, with greater concordance between the dentist and the radiologist. The rheumatologist tended to assign lower scores. Among disease activity parameters, the lowest agreement was seen for bone edema, whereas the most challenging joint damage parameter was disc abnormalities. There was a moderate agreement between the CE recorded by the study assessors and that obtained through the ROI calculation, especially for the rheumatologist and the dentist.

**Conclusion:** We have developed a new simple and feasible MRI score for the detection and quantification of disease activity and damage of the TMJ in children with JIA. Although the overall score proved reliable across different specialists, further work is needed to increase concordance for assessment of bone edema and disc abnormalities.

**Reference**: 1. Resnick CM et al. Quantifying Temporomandibular Joint Synovitis in Children With Juvenile Idiopathic Arthritis. Arthritis Care Res (Hoboken). 2016;68:1795-1802.

**Disclosure of Interest:** None Declared

### P48 Determining disease activity in CIA by optical imaging of phagocyte migration

#### Sandra Gran^1^, Lisa Honold^2^, Olesja Fehler^1^, Stefanie Zenker^1^, Sven Hermann^2^, Michael Schäfers^2^, Thomas Vogl^1^, Johannes Roth^1^

##### ^1^Institute of Immunology, Münster, Germany; ^2^European Institute for Molecular Imaging, Münster, Germany

###### **Correspondence:** Sandra Gran

**Introduction:** Phagocyte recruitment and migration to the site of inflammation are key events in the early phase of inflammation. They are indispensable for pathogen elimination, tissue repair and restoration of tissue homeostasis. However, dysregulated phagocyte infiltration and a subsequently overwhelming immune response can also cause severe inflammatory disorders. Therefore, targeting and modulation of phagocyte infiltration represents a promising new approach to fight inflammatory disorders and diseases, such as rheumatoid arthritis. Furthermore, non-invasive tracking of phagocyte migration to the site of inflammation could extend both scientific knowledge as well as the repertoire of diagnostic strategies in clinical use.

**Objectives:** Within this study we aimed to establish a Fluorescence reflectance imaging (FRI) based system to visualize and analyze migration properties of different cell populations in inflammatory disease models, like experimental arthritis, *in vivo*.

**Methods:** Immortalized murine myeloid progenitor ER-HoxB8 cells were differentiated to neutrophils or monocytes (Wang et al., 2006). Differentiated cells were labeled *in vitro* with the membrane-selective fluorescent dyes DIR (Eisenblätter et al., 2009) or DID, respectively. Viability and functionality of labeled cells were confirmed by *in vitro* assays. In several mouse models - particularly in a collagen induced arthritis (CIA) mouse model - we investigated the ability and specific properties of different cell populations to migrate to sites of inflammation *in vivo* via fluorescence reflectance imaging (FRI). Using CRISPR-Cas9 technology we introduced targeted gene deletions for main adhesion molecules.

**Results:** ER-HoxB8 monocytes and neutrophils could effectively be labeled with DIR or DID. *In vitro* assays confirmed that viability and functionality of ER-HoxB8 cells was not affected by cell labeling. Subsequent *in vivo* imaging experiments allowed the visualization of migrated labeled phagocytes in different murine disease models, thereby cells could be detected at sites of inflammation with high sensitivity and specificity. In a CIA mouse model the amount of immigrated cells could even be associated closely to disease score and disease severity. Thus, the detection of immigration of labeled cells might also give hints about new inflammatory spots that are about to settle up before they can be detected macroscopically. Furthermore, differential cell labeling allowed direct quantitative comparison of differences in migration rates of wildtype and CD18 or CD49d knockout cells *in vivo*.

**Conclusion:** Specific and distinguishable labeling of diverse cell types allows *in vivo* tracking and subsequent quantification of migrated cells within the same animal. Targeted gene deletion allows analysis of molecular mechanisms relevant for leukocyte recruitment during different stages of arthritis. Correlation of the amount of immigrated cells to disease severity offers new opportunities to non-invasively detect and monitor inflammatory sites *in vivo*.

**Disclosure of Interest:** None Declared

### P49 Juvenile Sjögrens syndrome (JSS): comparing glandular ultrasound in primary and secondary JSS

#### Johannes-Peter Haas, Manuela Krumrey-Langkammerer

##### German Center for pediatric and adolescent rheumatology, Garmisch-Partenkirchen, Germany

###### **Correspondence:** Manuela Krumrey-Langkammerer

**Introduction:** Diagnosis of juvenile Sjögrens syndrome (jSS) although rare seems to be underestimated in pediatric patients. Especially in patients with undifferenciated mixed connective tissue disease (MCTD) secondary jSS is frequently present but missed in the diagnostic work-up.

**Objectives:** Our intend was to characterize distinct, ultrasonographic (US) findings in a cohort of primary- and secondary jSS patients, the latter with a focus on MCTD and attribute these findings to US scores defined in adult SS.

**Methods:** A single-center study collected data from clinical charts of jSS-patients admitted to the GCPAR. According the EULAR/ACR criteria 8 patients with primary jSS, 8 patients with secondary jSS and MCTD and 9 patients with secondary jSS and other collagenoses were included. All ultrasonographic (US) findings were performed by two experienced investigators between 5/2014 and 3/2017 using a GE logic 8 system. Different scoring systems from the literature were compared for accuracy for gland echostructural abnormalities.

**Results:** A total of 25 patients (22 females, 3 males) with a mean age of 15,8 years have been included. All 9 pjSS patients had sicca symptoms, this was present in only 56,3% of sjSS. Swelling or pain in major salivary glands occurred in 7 of 9 pjSS but only 5 of 16 sjSS (including MCTD). US scoring of salivary glands according to Hocevar [1] which had been proven to be accurate in jSS as well [2] showed a mean score of 26 in pjSS and 18,28 in sjSS patients. We could not correlate US score (Hocevar) with the duration of the disease (Pearsons r =0.197) (Table 19).

**Table 19:** **(Abstract P49).** Salivary gland US-scores according Hocevar´s classification

	pjSS (n = 9)	sjSS other (n = 8)	sjSS in MCTD (n = 8)	All (n = 25)
Decrease in echogenity	3 (33,3%)	3 (37,5%)	2 (25%)	18 (72%)
Inhomogenous parenchyma	9 (100%)	7 (87,5%)	6 (75%)	22 (88%)
Hypoechoic areas	9 (100%)	8 (100%)	7 (87,5%)	24 (96%)
Hyperechoic reflexes	7 (77,7%)	6 (75%)	6 (75%)	19 (76%)
Disturbed border	4 (44,4%)	2 (25%)	0	6 (24%)

**Conclusion:** In patients with pjSS and sjSS salivary gland ultrasound is a helpful, first-line tool not only to detect salivary gland involvement but to score the severity of inflammation as well. As sjSS is supposed to be underestimated in collagenoses and MCTD, screening of salivary gland by US is usefull even in patients without sicca-symptoms to identified typical, inhomogeneous parchenchymal appearance with hypoechoic lesions suggestive for jSS.

References:

1. Hocevar A. et al;. Rheumatology 2005; Vol 44; pp 768

2. Krumrey-Langkammerer M. et al. 2015: Ultraschall in Med 2015; 36 - A349

**Disclosure of Interest:** None Declared

### P50 Toward the achievement of an agreement between clinical and ultrasound assessment of the ankle region

#### Stefano Lanni^1^, Alessandra Alongi^1^, Adele Civino^2^, Alessandro Consolaro^1,3^, Giovanni Filocamo^4^, Angelo Ravelli^1,3^

##### ^1^IRCCS Istituto Giannina Gaslini, Genova, Italy; ^2^Pia Fondazione di Culto e Religione Card. G. Panico, Tricase, Italy; ^3^Università degli Studi di Genova, Genova, Italy; ^4^Fondazione IRCCS Ca' Granda Ospedale Maggiore Policlinico, Milano, Italy

###### **Correspondence:** Stefano Lanni

**Introduction:** The ankle is a complex anatomical structure owing to the multiple joint recesses and surrounding tendons. The clinical examination of this joint is a challenging task even for the expert physician. Ultrasound (US) is becoming a useful adjunctive tool to clinical evaluation for the assessment of children with juvenile idiopathic arthritis (JIA). Disagreement between clinical and US examinations in the assessment of the ankle region has been shown in some studies.

**Objectives:** The aims of the study were: 1) to assess the frequency of clinical signs of articular and periarticular involvement and of US abnormalities in the different joint recesses and tendon compartments of the ankle region; 2) to investigate the correlation between clinical signs of articular involvement and US abnormalities in the different joint recesses of the ankle region.

**Methods:** Twenty-seven ankles of 19 patients with JIA with a clinical suspicion of disease involvement were included in the study. At the same consultation, the ankles were scanned by a physician with experience in the US assessment of children with JIA, who was blinded to clinical findings. Clinical and US evaluations focused on tibiotalar and subtalar joints, the tarsal area and on tendon compartments. For each of the joint recesses the presence of swelling, pain on motion and restricted motion and the detection of joint effusion (JE), synovial hypertrophy (SH) and power Doppler (PD) signal inside the area of SH were recorded on clinical and on US evaluation, respectively. US abnormalities were graded on a 4-point semiquantitative scale. Correlation between clinical signs of articular involvement and US abnormalities in the different joint recesses of the ankle region was estimated using the Kendall’s tau (τ) coefficient.

**Results:** Overall, on clinical assessment swelling was found more frequently in the tibiotalar joint (52%), whereas both pain and restricted motion were documented more commonly in the subtalar joint (44% and 41%, respectively). On US assessment JE and SH were detected more frequently in the tibiotalar joint (56% and 67%, respectively); PD signal was displayed more commonly in the subtalar joint (44%). Among tendon compartments, both clinical and US assessments documented a more frequent involvement of flexor tendons (11% and 52%, respectively). Correlation was found between presence of pain or restricted motion on clinical evaluation and detection of PD signal on US in the tibiotalar joint (τ = 0,58, p = 0,002 and τ = 0,57, p = 0,002, respectively). In the tarsal area correlation was found between presence of restricted motion on clinical assessment and detection of PD signal on US (τ = 0,43, p = 0,024) and between presence of pain on clinical assessment and detection of SH on US (τ = 0,41, p = 0,033). No correlation was found in the subtalar joint between any clinical sign of articular involvement and US abnormalities.

**Conclusion:** Tibiotalar and subtalar joints are more commonly affected than the tarsal area on both clinical and US assessment in case of ankle involvement in JIA. Flexor tendons are more frequently inflamed than the anterior and lateral tendon compartments. On clinical examination, pain or restricted motion, but not swelling, in the tibiotalar joint and in the tarsal area seem to correlate with the presence of synovitis on US in these joint recesses of the ankle region. The assessment of the subtalar joint remains a challenging task for the physician without the use of US.

**Disclosure of Interest:** None Declared

### P51 Kawasaki disease: initial echocardiogram predicts subsequent coronary disease and immunoglobuline resistance

#### Dima Chbeir^1^, Jean Gaschinard^1^, Ronan Bonnefoy^1^, Constance Beyler^2^, Isabelle Melki^1^, Albert Faye^1^, Ulrich Meinzer ^1^

##### ^1^Pédiatrie générale, maladies infectieuses et médecine interne, Paris, France ^2^Service de cardiologie pédiatrique, Hôpital Robert Debré, Paris, France

###### **Correspondence:** Ulrich Meinzer

**Introduction:** Kawasaki disease (KD) is an acute febrile systemic vasculitis that affects blood vessels of small and medium calibre. With the availability of intensified treatments for most severe patients, it is crucial to early identify patients at high risk for coronary artery aneurysms (CAA). However, the available risk scoring systems from Japan have not been validated in European populations. There is little data concerning the link between initial echocardiogram findings and cardiac prognosis.

**Objectives:** To investigate the first echocardiogram can predict resistance to conventional therapy and/or subsequent development of CAA

**Methods:** We retrospectively analysed demographic, clinical, biological, echocardiographic and therapeutic data from children diagnosed with KD between 2006 and 2016 at the Robert-Debré University Hospital, Paris, France.

**Results:** A total of 157 children with KD were included. Initial echocardiogram was performed after a median of 6 days of fever and was abnormal in 48 cases (31%). The initial presence of any echocardiographic abnormality was strongly associated with resistance to intravenous immunoglobulin (p = 0.005) and development of coronary artery lesions within the first six weeks of disease (p = 0.01). All patients (n = 7) with persistent coronary abnormalities at one year already had an abnormal initial echocardiogram. In contrast, severity scores had low sensitivity (24-33%) and low specificity (72-81%) to predict immunoglobulin resistance or cardiac involvement.

**Conclusion:** Abnormalities in the initial echocardiogram can be used to early identify patients with severe Kawasaki disease in populations with mixed ethnic backgrounds.

**Disclosure of Interest:** None Declared

### P52 Childhood-onset Takayasu arteritis: a referral center experience in Turkey

#### Sezgin Sahin^1^, Duhan Hopurcuoglu^1^, Sule Bektas^1^, Ezgi Belhan^1^, Amra Adrovic^1^, Kenan Barut^1^, Nur Canpolat^2^, Salim Calıskan^2^, Lale Sever^2^, Ozgur Kasapcopur^1^

##### ^1^Pediatric Rheumatology, Istanbul University, Cerrahpasa Medical School, Istanbul, Turkey ^2^Pediatric Nephrology, Istanbul University, Cerrahpasa Medical School, Istanbul, Turkey

###### **Correspondence:** Sezgin Sahin

**Introduction:** Takayasu arteritis is a chronic granulomatous vasculitis that effects primarily aorta and its main branches and rarely seen before the age of 18. Delay in diagnosis and treatment is a common feature due to the nonspecific systemic features. There is limited data on clinical features and long-term outcome of Takayasu arteritis. Also, there is need to evaluate efficacy of current therapies, since the spectrum of treatment options have been increased compared to the past.

**Objectives:** We aimed to report our referral center experience on the clinical features and treatment options of Takayasu arteritis. In addition, we have evaluated the correlation of various activity and damage scores with eachother

**Methods:** We performed a retrospective chart review for Takayasu arteritis between October 2002 and October 2017 at our tertiary referral pediatric rheumatology center. In addition to the current cases that are followed-up, we have comprehensively searched the hospital database for the ICD-9 code 446.7 and ICD-10 code M31.4 to identify Takayasu arteritis patients.

**Results:** Overall, 16 patients (12 female) have been diagnosed with Takayasu arteritis in last 15 years. Mean age of the patients at disease onset and diagnosis were 10.9 ± 4.8 years and 11.5 ± 4.7 years, respectively. While the median duration from the onset of first symptom until the diagnosis was 2.4 months (range 0.1-65 months), the mean disease duration was 6.1 ± 5.9 years. The most frequent manifestation at admission was nonspecific systemic features (fever, fatigue, anorexia, weight loss) (n = 13, 81.2%) followed by hypertension (n = 10, 62.5%) and neurologic findings (n = 4, 25%). Tuberculosis disease was also detected in 4/16 subjects (25%) at disease onset and anti-tuberculosis therapy initiated concomitantly with immunosuppressive drugs. All patients had met the ACR 1990 criteria for the classification of Takayasu arteritis. The most common angiographic type was Numano’s type IV seen in 5 patients, followed by type V in 4, type III in 4, type IIa in 2, type IIb and type I one patient each. Subjects had extensive disease at presentation with mean DEI.Tak (±SD) of 13.8 (±6); All of the subjects had elevated acute-phase reactants, and were active at presentation [mean ITAS2010 score (±SD): 18.7 (±7.1) and mean PVAS score (±SD): 14.0 (±4.4)). These three activity scores correlated with each other both at disease onset and last visit (p < 0.05). The most frequently used immunosuppressive drug in therapy was corticosteroids (n = 16/16, 100%), followed by azathioprine (n = 15/16, 93.7%), cyclophosphamide (n = 10/16, 62.5%), methotrexate (n = 6/16, 37.5%) and tocilizumab (n = 6/16, 37.5%). The rate of angioplasty and/or bypass was not rare (n = 7/16, 43.7%). Despite the high frequency of surgical procedure, there was no deceased patient with Takayasu arteritis in the last 15 years.

**Conclusion:** There is no deceased Takayasu patient in the last 15 years. Fairly good clinical outcomes compared to the past may be due to new biologic drugs and/or improved surgical techniques. Three different disease activity scores were in a great concordance with each other.

References

1. Brunner J, Feldman BM, Tyrrell PN, et al. Takayasu arteritis in children and adolescents. Rheumatology (Oxford) 2010; 49(10): 1806-14.

2. Eleftheriou D, Varnier G, Dolezalova P. Takayasu arteritis in childhood: retrospective experience from a tertiary referral centre in the United Kingdom. Arthritis Res Ther 2015; 17: 36.

3. Misra DP, Aggarwal A, Lawrence A, Agarwal V, Misra R. Pediatric-onset Takayasu's arteritis: clinical features and short-term outcome. Rheumatol Int 2015; 35: 1701-6.

**Disclosure of Interest:** None Declared

### P53 Low dose CT coronary angiography and calcium scoring in patients with Kawasaki disease in convalescent phase- a preliminary study

#### Rajkumar Chakraborty^1^, Surjit Singh^2^, Manphool Singhal^1^, Deepti Suri^2^

##### ^1^Radiodiagnosis and Imaging, Post Graduate Institute of Medical Education and Research, Chandigarh, India, Chandigarh, India; ^2^Allergy immunology Unit, Advanced Pediatrics Centre, Post Graduate Institute of Medical Education and Research, Chandigarh, India, Chandigarh, India

###### **Correspondence:** Surjit Singh

**Introduction:** Development of coronary artery abnormalities (CAA) is the hallmark of Kawasaki disease (KD) and accounts for most of the morbidity and mortality associated with the disease. Concerns have been raised as to whether patients with KD are really at risk for premature atherosclerosis later in adulthood. With advancements in technology, dual-source computerized tomography (DSCT) coronary angiography can be developed as a low radiation imaging paradigm for patients with KD. Advantages over echocardiography include the ability to visualize middle and distal coronary segments with little or no inter-observer variability. Unlike catheter angiography, DSCT angiography is non-invasive and clearly delineates calcification and intraluminal abnormalities.

**Objectives:** This study is designed to evaluate whether KD is a risk factor for premature atherosclerosis by calcium scoring (Agatson’s algorithm) and to detect the CAA (aneurysm/thrombus/stenosis/ectasia) using DSCT with low radiation protocols.

**Methods:** In this prospective study, 21 patients (male: female- 15:6) with KD in convalescence for more than 10 years were enrolled (mean: 15.76 years). Diagnosis of KD was based on the 2004 American Heart Association (AHA) Criteria for KD. Written informed consent was taken from all the patients prior to the enrolment. Patients were scanned during a single breath-hold with a customized protocol designed to minimize the administered radiation dose. Non-ionic contrast (Omnipaque 350, GE Healthcare, Ireland) 2-4 ml/kg was injected at a rate of 4-5 ml/s (through 20G cannula), in the right antecubital vein, followed by a saline push (1-2 mL/kg at a rate of 1.5 mL/second) with a dual head power injector. An 128 slice SOMATOM Definition flash DSCT scanner (SIEMENS HEALTH CARE; Germany) was used for coronary angiography. The data acquisition parameters were 80-100 KVp voltage, tube current (mAs) (care dose 4D), detector configuration 128x0.6 mm, gantry rotation time 0.28 seconds, and the scan length was adjusted from the scout image to encompass the entire heart. The scan was taken from carina till the apex of heart. Images were analyzed on dedicated work station using proprietary soft ware (Syngovia). Coronary arteries was evaluated using the 13 segment model proposed by the AHA. The calcium score was calculated by determining the density of the highest density pixel in each plaque taking the threshold of 130 HU and applying a weighting factor to each plaque, depending upon the peak density in the plaque. Heart rate at which data was acquired, was recorded along with electrocardiogram tracing.

**Results:** Mean age of the patients at the time of diagnosis was 3.21 years and mean time interval time interval between diagnosis and imaging 12.59 yrs. At the time of data acquisition the mean heart rate of the patients were 80 beat/min, mean tube current 2417.66 mAs, median scan time 2.5 seconds and median effective dose 2.52 mSv. CAAs are seen in the form of aneurysms and ectasias in two patients (9.52%) in our study. No patient with significant stenosis was seen. The aneurysms were detected in proximal segments of RCA (Saccular, medium sized) and LAD (fusiform, small sized). The ectasias were detected in one patient (4.76%) in LAD and LCx. Raised calcium score is seen only one (4.76%) patient with abnormal coronary arteries. The calcium deposition is seen in only in abnormal segments.

**Conclusion:** Our study suggests that KD itself may not be a cause for premature atherosclerosis unless the coronary arteries are involved in form of aneurysms. However, the results of this preliminary study done in a small population size has to be replicated in larger cohorts to make a firm conclusion.

**Disclosure of Interest:** None Declared

### P54 Low dose dual source CT coronary angiography and calcium scoring in children with Kawasaki disease not treated with intravenous immunoglobulin– a preliminary study

#### Santosh Dusad^1^, Surjit Singh^1^, Manphool Singhal^2^, Deepti Suri^1^

##### ^1^Allergy immunology Unit, Advanced Pediatrics Centre, Post Graduate Institute of Medical Education and Research, Chandigarh, India, Chandigarh, India ^2^Radiodiagnosis and Imaging, Post Graduate Institute of Medical Education and Research, Chandigarh, India, Chandigarh, India

###### **Correspondence:** Surjit Singh

**Introduction:** Coronary artery abnormalities (CAA) develop in 20-25% of untreated cases and 3-5% of treated cases of Kawasaki disease (KD), that accounts for most of the morbidity and mortality associated with the disease. Although, transthoracic 2-D Echocardiography (2DE) is the initial preferred modality for imaging coronary arteries, it has several inherent limitations- unclear views of middle and distal segments of coronaries, chances of inter-observer variation, and poor acoustic windows in older children to view the coronaries. Dual source CT (DSCT) coronary angiography has recently emerged as an non-invasive tool that has low radiation exposure and can accurately demonstrate the coronary wall anatomy and intraluminal abnormalities like thrombosis, stenosis, calcification. It has several advantages over echocardiography including the ability to visualize middle and distal coronary segments with little or no inter-observer variability.

**Objectives:** To evaluate the role of DSCT coronary angiography in detecting CAAs in children with KD who were not treated with intravenous immunoglobulin during acute stage and its comparison with 2DE.

**Methods:** Nineteen (19) children (M:F = 15:4) with KD who did not receive intravenous immunoglobulin and are on regular follow up in Pediatric Rheumatology Clinic were enrolled (median age: 11.03 years; range: 3.7-23 years). Diagnosis was based on diagnostic criteria given by AHA. All of the patients were the cases of incomplete KD and were not given IVIg because they either presented late in convalescent phase or when then presented to us, they were afebrile and ESR, CRP were not raised. Mean duration since diagnosis of KD to the time when study was conducted was 3.84 ± 2.27 years. The study was approved by the Ethics Committee of the Institute. A written informed consent from the parents was obtained prior to enrolment. Children underwent both SOMATOM 128-slice Definition flash DSCT coronary angiography (SIEMENS HEALTH CARE; Germany) and 2DE on the same day. Diameters of major coronary arteries were measured and presence of any coronary artery lesions was looked for. Results of 2DE carried out at the time of diagnosis were also taken into account. Student paired T test was used to compare the average diameter of coronaries obtained by two imaging modalities.

**Results:** None of the patients showed any coronary artery abnormalities by both DSCT and 2DE. All coronary arteries were well visualized by DSCT and even LCX branches and distal RCA was also well visualized. However, coronary artery could not be visualized in 7 patients out of 19 by 2DE. In 3 patients, even main arteries were also not seen due to poor acoustic window. However, in 4 cases, RCA and LMCA were well visualized, but it was difficult to visualize left circumflex artery clearly. The 2DE and DSCT had almost the same results of proximal coronary artery especially LMCA, proximal RCA, and LAD. However, DSCT provided the more accurate visualization of distal coronary artery than 2DE. The average diameter of coronary artery measured by 2DE and CT angiography was almost similar. 2DE at the time of diagnosis showed dilatation of coronaries in 3 children (2 LMCA, 1 LAD) which were not seen in current echo and DSCT.

**Conclusion:** DSCT coronary angiography more accurately delineated the anatomy and lumen of coronary arteries especially in older children with KD when compared with 2DE. DSCT is superior to echo for distal coronary artery visualization. It can reveal coronary artery lesions in patients with KD in timely manner and help with therapeutic decision making to improve the prognosis.

**Disclosure of Interest:** None Declared

### P55 Flow mediated dilatation of brachial artery during the acute and convalescent stages of Kawasaki disease-a preliminary study

#### Santosh Kumar^1^, Surjit Singh^1^, Manphool Singhal^2^, Vivek Kumar^3^, Deepti Suri^1^

##### ^1^Allergy immunology Unit, Advanced Pediatrics Centre, Post Graduate Institute of Medical Education and Research, Chandigarh, India, ^2^Radiodiagnosis and Imaging, Post Graduate Institute of Medical Education and Research, Chandigarh, India, ^3^Nephrology, Post Graduate Institute of Medical Education and Research, Chandigarh, India,

###### **Correspondence:** Surjit Singh

**Introduction:** Kawasaki disease (KD) is usually considered as an acute, self-limited vasculitis of childhood. However, of late, several long-term complications including development of major coronary events and premature atherosclerosis have been recognized in patients with KD. The evaluation of vascular endothelial function is important in predicting several long-term complications in patients with KD. Flow mediated dilatation (FMD) of the brachial arteries is considered one of the non-invasive ways to study the vascular endothelial dysfunction.

**Objectives:** To study the FMD of brachial artery (BA) and evaluate for endothelial dysfunction in children with KD in both acute and convalescent phases.

**Methods:** Sixteen (16) children with KD and an equal number of controls were enrolled. The diagnosis of KD was based on the standard clinical diagnostic criteria enunciated by the 2004 American Heart Association (AHA). FDA approved automated edge detection software program (Brachial artery analyser^TM^) combined with high resolution ultrasound images were used to study the BA. Ultrasonography (USG) on a standard Philips Medical system was performed by a trained and expert radiologist. The software provided the diameter measurement every second. A 10-MHz multifrequency linear array probe attached to a high-resolution ultrasound machine was used to acquire images of the right BA. Baseline images of the right brachial artery were obtained for 2 minutes. A forearm cuff was positioned 1 cm under the antecubital fossa and inflated to 250 mm Hg for 5 minutes to induce forearm-reactive hyperemia. The cuff was then released and images of BA were recorded continuously for next 5 minutes. The basal diameter was taken as the average of measures collected during the first minute, and FMD was calculated as the maximum percentage change in the brachial artery diameter compared with the basal value. The USG was repeated in convalescent stage, at least three months later the diagnosis.

**Results:** Mean age of the study population was 4.8 years in cases (n = 16; M:F- 12:4) and 5.75 years in control population (n = 16; M:F- 10:6). The baseline BA diameter in acute phase of KD children is 2.3 ± 0.34 mm and that of convalescent phase is 2.49 ± 0.35 mm. Healthy control population had a mean baseline BA diameter of 2.35 ± 0.46 mm. Maximum dilatation was 2.56 ± 0.36 mm in the group of acute KD, 2.93 ± 0.31 mm in the group of convalescent KD and 2.95 ± 0.56 mm in healthy controls (p- 0.02). Children with KD had FMD of 12.32 ± 6.2% in acute phase and 17.99 ± 8.13% in convalescent phase, whereas, the control population had FMD of 26.88 ± 12.76% (p <0.01).

**Conclusion:** Our results suggest that FMD is significantly reduced in children with KD in acute phase and convalescent phase as compared to healthy controls. To the best of our knowledge, ours is the first study done in both phases of KD and suggests that endothelial dysfunction starts as early as in the acute phase of KD.

**Disclosure of Interest:** None Declared

### P56 Discrepancies between clinical assessment and MR imaging in JIA

#### E. Charlotte van Gulik^1^, Robert Hemke^1^, Mendy M. Welsink-Karssies^2^, Dieneke Schonenberg^1^, Koert M. Dolman^3^, Anouk M. Barendregt^1^, Charlotte M. Nusman^1^, Taco W. Kuijpers^1^, Mario Maas^1^, J. Merlijn van den Berg^1^

##### ^1^AMC, Amsterdam, Netherlands; ^2^OLVG, Amsterdam, Netherlands; ^3^Reade, Amsterdam, Netherlands

###### **Correspondence:** E. Charlotte van Gulik

**Introduction:** With the increased use of imaging as a supportive tool for starting or intensifying treatment in JIA patients, discrepancy between the MR imaging and clinical assessment will pose a dilemma.

Objectives

To evaluate patient characteristics and disease activity parameters in a single tertiary center cohort of JIA patients, both with and without synovial hypertrophy upon MRI, in order to explore frequency of mismatching results.

Methods

Clinical, laboratory, and contrast-enhanced MRI data of the knee of prospectively enrolled JIA patients from 2008-2014 were analyzed. JIA was diagnosed according to the ILAR criteria. The Wallace criteria for inactive disease were used. The validated Juvenile Arthritis MRI Scoring (JAMRIS) system for the knee was used to evaluate the presence of synovial hypertrophy (1). Clinically active and inactive patients were, as separate groups, divided into two groups based on the JAMRIS (four groups in total): JAMRIS score ≥1, meaning synovial hypertrophy, or JAMRIS score 0. Patient characteristics and disease activity parameters were then compared using the Chi-square and Fishers exact test if data were categorical and with the Mann–Whitney U if continuous.

Results

The MRI showed contradictory findings with reference to the clinical assessment in 43 of 124 patients (34.7%) (Table 20). The clinically active patients with discordant findings ((i.e. no synovial hypertrophy seen on MRI; n = 25) were significantly older and had been more often diagnosed with polyarticular JIA, compared to the clinically active patients with concordant findings (n = 47) (median age 13.2 years vs 10.9; p = 0.006 and 72% vs 34%; p = 0.003, respectively). The clinically inactive patients with discordant findings (i.e. synovial hypertrophy seen on MRI; n = 18) were significantly younger than the clinically inactive patients with concordant findings (median age 10.7 years vs 14.4; p = 0.008). Other clinical parameters, including CHAQ, laboratory results and medication usage were, however, *not* significantly different between the discordant and concordant groups (Table 20).

**Table 20:** **(Abstract P56).** See text for description

	Clinically Active			Clinically Inactive		
Variable	Concordant n = 47	Discordant n = 25	p-value	Concordant n = 34	Discordant n = 18	p-value
Female gender, n(%)	26 (55.3)	18 (72.0)	0.209	23 (44.2)	10 (19.2)	0.389
Age, years	10.9 (8.6-13.7)	13.2 (11.3-15.6)	0.006	14.4 (12.1-16.3)	10.7 (9.3-13.6)	0.008
Oligoarticular JIA, n(%)	21 (44.7)	2 (8.0)	0.001	11 (32.4)	11 (61.1)	0.076
Polyarticular JIA, n(%)	16 (34.0)	18 (72.0)	0.003	19 (55.9)	4 (22.2)	0.038
Other subtypes, n(%)	10 (21.3)	5 (20)	1.000	4 (11.8)	3 (16.7)	0.682

Conclusion

Nearly 35% of the JIA patients showed discordant findings between clinical assessment and MRI. Clinically active patients without synovial hypertrophy on MRI were significantly older at the time of MRI than the clinically active patients with synovial hypertrophy on MRI.

In the clinically inactive JIA patients, the discrepancy between clinical assessment and MRI was especially seen in younger patients. The meaning of synovial hypertrophy in the clinically inactive patient is still unknown, but it might indicate an incomplete disease remission.

References

1. Hemke R, van Rossum MA, van Veenendaal M, Terra MP, Deurloo EE, de Jonge MC, et al. Reliability and responsiveness of the Juvenile Arthritis MRI Scoring (JAMRIS) system for the knee. European radiology. 2013;23(4):1075-83.

**Disclosure of Interest:** None Declared

### P57 Flow cytometry based assay of platelet reactivity in children with Kawasaki disease- a preliminary study

#### Pandiarajan Vignesh^1^, Surjit Singh^1^, Amit Rawat^1^, Man Updesh S. Sachdeva^2^, Jasmina Ahluwalia^2^

##### ^1^ALLERGY IMMUNOLOGY UNIT, PEDIATRICS, POSTGRADUATE INSTITUTE OF MEDICAL EDUCATION AND RESEARCH, Chandigarh, India; ^2^Hematology, POSTGRADUATE INSTITUTE OF MEDICAL EDUCATION AND RESEARCH, Chandigarh, India

###### **Correspondence:** Pandiarajan Vignesh

**Introduction:** The acute phase of Kawasaki disease (KD) involve endothelial damage and activation of platelets. Markers of platelet activation were found to be elevated in adults with coronary artery disease (CAD) and venous thromboembolism. The actual evidence for platelet activation in KD, however, is limited to platelet aggregation studies and few studies on platelet activation markers and circulating microparticles. Monocyte-platelet aggregates (MPA), one of the most sensitive markers for activated platelets has not been studied in the acute phase of KD.

**Objectives:** To assess platelet reactivity by flow cytometry based measurement of MPA in children with KD.

**Methods:** Fourteen (14) children with KD, 15 age-matched febrile controls, and 13 age-matched healthy controls were enrolled in this prospective study. Diagnosis of KD was based on the American Heart Association (AHA) 2004 criteria. Samples were collected at three different phases of illness in patients with KD- acute stage prior to administration of IVIg and aspirin, 24 hours after administration of IVIg, and 3 months after onset of illness. Immediate fixation of the sample and lysis of red blood cells (RBC) were carried out at the room temperature with the use of BD FACS lysing solution. The washed samples were immunolabeled with a saturating concentration of PerCP-conjugated CD14 (lipopolysaccharide-protein receptor, BD Pharmingen^TM^, *Catalogue no.* 555398) and fluorescein isothiocyanate (FITC) conjugated GP IIb (CD41, BD Pharmingen^TM^, *Catalogue no.* 555466). Following incubation for 20 minutes at room temperature, the cells were acquired on the flow cytometry (Beckman coulter, Navios). The monocytes were gated from the lymphocyte side scatter plot and MPAs was identified from the monocyte population that had expressed both CD14 (monocyte marker) and CD41 (platelet marker) using the *Kaluza* software.

**Results:** The median age group (inter-quartile range) in the cases, febrile controls, and normal controls were 6 (3.0, 7.25), 5 (3.6, 9.0), and 5.5 years (4.15, 6.5), respectively. Male to female ratio among the groups were 12:2, 13:2, and 11:2 respectively. Echocardiographic abnormalities were detected in 3 children (2- transient abnormalities such as coronary wall brightness and coronary ectasia, 1- giant coronary aneurysms). Percentage of MPA% values were significantly high in children with KD [Median (IQR)- 41.3% (26.6, 52.7)] compared to the febrile [Median (IQR)- 5.98% (2.98, 9.72)] and normal controls [Median (IQR)- 4.48% (2.57, 5.59)], p < 0.01. A consistent drop in serial MPA% was observed from the diagnosis of KD to the end of third month follow-up [Table 21]. The MPA% values at 3 months post diagnosis of KD [Mean (SD)- 9.59% (7.65)] were significantly higher compared to the normal controls [Mean (SD)- 4.3% (2.03)], p-0.004.

**Table 21:** **(Abstract P57).** See text for description Median percentage values of MPA (CD14 + CD41+) in children with KD in follow-up: at diagnosis, 24 hour after completion of IVIg infusion and 3 months after diagnosis

S no	Category	Median MPA% (IQR)	P value
1	At enrolment (n = 14)	41.31 (26.6, 52.69)	<0.001*
2	24 hours after IVIg (n = 14)	18.55 (9.2, 22.99)	
3	Follow-up at 3rd month (n = 11)	7.55 (4.15, 14.6)	

*p < 0.05 considered significant

**Conclusion:** MPA% as an objective measure of platelet activation was significantly elevated in children with acute stages of KD when compared with age and sex-matched febrile and normal controls. Elevated levels of MPA% in patients with KD 3 months post diagnosis suggest that the endothelial inflammation or damage in KD persists for a longer duration even after control of symptoms and signs of systemic inflammation. Long-term studies on platelet activation markers in a larger multicentric cohort would conclusively establish the role of platelets in acute and long-term cardiac morbidity in KD.

**Disclosure of Interest:** None Declared

## Immunodeficiency and infection related arthritis

### P58 Deficiency of CD70 is responsible of a case of chronic active EBV (CAEBV) infection presenting as periodic fever

#### Roberta Caorsi^1^, Marta Rusmini^2^, Stefano Volpi^1^, Sabrina Chiesa^1^, Caludia Pastorino^1^, Francesca Minoia^1^, Alice Grossi^2^, Sara Signa^1,3^, Paolo Picco^1^, Angelo Ravelli^1,3^, Isabella Ceccherini^2^, Marco Gattorno^1^

##### ^1^Second division of Pediatrics, G. Gaslini Institute, Genova, Italy; ^2^Division of Human Genetics, G. Gaslini Institute, Genova, Italy’; ^3^DINOMGI, University of Genova, Genova, Italy

###### **Correspondence:** Roberta Caorsi

**Introduction:** Chronic active EBV (CAEBV) infection is a rare condition associated to a chronic activation of Epstein Barr virus and therefore to a chronic and potentially life-threating lymphoproliferation. The prognosis is poor: most of the patients not treated with bone marrow transplantation die for lymphatic malignancies and the common immunosuppressive and antiviral therapy are usually not effective. Even if with a wide heterogeneity, most of the patients with CAEBV present severe clinical manifestations with early onset and poor prognosis; the presence of an underlying immunodeficiency causing an unusual EBV replication is therefore reasonable.

**Objectives:** To describe the clinical course and the genetic characterization of a patient with CAEBV mimicking PFAPA at disease onset.

**Methods:** In a patient with CAEBV infection a whole exome sequencing (WES) approach was undertaken and variants were prioritized with a custom pipeline to identify the genetic cause of his condition that was validated through Sanger Sequencing in the trio.

**Results:** The patient, born of consanguineous parents, at the age of 15 months presented, in complete wellbeing, a not-complicated infectious mononucleosis; in the following months he presented recurrent episodes of high fever with exudative tonsillitis, adenitis, splenomegaly and sweating, lasting 3-5 days and treated with NSAIDS or antibiotics. Blood examinations revealed neutrophilic leukocytosis and elevation of acute phase reactants; plasmatic immunoglobulins were within the normal range. An autoinflammatory condition with periodic fever was suspected and therefore on-demand steroidal treatment was suggested, with good response. The child continued to present periodic fever, associated to the occurrence of respiratory infections requiring antibiotics, and recurrent episodes of cheratitis. Several destructive dental caries were found as well as hyper sensibility to mosquitoes bite.

Immunologic test revealed then a reduction in the level of plasmatic immunoglobulins. The detection of EBV DNA with quantitative PCR revealed 21935 copies for 100000 leucocytes with prevalence of infection in the B cells. Whole body positron emission tomography revealed a retroperitoneal formation of about 35 mm with an increased metabolism that, at biopsy, revealed a reactive lymphadenopathy with paracortical involvement associated with EBV infection.

Genetic conditions associated to chronic EBV infection and immunodeficiency were ruled out: the genetic tests for SAP, XIAP, BAFF-R and ICOS were negative and the cytofluorimetric analysis of perforin, CD107 and 2B4 receptor were normal. In light of these findings a CAEBV was suspected.

Being the patient in good general conditions and in light of the prevalent involvement of CD20+ lymphocytes, on demand treatment with rituximab was started. The clinical response to treatment was initially very satisfying; however after two years the child presented severe infections requiring prolonged hospitalisation; stem cell transplantation was then performed with a complete normalization of both clinical and immunological features.

Among variants identified through a WES analysis of the patients and his parents, a homozygous mutation of the CD70 gene appeared to fit the recessive model of inheritance. The variantc.163-2A > G affects the exon2AG-acceptor splice site of the CD70 gene (NM_001252) leading to a deficiency in CD70, the ligand of CD27, a gene involved in isolated immunodeficiency. In the meantime our analysis was ongoing, CD70 mutations were reported in a few patients with a similar condition.

At the protein level, the anti-CD70 antibody failed to detect CD70 by flow cytometry at the cell surface of PHA-stimulated T and EBV-trasformed B cells of the patient. In contrast, expression of CD70 was detectable on cells from healthy donors.

**Conclusion:** This case describe a new case of a newly identified genetic cause of CAEVB presenting with recurrent periodic fever**.**

**Disclosure of Interest:** None Declared

### P59 Clostridium difficile enterocolitis and reactive arthritis: a case report ad re-view of the literature

#### Lorenzo Mambelli^1^, Michela Cappella^2^, Martina Mainetti^1^, Federico Marchetti^1^

##### ^1^Pediatrics, S. Maria delle Croci Hospital, Ravenna, Italy; ^2^Pediatric and Adolescence Rheumatology, S.Maria Nuova Hospital, Reggio Emilia, Italy

###### **Correspondence:** Lorenzo Mambelli

We report the case of a 6-year-old boy with pain in the right knee and both ankles after a 10-day course of oral amoxicillin-clavulanate completed three weeks earlier; at the same time he developed diarrhea with positive culture for *Clostridium difficile* (CD). Three days later the knee and ankles arthritis resolved spontaneously but appeared in the right shoulder and homolateral hip with a migratory pattern and with fever. Synovial fluid and blood cultures, rheumatoid factor, ANA and HLA-B27 antigen were negative. The improvement of colitis and the negativity of fecal calprotectin at follow-up allowed to exclude an inflammatory bowel disease. Nonsteroidal anti-inflammatory drugs (NSAIDs) and metronidazole completely resolved pain, joint swelling and diarrhea. After 24 months of follow-up there has been no recurrence.

Reactive Arthritis (ReA) is an aseptic acute inflammatory arthritis occurring after an intercurrent infection in which the causative organism is outside from the joint. It is typically an asymmetrical oligo/polyarthritis predominantly in the lower limbs large joints (knee, ankle, hip) and present approximately 2-4 weeks following infection. Several patients present a migratory pattern and acute arthritis is often characterized by severe pain and sometimes erythema over the affected joints; enthesitis/tenosynovitis may occur. Most frequent causes are S*almonella, Shigella, Yersinia, C. jejuni, group A Streptococcus* and *C. trachomatis.*

CD is the most common cause of diarrhea after antibiotic therapy but CD ReA is rare and its pediatric epidemiology is poorly known; it was generally migratory and polyarticular, involving large joint and tipically with a good outcome and self-limiting course.

For the diagnosis of ReaA following CD enterocolitis there are criteria established by Putterman and Rubinow [1], but basically a history of diarrhoea after a course of antibiotic with the exclusion of other causes of gastroenteritis or noninfectious arthritis is essential.

Jacobs et al [2] documented until 2001 35 cases of CD ReA in adults; epidemiology in child instead is rare and unknown, but incidence of CD infection has increased among children. We performed a literature search of MEDLINE, Google Scholar, and Cochrane Reviews computerized databases using the keywords “Clostridium difficile”, “arthritis”, and “child” to identify all papers reporting CD enterocolitis-associated ReA in childhood. Six papers met our review's inclusion criteria with a total of 32 pediatrics cases of CD ReA. The most recent study identified, from 2004 to 2013, 26 cases with acute arthritis/tenosynovitis after CD infection and authors estimate that CD ReA affected 1.4% of child with CD infection; only 35% of CD ReA were correctly diagnosed, occasionally misdiagnosed as septic arthritis [3].

Conclusion: CD infection should be suspected in children presenting with an acute inflammatory arthritis following an episode of diarrhea, especially when culture for common enteric infection are negative and the patient has received antibiotic therapy before the onset of diarrhea.

Written informed consent for the publication of patient details was obtained.

**Disclosure of Interest:** None Declared

References

[1]Putterman et al,Reactive arthritis associated with Clostridium difficile pseudomembranous colitis.Seminars in Arthritis and Rheumatism;22(6),pp 420-6,1993

[2]Jacobs et al,Extracolonic manifestations of Clostridium difficile infections:Presentation of 2 cases and review of the literature.Medicine;80(2),pp 88-101,2001

[3]Horton et al,Epidemiology of Clostridium difficile Infection–Associated Reactive Arthritis in Children.An Underdiagnosed,Potentially Morbid Condition.JAMA Pediatr. 2016;170(7)

## Juvenile idiopathic arthritis (JIA) in practice

### P60 The effect of task-oriented training with video-based games on activity performance and participation in children with juvenile idiopathic arthritis

#### Nilay Arman^1^, Ela Tarakci^1^, Devrim Tarakci^2^, Ozgur Kasapcopur^3^

##### ^1^Division of Physiotherapy and Rehabilitation, Faculty of Health Science, Istanbul University, Istanbul, Turkey; ^2^Division of Ergotherapy, Istanbul Medipol University, Istanbul, Turkey; ^3^Department of Pediatric Rheumatology, Medical Faculty of Cerrahpasa, Istanbul University, Istanbul, Turkey

###### **Correspondence:** Nilay Arman

**Introduction:** Juvenile idiopathic arthritis (JIA) is most common chronic rheumatic disease in childhood. The upper extremity involvement in JIA causes muscle imbalance, joint destruction, pain, stiffness and limitations on activities of daily living (ADL) in varying degrees. However, the information about prevalence of symptoms, disorders, ADL limitations, participation restriction and options of treatments for upper extremity involvement in JIA are limited. It has been reported that improvements of upper extremity functions were achieved by video-based games (VBG) in various disease groups. However, in the literature, no study has been found about effectiveness of VBG in children with arthritis.

**Objectives:** The aim of the study was to investigate the effects of two different task-oriented activity training to activity performance and participation in children who have involved upper limb with JIA.

**Methods:** 62 patients included in the study were randomized into two groups as group I (patient-centered task-oriented activity training in daily living conditions) and group II (patient-centered task-oriented activity training with VBG). The patients’ pain, by “Numeric Rating Scale (NRS)”, range of motion (ROM) by “goniometer”, muscle and grip strengths by “dynamometer”, activity performance and participation by “Childhood Health Assessment Questionaire (CHAQ)”, “Duruoz Hand Index (DEI)”, “Jebsen-Taylor Hand Function Test (JTHFT)” and “Nine Hole Peg Test (NHPT)” were evaluated. In group I, ADL were trained with real materials used in daily life and various rehabilitation products. In group II, ADL were trained with VBG. We have created training protocol with Xbox 360 games for patient-centered task-oriented activity training with VBG. We prefered ‘Dance Central2’, one of the Xbox 360 games, for warming (macerana dancing, 10 minutes). Other games, ‘Fruit Ninja’, ‘Table Tennis’, ‘Boxing’, ‘Volleyball’, ‘Darts’ and ‘Bowling’ were selected appropriately according to patients performance. All the patients completed 8 weeks (45 minutes for every session, 3 times in a week) of client-centered treatment.

**Results:** After treatment in both groups, significant changes were found in NRS, ROM, muscle strength, grips strength, CHAQ, DEI, JTHFT and NHPT (p < 0,05). Table 1 shows comparison between the groups for the activity performance, pain severity, hand grip and pinch grip strengths. After the treatment, group II was statistically more superior than group I in changes of wrist extension ROM and DEI (p <0.05). In addition to, almost all changes of muscles strength in group II were statistically more superior than changes of group I (p < 0.001).

**Conclusion:** In our study, two different task-oriented activity training provided significant changes in pain, ROMs of upper extremity, muscle strength, grip strength, activity performance and participation and patient-centered task-oriented activity training with video-based games has been proved as an alternative treatment in children who have involved upper limb with JIA.

Trial registration identifying number: Clinical Trial Number: NCT02954718

This work was supported by Research Fund of Istanbul University. Project No: 51144

**Disclosure of Interest:** None Declared

### P61 Analysis of activity performance problems in patients with juvenile idiopathic arthritis

#### Nilay Arman^1^, Ela Tarakci^1^, Ozgur Kasapcopur^2^

##### ^1^Faculty of Health Sciences, Division of Physiotherapy and Rehabilitation, Medical Faculty of Cerrahpasa, Istanbul University, Istanbul, Turkey; ^2^Department of Pediatric Rheumatology, Medical Faculty of Cerrahpasa, Istanbul University, Istanbul, Turkey

###### **Correspondence:** Nilay Arman

**Introduction:** Juvenile idiopathic arthritis (JIA) is the most common chronic rheumatic disease in children and is an important cause of short-term and long-term disability. Many children appeared to have elbow, hand- and/or wrist-related symptoms and impairments, with resulting moderate to severe levels of activity limitations and participation restrictions at daily living. However, in the literature, no study has been found about variety of activity performance problems in patients with JIA. The Canadian Occupational Performance Measure (COPM) is a client-centered, patient reported outcome measure with which clients evaluate their activity performance and satisfaction with performance in areas of self-care, productivity and leisure. COPM is generally used for analyzing activity performance problems in many chronic diseases.

**Objectives:** The aim of the study was to analyze the activity performance problems of patients with JIA.

**Methods:** 50 patients with JIA (42 Female, 8 Male) were included in the study. Inclusion criteria consisted of a diagnosis of JIA according to the International League of Associations for Rheumatology criteria, being aged between 6–18 years, having at least one affected joint in upper extremity (shoulder, elbow, wrist, and finger joints). Activity performance problems as perceived by the individual were measured using face to face interview by the Canadian Occupational Performance Measure (COPM). During the interviews, the patients were encouraged to identify any daily activity that they would like or need to do but found difficult to complete because of their rheumatic diseases. Patients then identified the five most important daily activities and rated, first, their current level of performance, and then, how satisfied they were with this current level of performance. These performance and satisfaction scores were rated by on a 10-point scale, with higher scores indicating better performance and satisfaction. The statistical software SPSS 21.0 was used for the analyses.

**Results:** The mean age was 12,76 ± 3,16 and the mean disease duration was 5,92 ± 3,82 years. 8% of patients had involvement shoulder joint, 74% of them had involvement elbow joint, 88% of them had involvement wrist joint and also 64% of them had involvement finger joint. The patients with JIA described 36 different types of problematic activities and identified 30 of them as the five most important daily activities. Table 22 shows frequencies of five most important daily activities of patients with JIA. The five most identified problems were “carrying something” (66%), “writing” (50%), “opening a bottle cap” (48%), “dressing” (38%) and “opening a door with handle/knob” (38%) according to COPM. The mean of COPM-performance scores was 3,99 ± 1,72 and the mean disease COPM-satisfaction scores was 2,56 ± 1,70.

**Table 22:** **(Abstract P61).** Frequencies of five most important daily activities of patients with JIA

Activity	%	Activity	%	Activity	%	Activity	%
Carrying something	66	Using a knife	20	Drinking	8	Gripping small objects	4
Writing	50	Buttoning	18	Cutting nail	8	Using a scissors	4
Opening a bottle cap	48	Taking a bath	16	Washing dishes	6	Stirring a soup	2
Dressing	38	Peeling a fruit	14	Pushing something	6	Opening a zipper	2
Opening a door with handle/knob	38	Washing hair	14	Removing a sock	4	Acclaiming	2
Combing	28	Tying a shoelace	12	Reaching to grab something	4	Using a fork	2
Unlocking	28	Brushing teeth	10	Unpacking	4		
Tapping	26	Eating	10				

**Conclusion:** Our results showed that patients with JIA, who have at least one affected joint in upper extremity, reported problems with a wide range of activities. We suppose that the COPM can be a useful tool for identifying activity performance problems as a patient-focused outcome measure and also, it could provide information about patient centered management for patients with JIA.

**Disclosure of Interest:** None Declared

### P62 Vaccination coverage in children with rheumatic diseases

#### Maša Bizjak^1^, Tadej Avčin^1,2^, Nataša Toplak^1,2^

##### ^1^Department of Allergology, Rheumatology and Clinical Immunology, University Children's Hospital, University Medical Centre Ljubljana, Ljubljana, Slovenia; ^2^Department of Pediatrics, Faculty of Medicine, University of Ljubljana, Ljubljana, Slovenia

###### **Correspondence:** Maša Bizjak

**Introduction:** The evidence of vaccine safety and efficacy in patients with rheumatic diseases is increasing. However, the data on vaccination status in children with rheumatic diseases remain scarce. So far, only few smaller studies were published that found suboptimal vaccination coverage in patients with rheumatic diseases.

**Objectives:** To assess vaccination status in a cohort of children with rheumatic diseases followed at the University Children's Hospital Ljubljana and to evaluate the most common reasons for vaccination dropout.

**Methods:** All consecutive patients with rheumatic diseases evaluated at the rheumatology outpatient clinic between 1 January 2015 and 15 January 2017 received a questionnaire about their vaccination status and reasons for potential vaccination dropout. Descriptive statistics were used to analyse the proportion of children with full vaccination status at 5, 10, 18 years and at their last clinic visit. The study was approved by the national ethics committee.

**Results:** By May 2017 the data were received from 125 out of 422 enrolled patients (29.6%). Among included patients 64.8% were female, median age was 12.3 (1.5-22.9) years and median age at diagnosis 5.8 (0.7-16.6) years. The majority of included children had JIA (n = 114), followed by SLE (n = 5), CRMO (n = 2), JDM (n = 2), MCTD (n = 1) and systemic sclerosis (n = 1). Vaccination coverage was complete in 92.1%, 81.3%, 72.2% and 71.2% of patients at 5, 10, 18 years and at their last clinic visit, respectively. Most commonly omitted vaccines were Hepatitis B (22.9%) and second dose of Measles, Mumps and Rubella vaccine (18.3%). Most common additional vaccine was against tick-borne encephalitis (26.4%), which is an endemic disease in Slovenia. Most common reasons for vaccination dropout were suggestion of the treating rheumatologist and active disease.

**Conclusion:** We experienced a low initial response rate of 30%, which might be due to increasing parents' vaccine hesitancy. In our sample we found an approximately 10% decrease in complete vaccination coverage between consecutive age cohorts. Overall vaccination coverage in children with rheumatic diseases is lower than in general population in Slovenia. Parent and patient education on vaccination remains crucial and when possible, vaccination catch-up plans and booster vaccinations should be advocated on an individual basis.

**Disclosure of Interest:** None Declared

### P63 Quality of referral letters to pediatric rheumatology and its impact on access to care

Abstract withdrawn

### P64 Contextualizing guidelines for the management of juvenile idiopathic arthritis in developing countries: a needs assessment

#### Mercedes O. Chan^1^, Ricardo Russo^2^, Lawrence Okong'o^3^, Christiaan Scott^4^

##### ^1^Pediatrics (Rheumatology), University of Alberta, Edmonton, Canada; ^2^Immunology and Rheumatology, Hospital de Pediatría Garrahan, Buenos Aires, Argentina; ^3^Paediatrics and Child Health, University of Nairobi, Nairobi, Kenya, ^4^University of Cape Town, Cape Town, South Africa

###### **Correspondence:** Mercedes O. Chan

**Introduction:** Juvenile idiopathic arthritis (JIA) is the most common rheumatic disease of childhood (4/1000 children) and untreated, can lead to significant morbidity including joint deformities, loss of function and blindness (uveitis). Guidelines for the management of JIA have been created for use in the developed world. Challenges in diagnosis and management arise when these guidelines are applied in low-resource settings given different endemic diseases, access to care, and sociocultural practices.

**Objectives:** We performed a needs assessment of clinicians managing JIA in low-resource settings with a view to applying our findings to recommendations for JIA management in developing countries.

**Methods:** An anonymous needs assessment regarding the management of JIA in developing countries was developed based on four areas of attention identified by rheumatologists in the developing world: patient management (access to care and medications, clinical work-up, transition); education (to the health professions and the public); advocacy, networks and policy; and research. The survey (available in English and Spanish) was tested, validated and electronically circulated to healthcare practitioners who see children with JIA in developing countries. Contacts were acquired through pediatric rheumatology professional networks

**Results:** The survey was distributed to 502 practitioners, with 116 responses from clinicians in South America (50%), Africa (28%), Asia (12%), the Middle East (1.5%), Central America (1.5%) and other countries (7%). Respondents were pediatric rheumatologists (66%), adult rheumatologists (22%), allied health professionals (7%), general pediatricians (4%) and general practitioners (1%); median age 40-50 years, median 10-15 years in practice. The percentage of time devoted by these clinicians to pediatric rheumatology in their practice was: <25% (29%), between 26-50% (19%), between 51-75% (13%), >75% (17%) and 100% (22%).

Barriers to accessing care for children with JIA identified by respondents were: insufficient training in JIA among pediatricians (74%), lack of awareness of JIA in the healthcare community (73%), long referral pathways (67%) and few pediatric rheumatologists (63%). Endemic diseases that complicate the management of JIA in participants’ settings were: tuberculosis (84%), HIV (41%), sickle cell anemia (31%), malaria (19%) and diarrheal disease (19%). Transition planning was not a programmed strategy in >40%; and non-adherence to uveitis screening guidelines was reported in >40%.

Education on JIA was available to 71% of pediatrics trainees, 50% of adult rheumatology trainees and <50% of medical students in participants’ settings. Established pediatric rheumatology training programs were unavailable to 40%; access to educational resources and educating the public on JIA was available to only one third of respondents.

Policy and advocacy interventions felt to potentially improve access to care for JIA patients were: increasing the availability of pediatric rheumatologists (79%) and jobs for them (50%), better training on managing JIA (74%), formulation of clear referral channels (65%), formation of patient support groups (62%) and availability of management guidelines (49%).

One third of participants were not involved in research. Epidemiological and clinical research were felt to be research priorities in JIA by 75% and 62% of participants to ascertain local burdens of disease. Major barriers to performing research include: lack of funding (79%), lack of time (58%), lack of support systems, e.g., laboratory (52%), lack of trained personnel (46%) and lack of experience (26%).

**Conclusion:** Management of JIA remains a challenge in the developing world; is rooted in educational gaps in the medical and general community; and complicated by endemic disease and insufficient access to resources. Guidelines specific to low-resource settings, and informed by research exploring the local burdens of disease and experiences of stakeholders, may serve as a powerful educational document to advocate for policy change and best practices.

**Disclosure of Interest**: M. Chan Grant/Research Support from: International League Against Rheumatism Grant, R. Russo Grant/Research Support from: International League Against Rheumatism Grant, L. Okong'o Grant/Research Support from: International League Against Rheumatism Grant, C. Scott Grant/Research Support from: International League Against Rheumatism Grant

### P65 Evaluation of the diagnostic delay and access to remission in JIA patients of the JIR cohorte

#### Caroline Freychet^1^, Natalia Cabrera^2^, Lega Jean Christophe^2^, Michael Hofer^3^, Alexandre Belot^4^ and JIR cohorte

##### ^1^Laboratory HESPER (Health Services and Performance Research), University Claude Bernard Lyon 1, Lyon, France; ^2^Team Evaluation et Modélisations des Effets Thérapeutiques, University Claude Bernard Lyon 1, Lyon, France; ^3^Paediatric Rheumatology, University hospital of Lausanne, Lausanne, Switzerland; ^4^Paediatric Rheumatology, University hospital of Lyon, Lyon, France

###### **Correspondence:** Caroline Freychet

**Introduction:** Many studies in pediatric rheumatology underline the concept of « window of opportunity », early in the disease course to prevent joint or ocular damage in the context of juvenile idiopathic arthritis (JIA). In Europe, despite facilitated access to health care, children with JIA are referred to pediatric rheumatology centres with significant delay. This delay is closely linked to the organization of the country’s health care delivery system. In France and Switzerland nothing is known about diagnostic delay and little about access to remission in JIA.

**Objectives:** To assess the diagnostic delay and the rate of remission at one year and 2 years in JIA patients of the *JIR cohorte*.

**Methods:** The *JIRcohorte* is an international network dedicated to collect prospective and retrospective data on juvenile inflammatory rheumatic diseases. Data collection started in 2013. We included all French and Swiss JIA patients according to the ILAR classification. We focused on the relationship between (i) diagnostic delay (defined by the number of weeks between the first symptoms and the diagnosis), (ii) distance between parent’s home and the place of the diagnosis, and (iii) remission rate at one year, two years and treatments.

**Results:** We included 709 JIA patients (50% oligoarthritis, 17% RF– polyarthritis, 15% enthesitis-related arthritis [ERA], 10% systemic JIA [sJIA], 3% RF+ polyarthritis, 3% psoriatic arthritis, and 2% undifferentiated arthritis).

The median diagnostic delay of the whole cohort was 17 weeks (interquartile range (IQR) 9-48). The patients with ERA had the longest delay: 45 weeks (13-104), whereas children with sJIA had the shortest delay: 9 weeks (4-21). The median diagnostic delay was not correlated with parents’ home distance from the diagnostic center. 59% and 57% of the patients were in remission and 57% at 1 and 2 years of follow-up, respectively.

The median delay between diagnosis and treatment onset was 3 weeks (IQR 0-34) for oral corticosteroids, 8 weeks (IQR 0.3- 30) for intra-articular corticosteroids, 9 weeks (IQR 0-52) for DMARDS and 52 weeks (IQR 19-147) for biologics.

The diagnostic delays for the whole cohort and for the different JIA subtypes are in line with the international literature. JIA with indolent symptoms as ERA had the longest diagnostic delay whereas sJIA associated to overt manifestations including fever had the shortest delay. In France and Switzerland, there are no guidelines about diagnostic delay but UK guidelines advocate a diagnostic delay inferior to 10 weeks. According to these guidelines, 68% of our patients exceed this delay, which is shlighly lower than previous studies. The absence of correlation with parents’ home distance from the diagnostic center is also described in other countries. Our remission rate at 1 year is higher than described in other studies but it decrease at 2 years. The treatment delay is longer in our cohort than in other studies.

**Conclusion:** The diagnostic delay and treatment delay in France and Switzerland are too long. Strong efforts have to be made to understand the underlying factors to suggest improvements in care pathway organization and practices by the elaboration of care course management guidelines. The JIR network permit to design a prospective study to identify these factors and improve the short-term prognosis of JIA, in the context of national and European rare disease health programs.

**Disclosure of Interest:** None Declared

### P66 Performance of disease activity measures in pediatric patients with enthesitis-related arthritis

Abstract withdrawn

### P67 Teaching adult rheumatology fellows to help young adult patients ‘stick the landing’ when transferring from pediatric to adult rheumatology care

#### Rebecca E. Sadun^1^, Gary R. Maslow^2^, Lisa G. Criscione-Schreiber^3^

##### ^1^Internal Medicine and Pediatrics, Duke University, Durham, NC, United States; ^2^Pediatrics and Psychiatry, Duke University, Durham, NC, United States; ^3^Internal Medicine, Duke University, Durham, NC, United States

###### **Correspondence:** Rebecca E. Sadun

**Introduction:** The transition from pediatric to adult healthcare is a vulnerable time for adolescents and young adults (AYA) with chronic conditions. Thie year the European League Against Rheumatism (EULAR) and the Pediatric Rheumatology European Society (PReS) jointly offered expert-opinions regarding transitional care of AYA with juvenile-onset rheumatic diseases and the American College of Rheumatology (ACR) developed a toolkit. For these guidelines to be implemented or for these resoures to be utilized, adult and pediatric rheumatologists need to understand their application. Nevertheless, there are no published curricula for teaching transition skills to rheumatologist-in-training.

**Objectives:** We sought to develop and assess the impact of a workshop designed to help adult rheumatology fellows learn key skills for providing effective transition care to transferring young adult patients.

**Methods:** A 1-hour skills-based workshop on transition and transfer best practices was developed alongside an objective standardized clinical examination (OSCE) station in which trainees were given 12 minutes to welcome a young adult with lupus – and her parent – to a first visit in an adult clinic and perform a full history. The OSCE evaluation rubric assessed five transition/transfer skills (skills 1-5) and one “control skill” (skill 6), on a Likert scale of 1-5, with 5 being the best performance. Adult rheumatology fellows (n = 19) from 5 institutions were evaluated with the OSCE; 12 were tested with the OSCE de novo, whereas 7 were tested with the OSCE after participating in the workshop. Unpaired, aggregated OSCE scores were complemented with unpaired pre- & post-survey data in which fellows reported their self-assessed level of preparation for 12 transition and transfer skills.

**Results:** Fellows’ self-assessed proficiency with 12 key transition/transfer skills increased significantly with participation in the workshop and accompanying OSCE, including fellows’ confidence in their ability to discuss the needs of the transferring patient with the pediatric rheumatologist (p < 0.01). In addition, OSCE performance (see Table 23) was greater in the group of fellows that participated in the workshop prior to the OSCE (“post-workshop” - average score of 4.3) than in the group that took the OSCE de novo (“pre-workshop” - average score of 3.3, p = 0.01). The skills demonstrating statistically significantly higher scores post-workshop were: skill 1) highlighting differences between adult and pediatric care and setting expectations (p < 0.01); skill 2) placing the patient in the primary role and utilizing the parent for corroboration (p < 0.05); and skill 4) performing a confidential adolescent social history. There was a trend towards improved performance for skill 3, assessing self-management skills, whereas there was no significant change in skill 5, assessing barriers to transition and medication adherence. Performance on skill 6, the control skill of assessing patient and parent understanding of the disease process, was lower in the group of fellows’ that participated in the workshop prior to being tested with the OSCE (p = 0.01).

**Table 23:** **(Abstract P67).** See text for description

	Skill 1	Skill 2	Skill 3	Skill 4	Skill 5	Total/Avg	Skill 6
Pre-workshop (n = 12)	3.5	4.3	3.8	2.8	2.3	16.7/3.3	3.5
Post-workshop (n = 7)	4.6	5.0	4.4	4.7	2.6	21.3/4.3	2.0
p-value	<0.01	<0.05	0.18	0.01	0.86	0.01	0.01

**Conclusion:** A brief educational intervention successfully increased adult rheumatology fellows’ perception of their proficiency with key transition/transfer skills. In addition, fellows who participated in the educational intervention had significantly higher scores on their OSCE performance. It is likely that the “control skill” score was lower in the post-workshop group owing to more time being spent on newly acquired peri-transfer skills: during the 12-minute OSCE scenario, discussing disease pathogenesis took a backseat to conversations about expectations in the adult care model, self-management skills, and goal setting. Making this curriculum available to all rheumatologists-in-training would likely improve the care young adult rheumatology patients receive when transferring from pediatric to adult rheumatology. A complementary curriculum for pediatric rheumatology fellows could further improve the care of AYA rheumatology patients.

**Disclosure of Interest:** None Declared

### P68 No radiographic damage after early aggressive treatment in juvenile idiopathic arthritis

#### Dieuwke Schreurs^1,2^, Willemieke van Braak^2^, Petra Hissink Muller^1^, Charlotte Nusman^2^

##### ^1^Pediatric Rheumatology, LUMC, Leiden, Netherlands; ^2^Radiology, AMC, Amsterdam, Netherlands

###### **Correspondence:** Dieuwke Schreurs

**Introduction:** Juvenile idiopathic arthritis (JIA) is characterized by chronic inflammation of the joints which can lead to structural bone damage.

**Objectives:** The primary objective of this study was to evaluate response of newly diagnosed JIA patients to an early aggressive treatment by conventional radiography. Secondary objective was to compare Poznanski and BoneXpert methods to evaluate presence and progression of radiological damage in wrists of JIA patients.

**Methods:** JIA patients participating in the BeSt for Kids study (NTR 1574) were eligible in case of wrist involvement at inclusion if conventional radiographs were available at baseline or within 6 months before/after study inclusion. Follow-up radiographs after 12-36 months were available for comparison. Radiographic bone damage as reflected by carpal length was assessed using Poznanski score. BoneXpert method was used to determine bone age and bone mineral density (BMD).

**Results:** Forty JIA (27 female) patients were evaluated for Poznanski score and BMD (mean age 7.2 ± 3.4 years), 26 patients (15 female) were evaluated for bone age (mean age 9.3 ± 2.2). At baseline mean Z-score of RM/M2 was 0.047. At follow-up mean Z-score changed to 0.055 (p = 0.937). Baseline and follow-up Z-scores were not different from a healthy population. Bone age did not significantly differ at baseline (Z-score 0.08) and follow-up (Z-score 0.25). At baseline BMD was significantly diminished compared to healthy controls (Z-score -0.71) and improved significantly (Z-score -0.44 (p = 0.032)).

**Conclusion:** In this cohort of JIA patients treated early and aggressively we have detected no radiographic damage in the wrist at baseline or follow up. BMD was significantly diminished at baseline but improved significantly after follow-up.

**Disclosure of Interest:** None Declared

### P69 The characteristic of the undifferentiated arthritis in juvenile idiopathic arthritis

#### Betul Sozeri^1^, Eylem Topaktas^1^, Duygu Kurtulus^2^

##### ^1^Pediatric Rheumatology, University of Health Sciences, Istanbul, Umraniye Training and Research Hospital, Istanbul, Turkey; ^2^Physical Medicine and Rehabilitation, University of Health Sciences, Istanbul, Umraniye Training and Research Hospital, Istanbul, Turkey

###### **Correspondence:** Betul Sozeri

**Introduction:** Juvenile idiopathic arthritis, JIA, is classified into seven categories; systemic-onset type, persistent and extended oligoarthritis, polyarthritis with rheumatoid factor negative, polyarthritis with rheumatoid factor positive, psoriatic arthritis, enthesitis-related arthritis and undifferentiated arthritis classified by the International League of Associations for Rheumatology. As each category of JIA has different features in clinical phenotypes, precise subtyping is required for research and management. Due to inclusion and exclusion criteria, some patients is ended up in the undifferentiated category. Undifferentiated arthritis includes patients who do not meet criteria for any category. Or, they meet the criteria for more than 1 category but with either a close relative who has psoriasis or the presence of RF. The prevalence of subtypes of JIA was reported; Oligoarticular JIA occurs most frequently (50% to 60% of cases), followed by polyarticular JIA. However, there is not enough information in the literature about the undifferentiated arthritis group.

**Objectives:** We aimed to analysis the characteristic findings of the undifferentiated arthritis group in JIA patients.

**Methods:** This was a retrospective longitudinal cohort study of patients with JIA at a tertiary care pediatric rheumatology clinic. Subjects were included if they were evaluated at our pediatric rheumatology clinic between June 2016 and May 2017, had a diagnosis of JIA according to ILAR criteria.

Demographics (sex, age at diagnosis, ANA, RF, HLA B27 status), clinical disease elements (active joint count, tender enthesis count) and current medication use at each visit were identified by querying the electronic medical record. All available visits were included in the analysis. All analyses were performed using SPSS 16.0

**Results:** During the study period there were 215 JIA patients evaluated at 640 visits. 180(84%) of patients had newly-diagnosed JIA.

There were 21 (84%) girls and 4 (16%) boys. Mean age at disease onset was 13.4 ± 2.2 years.The median duration of disease was 8 years.The median duration prior to treatment was 1 months. Among 25 patients with undifferentiated arthritis, 11 (48%) patients had enthesitis, 15 (60%) had sacroiliitis. Joints mainly affected in the undifferentiated arthritis group with were knees (16, 64%) and 2 patients were RF-positive and 7patients had positive ANA had undifferentiated arthritis. 6 patients (24%) of undifferentiated JIA patients used more than one DMARD.Methotrexate was the mainstay of treatment (52%), with an initial dose of 10–15 mg/m2/week and a maximum dose of 20– 25 mg/m2/week or 25 mg/week. Sulfasalazine was used in36 % of undifferentiated JIA patients. 2 of the patients received biologic agents.

**Conclusion:** Since distribution of the JIA category is different upon ethnicity, this study showed that percentage of undifferentiated JIA patients in our cohort was found higher reported from other studies in the literature. This is a single center, referral-based cohort study. As such, it might not represent the entire population of Turkey. In our conclusion, to clarify characteristics of undifferentiated JIA patients, need large cohort and multicenter study.

**Disclosure of Interest:** None Declared

## Juvenile idiopathic arthritis

### P70 Time of onset of ocular disease in 336 children with juvenile idiopathic arthritis-associated uveitis: evidence-based data to refine current ophthalmologic screening guidelines

#### Serena Calandra^1^, Valentina Muratore^2^, Valentina Ravaschio^1^, Gabriella Giancane^1^, Alessandra Alongi^1^, Angela Pistorio^1^, Alessandro Consolaro^1^, Angelo Ravelli^1^

##### ^1^Istituto Giannina Gaslini, Genova, Italy; ^2^Fondazione IRCCS Policlinico San Matteo, Pavia, Italy

###### **Correspondence:** Serena Calandra

**Introduction:** Uveitis is the most common form of extraarticular organ involvement in juvenile idiopathic arthritis (JIA). The onset of chronic anterior uveitis in JIA is insidious and often entirely asymptomatic. Children with JIA must be subjected to periodic eye examinations with slit lamp according to the different risk of developing this complication. The decision to undertake a screening program depends on the cost-benefit balance. To date, however, there is uncertainty about the optimal frequency of eye examinations. Although guidelines for ophthalmologic surveillance have been proposed, none of them is universally embraced.

**Objectives:** To evaluate the time of the occurrence of uveitis after the onset of arthritis in children with JIA

**Methods:** The clinical charts of 1425 patients with JIA by ILAR criteria followed at study centers from January 1987 to October 2016 and with a follow-up of at least 6 months after disease onset were reviewed to identify those who had uveitis. In all patients, the diagnosis of chronic anterior uveitis was confirmed by an ophthalmologist and was defined according to the Standardization of Uveitis Nomenclature Working Group criteria. Children with systemic arthritis, rheumatoid-factor positive polyarthritis and enthesitis-related arthritis were excluded. Patients who developed uveitis before the onset of arthritis were also excluded. Patients who developed uveitis at the same time of the arthritis onset were included. Through the construction of a cumulative frequency curve, we defined the risk of developing uveitis for each year of disease, starting with the onset of arthritis.

**Results:** A total of 336 patients (23.6%) had uveitis a median of 1.1 years after onset of arthritis. The cumulative frequency curve showed that 47.6% and 67.3% of patients developed uveitis in the first 1 and 2 years of illness, respectively, and that less than 5% of all instances of uveitis occurred after 7 years from the onset of arthritis (Table 24).

**Table 24:** **(Abstract P70).** Cumulative frequency of uveitis

Follow-up (years)	0 to 1	1 to 2	2 to 4	4 to 6	6 to 8	8 to 9	> 9
N.	160	66	51	31	15	6	7
Cumulative %	47.6	67.3	82.4	91.7	96.1	97.9	100

**Conclusion:** Our study shows that nearly half of JIA patients with uveitis developed this complication in the first year after onset of arthritis and around two-third within two years. Less than 5% of patients had uveitis after 7 years from disease onset. These findings underscore the need for tight ophthalmologic monitoring in the first 2 years of disease and support the recommendation of current guidelines to lengthen ophthalmologic screening after 7 years of disease without ocular involvement.

**Disclosure of Interest:** None Declared

### P71 An open-label extension study to assess the long-term safety and clinical benefit of etanercept in pediatric patients with extended oligo, enthesitis related, and psoriatic JIA: 6-year data from the clipper studies

#### Ivan Foeldvari^1^, Tamas Constantin^1^, Jelena Vojinovic^1^, Gerd Horneff^1^, Joke Dehoorne^1^, Gordana Susic^1^, Katarzyna Kobusinska^1^, Violeta Panaviene^1^, Zbigniew Zuber^1^, Valda Stanevica^1^, Vyacheslav Chasnyk^1^, Ronald Pedersen^2^, Jack Bukowski^2^, Tina Hinnershitz^2^, Bonnie Vlahos^2^, Alberto Martini^1^, Nicolino Ruperto^1^ on behalf of the Paediatric Rheumatology International Trials Organisation (PRINTO)

##### ^1^PRINTO, Genoa, Italy; ^2^Pfizer, Collegeville, PA, United States

###### **Correspondence:** Ivan Foeldvari

**Introduction:** A Phase 3b, open-label (OL), multicenter study (CLIPPER) has shown efficacy of etanercept (ETN) in pediatric patients (pts) with extended oligoarticular (eo) juvenile idiopathic arthritis (JIA), enthesitis-related arthritis (ERA), and psoriatic arthritis (PsA). CLIPPER2 is an ongoing OL extension study assessing long-term safety and clinical benefits of ETN in this population.

**Objectives:** Describe safety and clinical benefit of 6 yr of ETN treatment in CLIPPER (2 yr) and CLIPPER2 (4 yr) studies.

**Methods:** 127 pts with eoJIA (2–17 yr), ERA, or PsA (12–17 yr) who received ≥1 ETN dose (0.8 mg/kg QW [max, 50 mg]) in CLIPPER were eligible to enter CLIPPER2. Efficacy endpoints included proportions of pts achieving JIA ACR30/50/70/90/100 and Wallace/Juvenile Arthritis Disease Activity Score (JADAS) inactive disease/clinical remission criteria. Time to disease flare for pts who met predefined study criteria for withdrawing from ETN treatment was assessed by Kaplan-Meier (KM) analysis. Safety was assessed from CLIPPER baseline (BL) to month (m) 72.

**Results:** 109/127 (86%) pts entered CLIPPER2. At m72, 46/127 (36%) were taking ETN (39 [31%] ongoing, 7 [6%] restarted treatment), 44 (35%) had stopped ETN treatment, and 37 (29%) had discontinued the study. Improvements in disease activity seen in CLIPPER (m0–24) were largely maintained at m72 (Table; observed cases). Proportion of pts achieving JIA ACR30/50/70 responses at m24 (% = 99/98/93) was maintained to m72 (% = 96/96/88). Proportion of pts achieving JIA ACR90/100/Wallace inactive disease at m24 (% = 65/54/34) was maintained to m60 (% = 71/51/31) but afterwards decreased (% = 50/26/10 at m72). Sustained remission (≥12 continuous months) was achieved by 7/98 (7%) pts. 22/127 (17%) pts withdrew from ETN treatment due to low/inactive disease; 13/22 (59%) experienced disease flare (median time to flare [KM] = 190 days).

Most frequently reported treatment-emergent adverse events (TEAEs) were (no. events [N], events per 100 pt-yr [EP100PY]), headache (28, 5.34), arthralgia (24, 4.58), and pyrexia (20, 3.81) (Table 25). Number and frequency (N, EP100PY) of TEAEs (excluding infections/injection site reactions [ISR]) decreased over the 6-yr study period from 193, 173.81 in yr1 to 37, 61.34 in yr6. Most commonly reported treatment-emergent (TE) infections were (N, EP100PY) upper respiratory tract infection (66, 52.0), pharyngitis (58, 45.7), and gastroenteritis (31, 24.4). 1 case of malignancy (Hodgkin’s lymphoma) and no cases of active tuberculosis, demyelinating disorders, or deaths were reported.

**Table 25:** **(Abstract P71).** See text for description

Clinical Outcomes Mean (95% CI)					Safety Summary (to m72) N (EP100PY) unless otherwise stated				
	BL^1^(n = 127)	m24^1^(n = 109)	m48^2^(n = 64)	m72^2^(n = 47)		eoJIAEXP = 245.607 PY	ERAEXP = 158.888 PY	PsAEXP = 119.945 PY	TotalEXP = 524.441 PY
PtGA	4.96 (4.6,5.4)	0.97 (0.7,1.2)	1.32 (0.9,1.8)	1.29 (0.9,1.7)	TEAEs*	244 (99.35)	151 (95.04)	90 (75.03)	485 (92.48)
PGA	5.02 (4.7,5.3)	n = 108 0.62 (0.5,0.8)	n = 63 0.67 (0.5,0.9)	0.76 (0.5,1.0)	TE infections	351 (142.91)	93 (58.53)	117 (97.54)	561 (106.97)
CRP, mg/L	8.26 (5.7,10.8)	n = 103 2.76 (1.7,3.8)	2.85 (1.6,4.1)	n = 46 1.58 (1.2,2.0)	TEAEs* causing withdrawal, n (%)	5 (2.04)	8 (5.03)	0	13 (2.48)
JADAS 73 joints	n = 119 17.16 (15.9,18.4)	n = 102 2.28 (1.7,2.9)	n = 61 2.48 (1.8,3.1)	n = 29 3.12 (2.2,4.1)	TE infections causing withdrawal, n (%)	2 (0.81)	0	1 (0.83)	3 (0.57)
No. active joints	6.74 (5.9,7.6)	0.61 (0.2,1.0)	n = 63 0.49 (0.3,0.7)	n = 30 0.60 (0.2,1.0)	Serious TEAEs	11 (4.48)	17 (10.70)	4 (3.33)	32 (6.10)
No. active joints (LOM)	5.72 (5.0,6.5)	1.06 (0.5,1.6)	n = 63 1.02 (0.6,1.4)	n = 30 1.10 (0.5,1.8)	Serious TE infections	5 (2.04)	4 (2.52)	4 (3.33)	13 (2.48)
					Opportunistic infections^†^	0	1 (0.63)	1 (0.83)	2 (0.38)

Data from patients taking ETN (full analysis set)

^1^CLIPPER study

^2^CLIPPER2 study

*Excluding infections/ISR

^†^All herpes zoster

CRP, C-reactive protein; EXP, exposure to ETN; LOM, limitation of motion; PGA, physician’s global assessment; PtGA, patient/parent global assessment; PY, pt-yr

**Conclusion:** OL ETN treatment to m72 was effective and well tolerated in pts with eoJIA, ERA, and PsA. Majority of pts who withdrew from ETN treatment experienced disease flare. TEAE frequency decreased over time.

Trial registration identifying number: NCT00962741/NCT01421069

**Disclosure of Interest**: I. Foeldvari: None Declared, T. Constantin: None Declared, J. Vojinovic Speaker Bureau of: Abbvie, G. Horneff Grant/Research Support from: Pfizer, Abbvie, Roche, Novartis, J. Dehoorne Consultant for: Abbvie, Speaker Bureau of: Abbvie, G. Susic Grant/Research Support from: Pfizer, K. Kobusinska: None Declared, V. Panaviene: None Declared, Z. Zuber: None Declared, V. Stanevica Grant/Research Support from: Pfizer, Consultant for: Abbvie, V. Chasnyk: None Declared, R. Pedersen Shareholder of: Pfizer, Employee of: Pfizer, J. Bukowski Employee of: Pfizer, T. Hinnershitz Employee of: Pfizer, B. Vlahos Shareholder of: Pfizer, Employee of: Pfizer, A. Martini Consultant for: Abbvie, Boehringer, Novartis, R-Pharm, N. Ruperto Grant/Research Support from: BMS, Hoffman-La Roche, Janssen, Novartis, Pfizer, Sobi, Speaker Bureau of: Abbvie, Ablynx, AstraZeneca, Baxalta Biosimilars, Biogen Idec, Boehringer, Bristol Myers Squibb, Eli-Lilly, EMD Serono, Gilead Sciences, Janssen, Medimmune, Novartis, Pfizer, Rpharm, Roche, Sanofi

### P72 Successful treatment of methotrexate intolerance in juvenile idiopathic arthritis using eye movement desensitization and reprocessing (EMDR)

#### Lea Höfel^1^, Bruno Eppler^1^, Elisabeth Schnöbel-Müller^1^, Johannes-Peter Haas^2^, Boris Hügle^2^

##### ^1^Psychology, German Center for Pediatric and Adolescent Rheumatology, Garmisch-Partenkirchen, Germany; ^2^Rheumatology, German Center for Pediatric and Adolescent Rheumatology, Garmisch-Partenkirchen, Germany

###### **Correspondence:** Lea Höfel

**Introduction:** Methotrexate (MTX) is commonly used in the treatment of children with juvenile idiopathic arthritis (JIA). Frequently it is discontinued due to intolerance with anticipatory and associative gastrointestinal symptoms. Eye Movement Desensitization and Reprocessing (EMDR) is a therapy where non-processed and dysfunctional experiences and memories are reprocessed by intensive recall combined with eye movements. This leads to selective processing of the negative affect.

**Objectives:** Objective of this study was to investigate effectiveness of EMDR in the treatment of MTX intolerance in JIA patients, with the underlying hypothesis that intolerance occurs due to dysfunctional memories and expectations.

**Methods:** An open prospective study on consecutive JIA patients with MTX intolerance was performed. Intolerance was determined using the Methotrexate Intolerance Severity Score (MISS) questionnaire and health-related quality of live was determined using the PedsQL, at 3 time points: directly before and after treatment (after treatment: MISS only), and 4 months after treatment. Patients were treated using a standardized EMDR protocol with 8 sessions over a time period of 2 weeks. Changes in MISS and PedsQL were compared using descriptive and non-parametric methods.

**Results:** 14 patients with MTX intolerance (median MISS at inclusion: 13.5, range: 6-26) were included. Directly after treatment, all patients reported marked improvement of MTX intolerance symptoms (median MISS: 0.5, range: 0-3, p = 0.001). After 4 months, lasting reduction of MTX intolerance symptoms was observed (n = 5, median MISS: 5, range: 0-10, p = 0.068). However, 2/5 patients (40%) showed MISS >6. The health-related quality of life showed a trend towards improvement 4 months after treatment (n = 5, median pedsQL prior to treatment 84.4%, 4 months after treatment 92.4%, p = 0.46).

**Conclusion:** MTX intolerance in children with JIA can effectively be treated using an EMDR protocol, with lasting effect over 4 months. This intervention could potentially increase quality of life in affected patients and enable continued treatment with MTX.

**Disclosure of Interest:** None Declared

### P73 The comparison characteristic between patients with systemic onset of juvenile idiopathic arthritis with and without lung involvement.

#### Mikhail M. Kostik^1^, Eugenia Isupova^1^, Irina Chikova^1^, Margarita Dubko^1^, Vera Masalova^1^, Ludmila Snegireva^1^, Olga Kopchak^1,2^, Tatyana Kornishina^1^, Natalia Abramova^1^, Maria Rumyantseva^1^, Daria Dzhilkaidarova^1^, Maria Kaneva^1^, Olga Kalashnikova^1^, Vyacheslav Chasnyk^1^

##### ^1^Saint-Petersburg State Pediatric Medical University, Saint-Petersburg, Russian Federation; ^2^Kirov’s regional children’s hospital, Kirov, Russian Federation

###### **Correspondence:** Mikhail M. Kostik

**Introduction:** Juvenile idiopathic arthritis with systemic onset (soJIA) is the most striking form of JIA with combination of arthritis and systemic features. The acute lung involvement (ALI) is not frequent manifestation of soJIA but often influence on disease course, outcomes and treatment. The mechanisms of lung involvement are still unclear.

**Objectives:** The aim of our study was to compare clinical and laboratorial features of patients with soJIA with and without ALI.

**Methods:** In the study were included retrospective data of 82 children with soJIA. ALI was described if patient had at least one: dyspnoe, shortness of breath, signs of respiratory distress syndrome, interstitial lung disease, pulmonary arterial hypertension.

**Results:** ALI was detected in the onset of soJIA in 18 (22.0%) of patients. Patients with ALI had more severe soJIA disease course (data in Table 26).

**Table 26:** **(Abstract 73).** See text for description

Parameter	ALI (n = 18)	W\o ALI (n = 67)	p
Hemoglobin, g/l	86,5 (80,0; 108,0)	105,0 (94,0; 116,0)	0,003
Platelets, x10^9^/l	201,0 (95,0; 492,0)	454,0 (361,0; 588,0)	0,006
CRP, mg/l	104,8 (37,5; 154,0)	47 (18,0; 104,0)	0,07
LDH, U/l	1042,0 (543,0; 1230,0)	516,0 (395,0; 676,0)	0,012
Ferritin, ng/ml	1759,0 (1144,0; 11300,0)	321,0 (139,0; 1278,0)	0,0009
Prothrombin, %	71,1 (63,0; 79,0)	91,0 (79,0; 98,0)	0,0009
Fibrinogen, g/l	1,8 (0,8; 4,0)	4,7 (3,2; 5,7)	0,012
Total protein, g/l	64,6 (55,0; 70,3)	71,2 (67,0; 77,0)	0,002
Albumin, %	24,1 (23,0; 25,6)	39,0 (31,8; 43,2)	0,00001

Patients with ALI more frequently had MAS (p = 0,0006), pericarditis (p = 0,0006), transient proteiunuria, attributed to MAS (p = 0,015), concomitant sepsis (p = 0,005) and morphological evidence of hemophagocytosis in bone marrow (p = 0,02).

The main predictors of development ALI were presence of macrophage activation syndrome (OR = 6.6 [2.1; 21.0], p = 0.0006), bone marrow hemophagocytosis (OR = 13.9 [0.7; 269.2], p = 0.02), heart involvement (OR = 6.4 [2.1; 19.7], p = 0.0006), kidney involvement (transient proteinuria) (OR = 6.1 [1.2; 30.3], p = 0.0015), evidence of concomitant sepsis (OR = 21.2 [1.0; 466.0], p = 0.005), albumin ≤ 25.6 g/l (OR = 73.3 [10.9; 495.0], p = 0.0000001), AST > 37.3 U/l (OR = 5.3 [1.4; 20.2], p = 0.008), CRP > 125.0 g/l (OR = 5.6 [1.7; 18.6], p = 0.003), ferritin > 690 ng/ml (OR = 22.7 [2.7; 191.4], p = 0.0002), hemoglobin ≤ 89 g/l (OR = 7.1 [2.3; 22.6], p = 0.0001), LDH > 882 U/l (OR = 4.8 [1.4; 16.4], p = 0.002), platelets ≤ 211x10^9^/l (OR = 10.1 [3.1; 32.9], p = 0.00002), prothrombin ≤ 74.6% (OR = 12.6 [2.8; 57.3], p = 0.0003), total protein ≤ 65.0 g/l (OR = 8.7 [2.7; 27.8], p = 0.00008).

**Conclusion:** The identified predictors of ALI in soJIA have to check to evaluation of prognosis and choosing treatment plans.

**Disclosure of Interest:** None Declared

### P74 Decreasing prevalence of uveitis in children with juvenile idiopathic arthritis seen over a 30-year period

#### Valentina Ravaschio^1^, Valentina Muratore^2^, Serena Calandra^1^, Gabriella Giancane^1^, Alessandra Alongi^1^, Angela Pistorio^1^, Alessandro Consolaro^1^, Benedetta Schiappapietra^1^, Angelo Ravelli^1^

##### ^1^Istituto Giannina Gaslini, Genova, Italy; ^2^Fondazione IRCCS Policlinico San Matteo, Pavia, Italy

###### **Correspondence:** Valentina Ravaschio

**Introduction:** Uveitis is the most common extra-articular manifestation of juvenile idiopathic arthritis (JIA). In recent years, there has been a major advance in the management of both JIA and of uveitis. Non-controlled studies have suggested that some medications, particularly methotrexate and anti-TNF antibodies, are potentially capable to prevent the occurrence of uveitis. However, whether the recent therapeutic progress had led to a reduction in the prevalence of uveitis is unknown.

**Objectives:** To investigate the temporal trend in the prevalence of uveitis in JIA patients seen over a 30-year period.

**Methods:** A retrospective analysis of the medical records of all children with JIA by ILAR criteria who were seen at authors’ units between January 1987 to October 2016 was carried out. The prevalence of uveitis in patients with onset of JIA in different time periods (before 1997, between 1997 and 2005 and after 2005) was evaluated. Patients without uveitis who had a disease duration of less than 6 months were excluded.

**Results:** A total of 1.425 patients with JIA, 336 (23.6%) of whom had developed uveitis were identified. The prevalence of uveitis was 60/211 (28.4%) among patients with onset before 1997, 149/602 (24.8%) among patients with onset between 1997 and 2005, and 127/612 (20,8%) among patients with onset after 2005. The follow-up of patients seen in the three time periods was comparable (Table 27).

**Table 27:** **(Abstract P74).** See text for description

	Before 1997 (n = 211)	1997-2005 (n = 602)	After 2005 (n = 612)
number of Uveitis	60 (28,4%)	149 (24,8%)	127 (20,8%)
Age at onset <3	96 (45,5%)	273 (45,3%)	302 (49,3%)
Age at onset 3-7	80 (37,9%)	203 (33,7%)	169 (27,6%)
Age at onset >7	35 (16,6%)	126 (20,9)	141 (23,0%)
Extended oligoarthritis	35 (16,6%)	84 (14,0%)	97 (15,8%)
Persistent oligoarthritis	117 (55,5%)	317 (52,7%)	348 (56,9%)
FR-negative poliarthritis	35 (16,6%)	139 (23,1%)	138 (22,5%)
Psoriatic arthritis	11 (5,2%)	21 (3,5%)	10 (1,6%)
Undifferentiated arthritis	13 (6,2%)	41 (6,8%)	19 (3,1%)

**Conclusion:** Our study shows a decrease in the prevalence of uveitis over time, which might be, at least partially, related to the increased tendency toward earlier use of methotrexate and the introduction of biologic agents. This finding should to be confirmed in patient series seen in other pediatric rheumatology centers.

**Disclosure of Interest:** None Declared

### P75 Comparison of patients with familial Mediterranean fever accompanied with sacroiliitis and patients with juvenile spondyloarthropathy

#### Hafize E. Sönmez, Ezgi D. Batu, Selcan Demir, Yelda Bilginer, Seza Özen

##### Department of Pediatrics, Division of Rheumatology, HACETTEPE UNIVERSITY FACULTY OF MEDICINE, Ankara, Turkey

###### **Correspondence:** Hafize E. Sönmez

**Introduction:** Familial Mediterranean fever (FMF) is the most common autoinflammatory disease manifesting with self-limited recurrent febrile attacks and polyserositis. Acute recurrent monoarthritis is the most common form of musculoskeletal involvement in FMF; however, up to 5% of FMF patients may develop chronic joint diseases including sacroileitis. It is difficult to distinguish whether sacroiliitis is a musculoskeletal finding of FMF or whether this is the coexistence of two diseases; FMF and SpA.

**Objectives:** In this study, we aimed to evaluate FMF patients with sacroiliitis, and compare their features with juvenile spondyloarthropathy (SpA) patients all of whom had sacroiliitis.

**Methods:** 15 pediatric FMF patients with sacroiliitis and 30 patients with juvenile SpA followed between 2014-2016 at the Department of Pediatric Rheumatology at Hacettepe University, Ankara, were included in the study.

**Results:** The median (min-max) age at diagnosis of sacroiliitis was 11 (7-15) for FMF + sacroiliitis, and 11.5 (7-16) years for juvenile SpA patients. All patients suffered from hip pain and morning stiffness. Only two FMF + sacroiliitis patients had enthesitis, while nearly half of juvenile SpA patients (46.7%) had enthesitis. Four FMF patients suffered from lower back pain, although none of them had spinal involvement. On the other hand, approximately one third of juvenile SpA patients had spinal involvement. The median white blood cell count, erythrocyte sedimentation rate, and C reactive protein values in FMF + sacroiliitis patients were higher (10.1x10^3^/mm^3^ vs 7.8x10^3^/mm^3^, p = 0.002; 41 vs 28 mm/h, p < 0.001; 4.6 vs 1.3 mg/dl, p < 0.001; respectively) than juvenile SpA patients. HLA B27 positivity was more common in juvenile SpA than FMF + sacroiliitis patients (86.6% vs 26.7%, respectively, p = 0.001). The most common *MEFV (MEditerranean FeVer)* mutation was M694V in FMF patients. All juvenile SpA patients but one were negative for *MEFV* mutations. One juvenile SpA patient was heterozygous for E148Q.

**Conclusion:** We demonstrated that pediatric patients with FMF + sacroiliitis showed different characteristics (higher inflammatory markers, less frequent spinal and enthesitis involvement and HLA-B27 positivity) from patients with juvenile SpA. The jury is out to decide whether FMF is a triggering factor for SpA or whether this is a feature of FMF per se.

**Disclosure of Interest:** None Declared

### P76 Epidemiology, clinical manifestations and treatment of systemic-onset juvenile idiopathic arthritis (SOJIA): a multicentric study through the international platform JIRcohorte.

#### Katerina Theodoropoulou^1^, Alexandre Belot^2^, Véronique Hentgen^3^, Isabelle Kone-Paut^4^, Carine Wouters^5^, Kenza Bouayed^6^, Elvira Cannizzaro^7^, Aurélia Carbasse^8^, Etienne Merlin^9^, Claire Ballot^10^, Daniela Kaiser^11^, Pascal Pillet, Andreas Woerner ^13^, Walter Bär^14^, Sylvaine Poignant^15^, Véronique Despert^16^, Caroline Freychet^17^, Gerald Berthet^18^, Laetitia Higel^19^, Tu Tran^20^, Federica Vanoni^21^, Sophie Georgin-Lavialle^22^, Michaël Hofer^1^

##### ^1^Pediatric Rheumatology, CHUV, Lausanne, Switzerland; ^2^Hospices Civils de Lyon, Lyon, France; ^3^CH de Versailles - Hôpital André Mignot, Paris, France; ^4^Centre Hospitalier Universitaire Kremlin-Bicêtre, Paris, France ; ^5^UZ Leuven, Leuven, Netherlands ; ^6^Hôpital d’Enfants Ebnou Rochd Casablanca, Casablanca, Morocco; ^7^Kinderspital Zürich, Zürich, Switzerland; ^8^CHU Arnaud de Villeneuve Montpellier, Montpelier, France; ^9^Centre Hospitalier Universitaire de Clermont-Ferrand, Clermont-Ferrand, France; ^10^CHRU Besançon, Besançon, France; ^11^Luzerner Kantonspital, Lucerne, Switzerland; ^12^CHU de Bordeaux, Bordeaux, France; ^13^Universitäts-Kinderspital beider Basel, Basel, Switzerland; ^14^Kantonspital Graubünden, Graubünden, Switzerland; ^15^CHU Nantes, Nantes, France; ^16^CHU Rennes, Rennes, France; ^17^CHU St-Etienne, St-Etienne, France; ^18^Kantonspital Aarau, Aarau, Switzerland; ^19^Hôpital Hautepierre Strasbourg, Strasbourg, France; ^20^CHU Nîmes, Nîmes, France; ^21^L’Ospedale Regionale di Bellinzona e Valli, Bellinzona, Switzerland; ^22^Hôpital TENON, Paris, France

###### **Correspondence:** Katerina Theodoropoulou

**Introduction:** Systemic-onset juvenile idiopathic arthritis (SoJIA) is a rare paediatric rheumatic condition with unclear pathogenesis, challenging diagnosis and treatment.

**Objectives:** The aim of our study is to describe the epidemiology, clinical presentation and biologic treatment of SoJIA patients registered in the JIRcohorte.

**Methods:** This is a multicentre, observational, retrospective and inception cohort study, through an international platform: JIRcohorte. Patients with SoJIA, diagnosed and followed in one of the participating centres in the JIRcohorte project in Switzerland, France, Belgium and Morocco are enrolled in the registry.

**Results:** 212 patients with SoJIA have been included; 119 were girls with a female: male ratio of 1.3:1. The median age at diagnosis was 5.4 years and the median diagnostic delay was 40 days. Data for initial systemic manifestations were available in 57 patients: 96% presented with typical fever (55/57), 56% with rash (32/57), 32% with lymphadenopathy (18/57), 21% with splenomegaly (12/57), 21% with hepatomegaly (12/57), and 16% with serositis (9/57). Data for joint involvement were available in 46 patients: arthritis was reported in only 61% of them during the first year of disease evolution, with a poly-articular predominance (61%). 169 out of 212 patients (80%) had a biologic treatment at least once during their disease course. Before 2008, anti-TNFa agents were more frequently used (69%), followed by anti-IL-1 agents (30%), with a very few patients receiving anti-IL-6 agent (1%). Since 2008, anti-IL-1 agents became the most common biologic treatment (46%), followed by tocilizumab (36%), with fewer patients under anti-TNFa agents (18%). Higher rate of infectious adverse effects was reported with Tocilizumab, while Anakinra was associated with skin and administration site effects.

**Conclusion:** We describe here the main epidemiologic and clinical characteristics, as well as the treatment of patients with SoJIA included in our international JIRcohorte platform. Biologic therapies, in particular anti-IL1 agents, are widely used in the treatment of this disease throughout the last years. Further prospective data analysis is required to evaluate the long-term efficacy and the safety of these treatments.

**Disclosure of Interest:** None Declared

### P77 Autoinflammatory mechanisms via NF-KB and caspase-1/IL-1 inflammasome pathways in ana negative juvenile idiopathic arthritis

#### Andrea Zacarias^1^, Anna Mensa^2^, Estibaliz Iglesias^1^, Rosa Bou^1^, Joan Calzada^1^, Jordi Anton^1^, Juan Ignacio arostegui^2^, Juan Manuel Mosquera^1^, Violeta Bittermann^1^

##### ^1^Rheumatology, Hospital Sant Joan de Deu, Barcelona, Spain; ^2^Inmunology, Hospital Clinic, Barcelona, Spain

###### **Correspondence:** Andrea Zacarias

**Introduction:** Classification criteria of Juvenile Idiopathic Arthritis (JIA) are clinical and of exclusion. Autoinflammatory Inherited Diseases (AID) are innate immune system disorders with clinical similarity with JIA that have been considered in differential diagnosis of this disease. Patients with AID previously wrongly diagnosis as JIA has been described

**Objectives:** To evaluate presence of variations on genes implicated in AID in a group of JIA patients attended at our center between January 2000-2015. To compare clinical and laboratory data between patients with and without variations on these genes.

**Methods:** Transversal (January 2000-2014) and prospective (January 2014-2015) study. Clinical, demographics and laboratory (erythrocyte sedimentation rate (ESR), C reactive protein (CRP), ANA, blood cell count) data were collected. *Next-generation sequencing* test of *MEFV*, *TNFRSF1A*, *MVK*, *NLRP3*, *NOD2* and *PSTPIP1* were done.

**Results:** One hundred and one patients were included (76 girls). 61 oligoarticular JIA, 20 rheumatoid factor (RF) negative polyarticular JIA, 19 extended oligoarticular JIA and 1 RF positive polyarticular JIA. Fifty patients were ANA positive. At least one variation was identified in 26.7% of our patients. Accumulated probability of having some variation in these genes in our JIA series was statistical significant greater than healthy Iberian population (20 vs 8.2% respectively, p = 0.01). Table 28 describes identified genetic variations. Variations in all searched genes were detected except for *MVK*. Oligoarticular extended JIA was the more affected subtype (47.4%, p = 0.082). We did not find differences according to ANA.

**Table 28:** **(Abstract P77).** See text for description

Genes	MEFV	MEFV	MEFV	TNFRSF1A	NLRP3	NOD2	NOD2	NOD2	PSTPIP1
Mutation	E148Q	I591T	M694I	P46L	V198M	D925G	N289S	E778K	G258a
JIA Patients	7/101 6,9%	4/101 3,9%	3/101 2,9%	1/101 0,9%	4/101 3,9%	1/101 0,9%	4/101 3,9%	1/101 0,9%	1/101 0,9%
Iberian Population	0/107 0%	2/107 1,8%	18/69.706 0,03%	0/107 0%	1/107 1,8%	1/107 1,8%	1/107 1,8%	0/107 0%	1/6498 0,01%

Based on the presence of a genetic variation we did not find differences in age at onset disease, presence of extra-articular symptoms, laboratory parameters at diagnosis.

**Conclusion:** Variations on all AID studied genes were detected except for *MVK*. We found greater prevalence of genetic variations in JIA patients than described in healthy Iberian population. We did not find differences according ANA. We did not find clinical nor laboratory predictors of presence of a genetic variation

**Disclosure of Interest:** None Declared

## Juvenile idiopathic arthritis (JIA) (oligo, poly, psoriatic)

### P78 The lull before the TMJ storm: identifying a window of opportunity for early identification of TMJ arthropathy

#### Clare Marie Adams^1^, Clarissa Pilkington^2^

##### ^1^Rheumatology, Great Ormond Street Hospital for Children, London, United Kingdom; ^2^Consultant Paediatric Rheumatologist, Great Ormond Street Hospital, London, United Kingdom

###### **Correspondence:** Clare Marie Adams

**Introduction:** Temporomandibular joint (TMJ) arthritis is common in children with Juvenile Idiopathc Arthritis (JIA); it confers significant functional and psychological morbidity to young people suffering with pain, functional impairment and facial asymmetry, as a result of TMJ destruction. Gadolinium-enhanced MRI is the gold-standard imaging modality for detection of synovitis, however early recognition of TMJ arthritis is challenging: clinical assessment is insensitive and early findings on MRI overlap with non-inflammatory synovial enhancement of normal children. With the expense and relative scarcity of MRI, coupled with challenging interpretation of results in early disease, there is an absence of international consensus with regard to screening in TMJ arthritis. Reducing the burden of deforming TMJ disease in young people would undoubtedly be aided by effective early detection of joint involvement.

**Objectives:** This observational study aims to understand the characteristics of the JIA patient cohort undergoing TMJ MRI in our centre. It aims to identify opportunities for improved clinical practice with a view to early detection of TMJ arthritis, and to contribute to discussion regarding development of consensus based local guidance for TMJ surveillance in children with JIA.

**Methods:** The electronic clinical records of 30 consecutive patients undergoing TMJ MRI between March 2014 and April 2016 were retrospectively reviewed. They were split into two age groups, 0-4 years (n = 15) and 5-12 years (n = 15). Baseline characteristics including age, sex, JIA subtype, time lag between JIA diagnosis and MRI-TMJ involvement, indications for MRI, MRI reports and whether the MRI changed clinical management were assessed. All TMJ MRIs (n = 56) performed on these 30 patients were assessed.

**Results:** Children developed TMJ involvement aged 8-10 years old, regardless of age of onset of JIA. Following diagnosis of JIA, TMJ involvement was identified after 6.75 years (median, range 1.25–9.33 years. Mean 5.92 years) in 0-4 age group, and 1 year (median, range 0-9year. Mean 1.83 years) in 5-12 group. 40% (6 children, aged 7-11years) in 5-12 group had TMJ involvement at diagnosis. Most common indications for MRI were pain and restriction (0-4 group), with deviation and asymmetry (5-12 group). The 5-12 group had more bony erosions on MRI report (13/15) and more unilateral disease compared to 0-4 group. The burden of TMJ disease occurs predominantly over the age of 5 (28/30). MRI changed clinical management (18/30).

**Conclusion:** Irrespective of age of onset of JIA, children developed TMJ involvement between 8-10 years of age. This indentifies a group upon which to focus targetted MRI surveillance with the aim of detecting early TMJ disease. This is a small study, but it contributes to the ongoing discussions regarding formation of pragmatic consensus guidance in TMJ surveillance in asymptomatic children with JIA. Further study is required.

**Disclosure of Interest:** None Declared

### P79 Burden of disease in the pediatric rheumatology clinic at Alain Hospital: the UAE experience

#### Amal A. Alhosani

##### child health institute, Alain Hospital, alain, abu dhabi, United Arab Emirates

**Introduction:** Introduction: The subspeciality of pediatric rheumatology is the newest discipline established at child health institution in Alain hospital, in 2013. The service provides tertiary care for children with rheumatic diseases from all over United Arab Emirates. Currently there are no data that describe the epidemiology of rheumatic diseases in children.

**Objectives:** The aim of this study is to describe the demographics and diagnostic classification of children with rheumatic conditions followed up in the clinic.

**Methods:** Methods: a retrospective cohort was conducted. All the files from August 2013 to July April 2017 were reviewed. Case description and diagnosis were captured. Standardized description of diagnosis was used. All patients were screened for uveitis by ophthalmologist using slit lamp examination

**Results:** Results: rheumatic conditions were diagnosed in 130 subject. JIA was the most frequently encountered rheumatic condition 44% (n = 58), followed by FMF 10% (n = 14) and SLE 9% (n = 12). Among JIA group oligoarticular JIA was the most common subtype 60% (n = 35), 10% (n = 10) of the cases were complicated by anterior uveitis. The remaining 37% of patients had a variety of other conditions. MTX was used to treat 38% (n = 51) of the cases. Biologics were started in 20% (n = 25) cases.

**Conclusion:** Conclusion: this is the first cohort that describes the epidemiology of rheumatic conditions among UAE children, it highlights the rheumatologist conditions and their frequencies among pediatric population, however continuos prospective data is needed to confirm our initial observation.

**Disclosure of Interest:** None Declared

### P80 Investigating longitudinal course of ankle involvement in relation to treatment in children with JIA using a multistate markov model approach

#### Alessandra Alongi^1,2^, Alessandro Consolaro^2^, Angelo Ravelli^2^

##### ^1^Università degli Studi di Genova; ^2^Instituto Giannina Gaslini, Genova, Italy

###### **Correspondence:** Alessandra Alongi

**Introduction:** The ankle-foot complex (AFC) is a commonly involved joint site in children with Juvenile Idiopathic Arthritis (JIA) that requires special attention from clinicians due to the complexity of its anatomical structure and the recognized association with a more severe disease course. Some patients experience recurrent, treatment refractory involvement of this region, posing unique challenges for therapeutic management. Little information concerning the longitudinal course of AFC arthritis in relation to treatment and its predictors is available to date to guide treatment strategies for JIA patients.

**Objectives:** To describe trajectories of AFC involvement in a cohort of JIA patients in relation with received treatments, and identify subgroups of patients at risk of recurrent or treatment resistant AFC arthritis over time.

**Methods:** Records of oligoarticular and polyarticular RF-negative JIA patients followed in a single tertiary center and diagnosed between 2005 and 2012 were retrospectively evaluated for the occurrence of clinically assessed tibiotalar, subtalar and tarsal arthritis. 143 patients with at least one episode of AFC involvement in the first 5 years from disease onset and complete records were identified. Data collected during a total of 2288 visits - at baseline and at intervals of 3-4 months during the first 5 years of follow-up - were included in the analysis. Specifically, time-varying variables such as disease status, patterns of joint involvement, and current treatments including intra-articular steroid injections were analyzed. Probabilities of AFC arthritis and of being treated with methotrexate (MTX), etanercept (ETN), other TNFa inhibitors or systemic steroids at each evaluation from the second to the fifth year of follow-up were evaluated through multistate Markov modeling, which allows detecting transitions among different clinical states. The relationship between the identified states and clinical-demographical features at onset and in the first year of follow-up was then assessed through regression modeling.

**Results:** The model identified 6 latent states, defined by the combination of AFC activity and treatment status at each time point: absence of AFC involvement in patients currently off medication (State 1, 906 observations); absence of AFC involvement while receiving MTX treatment (State 2, 622 observations); low probability of AFC arthritis and during treatment with both MTX and ETN (State 3, 316 observations); AFC arthritis in patients currently off medication (State 4, 158 observations); State 5 (197 observations) and State 6 (89 observations) are associated with AFC arthritis during MTX therapy but with high and low probability respectively. After adjusting for active joints count, the number of relapses involving the tibiotalar joint during the first year from onset predicted the transition from State 1 to State 4 - relapse off therapy - and were inversely correlated with probability of transition from State 3 to State 1, namely maintaining remission after therapy withdraw (p = 0.014 and p = 0.035, respectively). Tarsal joints arthritis in the first year of disease was negatively associated with transition from State 5 to State 2, namely AFC response to MTX therapy (p = 0.030). A negative correlation between the occurrence of subtalar involvement in the first year of disease and State 1 was also observed (p = 0.021).

**Conclusion:** Analysis of longitudinal data provides insight for the identification of patients at risk of recurrent AFC involvement and their clinical management. Further studies are needed to assess the potential value of such information in guiding therapeutic strategies for JIA patients.

**Disclosure of Interest:** None Declared

### P81 Novel mutations in PRG4 gene in one Greek family with camptodactyly-arthropathy-coxa vara-pericarditis (CACP) syndrome

#### Sorina Boiu^1^, Mouna Barat^2^, Isabelle Touitou^3^, David Geneviève^4^, Vasileios Kontogeorgakos^5^, Erato Atsali^6^, Dimitrios Boumpas^7^, Vana Papaevangelou^8^

##### ^1^Pediatric Rheumatology Unit, Third Department of Pediatrics, "Attikon" University Hospital, National and Kapodistrian University of Athens, Athens, Greece, ^2^Laboratoire de Génétique, Hôpital Arnaud de Villeneuve, CHRU Montpellier Montpellier, France, ^3^Laboratoire de Génétique, Hôpital Arnaud de Villeneuve, CHRU Montpellier, Montpellier, France ^4^Département de Génétique Médicale, Maladies Rares et Médecine Personnalisée, Unité Inserm U1183, CHU Montpellier, Université Montpellier, Montpellier, France, ^5^First Department of Orthopedics, “Attikon” University Hospital, National and Kapodistrian University of Athens, Athens, Greece ^6^Pediatric Rheumatology Unit, Third Department of Pediatrics, “Attikon” University Hospital, National and Kapodistrian University of Athens, Athens, Greece ^7^Joint Rheumatology Program, Fourth Department of Medicine, “Attikon” University Hospital, National and Kapodistrian University of Athens, Athens, Greece ^8^Third Department of Pediatrics, “Attikon” University Hospital, National and Kapodistrian University of Athens, Athens, Greece

###### **Correspondence:** Sorina Boiu

**Introduction:** Camptodactyly-Arthropathy-Coxavara-Pericarditis (CACP) syndrome is a rare autosomal recessive disorder caused by mutations in the PRG4 (proteoglycan 4) gene located on chromosome 1q31.1 (OMIM:*604283). Hallmarks of the syndrome include congenital or early-onset camptodactyly and arthropathy with synovial hyperplasia, progressive coxa vara deformity and non-inflammatory pericarditis. Patients affected by CACP syndrome lack the glycoprotein lubricin, a major lubricant expressed mainly in joints and in pericardial and pleural cavities. Features of CACP syndrome mimic juvenile idiopathic arthritis (JIA) and children with CACP are often misdiagnosed as JIA. To date, twenty-two homozygous mutations and 1 case of CACP syndrome resulting from uniparental disomy of chromosome 1 have been reported in different ethnic populations, none from Greece.

**Objectives:** To report the mutations in the PRG4 gene in 2 siblings with CACP syndrome in one family from Greece.

**Methods:** A 6 years old boy suffered from congenital camptodactyly and progressive non-inflammatory arthropathy from the age of 2 years (knees, ankles, elbows, wrists, and cervical spine). Radiographs, ultrasound and MR images revealed features of CACP syndrome. He was diagnosed with juvenile idiopathic arthritis at the age of 3 years and received corticosteroids, methotrexate, and anti-TNF treatment without efficacy. The patient's younger brother, aged 2 years, had a similar history, albeit more severe, starting at the age of 2 months. The family consulted in our Clinic for a second opinion at the age of 6 years. Molecular genetic studies were done for all coding exons and the exon-intron boundaries of the PRG4 gene. NGS was performed on hybridization-captured region of interest. All variants were confirmed by Sanger sequencing.

**Results:** The 2 siblings were found to be compound heterozygotes for c.[957del];[3254_3260del] p.[(Asp320Ilefs*2)];[(Ser1085*)]. These rearrangements lead to a shift of the reading frame and the appearance of a premature stop codon. This type of mutations is likely to lead to the synthesis of a non-functional truncated protein.

**Conclusion:** To our knowledge, this is the first report of PRG4 mutations from Greece as well as the first case of CACP syndrome phenotype caused by novel heterozygous mutations. The presence of arthropathy with camptodactyly, especially if familial, should alert the clinician to the possibility of CACP syndrome and avoid misdiagnosis of JIA and unnecessary treatment with potentially harmful drugs.

**Disclosure of Interest:** None Declared

### P82 Dental anxiety and oral health in juvenile idiopathic arthritis

#### Christina Boros^1^, Jason Armfiled^2^

##### ^1^Discipline of Paediatrics, University of Adelaide, Adelaide, Australia; ^2^Australian Research Centre for Population Oral Health, University of Adelaide, Adelaide, Australia

###### **Correspondence:** Christina Boros

**Introduction:** Previous studies suggest that children with Juvenile Idiopathic Arthritis (JIA) have poorer oral health compared to their healthy peers despite adequate knowledge of appropriate dental care. We hypothesised that this may be due to dental anxiety and that there may be specific dental and/or disease related factors which predict dental fear in this population

**Objectives:** We aimed to determine the prevalence and predictors of dental fear in a single centre population of JIA patients.

**Methods:** JIA patients aged 5–18 years attending the rheumatology clinic at the Women’s and Children’s Hospital in Adelaide, South Australia completed a questionnaire regarding dental visit frequency, past dental experiences, oral health behaviour, self-rated dental health, assessment of dental anxiety using the Modified Child Dental Anxiety Response Scale (MCDAS_f_), their cognitive vulnerability-related perceptions of dental treatment-related events and the degree of pain and reduced movement in upper limb and temporo-mandibular joints (TMJs). Clinical data relating to JIA subtype, medications, arthritis activity in upper limbs and TMJs and JADAS score on the day of questionnaire completion were also documented.

**Results:** Ninety four patients, (24 boys, 25.5%) participated. Average age was 12.3 years (SD = 4.3), average disease duration 5.6 years (SD = 4.3) and median JADAS score 1 (range 0–17.2). Thirty eight (40.4%) had oligoarticular and 33 (35.1%) had polyarticular onset disease. Nine (9.6%) had examination findings of TMJ involvement and four (4.3%) had active arthritis in upper limb joints of the dominant hand. Mean MCDAS_f_ score (Cronbach’s alpha = 0.93) was 2.97 with 50.5% of children having a score above the cut point for classifiable high dental fear. While dental fear was not related to having received any specific previous dental treatment, those who had not received any treatment had higher dental fear than those who had experienced at least one treatment (3.61 cf 2.87, *p* = 0.002). There was no statistically significant association between having had a previously unpleasant dental experience and dental fear (*F* = 2.06, *p* = 0.112). However, dental fear was associated with anxiety related to receiving blood tests (*r* = 0.32, *p* = 0.002) and injectable medications (*r* = 0.34, *p* = 0.001). Vulnerability-related cognitions, ie perceptions of the dental visit as dangerous, disgusting, uncontrollable and unpredictable were significantly correlated with dental fear (*r* = 0.44, *p* < 0.001) and in a multivariable stepwise regression model accounted for a statistically significant amount of variance in dental fear (adjusted *R* ^2^ = 31.1%) after controlling for participant demographics, medically-related fears, and having received a past dental treatment.

**Conclusion:** In a single-centre cohort of JIA, approximately 50% reported high levels of dental fear associated with procedures related to their arthritis care rather than a previously unpleasant dental experience. Those who had not yet visited a dentist had higher dental fear with vulnerability-related cognitions of the dental visit contributing most significantly to dental fear. These results may explain poorer dental health reported in JIA patients.

**Disclosure of Interest:** None Declared

### P83 Characterization of a Colombian cohort with juvenile idiopathic arthritis: a single multicenter experience

#### Miguel Cañola^1^, Maria A. Alzate^2^, Deicy Hernandez-Parra^2^, Diana Echeverry^2^, Juan C. Uribe-Salazar^3^, Javier Alvarez^4^, Lorena Londoño^1^, Paula Ortiz-Salazar^2^, Ricardo Pineda^2^

##### ^1^Rheumatology division, Artmedica, IPS, Medellin, Colombia; ^2^Clinical information group, Artmedica, IPS, Medellin, Colombia; ^3^School of Statistics, Faculty of Sciences, National University of Colombia, Medellin, Colombia; ^4^Medical student, Universidad CES, Medellin, Colombia

###### **Correspondence:** Miguel Cañola

**Introduction:** Juvenile idiopathic arthritis (JIA) is the most common chronic rheumatologic disease in children (1). Artmedica is one of the main medical facilities specialized in pediatric rheumatology.

**Objectives:** To describe a large cohort of patients with JIA from several regions of Colombia that are followed in a specialized private medical center.

**Methods:** A cross-sectional study was conducted in 366 patients who were enrolled in the registry between December of 2011 and August of 2016. There were 342 patients who met the International League of Statistical Associations for Rheumatology criteria who were included in the analysis. Association was examined by means of Chi-square tests, Kruskal-wallis test, and logistic regression analyses (to adjust for possible confounders).

**Results:** Analysis of clinical characteristics by subtypes showed differences in gender, rheumatoid factor positivity, bone erosions, axial and peripheral involvement (Table 29). Hand and/or feet deformities were present in 21,3% of the patients, bone ankylosis of the wrist in 9,6%, asymmetric extremities in 2,9%, and ankylosis of the hip in 1,2%. Complications such as uveitis, delayed growth and osteoporosis presented in 4,7%, 7,9% and 5,5%, respectively. Three cases of avascular necrosis and one case of macrophage activation syndrome were registered during follow up. No significant differences in DMARD and biologic therapy were found among groups, but less use of steroid therapy in oligoarthritis (OR 0,24 95%CI 0,09-0,91) and enthesitis related arthritis (OR 0,05 95%CI 0,02-0,16).

**Table 29:** **(Abstract P83).** Clinical characteristics of patients with JIA by subtypes

	All N = 342	Oligoarthritis N = 91	Polyarthritis N = 120	Systemic N = 28	Enthesitis related – Psoriatic N = 83	Un classified N = 20	p-value
Age at onset (median)	7	8	10	6	10	9	<.0001
Duration of disease (median)	4	3	7	6,5	3	33	<.0001
Gender Male:Female	4:6	4:6	2:8	4:6	7:3	4:6	<.0001
	N %	N %	N %	N %	N %	N %	p-value
RF (+)	92/252 36,5	17/63 26,9	62/112 55,3	3/15 20	5/50 10	5/12 41,6	<.0001
HLA-B27	26/126 20,6	7/34 20,5	3/23 13,1	0/4 0	15/61 24,6	1/4 25	0,648
Bone erosions	33/147 22,4	4/38 10,5	17/66 25,7	4/7 57	2/27 7,4	6/9 66,6	0.0002
Axial involvement	60 17,5	10 10,9	14 11,6	4 14,2	30 36,4	2 10	<.0001
Peripheral involvement	285 83,3	80 87,9	103 85,8	22 78,5	72 86,7	8 40	<.0001

**Conclusion:** Polyarthritis was more common in preadolescent females while enthesitis related arthritis in preadolescent males. Bone erosivity was particularly conspicuous in polyarticular and systemic JIA, which implies a great risk of deformities and ankylosis, and could justify a more agressive and early management. The definition of regional epidemiological profiles in JIA helps to understand differences in disease patterns, that allow both the development of personalized interventions and resources optimization.

**Disclosure of Interest:** None Declared

References

1. Ravelli A, Martini A, Villa L, Al. E, Lenko H, Wulffraat N. Juvenile idiopathic arthritis. Lancet (London, England). Elsevier Saunders, Philadelphia; 2007;369(9563):767–78.

### P84 Effectiveness and safety of tocilizumab in juvenile idiopathic arthritis (JIA) in clinical practice.

#### Gisela Díaz-Cordovés^1,2^, Natalia Mena^1^, Esmeralda Nuñez-Cuadros^2^, Sara Manrique^1^, Rocio Galindo-Zavala^2^

##### ^1^Department of Rheumatology, at the University Regional Hospital of Malaga (HRUM), MÁLAGA, Spain; ^2^Pediatric Rheumatology Unit, at the University Regional Hospital of Malaga (HRUM), MÁLAGA, Spain

###### **Correspondence:** Gisela Díaz-Cordovés

**Introduction:** Tocilizumab, an anti-interleukin 6-receptor, its an effective treatment in the juvenile idiopathic arthritis, but there is not so much date about the use in clinical practice in subcuteneous administration and what happens when we optimize it.

**Objectives:** To study the effectiveness and safety of tocilizumab (TCZ intravenous and subcutaneous) in JIA patients in clinical practice

**Methods:** Design:Case series. Population:Consecutive patients with JIA treated with TCZ in HRUM. Inclusion criteria: Patients with JIA, infant-juvenile and adult, followed in the Maternal and Child Hospital and Civil Hospital respectively. Exclusion criteria: Missing data on primary outcome variable. Protocol: Patients with infant-juvenile AIJ treated with TCZ. They are reviewed during every infusion of TCZ. Before age of 14 years old, patients are derived to Rheumatology Unit to be reviewed in adult unit and then they are followed each time they receive an infusion of the drug in day-hospital or every three months if they are in sc therapy (sc biological therapy clinic). Outcomes: Mean changes from baseline in DAS28, HAQ and activity level at 3rd month from change to sc TCZ and at 6 months from the reduction; count and description of serious and non-serious side effects during the follow-up. Other variables: Demographics, therapeutic and clinical-analytical data: tenderness joint count (TJC), swollen joint count (SJC), CRP, ESR, physician VAS, patient VAS, adverse events. Statistical analysis: Paired t test or Wilcoxon signed rank between the first and last TCZ administration, between the dose reduction and after 6 months, and between switching to sc administration and after 3 months

**Results:** The main baseline characteristics of patients with JIA during last administration of TCZ are shown in Table. The mean of DAS28 was higher in JIA adults in the last administration of TCZ (p = 0.028).During the exposure to TCZ three non-severe adverse events were recorded. Three patients switched to sc. At 3 months after to start sc TCZ, no differences were observed in effectiveness outcomes and any adverse effect with sc TCZ was recorded. There was not any switch to sc TCZ in patients with infant-juvenile JIA, compared to patients older than 14 years where 70% of them changed to sc TCZ (p = 0.022).Seven patients reduced doses: 6 with iv and 1 sc. Six months after the reduction, no difference was observed in effectiveness outcomes; only trend to fewer non-severe adverse effects in patient with reduced doses (3 with full doses versus none patient in dose reduction;p = 0.07). Only one patient returned to full dose because inefficiency.

**Conclusion:** Conclusions: TCZ in JIA patients seem an effective option in daily clinical practice. The switch from iv to sc TCZ in JIA patients seems effective in clinical practice. Reduction in TCZ dose in JIA may possible, maintaining good control of the disease and possibly with fewer side effects

**Disclosure of Interest:** None Declared

### P85 Fat mass in juvenile idiopathic arthritis. Should we think about it?

#### Gisela Díaz-Cordovés^1,2^, Esmeralda Nuñez-Cuadros^1^, Rocio Galindo-Zavala^1^, Sara Manrique^2^, Mena Natalia^2^

##### ^1^PEDIATRIC RHEUMATOLOGY UNIT, HOSPITAL REGIONAL UNIVERSITARIO DE MÁLAGA, MÁLAGA, Spain; ^2^Rheumatology D., HOSPITAL REGIONAL UNIVERSITARIO DE MÁLAGA, MÁLAGA, Spain

###### **Correspondence:** Gisela Díaz-Cordovés

**Introduction:** Various studies have shown that the excess of adiposity is a proinflamatory state that conditions worse response to treatment. There are few studies on the subject in arthritis juvenile idiopathic (JIA).

**Objectives:** OBJECTIVE: To describe the body composition and anthropometric parameters of patients with JIA, and evaluate the possible relationship with the activity of the disease.

**Methods:** PATIENTS and methods: Observational, cross-sectional, in children aged 4-15 years with JIA (excluding forms monoarticular), study in a tertiary hospital pediatric Rheumatology unit. It collected data anthropometric, clinical and of treatment. Also held dual absorptiometry of x-rays (DEXA) body total with assessment of body composition.

Define the index of mass fat (MFI) as the mass fat divided by the mass total and (MFFI) fat-free mass index as lean mass (kg)/height (m2).

The JADAS27 index was used for the evaluation of the activity of the disease.

**Results:** RESULTS: We analyzed 80 patients, whose characteristics are shown in Table 30. The most frequent subtype was oligoarticular JIA (16.3% extended, 47.5% persistent) followed by polyarticular JIA (25.1%). Twenty-five percent had uveitis. Fifty percent had inactive disease with treatment, 26% had activity and 23% were inactive without treatment. 52.5% had methotrexate and 30% had a biological drug (22.5% antiTNFα, 5% antiIL-1, 2.5% antiIL-6), with an average disease duration of 6.6 years (±3.7DE).They had mean scores of JADAS27 of 2 (± 4DE), PCR of 4.7 mg/l (± 9,5DE), VSG of 8.7 mm (± 7,2DE) and CHAQ of 0.17 (± 0,38DE). The anthropometric parameters are shown in Table 30. The mean of JADAS27 in patients with normal BMI, was lower than 1.7 (± 3.6 SD) for those who were overweight or with obesity 3.3 (± 6.0 SD), although not significant (p = 0.255). In simple linear regression, an increase of 0.3 JADAS27 was observed for each unit of IMG increase (p = 0.03). This relationship was maintained in the multivariate analysis (B 0.015; p 0.01) independently of the JIA subtype and the treatment received during the evolution.

**Table 30:** **(Abstract P85).** See text for description

VARIABLE	n = 80
Age in years, median (±DE)	10,7 (3,3)
Sex, female; n (%)	56 (70)
BMI	
Median en kg/m2 (±DE)	18,2 (4,2)
Percentile, median (±DE)	42,0 (29,9)
Index waist/hip (±DE)	0,84 (0,06)
Total fat (kg), media (±DE)	11,36 (8,9)
Total Lean (kg), media (±DE)	26,1 (8,9)
Total mass (kg), media (±DE)	38,8 (16,7)
MFI (%) media (±DE)	7,5(5,3)
MFFI (%) media (±DE)	17,8 (3,8)

Conclusion

CONCLUSION: In JIA patients, there is a linear relationship between IMG and disease activity measured by JADAS, but most patients had a normal BMI. The establishment of this relationship (fat-inflammatory activity) would be transcendental because of the need to optimize the recommendations in the JIA approach

**Disclosure of Interest:** None Declared

### P86 Hypovitaminosis D in juvenile idiopathic arthritis: prevalence and involved factors

#### Rocio Galindo Zavala^1^, Laura Martín Pedraz^2^, Esmeralda Núñez Cuadros^1^, Gisela Díaz-Cordovés Rego^3^, Antonio L. Urda Cardona^2^

##### ^1^Paediatric Rheumatology, Universitary Regional Malaga Hospital, MALAGA, Spain; ^2^Paediatrics, Universitary Regional Malaga Hospital, MALAGA, Spain; ^3^Rheumatology, Universitary Regional Malaga Hospital, MALAGA, Spain

###### **Correspondence:** Rocio Galindo Zavala

**Introduction:** 25hydroxi-vitamin D has antiinflammatory and immunomodulatory properties. Hypovitaminosis D prevalence in Juvenile Idiopathic Arthritis (JIA) patients ranges from 6% to 30%

**Objectives:** Evaluate hypovitaminosis D in JIA pediatric patients in Spain and assess involved factors.

**Methods:** Observational cross-sectional study in JIA Spanish patients from 4 to 15 years old, monitored by a Pediatric Rheumatology Unit. Monoarticular forms and patients with other chronic diseases were excluded.

Anthropometric, clinical and treatment data were recorded. Blood test with bone metabolism parameters and validated diet (KIDMED- Mediterranean Diet Quality Index) and exercise (PAQ-C/PAQ-A-Physical Activity Questionnaire for Children) questionnaires were obtained.

Hypovitaminosis D was defined as 25hydroxi-vitamin D plasma levels lower than 30 ng/ml

**Results:** 76 children were enrolled. Their characteristics are included in Table 31.

**Table 31:** **(Abstract P86).** See text for description

PATIENTS` CHARACTERITICS (N = 76)		
Gender (Male),		23 (30,3)
Age (years), median (IR) n (%)		10,83 (8,52-13,54)
25OH-Vitamin D (ng/mL), mean (+/-SD)		34,04 ng/mL (8,90 ng/mL)
DISEASE CHARACTERISTICS (N = 76)		
JIA subtype, n (%)	Systemic	9 (11,8)
	Oligoarticular	47 (61,9)
	Polyarticular	20 (26,3)
Inflammatory activity duration (days), median (IR)		385,0(246,75-761,25)
RECEIVED TREATMENTS (N = 76)*		
Systemic GC, n (%)		61 (80,2)
Synthetic DMARDs treatment, n (%)		39 (51,3)
Biological DMARDs treatment subtype, n(%)	Anti-TNFα	15 (19,7)
	Anti-IL1	4 (5,2)
	Anti-IL6	2 (2,6)

JIA: Juvenile idiopathic arthrtis; RF: Rheumatic factor; GC: glucocorticoids;

DMARD: Disease-modifying antirheumatic drug; Anti- TNFα: anti- tumoral necrosis factor α;

Anti-IL1: anti-interleukin 1; Anti-IL6; anti-interleukin 6

The population's prevalence estimation of hypovitaminosis D was 16 - 35% (CI 95%).

We found no relationship between 25 hydroxi vitamin D levels and sex, JIA subtype nor duration or dose of systemic glucocorticoids.

In bivariate analysis we found direct association between hypovitaminosis D and Body Mass Index percentile (BMIp)(p = 0,05), received dose of prednisone (p = 0,03) and clinical activity duration (p = 0,04); and an inverse relation with physical activity level (p = 0,04).

In multivariate analysis, relationship between hypovitaminosis D and BMIp (B 0,024; p 0,016) and with disease activity (B 0,015; p 0,01) were maintained. Moreover, we found an inverse association with biological disease-modifiying antirheumatic drugs (B-4,69; p 0,048), specifically with anti tumoral necrosis factor α (antiTNFα) (B -4,7; p 0,042)

**Conclusion:** Hypovitaminosis D prevalence in our population is similar to previously described.

JIA patients with higher BMIp have more hypovitaminosis D, as it has been reported in other inflammatory diseases.

A direct relationship exists between inflammatory activity and hypovitaminosis D, but we need more studies to asses if one is cause or consecuence of the other.

Patients treated with antiTNF have higher plasma levels of 25 hydroxi-vitamin D, this can be explained because these drugs may increase 25 hydroxi-vitamin D levels or due to a better response to antiTNF of those patient with higher plasma levels of 25 hydroxi-vitamin D.

**Disclosure of Interest:** None Declared

### P87 Neuropsychological assessment in juvenile idiopathic arthritis - Part I: cross-sectional study in a single Hungarian centre

#### Diana Garan^1^, Gyurgyinka Gergev^1^, Wouter Wijker^2^, Imre Bozi^2^, Tamás Constantin^1^

##### ^1^Rheumatology, 2nd Department of Pediatrics Semmelweis University, Budapest, Hungary; ^2^Auxiliis-Pharma Kft., Budapest, Hungary

###### **Correspondence:** Diana Garan

**Introduction:** Juvenile idiopathic arthritis (JIA) is one of the most common pediatric chronic illnesses. A remarkable number of patients need long term or lifelong treatment. In ‘biological era’ many publications have strengthened the safety and effectiveness of TNF inhibitors and some of them noticed psychological or neuropsychological (NP) adverse events (AE). The relationship of these AEs with the treatment is unclear. There is no evidence available if these skills vary in different therapy and change during JIA therapy.

**Objectives:** Our aims are to estimate NP state in JIA treatment groups and specify NP state influencing factors. The next step was to compare NP state in different treatment groups.

**Methods:** Descriptive statistics and exploratory analysis were completed. 120 patients were examined at the Paediatric Rheumatology Unit (Semmelweis University, Budapest) between 01.01.2015 - 31.12.2016. 68 patients received combined therapy (TNF inhibitor + methotrexate (MTX) and/or salazopyrin (SSZ)), 42 patients received DMARD: MTX +/-SSZ, 18 patients were on only TNF inhibitor therapy. After a formal psychological assessment, all patients were tested with age specific questionnaires as well: Infant Behaviour Questionnaire, Children’s Behaviour Questionnaire, Child Behaviour Checklist. Furthermore, by using Woodcock Johnson III Tests of Achievement, three NP variables were compared in treatment groups: attention, learning, and working memory. In addition, current and baseline disease activity, duration of therapy and demographic parameters were assessed in each group. These parameters were stratified, and NP variables were compared in these strata. Comparison of NP variables of different treatment groups were age-, age at diagnosis-, gender-, duration of treatment-, current and baseline JADAS disease activity-matched.

**Results:** In the analysis of each treatment groups we did not observe any significant deviation of all NP variables based on age, gender, duration of treatment, current JADAS disease activity. All NP variables were the highest in patients whose JIA was diagnosed between 3,1-5,25 years of age. In combined therapy group attention was significantly (p < 0,05) higher in patients whose baseline disease activity was low. In the analysis of comparison of treatment groups the following differences were observed: learning score was significantly lower (p < 0,05) in male patients in MTX group than in the combined therapy group. Working memory score was significantly (p < 0,05) higher in patients whose JIA was diagnosed between 3,1-5,25 year of age, and whose treatment duration was longer than 3 years in combined therapy group. Attention score remained stable in age-, age at diagnosis-, gender-, JADAS disease activity-matched treatment groups. There was no significant discrepancy in all three NP variables between disease activity-matched treatment groups.

**Conclusion:** We have not observed any clinically significant difference in the neuropsychological state in different treatment groups assessed only at a definite date. Age, age at diagnosis, gender, duration of treatment, current and baseline JADAS disease activity were not clinically influencing factors of NP variables in this study. The analysis of cognitive function has already been assessed, but has not been analysed yet. In a prospective study we will follow patient’s psychological changes before and during therapy in order to observe if different therapies have any effect on psychological functions in JIA patients.

**Disclosure of Interest:** None Declared

### P88 Characteristics of TNF inhibitor treatment in juvenile idiopathic arthritis - 2-year-follow-up in a Hungarian centre

#### Diana Garan, Anita P. Juhász, Andrea Ponyi, Tamás Constantin

##### Rheumatology, 2nd Department of Pediatrics Semmelweis University, Budapest, Hungary

###### **Correspondence:** Diana Garan

**Introduction:** TNF inhibitors have been widely used in the therapy of juvenile idiopathic arthritis (JIA) since the early 2000s.

**Objectives:** We assessed the efficacy, change in efficacy, reason for treatment discontinuation, relapse rate of TNF inhibitor treatments for JIA started between 2012-2016 at the Unit of Pediatric Rheumatology, Semmelweis University, Budapest.

**Methods:** 146 treatments were started for 116 patients with polyarticular course JIA. The following variables were assessed: Giannini improvement and JADAS-71 score every 3 months, the time for achieving at least 90% Giannini improvement, reason for treatment discontinuation, relapse rate. We estimated the alteration of Giannini improvement and JADAS-71 disease activity in patients treated for at least 24 months. Finally, we analysed the features of patients achieved inactive disease. Paired Wilcoxon test, Kaplan-Meier survival analysis, log rank test, chi square test, Fisher’s test, T test were used with SPSS statistics program.

**Results:** Statistically significant (p < 0,05) alteration was detected in achieving ever higher improvement of the Giannini’s criteria in the first 12 months of treatment, afterwards this alteration remained in tendency but not statistically. The JADAS-71 disease activity significantly decreased in the first 6 months of the treatment, afterwards the decline remained in tendency. Giannini 90% improvement was achieved significantly more frequently in those who received etanercept treatment, first-line TNF inhibitor treatment and any first-line biological. Among those who achieved inactive disease, there were significantly more of those, who started treatment with fewer active joints, lower JADAS score and CHAQ values, and were significantly younger at diagnosis. Biological therapy was discontinued due to inefficacy in 50,8%, remission 28% and adverse events 10,5% of cases. In addition, 37,5% of patients had relapse whose treatment was discontinued due to remission, all within the first 3 months on off-medication.

**Conclusion:** Giannini’s criteria improved significantly over the first 12 months, the disease activity decreased significantly in the first 6 months, afterwards these observations remained in tendency in our cohort. Approximately one-third of treatments were discontinued due to remission almost two-third of these patients have not experienced relapse during our observation period.

**Disclosure of Interest:** None Declared

### P89 Body composition measured by DEXA in children with juvenile idiopathic arthritis. do they have sarcopenia?

#### Pilar Guarnizo^1,2,3^, Sally Pino^2,4^, Adriana Diaz-Maldonado^2,5,6^, Juan Manuel Reyes^2^

##### ^1^Fundacion Cardio Infantil, Bogota, Colombia; ^2^Care for Kids, Bogota, Colombia; ^3^Cayre, Bogota, Colombia; ^4^Hospital Universitario Infantil de San Jose, Bogota, Colombia; ^5^Hospital de La Misericordia, Bogota, Colombia; ^6^Instituto Roosevelt, Bogota, Colombia

###### **Correspondence:** Pilar Guarnizo

**Introduction:** Juvenile Idiopathic Arthritis (JIA) is the most frequent disease in pediatric rheumatology. There are few studies about body composition in these population, most of them suggest they have excess fat.

Sarcopenia in children and young adults is pathologic and constitute a risk factor of morbi-mortality due to its association with alteration in immunologic function, decrease strength muscle and thermoregulation capacity, exercise tolerance and functionality, all of them affect articular function and preservation. Additionally, bone mass accretion and bone mineral density are related proportionally direct to muscle mass.

Also patients with sarcopenia could present an increase in the fat mass known as sarcopenic-obesity.

**Objectives:** To describe body composition alterations in a cohort of children diagnosed with JIA in Bogota, Colombia.

**Methods:** Cross-sectional study where a cohort of children with JIA was assessed the anthropometry and densitometry measured by dual-energy X-ray absorptiometry (LUNAR iDXA). It was evaluated stature and weight. The fat mass was evaluated through Fat Mass Index (FMI): Kg/m^2^, subtotal percentage body fat for age and gender, and subtotal trunk fat mass. To measure lean mass was used: lean mass index (LMI): Kg/m^2^, appendicular lean mass (arm and leg lean): Kg/m^2^, and total bone mineral density less head (TBLH)

The statistical analysis was only descriptive where it involved measurement of central tendency and dispersion.

**Results:** Thirty children were included in the study. Fifty three percentage were female with average age of 13.8 years (ranked from 5 to 22 years).All subtypes of arthritis were included, 56% had systemic and polyarticular subtypes.

The average of onset of the disease was 5 year (ranked from 2 to 14 years).

In this cohort 33% of the patients had short stature.

36% patients had compromise in fat mass. FMI median z-score was -0.57 (ranked from -1.66 to 1.11), 26% of children presented fat deficiency (12.5% of them had severe fat deficit), and 10% excess fat - obese. In 20% of patients the percentage of fat was above of expected and trunk fat mass was higher in 16% of the studied children; the last measure is related to cardiovascular risks.

80% of the patients had low LMI z-score that is considered as low lean mass- sarcopenia. LMI z score median was -2.17 (ranked from -4.56 to 0.36). 58.6% presented low appendicular lean mass.

30% of patients had compromise in two compartments (FMI and LMI). 100% of patients with Fat deficit had low lean mass –sarcopenia, while 33% of patients had sarcopenic-obesity (fat excess with sarcopenia) which is associated to decrease in life- expectancy.

The median z score for Total bone mineral density less head (TBLH) was -1.25 (ranked from -3,8 to 1,5 . The TBLH density was low in a 30% of patients, from which 100% had low lean mass-sarcopenia.

**Conclusion:** The included children diagnosed with JIA presented high percentage of disorders of body compositions characterized by sarcopenia 80% followed by fat mass compromise in 36% (low fat mass 26% and excess fat 10%) and low bone density in 30%.

The abnormalities in the lean mass could be explained due to the increase in metabolic rate and catabolism which lead to skeletal muscle consumption. It has been described “rheumatic cachexia” in 67% of adults with rheumatoid arthritis, affecting bone mineralization, strength muscle loss, and is associated with cardiovascular risk increase, but there is not any data in children with JIA.

This is the first study which measure sarcopenia by DEXA in this population, additional studies including more patients are necessary to validated these results and establish opportune interventions to improve their nutritional status and quality of life.

**Disclosure of Interest:** None Declared

Reference

1. Evaluacion del estado nutricion en una población mexicana de pacientes adultos con artritis reumatoide. L. Puentes, G. F. Hurtado, C. Abud, A. Bravo. NutrHosp. 2009:24:233-238

### P90 Clinical TMJ involvement in JIA patients according to the ILAR categories : preliminary study in 137 children.

#### Severine Guillaume Czitrom, Gianpaolo de Filippo

##### Service de Medecine des Adolescents, Chu Bicetre, Kremlin-Bicetre, France

###### **Correspondence:** Severine Guillaume Czitrom

**Introduction:** Temporo-manidibular joint (TMJ) involvement in juvenile idiopathic arthritis (JIA) is the subject of many unanswered questions.

**Objectives:** We here wonder if the ILAR categories might help identifying JIA patients at risk of TMJ involvement.

**Methods:** In single cohort of consecutive JIA patients, TMJs were systematically examined at each follow-up visit of JIA patients according to the European recommendations. Clinical involvement of TMJs over time was longitudinally recorded during JIA follow-up. JIA categories were defined according to ILAR definitions.

**Results:** There were 137 JIA patient, comprising 89 girls and 44 boys aged 14.13 years +/- 4.99 at the time of the analysis. Mean age at disease onset was 7.84 years +/- 4.89 and mean follow-up was 4.41 years +/- 3.83 [0.2-23.9 years]. There were 63 ERAs, 33 oligoarticular JIAs with 26 persistent and 7 extended diseases, 27 polyarticular JIAs, 6 being RF positive, 10 psoriatic arthritis and 4 systemic JIAs.

44 JIA children had clinically detectable TMJ involvement (32%), but the percentages were rather different across the ILAR sub-groups : TMJs were symptomatic in 12 ERAs (19%), in 9 oligoarticular JIAs (27.3%), in 18 polyarticular JIAs (66.7%), in 3 psoriatic arthritis (30%) and in 2 systemics (50%) ; moreover, within the oligoarticular group, the most affected category was the oligo-extended group with 6 out of 7 patients having TMJs involved (85.7%).

Interestingly, the clinical manifestations were not the same since half of the oligoextended JIA patients reported no pain, no chewing difficulty, no evidence of mouth opening limitation but insidiously developped facial asymetry due to unilateral TMJ inflammation. In all cases, the spreading of the arthritic process happened before 5 of age. In contrast, ERA patients manifested pain and reduced mouth opening much more frequently and at a later age, prompting to a quick therapeutic answer.

**Conclusion:** In conclusion, TMJ arthritis might have severe consequences in those patients starting agressive disease early in life, as many extended-oligoarticular as well as part of polyarticular JIAs and systemics. Detecting the patients at high risk for structural damage in TMJs should be a major goal in the coming years. This preliminary study also shows that a significant proportion of ERA patients suffer from TMJ inflammation during the course of their disease. The limited number of patients is the drawback of this preliminary study and larger prospective studies are required to validate these data.

**Disclosure of Interest:** None Declared

### P91 MRP8/14 and conventional inflammatory markers explaining physician’s global assessment of disease activity in new-onset juvenile idiopathic arthritic patients

#### Paula Keskitalo^1^, Salla Kangas^2^, Paula Vähäsalo^1^

##### ^1^Department of Children and Adolescents, University of Oulu, Oulu, Finland; ^2^Medical Research Center Oulu, Oulu University Hospital and PEDEGO Research Unit, University of Oulu, Oulu, Finland

###### **Correspondence:** Paula Keskitalo

**Introduction:** Juvenile idiopathic arthritis (JIA) is a chronic inflammatory joint disease affecting children. In clinical practice it is occasionally difficult to assess the real inflammatory activity. Physician’s assessment of global disease activity (PGA) is a holistic assessment and it is always available. The myeloid-related protein complex 8/14 (MRP8/14, calprotectin) secreted by infiltrating phagocytes in synovial inflammation has been suggested to function better than conventional acute-phase reactants as a marker of low inflammatory activity and to be more sensitive and reliable indicator of disease activity.

**Objectives:** The aim of our prospective, observational cohort study was to analyze whether the MRP8/14 levels in serum (S-MRP8/14) or plasma (P-MRP8/14) and conventional inflammatory markers, such as erythrocyte sedimentation rate (ESR), C-reactive protein (CRP), leucocytes (leuc) and neutrophils (neutr), correlate with disease activity assessed by physician.

**Methods:** Serum and plasma MRP8/14 concentrations from 88 concecutive new-onset JIA patients collected before starting DMARDs were measured by human calprotectin ELISA kit (Hycult biotech). Clinical data of the patients with median age of 6.6 yrs (range 1 – 15.3 yrs) were collected during the visits in the pediatric rheumatology outpatient clinic in Oulu University Hospital between October 2011 and November 2014. Median PGA was 21 with the scale 0-100 (range 3 – 85), median patients/parents global assessment of wellness 31 (range 0 – 88), median patients/parents assessment of pain 31 (range 1 – 45), and median number of joints with active arthritis (AJC) 3 (range 1 – 45). Correlations were analyzed by Spearman’s coefficient. Multiple linear regression was used to quantify the strength of the relationship between laboratory inflammatory markers and to assess which parameter has the most powerfull coefficient of determination of PGA.

**Results:** Median P-MRP8/14 concentration was 184.9 ng/ml (range 1.2 – 685.9 ng/ml) and in S-MRP8/14 255.0 ng/ml (67.4 – 553.5 ng/ml). Median CRP was 1.5 mg/l (1.5 – 193 mg/l), ESR 13 mm/h (2 – 98 mm/h), leuc 7.4 x 10E9/l (3.4 – 15.2 x 10 E9/l) and neutr 3.9 x 10E9/l (1 – 10.5 x 10E9/l).

We found positive correlations between PGA and the laboratory parameters. PGA weakly correlated with leuc (Spearman’s Rho 0.281, P = 0.008) and neutr (0.313, P = 0.007). Association was better with ESR (0.432, P < 0.001), CRP (0.582, p < 0.001), P-MRP8/14 (0.682, P < 0.001) and S-MRP8/14 (0.409, P < 0.001).

PGA has stronger association with P-MRP8/14 than with the other inflammatory parameters (Table 32.). Multiple linear regression analysis showed leuc, ESR, CRP, neutr, S-MRP8/14 and P-MRP8/14 explaining 55% of PGA. When analyzed the standardized B the strongest relationship was found between P-MRP8/14 and PGA (B = 0.084 (95% CI: 0.045 – 0.123) P < 0.001, standardized B = 0.733), but association between S-MRP8/14 and PGA remained weak (B = -0.032 (95% CI: -0.074 – 0.010), P = 0.14, standardized B = -0.203).

**Table 32:** **(Abstract P91).** Relationships between PGA and laboratory parameters analyzed by linear regression

	B	95% CI	P-value	R^2^
ESR	0.358	0.194 – 0.523	<0.001	18.3%
CRP	0.345	0.227 – 0.463	<0.001	28.7%
Leucocytes	1.387	0.064 – 2.710	0.040	4.9%
Neutrophils	1.849	-0.289 – 3.986	0.089	4%
MRP8/14 in serum	0.056	0.024 – 0.087	0.001	12.9%
MRP8/14 in plasma	0.075	0.056 – 0.094	<0.001	47.8%

R^2^ coefficient of determination, B regression coefficient

**Conclusion:** P-MRP8/14 seemed to be much better than the other conventional inflammatory laboratory markers or MRP8/14 serum concentration when assessing disease activity in JIA patients. P-MRP8/14 concentration might be useful indicator to be used in clinical practice when making evalution of disease activity.

**Disclosure of Interest:** None Declared

### P92 Chronic arthritis as the only manifestation of FMF in Armenian children

#### Gayane Khloyan

##### ARABKIR JMS ICAH, Yerevan, Armenia

**Introduction:** Familial Mediterranean Fever (FMF) is the most common inh**e**rited auto inf**l**ammatory disease, characterized by recurrent, self–limited attacks of fever and aseptic polyserositis.FMF is widespread in Armenia and there is a higher than expected frequency of FMF-associated arthritis in children. Chronic arthritis can be the first, and sometimes the only manifestation of FMF.

**Objectives:** To the importance of genetic investigation for MEFV gene mutations in patients of high-FMF-prevalence ethnicities with chronic arthritis.

**Methods:** Case reports of two patients with chronic arthritis as their first symptom of FMF.

**Results: 1-st case**: A 6 year-old boy was admitted to the hospital with complaints of weakness and limping during the last 1.5 years. He had also self- limited episodes of knee and foot pain. The physical examination revealed pain on flexion of the left knee, painful palpation of both sacroiliac joints, but no visible or palpable changes of the joints. Initial laboratory examination showed elevation of ESR– 49 mm/h and CRP-48 mg/l. WBC, RBS, PLT were in normal range. An extensive diagnostic work up including biochemical examination, serology tests, urine test, chest X-ray, Echo-CG revealed no changes. Splenomegaly was found on abdominal sonography. MRI of the pelvic bones and hip joints indicated signs of bilateral sacroiliitis. Chronic arthritis was diagnosed. Despite of absence of clinical features of FMF, genetic analysis for MEFV gene mutations was done and homozygous genotype for M694V mutation (*M694V/M694V*) was found. Treatment with colchicine and sulfasalazine was started and both his clinical condition and laboratory findings improved significantly. Only occasionally the patient still has gait abnormalities.

**2-nd case.** A 1.8 year-old girl was admitted to the hospital because of pain, swelling and deformity of the left wrist, which had started 5 months earlier. In her past history 2 episodes of unexplained fever of 1 day duration were noticed. The physical examination revealed swelling, pain and limitation of motions of the left wrist. ESR was elevated within 53 mm/h, but CRP was negative. Thorough diagnostic work up (CBC, biochemical examination, serology tests, urine test, chest X-ray, Echo-CG, abdominal sonography) revealed no pathologies. MRI of the left wrist indicated signs of arthritis. Chronic arthritis was diagnosed. But also genetic analysis of *MEFV* mutations was done and 2 mutations (M694V/R761H) in compound-heterozygous state were found. Intraarticular injection of triamcinolone acetonide to the left wrist was done and colchicine treatment was started with significant improvement: during 4 months of follow up the girl had no complaints and no inflammatory activity with normal level of ESR.

**Conclusion:** In ethnicities with high prevalence of FMF, screening for MEFV gene mutations is recommended for patients with chronic arthritis even in the absence of FMF symptoms.

**Disclosure of Interest:** None Declared

### P93 The efficacy of etanercept in non-systemic juvenile idiopathic arthritis in Saint-Petersburg: the preliminary data of 148 patients from biologic registry.

#### Mikhail M. Kostik, Irina Chikova, Eugenia Isupova, Margarita Dubko, Vera Masalova, Ludmila Snegireva, Ekaterina Gaidar, Olga Kalashnikova, Vyacheslav Chasnyk

##### Saint-Petersburg State Pediatric Medical University, Saint-Petersburg, Russian Federation

###### **Correspondence:** Mikhail M. Kostik

**Introduction:** Etanercept was the first biologic drug approved in children. Russian rheumatologist received the access to etanercept only in 2009 after infliximab, adalimumab, abatacept. Currently etanercept reach the first position in the biologics, used in JIA in Saint-Petersburg.

**Objectives:** The aim of our study was to evaluate the efficacy of etanercept in the tertial hospital in Saint-Petersburg using the registry.

**Methods:** The data about all patient (n = 148) who received etanercept were included into the registry. We evaluated all routine JIA measures, ESR, CRP, achievement the remission every 6 months, dynamics of height, weight and side effects. The duration of study was more than 30 months.

**Results:** The demographic of study population was: girls were 92/148 (62.2%)**,** onset age - 4.9 (2.4; 9.6), age of etanercept initiation: 9.8 (5.9; 13.9) time between JIA onset and etanercept was 2.4 (1.2; 5.8). Duration of etanercept treatment was 3.5 (2.3; 4.3) years. The distribution of JIA categories was: persistent oligoarthritis 6/148 (4.1%), extended oligoarthritis 11/148 (7.4%), RF-negative polyarthritis 84/148 (56.8%), RF-positive polyarthritis - 3/148 (2.0%), enthesitis-related arthritis - 32/148 (21.6%), psoriatic arthritis - 9/148 (6.1%), non-differentiated arthritis 3/148 (2.0%). ANA-positivity was in 33/120 (27.5%), HLA B27 - 24/80 (30%) and RF-positivity - 5/148 (3.4%).

During the trial the etanercept shows high efficacy in the main JIA measures and laboratorial tests (Table 33). Also we have observed decreasing of WBC (p = 0.03), PLT (p = 0.000001), ESR (p = 0.03), increasing the hemoglobin (p = 0. 006), body mass index (p = 0.02). Frequency of morning stiffness decreased from 62.8% to 4.6% (p = 0.0001). The achievement of remission (ACRPedi100) reached 69.3% patients.

**Table 33:** **(Abstract P93).** See text for description

Parameter	Baseline	End of study	p
Morning stiffness, minutes	60.0 (30.0; 120.0)	20.0 (10.0; 20.0)	0.03
Global disease activity, mm	48.0 (35.0; 68.0)	0.0 (0.0; 28.0)	0.000001
MD VAS, mm	43.5 (30.0; 60.0)	0.0 (0.0; 18.0)	0.000001
Patient’s VAS, mm	20.0 (10.0; 20.0)	0.0 (0.0; 10.0)	0.000001
Painful joints, n	2.0 (1.0; 5.0)	0.0 (0.0; 0.0)	0.000001
Swollen joints, n	4.0 (2.0; 11.0)	0.0 (0.0; 0.0)	0.000001
Joints with LOM, n	4.0 (1.0; 9.0)	0.0 (0.0; 0.0)	0.000001
Active joints, n	7.0 (3.0; 15.0)	0.0 (0.0; 0.0)	0.000001
CRP, mg/l	1.9 (1.0; 5.5)	0.5 (0.2; 1.3)	0.000001

Etanercept was discontinued in 12/148 (8.1%) due to primary or secondary inefficacy or new onset of uveitis, injection site reactions were rare and lead to discontinuation of etanercept in 1/148 (0.7%). No serious infections, tuberculosis, malignancy were observed during the study.

**Conclusion:** Etanercept show high efficacy in non-systemic JIA patients. Serious side effects were rare and comparative to literature data. Registry of biologics is a good tool for collection data about patients. Future investigations required.

**Disclosure of Interest:** None Declared

### P94 The treatment of 40 polyarticular juvenile idiopathic arthritis children with tocilizumab: single center experience.

#### Mikhail M. Kostik, Irina Chikova, Eugenia Isupova, Margarita Dubko, Vera Masalova, Ludmila Snegireva, Ekaterina Gaidar, Olga Kalashnikova, Vyacheslav Chasnyk

##### Saint-Petersburg State Pediatric Medical University, Saint-Petersburg, Russian Federation

###### **Correspondence:** Mikhail M. Kostik

**Introduction:** Juvenile idiopathic arthritis (JIA) – is a chronic inflammatory joint diseases, which affects the children under the age 16. Methotrexate (MTX) is a basis of treatment of patients with polyarticular JIA (pJIA). Patients, who fall or intolerance MTX required in biologic treatment.

**Objectives:** The aim of our study was evaluate the efficacy and safety of tocilizumab (TCZ) in children with pJIA.

**Methods:** In the present retrospective study with maximal duration 42 months were included 40 children (82,5% girls) with active pJIA, resistant to previous therapy with MTX alone or combination with others non-biologic DMARDs, whom were treated with TCZ every 4 weeks 8 mg/kg (weight ≥ 30 kg) and 10 mg/kg (weight < 30 kg). Onset JIA age was 4,8 (2,9; 8,1) years, time between JIA onset and start of TCZ was 5,7 (1,8; 8,5) years. Duration of TCZ treatment was 1109 (452; 1542) days. TCZ monotherapy was in 13,2% children.

**Results:** During TCZ course NSAID were discontinued in all studied population, decreased part of children who were treated with combination of non-biologic DMARDs from 37.5% to 2.5% and proportion of whom were treated with MTX from 92.5% to 75.0%, and leflunomide up to 2.5%. The discontinuation of the second DMARD, corticosteroids, cyclosporine and NSAIDs were due to achievement of remission and rare due to intolerance. In first 6 months of therapy was revealed impressed decreasing of ESR (p = 0.000002), CRP (p = 0.00002), WBC and (p = 0.001) PLT (p = 0.00002) up to normal ranges during whole TCZ course. Number of active joints decreased gradually (p = 0,00002), reached the median = 0 to 24^th^ month, 15.3% obtained inactive disease status in 6 months and 80% in 42 months. During the study inactive disease status was reached totally in 60.5% patients and in 3 patients TCZ was discontinued due to persistent remission. The main predictors of achievement inactive disease were WBC < 9.0 x 10^9^/l (HR = 1,92, p = 0.16), absence of previous biologic treatment (HR = 1.92, p = 0.16). The absence of previous biologic treatment was also predictor of low disease activity status (HR = 2.4, p = 0.01).

Among adverse effects were transient hypercholesterolemia and hypertriglyceridemia and single episode of grade II neutropenia which not lead to TCZ discontinuation infection side effects and antibiotic treatment.

**Conclusion:** The safety and efficacy of TCZ was demonstrated in children with pJIA. Serious adverse events were not revealed.

**Disclosure of Interest:** None Declared

### P95 Atypical oligoarticular juvenile arthritis in children: elbow chronic monoarthritis

#### Aleksey Kozhevnikov^1,2^, Nina Pozdeeva^1^, Michael Konev^1^, Maxim Nikitin^1^, Anastasia Bryanskaya^1^, Evgeniy Prokopovich^1^, Konstantin Afonichev^1^, Gennadiy Novik^2^

##### ^1^Orthopedic and rheumatology department, Federal state budget institution “The Turner Scientific and Research Institute for Children's Orthopedics”, Saint-Petersburg, Russian Federation; ^2^Pediatric department n.a. prof. I.M. Voroncov, Federal state budget institution of higher professional education “Saint-Petersburg State Pediatric Medical University”, Saint-Petersburg, Russian Federation

###### **Correspondence:** Aleksey Kozhevnikov

**Introduction:** Juvenile arthritis is a broad term that describes heterogeneous group of a chronic inflammatory joint disease which characterized by progressive course leading reduced mobility and function. Progressive chronic arthritis of an unknown cause lasting for at least more than three months are the main diagnostic criteria of JIA. A typical classic variant of oligoarticular JIA (oligo-JIA) is well known. Monoarthritis of the elbow joint is atypical onset oligo-JIA. It’s difficult to differentiate of elbow chronic synovitis due to clinically heterogeneous. The instrumental and specific diagnostic tests are great assistance to determine cause of synovitis.

**Objectives:** Chronic synovitis in children arises from several causes. There are inflammatory, infectious, traumatic, reactive, hemorrhagic, neoplastic and undifferent etiologies of synovial diseases. Usually chronic elbow monoarthritis associated with tumor-like conditions (pigmented villonodular synovitis, synovial haemangioma and chondromatosis), osteomyelitis, tuberculosis arthritis, osteochondropathy and rare JIA. The aim of the present study was to determine diagnostic and treatment strategies of elbow monoarthritis manifested like oligo-JIA.

**Methods:** We carried out a retrospective review of sixteen children with chronic undifferentiated elbow monoarthritis which were hospitalized at Children Orthopedics Institute, Saint-Petersburg (rheumatology department) between 2011 and 2016. The data of clinical, serological, x-ray, ultrasound, MRI, arthroscopy and synovial fluid were analyzed. Detected atypical/diffuse form synovial proliferation or limited to a well-defined single nodule were recommended for arthroscopy and biopsy. Six children were excluded from study due to verification of cause elbow monoarthritis: 6-yr-old girl - PVNS, 10-yr-old boy cavernous haemangioma and 17-yr-old boy - synovial chondromatosis, 2 small girls and 1 boy - osteoid osteoma. Ten children were study group (median age 5,8 ± 2,5, range 3-11 years; female 90%, male 10%). Children with post-traumatic elbow joint transient effusion were controls.

**Results:** Trauma of elbow joint related to onset chronic arthritis, progressive flexure contracture with dry synovitis, low activity and the long-term period absence of clinical involvement of other joints were occurred in all children. Asymptomatic early-stage, progressive flexure/combined contracture less joint effusion and morning stiffness were cause of late diagnosis of oligo-JIA. Only seven children (all girls) were ANF positive ≥ 1:160, two HLA-B27 positive. Radiographic finding of early-stage JIA were accelerated cartilage model ossification of distal humeral epiphysis and trochlear, osteoporosis with subchondral bone sclerosis and cyst-like deformation. The MR imaging were non-specific inflamed elbow synovium. Overgrowth of epiphysis and trochlear with joint space narrowing, deformation articular surfaces with erosive changes were radiographic findings of late-stage JIA. MR imaging revealed significance multiple erosive synovitis with bone cyst-like deformation. Ultrasound wasn’t show elbow synovitis at half of the study children. 70% children were negative effect of monotherapy NSAID. Positive treated effect was achieved after intra-articular triamcinolone injection (20-40 mgs) and methotrexate therapy (15 mg/m^2^/week) > 6 months, splinting and physiotherapy. After 2-3 years 30% children were persistence oligo-JIA (involved wrist or knee), 40% - extended oligo-JIA, 30% - isolated monoarthritis. Children with post-traumatic elbow joint deformation haven’t revealed chronic synovitis.

**Conclusion:** Monoarthritis of elbow joint are rare atypical manifestation of pauciarticular JIA. Non-specific clinical signs and instrumental imaging may contribute to diagnostic problems of elbow chronic synovitis. Therapy of elbow chronic idiopathic monoarthritis must be coincide treat-to-target strategy in juvenile arthritis. Ultrasound can’t be the decisive diagnostic method of chronic elbow pathology.

**Disclosure of Interest:** None Declared

### P96 Platelet microparticles level in juvenile idiopathic arthritis

#### Sathish Kumar, Naresh Kumar

##### Rheumatology, Department of Pediatrics, Christian Medical College, Vellore, India

###### **Correspondence:** Sathish Kumar

**Introduction:** Juvenile idiopathic arthritis (JIA) encompasses a complex group of disorders comprising several entities in children. Multiple biomarkers have been used to detect the disease activity. Platelet Microparticles(PMP) are small vesicles released from plasma membrane upon platelet activation. Several studies stated that PMP has been associated with the thrombotic event and inflammatory process in adults. This is the first study in literature to estimate PMP in JIA.

**Objectives:** To estimate the level of plasma PMP in children with JIA and to study the relationship with disease activity of JIA.

**Methods: Study design:** Cross-sectional study

**Inclusion criteria:** Children with JIA who fulfilled the International League of Associations for Rheumatology (ILAR) classification criteria for juvenile idiopathic arthritis were included.They were categorized into active disease group and inactive disease as assessed by Wallace criteria. Complete Blood count, ESR, CRP, and PMP were estimated in all children. PMP level was calculated by Flow Cytometry.Blood investigations and baseline demographics were analyzed

**Results:** Out of 26 children with JIA, 12 had active disease group and 14 had an inactive disease as assessed by Wallace criteria. Mean PMP level was 83507 cells/μland 34904 cells/μlin active and inactive disease respectively (p = 0.06). There was no significant correlation between PMP level in JIA patients with corresponding ESR, CRP, and platelets

**Conclusion:** Our study states that level of PMP is significantly elevated in disease activity of JIA and could represent a new biomarker reflecting the state of cell activation in JIA. PMP role in the inflammatory processes needs to be further elucidated

**Disclosure of Interest:** None Declared

## Miscellaneous rheumatic diseases

### P97 The need for dedicated paediatric rheumatology services: retrospective review of a clinic service at Tygerberg Hospital, South Africa

#### Deepthi R. Abraham^1^, Monika M. Esser^2^

##### ^1^Dept of Paediatrics and Child Health, Faculty of Medicine and Health Sciences, NHLS Immunology Unit Tygerberg Hospital, Division Medical Microbiology, Faculty of Medicine and Health Sciences, Stellenbosch University, Cape Town, South Africa; ^2^Department of Pathology, NHLS Immunology Unit Tygerberg Hospital, Division Medical Microbiology, Faculty of Medicine and Health Sciences, Stellenbosch University, Cape Town, South Africa

###### **Correspondence:** Deepthi R. Abraham

**Introduction:** Paediatric Rheumatology in South Africa competes with limited health care resources allocated to epidemic diseases. Data on prevalence, incidence, disease categories, delay in diagnosis and management are crucial for defining the need for specialized services. Available literature is limited in South and sub-Saharan Africa. Major challenges include lack of awareness; lack of trained staff, facilities and support services.^1^ Education in Paediatric Rheumatology is neglected at undergraduate and postgraduate levels with a shortage of paediatric rheumatologists.^1^ Tygerberg Hospital offers a dedicated clinical service since 1995 with an accredited subspecialty training post since 2005.

**Objectives:** The primary aim is to document demography, disease profile and medical management of patients attending the Tygerberg Paediatric Rheumatology clinic. The secondary aim, based on these findings, will address the need of specialized services and subspecialty training posts.

**Methods:** Retrospective folder review, electronic data search and use of bivariate and multivariate statistical tools used for analysis to outline the disease profile of JIA (Juvenile Idiopathic Arthritis), other autoimmune (AI) or auto-inflammatory diseases and miscellaneous musculoskeletal conditions and management thereof.

**Results:** 450 patients were reviewed between March 1995 and March 2017. Referrals were derived from secondary and tertiary hospitals (68%), general and private practitioners (13%) and 8.2% by primary healthcare centers. 60% of referred patients resided in the greater Cape Town region and 40% in rural-country regions, depending on availability of public health transportation services. Most of the patients (74%) were from a family income bracket of less than 7700 USD per year.

Gender distribution reflected 56% female and 44% male. The most common age of presentation was between 10-14 years (46%) with a range of 0 to19 years. Racial distribution was Mixed Racial 79%, Blacks 16%, Whites 4% and Asians 1%. JIA (38%) was most common rheumatological condition. Reactive Arthritis (23%), autoimmune and autoinflammatory conditions (11%), vasculitides (2%) and miscellaneous musculoskeletal conditions (39%) e.g. growing pains, Raynaud's, infection related arthritis, malignancies etc. comprised the rest.

Common presenting symptoms were tender joints (71%), stiffness after rest (46%) and swollen joints (44%). Uveitis was present in 9% at initial diagnosis or during follow up. Delay in diagnosis was up to 9 years.

Ibuprofen (42%) was the most commonly used NSAID. Most frequently used DMARD's were methotrexate and hydroxychloroquine. Intra articular steroid injections were frequently indicated and biological therapy accessed by selected patients.

Referral to ancillary services included physiotherapy (51.6%), imaging predominantly X-rays (41%), ophthalmology (47.1%), orthopedics (37%), occupational therapy (27%) and dermatology (16.7%).

46.5% were offered social grants. 14.7% recorded, achieved remission. Remission could not be established in most patients due to high rate of patients lost to follow up (50%). 3.5% transitioned to adult Rheumatology services.

**Conclusion:** Patients reviewed were mainly of mixed racial origin from low-income homes. JIA was the most common paediatric rheumatological condition seen. Delay in diagnosis and late referrals were frequent. Increased awareness, education and training, early diagnosis of disease and appropriate funding for these neglected diseases could dramatically improve outcome. Addressing challenges unique to a developing country and resultant complexities of management are essential for advocacy and planning of such services.

**Disclosure of Interest:** None Declared

### P98 Raynaud phenomenon secondary to the use of illicit drugs

Abstract withdrawn

### P99 Unilateral recurrent ear chondritis in spondylarthritis does not always progress to polychondritis

#### Laura Damian^1,2^, Simona Rednic^2,3,4^, Cristina Pamfil^5,6^, Linda Ghib^3^, Maria-Magdalena Tamas^3^, Siao-Pin Simon^2,5^, Calin Lazar^7^, Laura Muntean^5^, Alma Maniu^3,8^

##### ^1^Rheumatology, Centre for Rare Musculoskeletal Autoimmune and Autoinflammatory Diseases, Cluj-Napoca, Romania; ^2^Emergency Clinical County Hospital, Cluj-Napoca, Romania; ^3^Iuliu Hatieganu University of Medicine and Pharmacy, Cluj-Napoca, Romania; ^4^Centre for Rare Musculoskeletal Autoimmune and Autoinflammatory Diseases, Cluj-Napoca, Romania; ^5^Rheumatology, Iuliu Hatieganu University of Medicine and Pharmacy, Cluj-Napoca, Romania; ^6^Centre for Rare Musculoskeletal Autoimmune and Autoinflammatory Diseases, Emergency Clinical County Hospital, Cluj-Napoca, Romania; ^7^Pediatrics, Emergency Clinical County Hospital, Cluj-Napoca, Romania; ^8^ENT, Emergency Clinical County Hospital, Cluj-Napoca, Romania

###### **Correspondence:** Laura Damian

**Introduction:** Ear perichondritis can be caused by trauma, relapsing polychondritis (RP), vasculitis, infections etc. Irrespective of etiology, cartilage deformity and necrosis may occur. Chondrodermatitis nodularis helicis (Winkler’s disease) is a localized cartilage inflammation, often with crusting, resulting from trauma or bland vascular impaiment.

**Objectives:** Long-term assessment of recurrent unilateral perichondritis in patients with systemic diseases, for the development of RP diagnostic criteria.

**Methods:** We retrospectively identified the cases of unilateral recurrent ear perichondritis in the patients with suspected polychondritis in the last 15 years in a tertiary referral adult Rheumatology department. The patients then fulfilled a questionnaire and were seen prospectively for two years. All chondritis episodes and the Mc Adam and Damiani-Levine polychondritis criteria were assessed. Laboratory tests for inflammation, immunology, uric acid, ENT examination, cartilage biopsy whenever accepted and an extensive search for infection,vasculitis, hematologic malignancies, as well as complications of other organs classically involved by polychondritis were recorded, as part of the regular screening for polychondritis.

**Results:** We identified 3 cases of recurrent unilateral chondritis, all occuring in spondylarthritis patients, that were followed up for 3 to 12 years for other polychondritis features, without development of RP criteria. None of these patients developed ulcerations or crusting to suggest Winkler’s disease, either.

Case 1: A 26-years female patient with a juvenile extensive oligoarthritis since the age of 5 and psoriasis since 11, on methotrexate, presented with recurrent episodes of right ear swelling and inflammation, healing within three days, not altered by empiric antibiotic short courses, accompanied by transient mild leukocytosis with neutrophilia. An active follow-up for 12 years did not reveal other chondritis sites. NSAIDs helped to alleviate the attacks, which became less frequent with time.

Case 2: A 48-year male patient with aseptic abscesses syndromes, Crohn’s disease and spondylarthritis with bilateral coxitis diagnosed for 3 years had a recurrent left ear upper lobe inflammation since the first visit. The nodular chondritis, with low-level local inflammation generally lasting for one week, independent from the joint flares, responded to the addition of NSAID and sulfasalazine to adalimumab and azathioprine. No progression to polychondritis was noted in 3 years.

Case 3: A patient with undifferentiated spondylarthritis and a history of treated medullar thyroid carcinoma presented with recurrent right ear painful inflammation. No signs of tumoral dissemination or relapse were detected in 6 years. The cartilage biopsy was normal. The attacks' frequency decreased after leflunomide.

**Conclusion:** Unilateral recurrent ear perichondritis may not always herald polychondritis, at least in spondylarthritides. It can be speculated that local triggers (pressure on the ear, etc) may activate the innate immunity through danger-associated molecular patterns, however, the progression to polychondritis probably requires activation of certain Toll-like receptors. An active search for an RP is nevertheless worthed in the unilateral chondritis, as RP requires a more aggressive therapy. Informed consent to publish has been obtained from the patient/parent/guardian.

Funding:PN-II-RU-TE-2014-4-2708

**Disclosure of Interest:** None Declared

### P100 Evaluation of the dental and temporomandibular joint status in children with generalized joint hypermobility

#### Ferhat Demir^1^, Tamer Tüzüner^2^, Özgül Baygın^2^, Mukaddes Kalyoncu^1^

##### ^1^Department of Pediatric Rheumatology, Karadeniz Technical University Faculty of Medicine, Trabzon, Turkey; ^2^Department of Pediatric Dentistry, Karadeniz Technical University Faculty of Dentistry, Trabzon, Turkey

###### **Correspondence:** Ferhat Demir

**Introduction:** Generalized joint hypermobility (GJH) is characterized by deficiency of collagen and changes in the proportion of collagen subtypes. It is a clinical feature associated with excessive laxity of joints. Oral mucosa, teeth and temporomandibular joint (TMJ) can be affected in these condition.

**Objectives:** It was aimed to evaluate the dental and temporomandibular status in children with GJH.

**Methods:** Sixty two children with GJH and age-sex match sixty two healthy children as a control group were enrolled to the study. The GJH was assessed by the Beighton Hypermobility Score (BHS). The subjects screened for dental and TMJ status. Assesment included index for ‘Decayed’, ‘Missing’ and ‘Filled Teeth’ (DMFT), visual plaque index (VPI) and gingival bleeding index (GBI) scores, tooth mobility and TMJ evaluation. The Mann–Whitney U and chi-square tests were used to evaluate the data.

**Results:** Mean BHS score was found 6.3 ± 1.2 in GJH group. VPI and GBI scores were found significantly higher in children with GJH then control group (p < 0.05). No differences were found regarding the DMFT scores between GJH and control groups (p > 0.05). Temporomandibular disease (TMD) frequency was significantly higher in children with GJH compared to the control group (p < 0.001) (Table 34). The most frequent TMD obtained as clicking while maximum active mouth opening. By the way, combined TMJ problems observed in approximately one third of the GJH children.

**Table 34:** **(Abstract P100).** Comparison of dental status, MT and TMD between in GJH and control groups

	GJH group	Control group	p
Age (median (min-max))	8 (5-17)	9 (5-17)	0.43*
Gender (M/F)	36/26	36/26	0.57^#^
VPI positivity (%)	88.7	74.2	0.03^#^
GBI positivity (%)	64.5	45.2	0.02^#^
DMFT score (median(min-max))	0.062 (0-0.23)	0.080 (0-0.23)	0.27*
MT positivity (%)	3.2%	1.6%	0.50^#^
TMD positivity (%)	48.4%	14.5%	<0.001^#^

*Mann-Whitney U test and #Chi-square test was used for analysis. VPI: Visual plaque index, GBI: Gingival bleeding index, DMFT: decayed, missing and filled teeth, MT: Mobility of tooth, TMD: temporomandibular disease.

**Conclusion:** High frequency of TMD in GJH group may demonstrate that TMJ is also affected in GJH. The higher VPI and GBI scores indicating the poor oral hygiene in children with GJH may point out that, this children can not perform properbly toothbrushing.

**Trial registration identifying number:** Ethical approval was obtained from the Ethics Committee of Karadeniz Technical University, Faculty of Medicine (Approval number: 2016/102).

**Disclosure of Interest:** None Declared

### P101 Successful treatment of pediatric IGG4 related ophthalmic disease with mycophenolate mofetil: case report and a review of the pediatric autoimmune dacryoadenitis literature

#### Talia Diaz, Yuridiana Ramirez, Sofia Osorio, Maria Teresa Braña, Luis Aparicio, Andres Rodriguez, Enrique Faugier, Rocio Maldonado

##### Pediatric Rheumatology, Hospital Infantil de Mexico Federico Gomez, Ciudad de Mexico, Mexico

###### **Correspondence:** Talia Diaz

**Introduction:** IgG4-related disease (IgG4-RD) is relatively a new growing entity of immune mediated origin, characterized by a unique pathological feature that affect a wide variety of organs with infiltration of IgG4-positive plasma cells and occasionally elevated serum IgG4. It is both a systemic inflammation and a sclerosing disease. The most common manifestations are parotid (14-26%) and lacrimal swelling (4-13%), lymphadenopathy and autoimmune pancreatitis. Several studies have examined orbital adnexal IgG4-related disease or IgG4-related orbital inflammation. The diagnosis should be proven histopathologically but other conditions such as lymphoma should be carefully excluded. Patients with IgG4-RD respond beneficially to glucocorticoid therapy especially when administered at early onset stages. In some cases, the combination of immunosuppressive agents is required.

**Objectives:** To describe a pediatric clinical case with orbital IgG4 related disease with an excellent response after immunosuppresive treatment.

**Methods:** We present a 16-year-old girl with IgG4-RD during one year with left ocular pain and eye inflammation, diagnosed as bacterial conjunctivitis and trated with ophtlamic antibioticoteraphy, with no improvement. Weight loss and increased acute phase reactants (erythrocyte sedimentation rate and C-reactive protein). On ocular computed tomography. Histopathology clearly defined the mass to be IgG4 related fibrosis with focally arranged fibrosis in a storiform pattern, and positive CD20 deposit ++++, positive IgG4 +++ in plasmatic cells and lymphocytes, positive CD38 deposit ++. Biopsy report: Autoimmune Dacryoadenitis Associated with IgG4 Disease. IgG4 serum normal level 9 mg/dl.

Ocular Magnetic resonance imaging was performed in which volume and lacrimal gland intensity is identified, which displaces the rectus and oblique muscle causing proptosis, the lacrimal gland presented an increase in it’s dimensions with measures of 23x8x10mm in its posterior axes, posterior No abnormal reinforcement is identified in the contrast medium. Infectious serological test were negative (EBV, CMV, HBV, HCV, toxocara and toxoplasma).

**Results:** The patient was started on oral corticosteroids and mycophenolate mofetil, which resulted in significant clinical improvement after 3 months of treatment, the mass became smaller and the patient was asymptomatic.

**Conclusion:** IgG4-RD remains a field not yet fully explored or understood. Early and efficient therapies aiming to achieve a beneficial outcome, and to improve prognosis are still required.

**Disclosure of Interest:** None Declared

### P102 Rheumatic fever and post-streptococcal arthritis in a tertiary hospital from Paraguay.

#### Jorge Garcete^1^, Luz Galeano^1^, Carla Montiel^2^, Dong Chin^2^, Maria del Carmen Cabrera^2^, Milagros Vargas Peña^2^, Carlos Verón^2^, María Lezcano^3^, Alicia Aldana^3^, Ana Campuzano de Rolón^1^, Zoilo Morel Ayala^4^

##### ^1^Pediatric Service, Hospital de Clinicas. Facultad de Ciencias Medicas.Universidad Nacional de Asuncion., San Lorenzo, Paraguay; ^2^Pediatric Cardiology, Hospital de Clinicas. Facultad de Ciencias Medicas.Universidad Nacional de Asuncion., San Lorenzo, Paraguay; ^3^Pediatric Neurology, Hospital de Clinicas. Facultad de Ciencias Medicas.Universidad Nacional de Asuncion., San Lorenzo, Paraguay; ^4^Pediatric Rheumatology, Hospital de Clinicas. Facultad de Ciencias Medicas.Universidad Nacional de Asuncion., San Lorenzo, Paraguay

###### **Correspondence:** Jorge Garcete

**Introduction:** Rheumatic fever (RF) and post-streptococcal arthritis (PSA) are characterized by autoantibodies secondary to beta hemolytic streptococcus group A infection. In our country, the cardiac sequelae acquired by the RF generate substantial costs for the State and family, becoming a public health problem.

**Objectives:** To assess the clinical characteristics and outcome of patients with RF and PSA from a Paraguayan referral hospital.

**Methods:** Retrospective and descriptive study of patients under the age of 18 years, diagnosed with RF and PSA between January 2009 and April 2017.

**Results:** 32 cases of RF and 8 PSA (all misdiagnosed as originally RF) were found. The population is distributed similarly by all states of the Eastern Region from Paraguay. The average monthly income per household is 18% with 2 minimum wages, 37% with a minimum salary, 30% less than 1 minimum wage, and 15% without data. 62% of children with RF sleeps with 3 or more family members in the same room. Mean age at diagnosis: 10.5 (3-17) years. The female to male ratio is 4:6. Only 5% are Native patients. During the acute phase, 52% had polyarthritis, 43% had carditis, 13% had chorea, 2% had subcutaneous nodules, and 2% had erythema marginatum. Jones minor criteria: arthralgia 20%, fever 14%, elevated ESR 21%, CRP positive 16%, EKG altered 13%. The most affected heart valve is the mitral (45%), followed by mitral and aortic simultaneously (10%), 8% aortic and 5% tricuspid alone; 32% with no affected heart valves. In 93% of patients not throat culture was done. ASO + in 21% of cases, performed in all patients. Treatment: all received benzathine penicillin G, 35% NSAID, 18% corticosteroids, 10% haloperidol, 14% valproic acid. Eleven patients discontinued treatment. Only 10% of patients with RF has required heart surgery (valve replacement), with one death.

**Conclusion:** RF remains a major cause of acquired heart disease in our country, perhaps by inadequate management of streptococcal infections, in addition to late diagnosis of the disease and household overcrowding.

**Disclosure of Interest:** None Declared

### P103 Immunoglobulin therapy in the treatment of cerebral consequences of neonatal lupus erythematosus

Abstract withdrawn

### P104 Validaty and reliability study for the Korean version of the haemophilia activity list in pediatric and adult patients with haemophilic arthropathy

#### Seonghoon Park, Hwajeong Lee, Jung-Yoon Choe

##### Department of mediciane, Catholic university of Daegu School of medicine, Daegu, Korea, Republic Of

###### **Correspondence:** Seonghoon Park

**Introduction:** There has been increasing interest in the patient’s perspective on outcome of treatment. The Haemophilia Activity List (HAL) has been developed as a disease-specific questionnaire for haemophilia patients and is a validated self-report measure of function developed according to WHO’s International Classification of Functioning, Disability and Health.

**Objectives:** In this study, we performed a cross-cultural adaptation and linguistic validation of the HAL questionnaire to assess the health-related quality of life in hemophilia patients in the future.

**Methods:** To validate HAL in Korean, the English versions of HAL were translated into Korean using ‘the forward–backward translation’ method and merged into a final Korean version. Validation was performed against the Korean version of the multidimensional health assessment questionnaires (MDHAQ) as general tool and Routine Assessment of Patient Index Data (RAPID3) as similar arthritis-specific tool. All processes were done with permission of the developer and according to WHO guidelines.

**Results:** Eighty-six patients with severe and moderate forms of haemophilia A and B with haemophilic arthropathy were invited to participate in the study. Spearman’s rank correlation test was used for validation and internal consistency of the HAL was calculated with Cronbach’s alpha. Seventy-five patients (86.2%) (15–62 years old) answered the questionnaires. The internal consistency of the Korean version of HAL was high, with Cronbach’s alpha being 0.80–0.95. Upper extremity function had the highest consistency and leisure activities and sport had the lowest. The correlation was good between the HAL overall score and MDHAQ overall (r = 0.78), MDHAQ pain (r = 0.79), and RAPID3 physical function (r = 0.82).

**Conclusion:** The Korean version of HAL has both internal consistency and convergent validity and may complement other functional tests to gather information on the patient’s self-perceived ability. This questionnaire of Korean version can be useful as a disease-specific instrument for evaluation of the health-related quality of life in Korean patients with haemophilic arthropathy.

**Disclosure of Interest:** None Declared

### P105 Inflammatory pseudotumor: T helper cell subtypes and relation to IGG4-related disease?

#### Erdal Sag^1^, Gulsev Kale^2^, Beril Talim^2^

##### ^1^Pediatric Basic Sciences, Pediatric Autoinflammatory Disease Programme, Hacettepe University Faculty of Medicine, Institution of Child Health, Ankara, Turkey; ^2^Department of Pediatrics, Pathology Unit, Hacettepe University Faculty of Medicine, Ankara, Turkey

###### **Correspondence:** Erdal Sag

**Introduction:** IgG4-related disease is a fibroinflammatory condition characterized by tumefactive lesions, dense lymphoplasmacytic infiltrate rich in IgG4-positive plasma cells, storiform fibrosis and often elevated serum IgG4 concentration. On microscopic examination, presence of IgG+ plasma cells (>10-50/HPF), IgG4+/IgG+ plasma cell ratio (>50%) and typical histopathologic pattern confirm the diagnosis. Inflammatory pseudotumor (inflammatory myofibroblastic tumor) is a mass lesion composed of myofibroblasts, plasma cells, lymphocytes and eosinophils.

**Objectives:** The aim of this study is to analyze the pediatric patients with inflammatory pseudotumor (inflammatory myofibroblastic tumor) of various organs for the diagnosis of IgG4 related disease, and to investigate the composition of T helper subsets.

**Methods:** 20 patients who were diagnosed as inflammatory pseudotumor (inflammatory myofibroblastic tumor) of various organs at Hacettepe University Faculty of Medicine, Department of Pediatrics, Pathology Unit were included in the study. Immunohistochemical expression of IgG, IgG4, CD3, CD20, CD68, IL-10, IL-17, Foxp3, IFN-gamma, IFN-alpha and IL-4 were studied from archived paraffin blocks.

**Results:** The mean age of the patients at the time of biopsy was 7,36 ± 3.85 yr. The biopsies were taken from orbita (2 patients), mediastinum (7 patients; 3 from lungs, 3 from mediastinal wall, 1 from bronchi), and abdomen (10 patients; 2 liver, 1 spleen, 2 intestinal, 4 intraabdominal, 1 retroperitoneal wall). Out of the 20 patients, 3 had more than 50 IgG4+ cells per HPF but none of them did fulfil the diagnostic criteria for IgG4-related disease. In terms of inflammatory milieu, all the biopsies were positive for CD3, CD20 and CD68. As for T helper cell subtypes, all of the biopsies were IL-10 positive, 85% of the biopsies were IL-4 positive, 95% were IL-17 positive, 80% were Foxp3 positive, 50% were IFN-alpha and 35% were IFN-gamma positive.

**Conclusion:** In our cohort, none of the inflammatory pseudotumors (inflammatory myofibroblastic tumors) fulfilled the diagnostic criteria for IgG4-related disease. B cells, macrophages and T cells are present in the inflammatory pseudotumor and all T helper cell subtypes such as Th1, Th2, Th17 and Treg are included in the inflammatory milieu.

Disclosure of Interest: None Declared

### P106 Hyperbaric oxygen therapy in a livedoid vasculopathy: a case report

#### Nihal Sahin^1^, Aysenur Pac Kisaarslan^1^, Sümeyra Ozdemir Cicek^1^, Betül Sözeri^2^, Mehmet E. Akcin^3^, Zubeyde Gunduz^1^, Ruhan Dusunsel^1^, Muammer H. Poyrazoglu^1^

##### ^1^Pediatric Rheumatology, Erciyes University Faculty of Medicine, Kayseri, Turkey ^2^Pediatric Rheumatology, Umraniye Research and Training Hospital, İstanbul, Turkey ^3^Underwater and Hyperbaric Medicine, Kayseri Research and Training Hospital, Kayseri, Turkey

###### **Correspondence:** Nihal Sahin

**Introduction:** Livedoid vasculopathy is characterized disease with recurrent ulceration and thrombosis. Its pathogenesis has been found to be related to occlusion of the cutaneous microcirculation leading to thrombosis, ischemia and minimal inflammation in the vascular wall. There is still no definitive treatment for this disease. Hyperbaric oxygen therapy (HBO) facilitates wound healing by affecting the fibrinolytic pathway. We used HBO in a patient resistant to other treatments.

**Methods:** A girl aged 15 years was admitted to the Erciyes University Faculty of Medicine Pediatric Rheumatology Department at the first time in May 2016 complaining of painful ulceration in the ankles and foot.

**Results:** A fifteen-year old female presented with a one year- old history of recurrent multiple painful ulcer, purpuric macule and porcelain – white stellate scars on the ankle and the foot. Patient did not give any history of any other medical illness, or any systemic complaints. There was no remarkable feature in baseline laboratory examinations. Doppler ultrasonography of the lower extremities did not find any vascular insufficiency in the major vessels. Skin biopsy from the lesion site revealed featured of focal necrosis of the epidermis, subepithelial focal hemorrhage, hyalinization of the capillary walls and thrombosis in some vessels. The tests containing C3, C4, RF, ANA, ANCAs, ACA IgM and IgG, lupus anti-coagulant, anti-thrombin III, protein C, protein S, homocysteine were normal. Factor V Leiden, prothrombin and methylenetetrahydrofolate reductase (MTHFR) gene mutation analysis were performed. Heterozygous mutation was detected in nucleotide region 1298 of the MTHFR gene. Anti-platelet, immunosuppressive, immunomodulatory drugs and systemic steroid were given to the patient with diagnosis of livedoid vasculopathy. Hyperbaric oxygen therapy was applied on frequent relapses with these treatments. The patient completely relieved from pain and ulcers showed marked improvement.

**Conclusion:** Treatment of livedoid vasculopathy is very challenging due to low incidence and unclear pathogenesis. Hyperbaric oxygen therapy is well tolerated, with few side-effects compared with the other treatment regime. We treated resistant livedoid vasculopathy with hyperbaric oxygen therapy.

**Disclosure of Interest:** None Declared

### P107 Echocardiographic findings in children with osteogenesis imperfecta

#### Shahrzad Fallah^1^, Mohammad Reza Alaee^2^, Reza Shiari^3^, Saeed Mojtahed zadeh^2^, Behnam Sobouti^4^

##### ^1^pediatrician, Shahid Beheshti Univesity of Medical Sciences Tehran, Iran, Islamic Republic Of’; ^2^Pediatric Cardiologist, Shahid Beheshti Univesity of Medical Sciences Tehran, Iran, Islamic Republic Of; ^3^Pediatric Rheumatology, Shahid Beheshti Univesity of Medical Sciences Tehran, Iran, Islamic Republic Of; ^4^pediatrician, Iran Medical Universuty, Tehran, Iran, Islamic Republic Of

###### **Correspondence:** Reza Shiari

**Introduction:** Osteogenesis imperfecta, an inherited connective tissue disorder with remarkable clinical variability, is caused by a quantitative or qualitative defect in collagen synthesis. Exact prevalence of cardiac involvement in Osteogenesis imperfecta is indistinct.

**Objectives:** The aim of this study was to determine the prevalence of cardiac involvement in children with Osteogenesis imperfect by Echocardiography.

**Methods:** During one year, Sixty five patients were referred to Out-patient Clinic of Musculoskeletal and endocrinology in Mofid Children’s Hospital. All of them were consulted with our Pediatric Cardiologist and echocardiography was performed.

**Results:** Among 65 patients (60% male, 40% female), mean age was 6.95 ± 4.36 year-old (ranging from 2 months to 17 years old). Twenty percent of patients were belong to subtype one, 47.7% to subtype III, and 32.3% to subtype IV according to silence category.

Prevalence of aortic root dilatation, mitral valve prolapse (MVP), mitral regurgitation (MR), and tricuspid regurgitation (TR) were 13.8%, 15.38%, 7.6%, and 15.38% respectively. We found a significant relationship between MVP and female gender, however, no relation with patient’s subgroups.

**Conclusion:** Because of the importance of cardiac involvement in our study, we suggest to perform echocardiography in all children with osteogenesis imperfecta.

**Disclosure of Interest:** None Declared

### P108 Acid ceramidase deficiency (Farber disease) causes symptoms resembling JIA and is likely underdiagnosed: we present a unique cross-sectional natural history study design to address the lack of clinical data on this rare disease

#### Alexander Solyom^1^, Boris Hügle^2^, Dustin Tetzl^3^, Edward H. Schuchman^4^

##### ^1^Enzyvant, Basel, Switzerland; ^2^German Center for Pediatric Rheumatology, Garmisch-Partenkirchen, Germany; ^3^Enzyvant, New York, NY, United States; ^4^Genetic and Genomic Sciences, Icahn School of Medicine at Mt. Sinai, New York, NY, United States

###### **Correspondence:** Alexander Solyom

**Introduction:** Acid ceramidase deficiency (Farber disease; Farber lipogranulomatosis) is caused by mutations in both alleles of the *ASAH1* gene, resulting in deficiency of acid ceramidase and accumulation of the pro-apoptotic and pro-inflammatory sphingolipid, ceramide. It is considered an ultra-rare disease, with only 109 cases reported in the medical literature since 1952. However, since 2014, we have identified a cohort of 45 patients (some of whom are since deceased) from 30 countries on 6 continents, which indicates a much broader phenotypic spectrum and potentially higher incidence than previously thought. The information collected from this cohort demonstrates that there are patients diagnosed in late adulthood with attenuated forms, as well as patients for whom years elapse between the appearance of the three typical Farber symptoms: joint disease (arthritis and/or contractures), subcutaneous nodules, and hoarseness (due to laryngeal involvement). Moreover, there is wide intra- and inter-patient variability of symptom severity.

**Objectives:** To develop a natural history study structure and design that would enable systematic collection of information on deceased patients using medical records, as well as on living patients who have or have not undergone hematopoietic stem cell transplantation (HSCT). In addition, the study will evaluate clinical assessment tools used for other diseases with similar symptoms, as well as those developed specifically for Farber disease.

**Methods:** We designed a cross-sectional cohort study with a unique data collection instrument, including sections for the collection of retrospective data on disease-specific and general medical history, as well as data from clinical assessments to be performed at prospective visits.

**Results:** The pre-existing clinical assessment tools include (among others) the Childhood Health Assessment Questionnaire (CHAQ), and Wong-Baker Faces Pain Rating Scale, 6-minute Walk Test, Pulmonary Function Testing, and Joint Range of Motion measurements. We also developed a unique patient reported outcome measure related to Farber disease symptoms including physical impairment, pain, and emotional distress, and a method for the assessment of change in size of subcutaneous nodules.

**Conclusion:** Consideration of the broad spectrum of ages and heterogeneous phenotypes in a small population of patients available to participate in a study of the natural history of acid ceramidase deficiency led to the design of a cross-sectional cohort study with retrospective and prospective components. By including deceased patients and patients having undergone HSCT in the study design, the amount of data that can contribute to the understanding of this very rare disease is substantially increased. Furthermore, the specific prospective assessments will allow us to follow disease progression over time, and to evaluate the degree to which the assessment tools and techniques may be appropriate measures to use to register change in certain symptoms. It is our hope that this unique design will provide data which is sufficiently robust to serve as the basis for preventing misdiagnosis of acid ceramidase deficiency as JIA, provide an indication of the most appropriate methods for measuring efficacy in future therapeutic trials, and serve as a potential point of reference in the design of similar studies in rare disease populations.

**Disclosure of Interest**: A. Solyom Employee of: Enzyvant, B. Hügle: None Declared, D. Tetzl Employee of: Enzyvant, E. Schuchman Shareholder of: Enzyvant

### P109 Involving patients, parents and carers in paediatric rheumatology research: best practices examples from lay representatives of the United Kingdom's clinical studies group

#### Simon Robert Stones^1,2^, Catherine Wright^2,3^ on behalf of Lay/Consumer CSG Representatives

##### ^1^School of Healthcare, University of Leeds, Leeds, United Kingdom; ^2^Paediatric Rheumatology CSG, NIHR CRN: Children/Arthritis Research UK, Liverpool, United Kingdom; ^3^Arthritis Care Northern Ireland, Arthritis Care, Belfast, United Kingdom

###### **Correspondence:** Simon Robert Stones

**Introduction:** The lived experiences and opinions of patients, parents and carers (PPCs) living with rheumatic and musculoskeletal diseases (RMDs) must inform and influence all aspects of research, from prioritisation through to dissemination. For example, contributing to grant applications, designing studies, identifying the most suitable outcomes to include in a clinical trial, facilitating study recruitment and sharing findings with the wider community. In turn, it is possible that involving PPCs may enhance expectations and subsequent levels of satisfaction with research.

**Objectives:** The aim of the PPC representatives on the clinical studies group is to provide strategic guidance for the paediatric rheumatology community about the most effective ways of engaging and involving PPCs in clinical and health services research.

**Methods:** To effectively represent PPCs living with RMDs, it is critical to have a means of ensuring that PPC representatives encapsulate the range of views within the PPC community. The paediatric rheumatology clinical studies group PPC representatives aim to do this through networking, linking with external rheumatology stakeholder groups and through hosting consumer research meetings. In addition, PPC representatives comment on various documents as part of their role, including trial protocols, ethics applications and and participant information sheets. They have also been involved in formulating three research questions as part of the clinical studies group research strategy. The top three research priorities were informed by a patient-led research study with young people and their families. With much delight, one research priority has informed the current development of a study to improve treatment tolerability for young people with RMDs.

**Results:** Activities of PPC representatives include attendance several face-to-face and teleconference meetings each year. This is an essential activity that enables researchers and healthcare professionals to actively interact with PPCs as equal partners, in order to shape ongoing and future research activities. This approach also emphasises the importance of physical meetings in fostering collaborative and participatory approaches to research. In addition, PPC represenatives bridge the gap between the research community, patient groups and charities, facilitating communication between individuals and teams.

**Conclusion:** By widely accepting and embracing the PPC voice as a catalyst for high quality, young person- and family-focused research, it is hoped that the often negative experiences of living with an RMD can be used positively to shape research, so as to be able to provide the best possible care, treatment and support for young people and their families living with RMDs in the future.

**Disclosure of Interest:** None Declared

### P110 Defining the clinical impact of symptoms in a diverse population of patients with a rare disease: a qualitative research study in acid ceramidase deficiency (Farber disease)

#### Alexander Solyom^1^, Brendan Johnson^2^, Karoline Ehlert^3^, Dustin Tetzl^4^, Karin Coyne^5^

##### ^1^Enzyvant, Basel, Switzerland; ^2^Roivant Sciences, Durham, NC, United States; ^3^University Medical Center, Greifswald, Germany; ^4^Enzyvant, New York, NY, United States; ^5^Evidera, Bethesda, MD, United States

###### **Correspondence:** Dustin Tetzl

**Introduction:** Acid ceramidase deficiency (Farber disease; Farber lipogranulomatosis) is considered an ultra-rare disease. Since 2014, Enzyvant has identified a cohort of 45 patients from 30 countries on 6 continents, with a much broader phenotypic spectrum and demonstrating a potentially higher incidence than previously thought. The cardinal symptoms of Farber disease are: arthritis, subcutaneous nodules, and hoarseness. Over 70% of the moderate and attenuated phenotype patients in the Enzyvant cohort were initially diagnosed with a form of Juvenile Idiopathic Arthritis.

**Objectives:** Initiate a qualitative research study to understand the symptoms and impact of Farber disease from the perspective of patients and caregivers by conducting one-on-one interviews for concept elicitation, content validity, and patient interpretation of newly developed patient-reported outcome tools.

**Methods:** This study consisted of two parts: semi-structured qualitative interviews to understand Farber disease (FD) symptoms and to evaluate new Farber-specific PRO measures to capture the impact of FD symptoms on patients including: subcutaneous nodules, voice, overall pain, arthritis, ability to move joints, ability to perform daily tasks, and level of fatigue. Participants were asked to rate the symptoms’ impact on a scale of 0–10, with 0 meaning no impact and 10 meaning greatest impact. Additional PROs for benchmark assessment were (among others): Childhood Health Assessment Questionnaire (CHAQ), SF-36 in patients and caregivers.

**Results:** Eight interviews were conducted with 4 caregivers and 4 patients. Included were: 2 adult patients (attenuated phenotype); 1 caregiver of deceased child (severe phenotype); 1 transplanted adult patient (moderate phenotype); 1 caregiver of transplanted pediatric patient (moderate phenotype); 1 caregiver of a pediatric patient (moderate phenotype); and 1 caregiver/patient dyad of a non-transplanted pediatric patient (moderate phenotype). Interviews revealed the symptoms of acid ceramidase deficiency have measurable clinical impact across a broad spectrum of phenotypes and symptom severity. In general, all participants found the questionnaires and assessments relevant to their condition. Feedback was provided by the participants to improve the formulation and scope of questions. With regard to the relative impact of symptoms, the ability to move joints had an average impact rating of 6.6 (range 1 to 10), followed by ability to perform daily activities and subcutaneous nodules with 5.9 (range 1 to 10 for daily activities and 3 to 9 for nodules), voice impact was rated 5 (range of 3 to 9). The remaining symptoms (arthritis, overall pain, level of tiredness, and other symptoms) all yielded lower levels of impact. In the cases where the SF-36 and CHAQ were completed, scores demonstrated significant impact and disability in the population of patients with acid ceramidase deficiency.

**Conclusion:** In spite of the small number of participants, the information gathered provides a better understanding of methods useful for measuring symptom impact, potentially in the context of future therapeutic trials, and may allow better discussion of symptom impact between physicians, patients and caregivers. This type of qualitative study can be particularly useful in rare disease populations, and may also help interpret the utility of both new and established patient reported outcomes in cases when the size and variability of the available population prohibits traditional methods of validation.

**Disclosure of Interest**: A. Solyom Employee of: Enzyvant, B. Johnson Employee of: Roivant Sciences, K. Ehlert: None Declared, D. Tetzl Employee of: Enzyvant, K. Coyne Employee of: Evidera

### P111 A novel homozygous mutation of gene WISP3 in progressive pseudorheumatoid dysplasia

Abstract withdrawn

### P112 Association of juvenile-onset primary Sjögren syndrome with type I C2 deficiency

#### Erika Van Nieuwenhove^1,2^, Lien De Somer^3^, Carine Wouters^3,4^

##### ^1^Center for Brain and Disease Research, VIB/KULeuven, Leuven, Belgium; ^2^Pediatrics, University of Leuven, Leuven, Belgium; ^3^Pediatric Rheumatology, University of Leuven, Leuven, Belgium; ^4^Department of microbiology and immunology, KULeuven, Leuven, Belgium

###### **Correspondence:** Erika Van Nieuwenhove

**Introduction:** Sjögren syndrome (SS) is a systemic autoimmune disease characterized clinically by sicca syndrome resulting from lymphocytic infiltration of the exocrine glands.

**Objectives:** To identify genetic susceptibility factors in juvenile-onset primary Sjögren syndrome through whole exome sequencing.

**Methods:** Paired-end sequencing was performed on genomic DNA on an Illumina HiSeq 2500 (Genomics Core Facility). Burrows-Wheeler Aligner software was used for alignment to Genome Build hg19, GATK Haplotype Caller for base calling and ANNOVAR software for annotation. Identified variants were confirmed by Sanger sequencing (LGC Genomics Facility, Berlin, Germany).

**Results:** A girl (P1) aged 14 years was born the second of four children to consanguineous parents of Moroccan origin. In the familial history we note beta thalassemia minor in father and index patient and type 1 diabetes in the father. Immunological screening was prompted by two occurrences of ethmoiditis with orbital cellulitis and progression to an orbital abscess in the first episode, before the age of 4 years. However, no notable infectious episodes recurred. Laboratory analysis revealed isolated IgG2 deficiency (0.55 g/L; 0.72-3.40), very low memory B cells (1.8%) and a low response to 1/3 pneumococcal serotypes tested. At the age of 9 years, she presented with recurrent transient episodes of fever and arthritis in her knees and ankles. Laboratory analysis showed clear immune activation with hypergammaglobulinemia (IgG 24.40 g/L; 5.30-13.06), mildly elevated sedimentation (26 mm/h) and positivity for rheumatoid factor (RF) (519 IU/ml; ≤40) and anti-Ro autoantibodies (>282.0; ≤7.0) (Table 1), suggestive for SS. In addition, increased CD5+ B cells (31.4%), glutamic acid decarboxylase (GAD) autoantibodies (4.2; ≤0.9), low total complement levels (<20%; 70-140) and undetectable levels of C2 were found (<7 mg/L; 14 – 25). Symptoms persisted and at the age of 10 years she developed cutaneous vasculitis of the legs, feet, hands and eyelids. Furthermore, she developed sicca symptoms of the eyes and mouth, secondary caries, hoarseness and a persistent elevation of amylase (214U/L; 28-100). Magnetic resonance imaging (MRI) demonstrated mild enlargement of the right parotid gland, which appeared lightly inhomogeneous with T2-hyperintesive foci and a limited number of intraglandular lymph nodes, suggestive for parotitis. Due to persistent disease activity at the age of 12 years, she was started on treatment with daily administration of 200 mg Plaquenil. While hypergammaglobulinemia and sicca symptoms persist, sustained remission of vasculitis and arthritis ensued. Whole exome sequencing revealed P1 is homozygous for the classic deletion in *C2* causal for type I C2 deficiency.

**Conclusion:** The presentation of primary SS at this age is very rare and P1 displayed both typical clinical (vasculitis, arthritis, parotitis) and laboratory (hypergammaglobulinemia, anti-Ro autoantibodies, RF positivitiy, low memory and high circulating CD5+ B cells) features. To the best of our knowledge C2 deficiency, the most frequent hereditary deficiency in the classical component pathway, was never reported in association with SS. One study on adult pSS detected low CH50 in 15% of patients and hypocomplementaemia correlated with systemic symptoms^1^. Unlike deficiency for the C1 complex or C4, the penetrance of C2 deficiency in SLE is merely 10%^2^. However, since the majority of C2 deficient patients do not manifest autoimmunity there are likely other genetic or environmental drivers for disease. C2 deficiency may be a negative modulating factor on the development and course of autoimmune disease and screening should be considered in patients with primary SS.

References

1. Ramos-Casals, M. *et al.* Hypocomplementaemia as an immunological marker of morbidity and mortality in patients with primary Sjögren’s syndrome. *Rheumatology* **44,** 89–94 (2005).

2. Lintner, K. E. *et al.* Early components of the complement classical activation pathway in human systemic autoimmune diseases. *Front. Immunol.* **7,** 1–22 (2016).

**Disclosure of Interest:** None Declared

## Psycho-social aspects and rehabilitation

### P113 Introducing RAiISE - raising awareness of invisible illnesses in schools and education

#### Sophie Ainsworth^1^, Jenny Ainsworth^1^, Jennifer Preston^2^, Simon Stones^1^, Robyn Challinor^1^, Marie Rowe^1^

##### ^1^RAiISE, Liverpool, United Kingdom; ^2^Patient and Public Involvement, Alder Hey Children's Foundation Trust, Liverpool, United Kingdom

###### **Correspondence:** Sophie Ainsworth

**Introduction:** RAiISE is a user-led research project inspired by the negative experiences that young people face while studying and living with an invisible illness. Many young people who live with chronic illnesses look no different to their healthy peers. The invisible nature of some illnesses can often lead to an invisible struggle, leading to misunderstandings, particularly in the case of young people. It can be a huge burden on the chronically ill to make the invisible, visible to others.

**Objectives:** The main objective of RAiISE is to improve the standard of care given to young people with invisible illnesses in school, college and university and to create a resource to teach education professionals a series of strategies and techniques to support their students. RAiISE will also offer support to young people with invisible illnesses and aim to empower them to take control of their own health.

**Methods:** A young patient of Alder Hey NHS Children's Foundation Trust decided to raise awareness of living with an invisible illness. A network of young people, parents, education and health professionals was created and a series of workshop and focus groups allowed each stakeholder to share their experiences and expertise as they inspired and advised the production of the RAiISE information pack. It is important that young people are able to shape research based on their lived experiences. Several international charities and organisations have offered support and knowledge in advising the process.

**Results:** At early workshop meetings, young people with invisible illnesses and their parents were able to offer personal accounts and experiences which highlighted that the most common themes were problems with communication and trust, as well as difficulty in understanding the erratic nature of many chronic illnesses. From this research, a draft information pack was written by the RAiISE committee, which was later presented to young people, parents, health and education professionals and charity representatives. All stakeholders were able to offer their expertise from their respective fields. Feedback was overwhelmingly positive and any adjustments are to be made in the coming weeks. The final pack will be completed and ready for distribution by the end of summer 2017.

**Conclusion:** The project has been a successful example of young patient led research and highlights the importance of self-management in young people living with invisible chronic illnesses. The collaboration between young people, parents, and education and health professionals has highlighted the necessity for cooperation between all stakeholders for the benefit of the young person.

**Disclosure of Interest:** None Declared

### P114 Development and feasability of a shared management tool for school children with juvenile idiopathic arthritis

#### Jeannette H. Cappon^1^, Bianca Knoester^1^, Marion A. van Rossum^2^

##### ^1^Paediatric Rehabilitation, Reade, center for Rheumatology and Rehabilitation, Amsterdam, Netherlands ^2^Paediatric Rheumatology, Amsterdam Rheumatology and Immunology Center |Reade, Amsterdam, Netherlands

###### **Correspondence:** Jeannette H. Cappon

**Introduction:** Health related quality of life (HRQOL) can be severely affected in children with juvenile idiopathic arthritis (JIA). Earlier studies have shown that, amongst other factors, school absence is one of the main predictors of HRQOL in children with JIA[i]. HRQOL is also related to pain intensity and coping strategies.[ii] In young JIA patients (aged 4–9 years) school functioning can be impaired due to pain, fatigue and limited joint function. Adequately coping with these symptoms of JIA during schooldays demands a shared management approach between the young child, parent(s), teacher and health professionals. An instrument supporting young children with JIA in managing their symptoms during schooldays in a structured way was lacking.

**Objectives:** 1. To develop an instrument to support a young child with JIA managing symptoms during schooldays.

2. Implementation and evaluation of the feasability of the developed shared management tool in the rehabilitation program.

**Methods:** 1. Elements necessary in the instrument are elected after consulting parents of young children with JIA and health professionals of Reade multidisciplinary team for children with JIA. With help of a professional designer an instrument is developed to provide a shared management tool for managing symptoms during schooldays.

2. Children with JIA (aged 4–9 years) with problems in school attendance and their parents are informed about the instrument and invited to use it during their rehabilitation program in Reade. Parents, children and health professionals evaluate the feasability after using the instrument by filling in a questionnaire.

3. Results of questionnaires are discussed in the multidisciplinary team for evaluation.

4. Improvement points for the instrument are proposed.

**Results:** 1. A personal diary called “Back and Forth Booklet” is designed which contains (1) education pages for the child, parents and the teacher about JIA, pain and energy levels and how to manage these, (2) a daily pain measure instrument for location and amount of pain for the child (colour-in pain puppet), (3) a level of energy instrument for expressing fatigue (image of full/half full/empty glass) (4) space for registration of appropriate alternatives for limited activities by the health professionals or parents, (5) daily feedback spaces for the teacher and (6) a visual analogue (VAS) Likert smiley scale for daily self-evaluation by the child.

2. 9 children and their parent(s), 3 occupational therapists, 3 physical therapists, 1 social worker, 1 psychologist and 9 teachers have used the Back and Forth Booklet. Two parents, two children, one teacher and seven health professionals completed a questionnaire.

3. Evaluation of the questionnaires so far showed that the use of the Back and Forth Booklet contributes in communication about the child’s pain and fatigue among the child, parents, teachers and health professionals. The Back and Forth Booklet facilitates schoolteachers in supporting a child with tailored pain and fatigue coping strategies. All users were satisfied with the design. Children appreciated the colour-in pain puppet for not having to explain verbally the teacher about their pain.

4. Items for improvement were: (1) Parents need open space for sharing daily information with the teacher (2) Occupational therapists suggest extra space for documentation of appropriate alternatives for limited activities.

**Conclusion:** The “Back and Forth Booklet” is a promising shared management tool, supporting young children to cope with JIA symptoms in school. Small adjustments can improve the feasability.

[i] Haverman et al A&R 2012; 64(5)694-703

[ii] Sawyer et al Rheumatology 2004;43:325-330

**Disclosure of Interest:** None Declared

### P115 A smiling childhood: a social window for families with children affected by chronic rheumatics and rare pathologies

#### antonella celano, on behalf of apmar onlus, raffaella arnesano, annalisa sticchi, on behalf of apmar onlus and apmar onlus

##### Italian national Association people with rheumatic and rare diseases, Lecce, Italy

###### **Correspondence:** antonella celano

**Introduction:** The Smiling Childhood project was launched by APMAR Onlus, supported by the Valdese Church and their generous 8x1000 funds donation.

The project comes from a long journey undertaken by our association in order to be closer to people affected by chronic pathologies during their daily lives, defining strategies of inclusion in order to create an understanding atmosphere about Rheumatic Pathologies, who unfortunately are still not very well known nowadays.

Some of these pathologies are diagnosed at a later stage, resulting in a late treatment and in some cases in a great degrade in self-sufficiency.

**Objectives:** The aim of our project is to offer support for families and develop empowerment over chronic and rare Rheumatic Pathologies in children; giving easier access to information, promoting actions in order to offer an early diagnosis, helping families face the new challenges they will be inevitably exposed to

Methods

The method is to create a partecipative process by involving all possible factors in an active process regarding chronic and rare rheumatic pathologies in children. We have involved, over the course of the years and through the help of educational and informational workshops, various family members, journalists and paediatricians.

**Results:** We have therefore been able to build a social window, in collaboration with professional staff, that works overall to make the journey that these families will have to face easier. With the use of this social window, the family nucleus can find information as well as a place to be heard and guided.

Moreover, available to them is also a psychotherapist psychologist, both over the phone or in the form of a private meeting. The clinical instrument used here is the meeting with our staff (both hearing and empathy), and with the use of monitoring sheets, drawings and illustrations according to the age of the child. At the end of this formative journey, the family will be asked to fill out a survey in order to rate our project.

Our social window is also available online in the form of a blog. Other communication and participation activities take place in local doctor’s surgeries, schools, and with the collaboration of Rheumatology Paediatricians. We have also developed a comic, ‘A new challenge together’, in order to inform young adults and children on these issues.

**Conclusion:** The presence of a Rheumatic Pathology can result in heavy effect on the life of a child and its family. These impacts vary depending on the resources that they might have available. May these be ‘external’ (financial and economical, available treatments, network support) or ‘internal’ (the possession of medical and bureaucratic information useful to the management of the problem, the relationship between the members of each family and their degree of flexibility).

Our project, ‘A Smiling Childhood’ has kept many families informed, emotionally supported and aware of their internal resources.

**Disclosure of Interest:** None Declared

### P116 Anxiety, depression, and parental perception of uncertainty among parents of children with juvenile idiopathic arthritis in Bogota, Colombia.

#### Adriana Diaz-Maldonado^1,2,3^, Sally Pino^2,4^, Pilar Guarnizo^2,5,6^, Juan Manuel Reyes^2^, Leonardo Ariza^2^

##### ^1^Hospital de la Misericordia, Bogota, Colombia; ^2^Care for Kids, Bogota, Colombia; ^3^Instituto Roosevelt, Bogota, Colombia; ^4^Hospital San Jose Infantil, Bogota, Colombia; ^5^Fundacion Cardio Infantil, Bogota, Colombia; ^6^Cayre, Bogota, Colombia

###### **Correspondence:** Adriana Diaz-Maldonado

**Introduction:** Juvenile idiopathic arthritis (JIA) is a chronic illness which affects among 2 – 150 children for 100.000 in Europe and North America, most of them are cared by their parents (1). It has been estimated that parents of a child with chronic illness can present mood problems, cognitive problems, anxiety, high level of distress and lower levels of quality of life (2).

**Objectives:** Describe the anxiety, depression, familiar functionality and parental perceptions among parents of a child with JIA in Bogotá, Colombia.

**Methods:** A cohort of parents of children with JIA were approached for this study in Bogota, Colombia. The questionnaires Hamilton rating scale for anxiety (HAM-A), Beck depression inventory (BDI), family APGAR scale, parental perception of uncertainty scale (PPUS), and parental coping strategy inventory (PCSI) were self-administrated. Descriptive analysis was performed according to the nature of the variables.

**Results:** Twenty two parents participated in the study characterized by 16 women and 6 men. The average age of the parents was 43.86 years (ranged from 33 to 62 years). Most of them with mild to high income and the 55% of the parents had graduate degrees. Age of children with JIA was 14.95 years (8 to 20 years), 55% were female. The most frequent type of JIA was polyarticular and enthesitis-related arthritis (64%), followed by systemic (16%), oligoarticular (16%) and psoriatic arthritis (5%).

In terms of anxiety, 46% of parents reported having moderate anxiety. Similar results related to stress were observed. Mild depression symptoms were found in 18% of parents and moderate in 9%. Severe and moderate familiar dysfunctionality were reported by the parents in 14% and 36%, respectively. There was not association of familiar dysfunctionality and the number of years living with the disease. However, those parents of children with more than two years of disease present an increase in moderate symptoms of depression.

Perception of uncertainty was moderate with mean score of PPUS of 83.85 (SD 15.66). Parents of children with recent diagnosis(less than 2 years) presented higher scores than parents of children which had been diagnosed longer than two years (mean score 88.5 (SD 8.70) vs 80.75 (SD 18.68), respectively). Among parents with higher PPUS score: 89% reported severe or moderate anxiety, 66% reported moderate symptoms of depression, and 44% informed moderate or severe familiar dysfunctionality.

Being optimistic, learning about the diseases and treatment, and interactions with their ill child were the most frequent coping strategies used by the parents. However, parents of children with recent diagnosis are more optimistic and interact more with their ill child compared with parents of children which had been diagnosed longer than two years.

**Conclusion:** This study shows that anxiety and stress in parents of children with JIA is present frequently. Perception of uncertainty, anxiety, and familiar dysfunctionality were observed more frequent and severe in parents of children with recent diagnosis(less than two years). Considering the limitations of the study, in order to alleviate anxiety and depression symptoms on parents, and prevent family dysfunctionality, psychological intervention and participation in group support are recommended strategies for parents of children with recent JIA diagnosis.

References

1. Ravelli A, Martini A. Juvenile idiopathic arthritis.. Lancet. 2007;369:767–778. doi: 10.1016/S0140-6736(07)60363-8

2.Murphy NA, Christian B, Caplin DA, Young PC: The health of caregivers for children with disabilities: caregiver perspectives. Child Care Health Dev 2007, 33:180 – 187.

**Disclosure of Interest:** None Declared

### P117 Rehabilitation games for juvenile idiopathic arthritis. Focus on hand and wrist.

#### Michaela Foà^1^, Rocco M. Chiuri^2^, Antonella Petaccia^1^, Fabrizia Corona^1^, Pier L. Lanzi^2^, Giovanni Filocamo^1^

##### ^1^Dipartimento della Donna, del Bambino e del Neonato, Fondazione Irccs Cà Granda Ospedale Maggiore Policlinico, Milano, Italy; ^2^Dipartimento di Elettronica, Informazione e Bioingegneria, Politecnico di Milano, Milano, Italy

###### **Correspondence:** Michaela Foà

**Introduction:** Juvenile idiopathic arthritis (JIA) is the most common chronic rheumatic disease in children and an important cause of short-term and long-term disability.

Physiotherapy and occupational therapy, with the aim to keep or restore joint function and to achieve a normal pattern of mobility, are important components of the therapeutic approach. In standard physical therapy patients tend to lose interest in the treatment, due to the monotony of the exercises, and as a consequence, patients either perform their exercises irregularly or quit the physical therapy. In recent years, the introduction of new gaming input devices, such as Nintendo’s Wii Remote™ and Microsoft’s Kinect, and the development of games that involve physical activity, induced the researchers to consider the possibility of using videogames to perform physical therapy.

**Objectives:** To design a set of rehabilitation games that could help patients affected with Juvenile Idiopathic Arthritis performing their physical therapy.

**Methods:** A multidisciplinary study group involving paediatric rheumatologists, physiotherapists and engineers of the Department of Electronics, Information and Bioengineering of the Politecnico of Milan was created.

During the first meeting, the rheumatologists explained the basic aspects of disease and the principal physical problem observed in patients with JIA; the therapists introduced how the physical therapy for JIA works.

In order to improve the final outcome, engineers used an iterative design approach, that is, they designed the games, and then they followed a cyclic process of prototyping, testing and analyzing designs. The key points on which engineers focused were: the ability of the game to adapt to the capabilities of the patient, the possibility for the therapists to create custom game levels, the need to save every information about how the patient performs during the exercises and the possibility to see again the exercise carried out by the patient.

For this study, the attention was focused on hand and wrist.

Four different games to play were developed. For each game was defined a rewarding system that increases the score when the player does something good, but does not decrease it when he/she makes a mistake. The scoring system was also a useful first qualitative feedback for the therapist about the patient’s performance.

The therapists were asked to create a custom level for the flight simulator game, so she could simulate an exercise performed in a typical training session.

**Results:** Three poliarticular JIA patients took part to the first experimental session. Ten, fifteen and twenty-one years old respectively. The first patient had ankle involvement, while the last two had wrist and small joints of the hand involvement.

Two games were tested on the 3 patients; a flight simulator and a Flappy Bird-like game. The flight simulator, in particular, was tested both with the hands and with the feet.

The feedback from the subjects was quite good; The therapists’ feedbacks were also good. The patients enjoyed the exercises much more and “it did not look like they were doing exercises”. The subjects were doing without complaint the same exercises that they found to be boring and difficult during standard physical therapy.

**Conclusion:** The feedbacks received during the experimental sessions validated the work. The patients liked the games and suggested some additional changes to make them more appealing. The therapists also were satisfied with the study design. They were glad to see how easily the patients performed their exercises by playing the games.

In future work it should be possible t dynamically adapt the difficulty during the game in relation to the patients’ performances. It would be consider also the possibility to use a webcam to see live the patients while they are playing and to record the patient’s performance, giving the therapist a second visual feedback in addition to the replay.

**Disclosure of Interest:** None Declared

### P118 Rehabilitation games for juvenile idiopathic arthritis. Focus on knee and ankle.

#### Amalia Lopopolo^1^, Mattia Giannotti^2^, Antonella Petaccia^1^, Fabrizia Corona^1^, Pier L. Lanzi^2^, Giovanni Filocamo^1^

##### ^1^Dipartimento della Donna, del Bambino o del Neonato, Fondazione Irccs Cà Granda Ospedale Maggiore Policlinico, Milano, Italy; ^2^Dipartimento di Elettronica, Informazione e Bioingegneria, Politecnico di Milano, Milano, Italy

###### **Correspondence:** Amalia Lopopolo

**Introduction:** Juvenile idiopathic arthritis (JIA) is the most common chronic rheumatic disease in children and a cause of short-term and long-term disability.

Physiotherapy is an important component of the therapeutic approach. The rehabilitation process is developed through a series of simple, repetitive exercises to be performed frequently and for a long time. Often patients lose interest and stop performing them before the results have been accomplished.

Recently a new frontier in the design of videogames has been explored: the games for rehabilitation. The development of this research field has been limited by the cost, technical limitations and technical expertise needed to use the products and the sensors needed.

The introduction of new gaming input devices and the development of games that involve physical activity, induced the researchers to consider the possibility of using videogames to perform physical therapy.

**Objectives:** To design a set of rehabilitation games for children with JIA, that would be considered amusing by the largest part of players, while helping them in performing their rehabilitation exercises for the mobility of knee and ankle.

**Methods:** The project was conducted in collaboration between pediatric rheumatologists, physiotherapists and engineers researchers of the Politecnico of Milan.

The specific rehabilitation principles were applied to the ones of the common game design, to obtain games both medical relevant and fun to play. The iterative design approach, a methodology based on a cyclic process of prototyping, testing, analysing, and refining of the games was applied by the researchers.

Four different games were designed, one for the ankle, three for the knee. The third and the fourth games were redesigned to perform two types of exercises.

The first exercise prescribe the use of a wobble board, a board mainly used to perform equilibrium exercises. During the exercise the patient had to tilt on all four different directions the board by performing the deflection of the extension of the ankles, if the patient is seated on a chair, or by changing the position of the whole body.

The second exercise prescribed to perform the extension-deflection movement of the knee while the patient was seated from the rest position to the maximum angle reachable by the patient.

For each of this games the therapist could completely personalize the parameters of the game in order to personalize the experience of the patient and make it suitable for his/her clinical condition.

Experimental sessions were performed to validate the result of the system.

The interaction of the patient with the games were tested, and it was verified how they react to the different type of experience.

Results

Tuning session.

Tuning session was completed by testing the games with 2 JIA patients, 15 and 12 years of age respectively, with predominant involvement of ankles (patient 1) and ankles and right knee (patient 2).

Therapeutic sessions.

Two therapeutic sessions were performed on 4 (3 female and 1 male) and 3 (female) patients respectively. Median age 7 years (5-15 years)

Feedbacks received from patients and therapists were good, and all the patients completed easily their sessions.

**Conclusion:** Four rehabilitation games for the mobility of knee and ankle were developed. The feedbacks received from the patients and therapists were very good. The rehabilitative sessions were easily controlled, the patients enjoied the sessions and the therapists appreciated also the possibility to monitor the patient even in remote.

**Disclosure of Interest:** None Declared

### P119 Recurrent arthralgia or functional pain? Evaluation of psychological distress in Italian school students

#### margherita Lo Curto, Maria Cristina Maggio, Fabio Campisi, Giovanni Corsello

##### University Department Pro.Sa.M.I. “G. D’Alessandro”, University of Palermo, Palermo, Italy

###### **Correspondence:** Maria Cristina Maggio

**Introduction:** Requests of rheumatologic visits for lims arthralgia are more frequent in these last years, expecially in adolescents. Functional Pain (without demonstrable organic cause), is often associated with psychological problems.

**Objectives:** to differentiate organic articular pain from functional pain;

to evaluate the real incidence of functional pain in healthy school students;

to investigate the correlation between functional pain and psychological disagreement, in a series of school students.

**Methods:** A questionnaire was given to a group of students of primary school; the following data were collected: a) sex, age; b) functional pain; c) relation with relatives, teachers and schoolfellows; d) failure in school-studies.

**Results:** 809 students, 354 females, 455 males, median age 14 years, participated to the study.

Functional Pain was referred from 537/809 students: 265 Females, 272 males: p = 0,155. Pain episodes showed a different incidence between females and males (p = 0,511).

Pain intensity vs. number of episodes in females showed a statistically significant correlation (p = -0,001). Most frequently abdominal pain was recorded in females and limbs pain was selectively described in males.

Psychological disagreement was referred from 513/809 students: 260 females, 253 males (p = 0,150).

Psychological disagreement was reported with: parents in 15; with siblings in 59; with other relatives in 45; with teachers in 42, with schoolfellows in 356.

The correlation disagreement vs. functional pain in all the students included in the study was statistically significant (p < 0,001).

**Conclusion:** Most students reported psychological disagreement and pain. The most frequent cause of disagreement was schoolfellows’ behaviors. Limbs pain is a frequent record, especially in adolescent males.

Functional pain correlates significantly with psychological disagreements: according to literature, it can lead to severe manifestations. Psychological approach, supported by several studies, demonstrated that it is mandatory to recognize such pain; to talk with the students concerning the cause of their psychological disagreements, to know their problems and to reassure them with the aim of gaining their confidence. A confidence with parents and/or teachers and/or their peers, may be useful for adolescents.

**Disclosure of Interest:** None Declared

### P120 Posture and balance deficit in children with JIA: a pilot study

#### Antonino Patti^1^, Giovanni Corsello^2^, Antonino Bianco^1^, Giuseppe Messina^1^, Angelo Iovane^1^, Antonio Palma^1^, Jessica Brusa^3^, Maria Cristina Maggio^2^

##### ^1^Department of Psychology and Educational Science, University of Palermo, Palermo, Italy; ^2^University Department Pro.Sa.M.I. “G. D’Alessandro”, University of Palermo, Palermo, Italy; ^3^Posturalab Italia, Palermo, Posturalab Italia, Palermo, Palermo, Italy

###### **Correspondence:** Maria Cristina Maggio

**Introduction:** Juvenile Idiopathic Arthritis (JIA) is the more frequent rheumatologic disease in paediatric age with a possible worsening on posture in a vulnerable period of life. This condition can show a negative impact on balance and on activities of daily life.

**Objectives:** The aim of the study is to evaluate the efficacy of regular physical activity on posture and balance deficit in children and adolescents affected by JIA.

**Methods:** We enrolled 56 patients (17 patients affected by JIA: JIA group; and 39 healthy control subjects, of comparable age: CG). Furthermore, JIA group was stratiphied in two subgroups: inactive (AIG-SED) and active (AIG-ACT). All the patients were tested by a computerized analysis of posturography, by a new-generation stabilometric platform (Sensor Medica; Guidonia Montecelio, Roma).

**Results:** The group AIG-SED showed statistically significant differences vs. the group CG on the following indexes: ellipse surface (*p <* 0,05), ball length (*p <* 0,0001) e Y mean (*p <* 0,05). At the opposite, no statistically significant difference was relieved between the AIG-ACT and the CG, with the exception of the ball length (*p <* 0,05), which is reduced respect the CG.

**Conclusion:** This pilot study confirms the relieve of regular physical activity on the motor deficit, on balance and posture secondary to JIA, especially in evolutive age.

**Disclosure of Interest:** None Declared

### P121 Improved transitional process by nurse guided transition program

#### Karina Mördrup, Anna Vermé

##### Pediatric Rheumatology Unit, Karolinska University Hospital, Stockholm, Sweden

###### **Correspondence:** Karina Mördrup

**Introduction:** Children’s experience of moving from children’s hospital department to adult department in hospital care are often related to increased stress and anxiety. The transition period could also be very demanding for the parents. Studies show that preparing the children from early age could prevent most of the stress and anxiety. By starting the transition process several years before the child’s move to adult care we presume the parents gets educated and supported for letting their child take more responsibility for its own illness. At the pediatric reumatology department, Astrid Lindgrens Childrens hospital we are working with a transition program inspired by Janet McDonagh. The program consists three meetings with the rheumatology nurse.

**Objectives:** To prepare and facilitate the child and its parents for the child’s transition to adult care.

**Methods:** The children and their parents are called to three meetings over a period of 5 years. The first meeting are placed when the child is 14 years old. Before the meeting the child and its parents filled in different transition formula. To evaluate the child’s knowledge about its disease and treatment we used the questionnaire MEPS abbreviation for medical issues, exercise, pain and social support.

**Results:** To evaluate if the child and its parents found it valuable to participate in the transition program, we did a pilot study. After the visit to the nurse the child got a evaluation formula. The results showed that 86% of the participants should totally recommend this to other children with rheumatic disease and the remaining 14% recommended it. Neither of the children found it embarrassing or hard to fulfilled the transition formula.

**Conclusion:** Both the children and their parents found the first transition meeting with the nurse very educational and valuable. The meeting with the nurse seems to facilitate the transition process for the child and its parents. By offering a structured transition program to the children and their parents the fear of moving to adult care can be reduced.

**Disclosure of Interest:** None Declared

### P122 Transition in rheumatology- 5 year experience

#### Marija Šenjug Perica^1^, Miroslav Mayer^2^, Mandica Vidović^3^, Lovro Lamot^3,4^, Miroslav Harjaček^3,4^, Lana Tambić Bukovac^1^

##### ^1^Department of Pediatric and Adolescent Rheumatology, Children’s Hospital Srebrnjak, Zagreb, Croatia; ^2^Department of Internal Medicine, Division of Clinical Immunology and Rheumatology, University Hospital Centre Zagreb, Zagreb, Croatia; ^3^Department of Pediatric and Adolescent Rheumatology, Clinical Hospital Centre Sisters of Charity, Zagreb, Croatia; ^4^University of Zagreb, School of Medicine, Zagreb, Croatia

###### **Correspondence:** Marija Šenjug Perica

**Introduction:** Transition for patients with chronic diseases is defined as the purposeful, planned movement of adolescents and young adults from child-centered to adult oriented health care system.^1^ Rheumatic diseases of childhood extend to adulthood as active diseases in 30-70%, which is the reason for requiring rheumatologic care into adulthood.

First coordinated transition of pediatric rheumatologic patients in Croatia was organized in 2012 as developmental type of transition, where patient is gradually prepared for the transition by a pediatrician rheumatologist throughout years preceding transfer. The moment of final transfer to adult care is organized in the pediatric rheumatology clinic, where patients and their parents are meeting adult rheumatologist in known environment (pediatric rheumatology ambulance). At the transfer, patient's history is being presented to the adult rheumatologist and afterwards patient is examined by both of the specialists. At the end of the examination, patient is given next checkup date at adult rheumatologist clinic, where the patient will continue with regular follow up visits.

**Objectives:** Our objective is to verify successfulness of organized transition of pediatric rheumatologic patients to adult care.

**Methods:** We have conducted a retrospective research of patients’ databases at Departments of Pediatric Rheumatology and correlated them with data from adult Clinical Immunology and Rheumatology Department in order to verify number of pediatric patients that have continued with regular follow up in adult care after coordinated transition.

**Results:** During 5 year period (2012.-2017.) 78 patients have been transferred to adult rheumatologist. There were 50 JIA patients, 18 SLE patients, and 10 patients with other rheumatic diseases (8 patients with mixed connective tissue disease, 1 with Wegener's granulomatosis, 1 with fever of unknown origin). Median age at the time of transfer was 19,6 years (14,6-26,6). Patients with SLE were older at the time of transfer than two other groups of patients (SLE 21,29 ± 2,99 vs. JIA 18,83 ± 1,22 vs. other diagnosis 19,42 ± 2,8, p < 0,01). Total of 63 patients (80,8%) have continued with regular follow up at adult rheumatologist and 15 patients never showed up at adult rheumatologist clinic, probably due to remission of the disease or possible follow up by other chosen adult rheumatologist.

**Conclusion:** Young adults with rheumatic diseases have complex medical and psychological needs and transition to adult care is a critical component of care. In order to maintain adequate level of follow up and prevent additional flares in turbulent adolescent period, continuity of rheumatologic care is essential. By organizing coordinated transition to adult rheumatologist, a most of the pediatric patients with rheumatic disease (80,8%) are continuing with regular follow up in the adult care. Each transition should be an individually tailored process according to needs and psychological maturity of patients.

^1^Blum RW et al. Transition from child-centered to adult health-care systems for adolescents with chronic conditions. A position paper of the Society for Adolescent Medicine. J Adolesc Health. 1993.

**Disclosure of Interest:** None Declared

### P123 The psychomotor effect of an organized summer camp program on children with rheumatic diseases: a 5 year evaluation

#### Maria Stavrakidou, Kyriaki Spanidou, Polyxeni Pratsidou-Gertsi, Artemis Koutsonikoli, Florence Kanakoudi-Tsakalidou

##### First Dept of Pediatrics,Pediatric Immunology and Rheumatology Referral Center, Hippokration Hospital, Thessaloniki, Greece ^2^Asklepeio Physiotherapy Clinic, Thessaloniki, Greece

###### **Correspondence:** Maria Stavrakidou

**Introduction:** According to the Referral Center’s policy regarding the patients’ psychomotor rehabilitation, a summer camp program is annually offered to children with Chronic Rheumatic Diseases (CRD) in clinical remission and ability for self-care.They attend a sport camp addressed to healthy children, where the CRD group participates in various camp activities (handicrafts, arts, games, sports and swimming), accompanied by a specialized physiotherapist and a pediatric rheumatologist. During their 2-week camp life, they additionally receive education and practice on maintaining group and personalized physiotherapy.

**Objectives:** To evaluate the psychomotor impact of organized summer rehabilitation program on children with CRD, in a setting away from the caregiver protection, for 5 consecutive years.

**Methods:** A 28-item questionnaire was created pertaining 5 domains: physical activity, self-care, pain, socialization and psychology and a score range 1-4 (4 the best). The questionnaire was annually completed by the children after the end of the camp and the responses were evaluated in respect to the participants’ age and their experience in the camp setting.

**Results:** Replies of 69 campers with a mean age of 11 yrs were analyzed. A mean total score of 54/65 was found, which was positively correlated with the participants age (p < 0.001). Campers with no previous experience had a significantly median lower score (48), after age adjustment (p = 0.05). The domains of physical activity and psychology had the worst scores for the naive campers (p = 0.018, and p = 0.027, respectively). Socialization was positively correlated with age (p = 0.008) and especially among the naïve young participants (p = 0.005). There was no correlation between pain and activities’ restriction. Home sickness regarding either the family, or peers or TV watching was reported by 82%, 74% and 37.5%, respectively. Noteworthy, 88% expressed the desire to become future camp team-leaders.

**Conclusion:** These findings indicate that the aforementioned kind of camp life offering a rehabilitation program for children with CRD has a beneficial impact on their quality of life. The valuable effect was found to be correlated with the campers’ age and was more prominent, in those with the least experience. The reported home sickness by the majority of children did not affect their active participation in the program. Moreover, they expressed the wish to gain more camp experiences and take future camp responsibilities as leaders.

**Disclosure of Interest:** None Declared

## Systemic lupus erythematosus

### P124 Emerging role for the renal glomerular endothelial cells as potent inflammatory contributors in juvenile-onset lupus nephritis

#### Paraskevi Dimou^1^, Matthew Peak^1,2^, Angela Midgley^1^, Simon C. Satchell^3^, Rachael D. Wright^1^, Michael W. Beresford^1,2^

##### ^1^Department of Women's and Children's Health, University of Liverpool, Liverpool, United Kingdom; ^2^Department of Paediatric Rheumatology, Alder Hey Children’s NHS Foundation Trust, Liverpool, United Kingdom; ^3^Academic Renal Unit, University of Bristol, Bristol, United Kingdom

###### **Correspondence:** Paraskevi Dimou

**Introduction:** Lupus nephritis (LN) is one of the most frequent complications of juvenile-onset systemic lupus erythematosus (JSLE). LN can cause serious renal damage due to formation of a highly inflammatory environment inside the kidneys, eventually leading to glomerular destruction, proteinuria and end-stage renal failure. The specific role of the resident renal cells in LN is still largely unknown.

**Objectives:** To investigate the role of the human glomerular endothelial cells (GEnCs) in renal inflammation, by measuring the production of pro-inflammatory factors under various stimuli.

**Methods:** Human conditionally immortalized GEnCs (ciGEnCs) were treated for 24 hours with 10 ng/ml of human recombinant cytokines previously identified to play a prominent role in juvenile-onset LN; Interferon (IFN) -alpha and -gamma (IFN-α, IFN-γ), interleukin (IL) -1β, IL-6 and IL-13, tumour necrosis factor-alpha (TNF-α) and vascular endothelial growth factor (VEGF). The effect of bacterial endotoxin inflammation was tested via lipopolysaccharide (LPS) treatment (1 μg/ml).

Real-time PCR, ELISA and Luminex multiplex assays were performed to assess changes in expression and secretion of pro-inflammatory cytokines and chemokines: TNF-α, IFN-γ, IL-6, IL-8, IL-10, soluble vascular cell adhesion molecule-1(sVCAM-1), monocyte chemoattractant protein-1 (MCP-1), monocyte inducible protein-1α (MIP-1α), IFN-γ induced protein-10 (IP-10), macrophage- and granulocyte macrophage- colony stimulating factors (M-CSF, GM-CSF). Flow cytometry was used to assess surface expression of intercellular adhesion molecule-1 (ICAM-1) and VCAM-1. Data were analysed by Kruskal-Wallis test with Dunn’s post-hoc test. All measurements were compared to untreated ciGEnCs.

**Results:** At 24 hours, TNF-α and IL-1β upregulated MCP-1, MIP-1α and GM-CSF protein secretion. IL-1β stimulated IL-6 and TNF-α secretion whereas IFN-γ promoted IP-10, IL-10 and TNF-α secretion. The combined cytokine treatment enhanced M-CSF secretion. IL-13 upregulated sVCAM-1 secretion whereas IL-13 and TNF-α increased surface expression of VCAM-1. TNF-α upregulated surface ICAM-1. LPS stimulated IL-6, IL-10, MIP-1α, IP-10, GM-CSF, IFN-γ and TNF-α secretion. The outcome of the real-time PCR assays reflected the protein assay results.

ELISA, Luminex and flow cytometry results of protein secretion and surface expression are presented in the table below.

**Conclusion:** This study provides evidence for a prominent role of GEnCs in the propagation of inflammation in LN. Four pro-inflammatory cytokines (TNF-α, IL-1β, IFN-γ and IL-13) and LPS were identified as key players in GEnC activation *in vitro*.

During a renal flare in LN, these cytokines together with LPS could stimulate inflamed GEnCs to produce other pro-inflammatory cytokines, chemokines and adhesion molecules which, in turn, would further enhance the glomerular inflammatory microenvironment by increasing the recruitment of immune cells inside the glomerulus.

**Disclosure of Interest:** None Declared

**Table 35:** **(Abstract P124).** See text for description

ciGEnC treatment	Pro-inflammatory factors	Protein concentration (pg/ml)/surface expression Median/GeoMean Fluoresecence; IQR, n = 6) Untreated Stimulated	
TNF-α	**·** GM-CSF	(0; 0-0)	(197.7; 189.7-272.3, P = 0.049)
	· MIP-1α	(208.5; 207-209)	(222.3; 221-223.3, P = 0.048)
	· ICAM-1	(247.5; 211.5-279)	(443; 353.3-472.3, P = 0.009)
IL-1β	**·** IL-6	(30.55; 16.9-108.5)	(3479; 1815-5243, P = 0.0004)
	· GM-CSF	(0; 0-0)	(950.5; 464.9-1128, P = 0.0004)
	· TNF-α	(4.484; 3.717-4.383)	(10.60; 7.048-12.71, P = 0.011)
	· MIP-1α	(208.5; 207-209)	(221.5; 215.8-225.8, P = 0.049)
IFN-γ	**·** IL-10	(0.514; 0.370-0.610)	(18.19; 15.69-19.77, P = 0.004)
	· TNF-α	(4.484; 3.717-4.383)	(8.381; 7.270-8.825, P = 0.029)
	· IP-10	(2.387; 2.279-3.688)	(3015; 2642-3114, P = 0.008)
IL-13	**·** sVCAM-1	(191.8; 88.54-559.1)	(1539; 1033-1646, P = 0.022)
Combined cytokine treatment	**·** MCP-1	(800.1; 446-1705)	(3755; 3359-3911, P = 0.008)
	· M-CSF	(192.4; 85.19-1427)	(3461; 2093-5098, P = 0.021)
	· VCAM-1	(59.60; 49.65-69.58)	(119; 85.13-163.3, P = 0.005)
LPS	**·** IL-6	(30.55; 16.9-108.5)	(3323; 2083-4886, P = 0.0007)
	· IL-10	(0.514; 0.370-0.610)	(17.32; 9.014-20.25, P = 0.009)
	· GM-CSF	(0; 0-0)	(312.2; 125.5-576.5, P = 0.017)
	· IFN-γ	(0; 0-0)	(28.47; 12.14-40.44, P = 0.003)
	· TNF-α	(4.484; 3.717-4.383)	(14.60; 10.38-20.15, P = 0.0005)
	· IP-10	(2.387; 2.279-3.688)	(2870; 1042-3147, P = 0.013)

### P125 Comparison of clinical and serological features of juvenile and adult-onset neuropsychiatric lupus in Spanish patients

#### Sandra Garrote Corral, Antía García Fernández, Walter A. Sifuentes Giraldo, Alina L. Boteanu, María L. Gámir Gámir, Antonio Zea Mendoza

##### Rheumatology, Ramon Y Cajal University Hospital, Madrid, Spain

###### **Correspondence:** Sandra Garrote Corral

**Introduction:** Neuropsychiatric (NP) manifestations are a main cause of morbidity and mortality in juvenile-onset systemic lupus erythematosus (jSLE). Some studies suggest that they are more frequent and severe in jSLE than in adult-onset SLE (aSLE).

**Objectives:** To compare the clinical and serological profile of pediatric and adult patients with neuropsychiatric lupus (NPSLE) treated in a Spanish tertiary center.

**Methods:** A retrospective study of patients with jSLE (age of onset: 0-18y) and aSLE (age of onset: >18y) seen in our center during the period 1988-2016 was performed. Case definitions of the American College of Rheumatology were used to identify NPSLE manifestations. Demographics, clinical and serological data were obtained through a review of their medical records.

**Results:** A total of 69 patients with NPSLE were included, aSLE 41 (59%) and jSLE 28 (41%), the comparison of groups is presented in the table. Most of them were Caucasian (92%), mean age at diagnosis in adults was 36.4 years (range: 19-68) and 13.9 years (range: 8-18) in children. The proportion of males was higher in the latter group. The mean duration of the disease was significantly greater in adults, as well as the time from SLE diagnosis to NP manifestation onset, although without significant difference. Central NP manifestations were the most frequent in both groups (aSLE 93%, jSLE 96%) regarding to the peripheral manifestations (aSLE 12%, jSLE 11%). The most frequent manifestations in aSLE were headache (29%), cerebrovascular disease (27%), seizures (17%) and myelopathy (15%), whereas in jSLE were seizures (46%), headache (29%), mood disorder/depression (25%), psychosis (18%) and autonomic disorders (18%). A significant group of patients presented ≥ 2 central manifestation during their evolution (aSLE 32%, jSLE 41%), with the mean number of manifestations in adults being 1.36 (range: 1-3) and in children 1.44 (range: 1-4). Patients with jSLE developed lupus nephritis with a significantly higher frequency, as well as higher titres of anti-DNA antibodies, erythrocyte sedimentation rate (ESR) and hypocomplementemia. During the study period there was mortality in 2 cases of aSLE and 2 jSLE (5% and 7%, respectively).

**Conclusion:** Our results corroborate that juvenile patients with NPSLE present higher disease activity compared to adults. There was no significant diference in the time from SLE diagnosis to NP manifestation onset, but tended to be shorter in jSLE. The spectrum of NPSLE was varied both groups and an important proportion of them developed ≥ 2 manifestation. Mortality continues to be important in NPSLE in both age groups.

**Disclosure of Interest:** None Declared

**Table 36:** **(Abstract P125).** See text for description

	Juvenile NPSLE	Adult NPSLE	p-value
N° of patients	28 (41%)	41 (59%)	-
Women:men	20:8	39:2	0.0060*
Time of disease (months)	19.8	232.5	0.0001*
NP manifestations at onset	7 (25%)	11 (27%)	0.8651
Lupus nephritis	16 (57%)	9 (22%)	0.0028*
Anti-DNA ab (IU/ml)	178.9	39.4	0.0005*
ESR (mm/h)	53.8	35.7	0.0199*
C3 low (< 80 mg/dl)	22 (79%)	16 (39%)	0.0012*
C4 low (< 16 mg/dl)	22 (79%)	13 (32%)	0.0001*

### P126 Cytokine profile and expression of STAT1 and STAT5 in peripheral blood in patients with childhood-onset systemic lupus erythematosus

#### Marija Holcar^1^, Aleš Goropevšek^2^, Tadej Avčin^3^

##### ^1^Unit for Special Laboratory Diagnostics, University Children's Hospital, University Medical Centre Ljubljana, Ljubljana, Slovenia ; ^2^Department for Laboratory Diagnostics, University Medical Centre Maribor, Maribor, Slovenia ; ^3^Department of Allergology, Rheumatology and Clinical Immunology, University Children's Hospital, University Medical Centre Ljubljana, Ljubljana, Slovenia

###### **Correspondence:** Marija Holcar

**Introduction:** Systemic lupus erythematosus (SLE) is a multisystemic chronic autoimmune disease with variable clinical and laboratory manifestations. Characteristic hyperactivity of the immune system in patients with SLE is reflected in dysregulation of proteins of various signaling pathways as well as in aberrant concentrations of pro- and anti-inflammatory plasma cytokines.

**Objectives:** In this study, we focused on STAT1 and STAT5 basal expression and phosphorylation as well as cytokine profile in peripheral blood of patients with cSLE.

**Methods:** Whole peripheral blood of 20 healthy donors (HD, mean age 17.2 years), 10 JIA patients (disease control, mean age 11.1 years), and 17 patients with cSLE (mean age 18.0 years) was analysed using the Phosflow flow cytometry. Cells were analyzed using FACSCantoII Flow Cytometer (BD Biosciences) and subsequent analysis using FlowJo software (Tree Star). We measured concentrations of IL-1α, IL-1β, IL-2, IL-4, IL-6, IL-8, IL-10, MCP1, VEGF, TNF-α and IFN-γ in the peripheral blood of 10 healthy donors (mean age 18.9 years), 5 JIA patients (mean age 15.0 years) and 10 patients with cSLE (mean age 18.2 years) using the multiplex immunoassay on a biochip. Two-tailed Kruskal-Wallis test was used to evaluate differences between the groups.

**Results:** Our results demonstrate a significant increase in basal expression of protein STAT1 (cSLE:JIA p < 0.05; cSLE: HD p < 0,0001) and protein STAT5 (cSLE:HD p < 0,001) in Th lymphocytes of cSLE patients compared to control groups. Basal phosphorylation of STAT1 was also elevated (cSLE:JIA p < 0.05; cSLE: HD p < 0,001), but we found no difference in basal phosphorylation of protein STAT5 between groups.This indicates strong IFN signature and suggests a mechanism of inflammation self-maintenance utilizing the JAK-STAT signaling pathway in cSLE patients. No significant differences were found in the peripheral blood concentrations of IL-1α, IL-1β, IL-2, IL-4, IL-6, IL-8, IL-10, MCP1, VEGF, TNF-α and IFN-γ between groups, which suggests a limited role of these cytokines in the pathogenesis of cSLE.

**Conclusion:** Many IFN regulated genes are dependent o STAT1 for optimal transcription, and STAT1 protein expression is under control of IFNs. Our findings support the role of aberrant expression and phosphorylation of STAT proteins, especially STAT1, but show normal physiological concentrations of many cytokines, including IFN-γ. This finding suggests that other cytokines, such as IFN-α and IL-17, may contribute to the pathogenesis of the disease.

**Disclosure of Interest:** None Declared

### P127 The expression of IFN receptor chains on naïve and primed neutrophils

#### Sophie Irwin^1^, Angela Midgley^1^, Matthew Peak^2^, Michael Beresford^1,2^

##### ^1^Women's and Children's Health, University of Liverpool, Liverpool, United Kingdom; ^2^NIHR, Alder Hey Clinical Research Facility, Alder Hey Children’s NHS Foundation Trust, Liverpool, United Kingdom

###### **Correspondence:** Sophie Irwin

**Introduction:** Juvenile-onset Systemic Lupus Erythematosus (JSLE) is a multi-organ autoimmune disease, characterised by autoantibodies directed against nuclear antigens caused in part by increased dysregulated apoptotic neutrophils. Type 1 (α and β) and 2 (γ) interferon (IFN) expression is increased in both adult-onset and JSLE. An associated granulocyte and IFN gene signature present in JSLE. IFNs have both anti- and pro-apoptotic effects, through the activation of anti-apoptotic STAT3 and pro-apoptotic STAT1 respectively. Previous studies demonstrated high concentrations of IFN induced apoptosis in primed neutrophils. Physiological levels of IFNs induced an increase in pSTAT1 and a decrease in pSTAT3 in primed neutrophils compared to naïve neutrophils. IFN signalling is dependent on IFN receptor chains, which may contribute to apoptosis. However, expression of IFN receptors that may contribute to this differential signalling is yet to be established.

**Objectives:** To investigate the effect of neutrophil activation on the expression of the type 1 and 2 IFN receptors.

**Methods:** Neutrophils were isolated from healthy adult control donor blood, and stimulated with 1 μg/mL each of: TNFα, IFNα, IFNγ, and IFNβ for 30 mins. Type 1 IFN receptor (IFNAR) and type 2 IFN receptor (IFNGR) were analysed using flow cytometry. Neutrophils were stained with IFNAR1-PE, IFNAR2-APC, IFNGR1-PE and IFNGR2-APC, and analysed on the flow cytometer. Neutrophil activation markers CD11b and CD62L were also assessed to confirm TNFα-associated priming.

**Results:** Activation of neutrophils following TNFα treatment was confirmed by: (1) the significant increase in CD11b (geometric mean ± SEM; 2135 ± 289 [IFNAR analysed cells], n = 5, p < 0.05; 2227 ± 310 [IFNGR analysed cells], n = 5, p < 0.05) compared to unstimulated cells (1259 ± 152 [IFNAR analysed cells]; 1242 ± 107 [IFNGR analysed cells]) and (2) the decrease in CD62L (90 ± 15 [IFNAR analysed cells], n = 5, p < 0.05; 68 ± 6 [IFNGR analysed cells] n = 5, p < 0.05) compared to unstimulated cells (377 ± 53 [IFNAR analysed cells]; 375 ± 51 [IFNGR analysed cells]). IFNα and IFNγ significantly increased CD11b (IFNα = 1334 ± 139 vs 1259 ± 152, n = 5, p < 0.05; IFNγ = 1325 ± 120 vs 1242 ± 107, n = 5, p < 0.05). There was no overall observed change in activation markers upon IFNβ stimulation. IFNAR1 was significantly decreased (53 ± 11 vs 123 ± 36, n = 5, p < 0.05) and IFNGR2 was significantly increased (58 ± 7 vs 47 ± 3, n = 5, p < 0.05) in TNFα activated neutrophils compared to unstimulated neutrophils. IFNγ induced a small increase in IFNGR2 (59 ± 10, n = 5, p = 0.345) compared to unstimulated neutrophils (47 ± 3), although this was not statistically significant. Overall, IFNs had little effect on receptor expression.

**Conclusion:** Here we show that in an *in vitro* model, neutrophils from healthy adults can be activated using TNFα, determined by the expression of established activation markers. IFNα and IFNγ have some activating effect on neutrophils; however IFNβ was shown to have no overall activating effect. There was a significant increase in the IFNAR1 expression and a significant decrease in IFNGR2 expression in activated neutrophils compared to unstimulated neutrophils.

Previous studies have shown an IFN induced increase in apoptosis in primed neutrophils, which may be due to the increase in pSTAT1 and decrease in pSTAT3 also seen in primed neutrophils. This increase in pSTAT1 and subsequent increase in apoptosis may be due to the differential expression of the receptors in IFN signalling. These observations may be important factors in the increase in JSLE neutrophil apoptosis, and create a potential therapeutic target.

**Disclosure of Interest:** None Declared

### P128 C1Q-deficiency lupus treated successfully with fresh frozen plasma in a sibling pair: first description of this novel therapy in paediatric patients

#### Rebecca A. James^1^, Yvonne Glackin^1^, Clarissa A. Pilkington^1,2^

##### ^1^Department of Rheumatology, Great Ormond Street Hospital, London, United Kingdom ^2^UCL Institute of Child Health, University College London, London, United Kingdom

###### **Correspondence:** Rebecca A. James

**Introduction:** We present the case of a paediatric male and female sibling pair with severe monogenic lupus due to C1q deficiency, refractory to prior treatments, who experienced substantial clinical and laboratory improvement following treatment with fresh frozen plasma (FFP). FFP has previously been described in the treatment of adult and adolescent patients with C1q deficiency, with good results. To our knowledge this is the first description of its use in paediatric patients.

**Objectives:** To describe fresh frozen plasma as a novel therapy for the treatment of children with lupus due to C1q-deficiency, and to describe the clinical and laboratory improvement experienced by a sibling pair who received this treatment.

**Methods:** A seven year old girl and her three year old brother were well known to our paediatric rheumatology service with severe lupus due to C1q deficiency. The pair were born of consanguineous parents of Pakistani background. The female patient is known to be homozygous Q208X in the C1QA gene; both children had unrecordable C1q levels and severely reduced classical complement pathway functioning (0-1% normal levels).

The female patient presented at 7 months with fever, rash and myositis. She has had persistent disease activity since, and has failed to respond adequately to multiple prior therapies including ciclosporin, methotrexate, azathioprine, hydroxychloroquine, mycophenolate and rituximab. Her disease was characterised by ongoing severe facial rashes, scarring alopecia and cytopenias. She was also under neurology follow-up for spastic dystonic motor disorder in her lower limbs which had been extensively investigated with no alternate cause found. Her disease was complicated by an episode of meningococcal sepsis with acute renal failure aged 13 months.

Her brother was diagnosed with C1q deficiency on sibling screening at three months of age, and developed malar rashes aged 10 months. His phenotype is similar to that of his sister but somewhat milder, with photosensitive rashes, scarring alopecia and cytopenias, as well as increased lower limb tone. He had previously failed to respond adequately to azathioprine, methotrexate, rituximab and mycophenolate.

Both patients were steroid dependent and showed signs of profound steroid toxicity including cushingoid habitus, growth restriction, hirsuitism and hypertension.

In light of ongoing steroid dependence, and limited treatment options, the pair were commenced on regular infusions of intravenous fresh frozen plasma. Dosing regimen was: 10 ml/kg given weekly for 4 infusions, and fortnightly thereafter. Complement function and C1q levels were monitored.

**Results:** Both siblings experienced a significant improvement in their skin disease and alopecia, with improved rashes, return of hair growth and a reduction in their steroid requirements. After prolonged periods of steroid dependence, the male sibling came off steroid therapy 3 months after commencing FFP infusions; his sister came off steroid therapy 6 months after commencing infusions, by which point she was seven years old and had been on steroids since 8 months of age. Classical pathway complement function testing normalised in both patients, although measured C1q levels remain unrecordably low. To date, neither patient has experienced adverse effects from the FFP infusions.

**Conclusion:** To our knowledge, this is the first description of the use of FFP for the treatment of paediatric patients with C1q deficiency, and proposes FFP as a potentially safe and effective treatment for this rare condition. Further studies are warranted to explore its safety and efficacy profile, and its role as an adjuvant treatment option in C1q-deficiency lupus. (Consent for publication was obtained from the children’s mother.)

**Disclosure of Interest:** None Declared

### P129 Childhood lupus glomerulonephritis outcome is associated with low C3 levels and anti-DNA antibodies at disease onset

#### Claudia S. Magalhaes^1^, Daniele F. Miguel^1^, Luciana G. Portasio^1^, Jose E. Corrente^1^, Glaucia F. Novak^2^, Beatriz Molinari^2^, Ana P. Sakamoto^3^, Rosa R. Pereira^4^, Teresa Terreri^3^, Eloisa Bonfa^4^, Lucia A. Campos^2^, Simone Appenzeller^5^, Claudio A. Len^3^, Clovis A. Silva^2^

##### ^1^Sao Paulo State University (UNESP), Botucatu, Brazil ^2^University of Sao Paulo (ICr-USP), Sao Paulo, Brazil ^3^Sao Paulo Federal University (UNIFESP), Sao Paulo, Brazil ^4^University of Sao Paulo (USP), Sao Paulo, Brazil ^5^Campinas State University (UNICAMP), Campinas, Brazil

###### **Correspondence:** Claudia S. Magalhaes

**Introduction:** Complement activation can damage the kidneys by attracting leukocytes, which in turn causes inflammation, contributing to the pathogenesis of lupus glomerulonephritis (LN). Immunodeficiency of early classical pathway of Complement components can also induce tissue damage with low complement levels. Anti-DNA antibodies and Complement C3, C4, CH50, reflecting complement activation, are currently used parameters to assess SLE disease activity, in daily practice. Nevertheless, their utility remains controversial as C3 and C4 may not accurately reflect complement activation, due to increased levels during acute phase reaction.

**Objectives:** Evaluate serum levels of C3, C4, CH50 and anti-DNA antibodies at disease onset and its association with lupus glomerulonephritis (LN) occurrence and outcome, related to the multifactorial pathogenesis of complement activation in LN.

**Methods:** We combined a cumulative historical database of childhood- Systemic Lupus Erythematosus (c-SLE) (Gomes RC et al. Arthritis Care Res 2016 10.1002/acr.22881) to assess clinical and laboratory features and their relationship with onset age and kidney involvement. It is a retrospective multicenter cohort started in 2013 enrolling up to 2016, 846 pediatric subjects diagnosed with c-SLE by ACR-1997 criteria, in 10 Pediatric Rheumatology centers. Parameters of c-SLE activity were SLEDAI-2 K scores at onset and the last follow up assessment, major organ involvement and disease damage scored by SLICC-DI, during the last visit. Laboratory data, including antibody tests, renal function and Complement tests, were obtained using standard methods in clinical laboratory according to each center standard practice. Renal biopsy was performed and classified according to WHO and ISN/RPS in each of the centers. Subjects were classified in 3 age groups, <6, 6-12 and >12 years of onset age, and according to the presence or absence of LN, during the disease course, estimated by clinical and biopsy findings, during the first and last assessments.Data analyzes was performed by descriptive and parametric statistics.

**Results:** Of the 846 subjects enrolled, mean age 11.6 (SD 3.6) years with 5 (SD 3.6) years of disease duration; 427 (50.5%) had LN, of those 228 were diagnosed with renal biopsy in addition to renal function parameters, and 419 (49,5%) did not have LN. Median SLEDAI-2 K scores at the first and last visits were 15 and 2, respectively. SLICC-DI scores varied from 0-9, median 0. There was no significant difference (NS) of LN proportion, in the three age groups, <6, 6-12, >12 years onset age. Low C3, but not C3 levels, associated significantly with LN at any time of disease course (chi-square test, p = 0.03). Low C4, C4 levels, low CH50 and CH50 levels had no significant association (NS). In the same line, onset hematuria (p < 0.001), proteinuria (p < 0.001) urine casts (p = 0.02), but not urine leukocytes (NS), arterial hypertension (p = 0.0005), neuropsychiatric events at any time (p = 0.02), high ESR (p = 0.008) but not CRP levels, had significant association with LN outcome. High anti-DNA antibody test associated significantly with LN (p < 0.00001), but anti-Sm, anti-RNP, anti-Ro, anti-La antibodies had no significant association (NS). LN at any time associated significantly with higher SLICC-DI scores (p = 0.003).

**Conclusion:** We confirmed in this population of c-SLE with a wide range of manifestations and organ involvement, the role of C3 and anti-DNA antibodies association with the occurrence and outcome of LN. In the same extent, the urine parameters and ESR at disease onset may be reliable and cost-effective tests for identifying early childhood LN flare or remission, and optimize clinical management and treatment in daily practice.

Acknowledgement: Ms. Daniele F. Miguel^1§^ is undergraduate FAPESP scholar (2016/09092-3)

**Disclosure of Interest:** None Declared

### P130 Decreased antibodies against rubella in previously vaccinated treatment naïve-JSLE patients: a prospective case control study

#### Despoina Maritsi^1^, Olga Vougiouka^1^, Margarita Onoufriou^2^, Susan Coffin^3^, Maria Tsolia^1^

##### ^1^Second Department of Paediatrics, Medical Faculty, University of Athens, Athens, Greece, ^2^Pediatrics, "Archbishop MAkarios III" Children' Hospital, Nicosia, Cyprus, ^3^Infectious Diseases Department, Children's Hospital of Philadelphia, Philadelphia, PA, United States

###### **Correspondence:** Despoina Maritsi

**Introduction:** Systemic lupus erythematosus(SLE) is a multisystem autoimmune disease primarily affecting young females. SLE patients are susceptible to infections due to their defective immune system and the immunosuppressive treatment they receive. However we lack data regarding response to specific vaccines. Rubella infection in pregnant women is associated with serious neonatal consequences, including miscarriage, fetal death and congenital rubella syndrome. Thus it is critical that we understand the impact of SLE on young women’s immunity to rubella.

**Objectives:** In this study we determined the immune status against rubella in previously vaccinated juvenile SLE (jSLE) patients, prior to commencement of treatment and at one and three years, and compare this to healthy controls.

**Methods:** This was a prospective controlled study including 21 newly diagnosed jSLE patients and 76 healthy controls. The control group consisted of randomly selected healthy adolescents matched for ethnic origin, age and gender to the jSLE group, attending the Pediatric Outpatients Department for routine checks; the same exclusion criteria were applied. All participants had two doses of the live attenuated MMR vaccine in early childhood. Exclusion criteria were underlying immunodeficiency, recent blood transfusion (<6 months) and previous treatment with immunomodulating agents. Demographic, clinical and laboratory data as well as data regarding immunization status, vaccine history and mean time between the doses of the vaccine were collected. Seroprotection rates and rubella-IgG titers were measured at enrollment and at specific intervals afterwards(0,12,36 months). Total IgG levels were measured simultaneously. Rubella-IgG antibodies were assessed by ELISA. The cutoff value for seroprotection was deemed at 200mIU/ml. The Hospital’s Research and Ethics’ Committee approved the study; written informed consent was obtained. Statistical significance was set at p < 0.05 and analyses were conducted using SPSS.

**Results:** The two groups had similar demographic characteristics, vaccination history and immunization status. No significant differences were detected in terms of vaccine type, time interval between the two groups as well as mean time from last vaccination to blood sampling. Seroprotection rates were adequate for both groups. Nonetheless, the jSLE group had consistently inferior (but not statistically meaningful) seroprotection rates at all time-points. Mean rubella-IgG antibodies were significantly lower in the jSLE compared to the control group(p < 0.01). Similar results were found at one and three years’ follow up(Table 37). None of the participants had hypogammaglobulinaemia at the time of blood sampling. During the follow up period, the jSLE group had greater decrease in antibody levels as indicated from the significant interaction effect of analysis. There was no association detected between the degree of antibody loss and type of jSLE treatment received or jSLE disease activity.

**Table 37:** **(Abstract P130).** Demographic characteristics, seroprotection rates and mean rubella-IgG titers for the SLE and the control group

Parameters	SLE group	Control group	P value
Study sample, n	21	76	0.9*
Age, years, mean (SD)	13.3 (2.3)	13 (2.7)	0.8*
Gender n (%)			
female	20 (95%)	72 (95%)	0.83+
male	1 (5%)	4 (5%)	
Steroids (21/21)			-
- · mean dose	10 mg	NA	
- · mean duration of treatment	17 months		
HCQ (21/21)			
- · mean dose	200 mg		
- · mean duration of treatment	36 months		
Azathioprine (9/21)			
- · mean dose	75 mg		
- · mean duration of treatment	18 months		
SLEDAI score			
- · Enrolment	7		
- · 1 year	1		
- · 3 years	0		
Seroprotection rate at diagnosis (%)	95	98	0.4+
Seroprotection rate at 1 year (%)	92	97	0.1+
Seroprotection rate at 3 years (%)	89	96	0.04+
Mean IgG titers at diagnosis, mIU/ml	432	590	< 0.01*
Mean IgG titers at 1 year, mIU/ml	340	554	< 0.01*
Mean IgG titers at 3 years, mIU/ml	298	468	< 0.01*

**Conclusion:** Although seroprotection rates were similar between the two groups, mean rubella-IgG titers were significantly lower in the jSLE group at all time-points. Further studies are required to address the question of long-term immunity conveyed by immunizations given at an early stage in children with rheumatic diseases. However, evaluation of immunization status against all vaccine preventable diseases in such patients may be beneficiary.

**Disclosure of Interest:** None Declared

### P131 Features of 1,555 childhood-onset lupus in three groups based on distinct time intervals to disease diagnosis: a Brazilian multicenter study

#### Glaucia V. Novak^1^, Beatriz C. Molinari^1^, Ana P. Sakamoto^2^, Maria T. Terreri^2^, Rosa M. Pereira^3^, Claudia Saad-Magalhães^4^, Nadia E. Aikawa^3^, Lucia M. Campos^1^, Claudio A. Len^2^, Simone Appenzeller^5^, Virgínia P. Ferriani^6^, Marco F. Silva^7^, Sheila K. Oliveira^8^, Aline G. Islabão^9^, Flávio R. Sztajnbok^10^, Luciana B. Paim^11^, Cássia M. Barbosa^12^, Maria C. Santos^13^, Blanca E. Bica^14^, Evaldo G. Sena^15^, Ana J. Moraes^16^, Ana M. Rolim^17^, Paulo F. Spelling^18^, Iloite M. Scheibel^19^, André S. Cavalcanti^20^, Erica N. Matos^21^, Teresa C. Robazzi^22^, Luciano J. Guimarães^23^, Flávia P. Santos^24^, Cynthia T. Silva^25^, Eloisa Bonfá^3^, Clovis A. Silva^1^

##### ^1^Pediatric Rheumatology Division, CHILDREN’S INSTITUTE, HOSPITAL DAS CLINICAS HCFMUSP, FACULDADE DE MEDICINA, UNIVERSIDADE DE SAO PAULO, São Paulo, Brazil ^2^Pediatric Rheumatology Division, Federal University of São Paulo (UNIFESP), São Paulo, Brazil ^3^Rheumatology Division, HOSPITAL DAS CLINICAS HCFMUSP, FACULDADE DE MEDICINA, UNIVERSIDADE DE SAO PAULO, São Paulo, Brazil ^4^Pediatric Rheumatology Division, Sao Paulo State University (UNESP), Botucatu, Brazil ^5^Pediatric Rheumatology Division, State University of Campinas (UNICAMP), Campinas, Brazil ^6^Pediatric Rheumatology Division, University of São Paulo (FMUSP-Ribeirão Preto), Ribeirão Preto, Brazil ^7^Pediatric Rheumatology Division, Hospital Geral de Fortaleza, Fortaleza, Brazil ^8^Pediatric Rheumatology Division, Rio de Janeiro Federal University (IPPMG-UFRJ), Rio de Janeiro, Brazil ^9^Pediatric Rheumatology Division, Hospital Jose Alencar, Brasilia, Brazil ^10^Pediatric Rheumatology Division, Pedro Ernesto University Hospital, Rio de Janeiro, Brazil ^11^Pediatric Rheumatology Division, Albert Sabin Hospital, Fortaleza, Brazil ^12^Pediatric Rheumatology Division, Hospital Darcy Vargas, São Paulo, Brazil ^13^Pediatric Rheumatology Division, Santa Casa de São Paulo, São Paulo, Brazil ^14^Rheumatology Division, Federal University of Rio de Janeiro, Rio de Janeiro, Brazil ^15^Pediatric Rheumatology Division, Lauro Vanderley University Hospital, João Pessoa, Brazil ^16^Pediatric Rheumatology Division, Federal University of Pará, Pará, Brazil ^17^Pediatric Rheumatology Division, Obras Sociais Irmã Dulce, Salvador, Brazil ^18^Pediatric Rheumatology Division, Hospital Evangélico de Curitiba, Curitiba, Brazil ^19^Pediatric Rheumatology Division, Hospital Conceição, Porto Alegre, Brazil ^20^Pediatric Rheumatology Division, Federal University of Pernambuco, Recife, Brazil ^21^Pediatric Rheumatology Division, Federal University of Mato Grosso do Sul, Campo Grande, Brazil ^22^Pediatric Rheumatology Division, Federal University of Bahia, Salvador, Brazil ^23^Pediatric Rheumatology Division, University of Brasilia, Brasilia, Brazil ^24^Pediatric Rheumatology Division, Federal University of Minas Gerais, Belo Horizonte, Brazil ^25^Pediatric Rheumatology Division, Hospital Municipal Piedade, Rio de Janeiro, Brazil

###### **Correspondence:** Glaucia V. Novak

**Introduction:** Longer or shorter interval between the first manifestation attributable to SLE and the clinical diagnosis may influence disease expression in terms of initial clinical and laboratorial presentation and disease activity in childhood systemic lupus erythematosus (cSLE) patients.

**Objectives:** The objective of the present large multicenter study was to compare demographic data, Systemic Lupus International Collaborating Clinics Classification Criteria for Systemic Lupus Erythematosus (SLICC) parameters, neuropsychiatric involvement and SLEDAI-2 K at diagnosis in three different groups with distinct time intervals between onset of signs/symptoms and disease diagnosis.

**Methods:** A retrospective multicenter study was performed in 1,555 cSLE (ACR criteria) patients from 27 Pediatric Rheumatology services of 5 regions of Brazil: North (n = 34), Northeast (n = 259), Central-West (n = 124), Southeast (n = 1075) and South (n = 63). Patients were divided in three cSLE groups: A: short time interval to diagnosis (<1 month), B: intermediate time interval (≥1 and <3 months) and C: long time interval (≥3 months). An investigator meeting was held to define the protocol and to harmonize clinical parameters definition in Brasilia, at the time of Brazilian Congress of Rheumatology in 2016. Demographic data, SLICC, neuropsychiatric involvement according to ACR classification criteria and disease activity (SLEDAI-2 K) were systematically evaluated.

**Results:** The number of patients in each group was: A = 60 (4%), B = 522 (33.5%) and C = 973 (62.5%). The median age at diagnosis [11.1 (4.2-17) vs. 12 (1.9-17.7) vs. 12.5 (3-18) years, p = 0.025] was significantly lower in the group A compared to B and C. The median number of diagnostic criteria according to SLICC [7 (4-12) vs. 6 (4-13) vs. 6 (4-12), p < 0.0001] and SLEDAI-2 K score [18 (6-57) vs. 16 (2-63) vs. 13 (1-49), p < 0.0001] were significantly higher in the group A than the other two groups. cSLE groups were distinct with regard to: SLEDAI-2 K ≥ 8 (90% vs. 89% vs. 82%, p = 0.002), serositis (37% vs. 33% vs. 25%, p = 0.002), renal disorder (52% vs. 47% vs. 40%, p = 0.009), neuropsychiatric disorder (22% vs. 13% vs. 10%, p = 0.008), leucopenia/lymphopenia (65% vs. 46% vs. 40%, p < 0.0001) and thrombocytopenia (32% vs. 18% vs. 19%, p = 0.037); as well as synovitis (61% vs. 66% vs. 71%, p = 0.032) and headache (5% vs. 7% vs. 11%, p = 0.043).

**Conclusion:** Our large Brazilian multicenter study identified distinct features of cSLE patients suggesting that a shorter time interval to diagnosis was associated with a more active disease and multisystem severe presentation.

**Disclosure of Interest**: G. Novak: None Declared, B. Molinari: None Declared, A. Sakamoto : None Declared, M. Terreri : None Declared, R. Pereira : None Declared, C. Saad-Magalhães : None Declared, N. Aikawa : None Declared, L. Campos : None Declared, C. Len : None Declared, S. Appenzeller : None Declared, V. Ferriani : None Declared, M. Silva : None Declared, S. Oliveira : None Declared, A. Islabão : None Declared, F. Sztajnbok : None Declared, L. Paim : None Declared, C. Barbosa : None Declared, M. Santos : None Declared, B. Bica : None Declared, E. Sena : None Declared, A. Moraes : None Declared, A. Rolim: None Declared, P. Spelling : None Declared, I. Scheibel : None Declared, A. Cavalcanti : None Declared, E. Matos : None Declared, T. Robazzi : None Declared, L. Guimarães : None Declared, F. Santos : None Declared, C. Silva : None Declared, E. Bonfá : None Declared, C. Silva Grant/Research Support from: Conselho Nacional de Desenvolvimento Científico e Tecnológico (CNPq 302724/2011-7)

### P132 Adult outcomes in a large cohort of childhood-onset SLE patients: coping and resilience in relation to health-related quality of life - the CHILL-NL study

#### Radhevi A. S. Ramnath^1^, Noortje Groot^1, 2^, Ayse Kaynak^1^, Marc Bijl^3^, Radboud J.E. M. Dolhain^4^, Y.K. O. Teng^5^, Els Zirkzee^6^, Karina de Leeuw^7^, Ruth Fritsch-Stork^8^, Irene Bultink^9^, Sylvia S. M. Kamphuis^1^ on behalf of the CHILL-NL study group

##### ^1^Department of Pediatric Rheumatology, Sophia Children's Hospital - Erasmus University Medical Center, Rotterdam, Netherlands ^2^Department of Pediatric Immunology, Wilhelmina Children's Hospital - University Medical Center Utrecht, Utrecht, Netherlands ^3^Department of Internal Medicine and Rheumatology, Martini Hospital, Groningen, Netherlands ^4^Department of Rheumatology, Erasmus University Medical Center, Rotterdam, Netherlands ^5^Department of Rheumatology, Leiden University Medical Center, Leiden, Netherlands ^6^Department of Rheumatology, Maasstad Hospital, Rotterdam, Netherlands ^7^Department of Rheumatology and Clinical Immunology, University Medical Center, Groningen, Netherlands ^8^Department of Rheumatology and Clinical Immunology, University Medical Center Utrecht, Utrecht, Netherlands ^9^Amsterdam Rheumatology and Immunology Center, Location VUmc, Amsterdam, Netherlands

###### **Correspondence:** Radhevi A. S. Ramnath

**Introduction:** Childhood-onset SLE is a lifelong severe disease and results in significant disease/therapy-related damage. This chronic disease has a clear impact on HRQoL, requesting adequate resilience and coping mechanisms. Not much is known regarding coping/resilience capacities in patients with cSLE.

**Objectives:** To investigate individual resilience capacities and coping strategies in a large cohort (n = 106) of adults with childhood-onset SLE and the relation with HRQoL

**Methods:** Adults with cSLE were included in the CHILL-NL (CHILdhood Lupus-NetherLands) study via Dutch medical specialists and patient organizations. The study consisted of a single study visit containing a structured history and physical examination. Individual resilience capacities were assessed using the Brief Resilience Coping Scale (BRCS). This is a 4-item self-reported questionnaire that aims to assess the ability to handle stress in an adaptive manner, score range from 4 to 20: low-resilience ≤ 13, medium-resilience 14-16, high-resilience: ≥17. Coping strategies were assessed by the Assimilation-Accommodation Coping Scale (AACS). The AACS was utilized to measure coping methods, it consists of two subscales: tenacious goal pursuit (TGP) and flexible goal adjustment (FGA). Higher scores on one of the subscales indicate more use of that strategy. HRQoL was assessed with SF-36. Outcomes were compared to Dutch norm data.

**Results:** 106 cSLE patients (92% female, 73% white) were included, median age at study visit was 33 years and median disease duration 20 years. 61% had disease damage (SDI ≥ 1). HRQoL of cSLE patients was impaired (7/8 domains SF36): remarkably mental health was similar between patients and Dutch norm data. Surprisingly, HRQoL was not related to disease damage(7/8 domains SF36).

Low-resilient copers (BRCS) had impaired HRQoL in 2/8 domains of SF36 (general health and vitality), when compared to medium and high-resilient copers. Preference for a coping strategy (AACS) also influenced HRQoL: patients who applied tenacious goal pursuit (TGP) appeared to have impaired HRQoL (5/8 domains SF36) compared to patients preferring flexible goal adjustment. The extent to which the coping strategies were scored by patients on a scale from 0 to 60 seemed to relate to their resilience capacities with low-resilient patients having lower scores on the coping list.

Resilience measured by BRCS: Sinclair, V.G. & Wallston, 2004, Assessment. Individual coping measured by AACS: Brandtstädter & Renner, 1990, Psychology and Aging.

**Conclusion:** In this large cohort of adults with cSLE, self-reported resilience and coping capacities have an impact on HRQoL. This opens perspective to improve HRQoL with cognitive training methods focused on improving resilience and coping strategies in individual patients.

**Disclosure of Interest:** None Declared

### P133 Understanding the immunopathogenesis of juvenile-onset SLE using immune and metabolic phenotyping

#### George Robinson^1,2^, Marsilio Adriani^1^, Ines Pineda Torra^3^, Yiannis Ioannou^2^, Elizabeth Jury^1^ and Jury Group

##### ^1^Rheumatology, University college london, London, United Kingdom ^2^Centre for Adolescent Rheumatology, University college london, London, United Kingdom ^3^Clinical Pharmacology, University college london, London, United Kingdom

###### **Correspondence:** George Robinson

**Introduction:** Juvenile-onset systemic lupus erythematosus (JSLE) is an autoimmune disorder characterized by immune cell dysregulation, chronic inflammation and increased cardiovascular risk. Disease onset dominates mid-puberty and the female to male ratio is 4.5:1, suggesting a hormonal importance in disease pathogenesis. JSLE patients have more aggressive disease, more major organ involvement and increased standardised mortality ratios compared to patients with adult-onset SLE yet research into JSLE is uncommon. Our previous findings show that defects in immune cell lipid metabolism contribute to disease pathogenesis in adult-onset SLE. However, in JSLE little is known about the immune profile or whether abnormal lipid metabolism also contributes to pathogenesis.

**Objectives:** Since altered metabolism plays a role in adult-onset SLE our objective was to explore this in JSLE through in depth immune and metabolic phenotyping of a cohort of JSLE patients and age and gender matched healthy donors (HCs).

**Methods:** Flow cytometry was carried out using two 15-colour panels to immune-phenotype peripheral blood mononuclear cells from 39 healthy donors (HCs, 17 male, 22 female, mean age 18) and 35 JSLE patients (12 male, 23 female, mean age 19). Data was analysed by cluster and phenotype–phenotype correlation. Flow cytometry was also used to measure functional and metabolic marker expression on immune cell subsets. Data was correlated with clinical assessments of disease.

**Results:** Patients with JSLE were characterised by increased naïve and decreased memory B-cell and T-cell subsets and increased monocyte frequency (p = 0.0013) compared to HCs. Furthermore, phenotype-phenotype correlation analysis identified differential associations between naïve and memory immune cell subtypes when comparing the HC and JSLE cohorts.

CD4^+^ and CD8^+^ T-cells from JSLE patients had elevated membrane lipid raft (p = 0.0185, p = 0.0087) and glucose transport receptor (GLUT-1) (p = 0.0205, p = 0.0017) expression suggesting that they were more metabolically active. Metabolic defects were also found in monocytes and plasmacytoid dendritic cells. The expression of these metabolic markers on different subsets correlated with cell frequency suggesting a role of cell metabolism in driving the JSLE phenotype. Furthermore the metabolic immune-phenotype in JSLE correlated positively with disease activity, erythrocyte sedimentation rate and dsDNA titre and negatively with complement protein C3 supporting the hypothesis that altered metabolism is associated with JSLE development and severity.

Unsupervised hierarchical cluster analysis of patient clinical data revealed that JSLE patients in this cohort could be stratified into 5 groups each with a unique clinical identity mainly associated with disease activity markers and the presence of anti-cardiolipin antibodies. Each group had a unique immune-phenotype and metabolic profile.

**Conclusion:** Differences in the metabolic profiles of immune cell subsets in JSLE contribute to disease pathogenesis and severity. Cellular metabolic regulators may therefore have therapeutic benefit for JSLE patients. Defining these patient groups further may help to determine the therapeutic benefit of these and other therapeutics and allow for the treatment patients in a more effective and personalised manner.

**Disclosure of Interest:** None Declared

### P134 Hepatitis a virus vaccination in juvenile-onset systemic lupus erythematosus

#### Sevinc Mertoglu^1^, Sezgin Sahin^1^, Omer F. Beser^2^, Amra Adrovic^1^, Pelin Yuksel^3^, Soner Sazak^4^, Bekir S. Kocazeybek^3^, Ozgur Kasapcopur^1^

##### ^1^Pediatric Rheumatology, Istanbul University, Cerrahpasa Medical School, Istanbul, Turkey ^2^Pediatric gastroenterology, Okmeydani Education and Training Hospital, Istanbul, Turkey ^3^Microbiology, Istanbul University, Cerrahpasa Medical School, Istanbul, Turkey ^4^Pediatrics, Okmeydani Education and Training Hospital, Istanbul, Turkey

###### **Correspondence:** Sezgin Sahin

**Introduction:** Systemic lupus erythematosus (SLE) is an autoimmune disease and various infections play significant roles in flares of this chronic remitting-relapsing disease. Hepatitis A virus is one of these infectious agents that has high endemicity particularly in developing countries and countries with poor sanitary conditions. Hence, immunization via vaccination against this infectious agent would provide a better management of the disease. However, both immunosuppressive drugs and the disease itself are believed to impair the normal functioning of immune system. Little is known regarding the safety and immunogenicity of vaccinations in SLE patients. Moreover, to the best of our knowledge safety and efficacy of hepatitis A vaccination were not studied in children with SLE.

**Objectives:** In the present study, we aimed to compare the antibody titers and seropositivity in juvenile SLE and healthy subjects after hepatitis A vaccination. Besides, we examined the effect of immunosuppressive drugs and disease activity on antibody responses.

**Methods:** Sixty-nine juvenile SLE patients were enrolled in the study. Initially, we evaluated anti-HAV IgM and anti-HAV IgG titers in juvenile SLE patients. Of the 69 subjects, 37 patients were seronegative and eligible for hepatitis A vaccination. However, 7 juvenile SLE patients refused to participate to the study. Finally, anti-HAV Ig G negative 30 patients and 39 healthy subjects were vaccinated with two doses of hepatitis A vaccine (at 0 months and at sixth months). After vaccinations, anti-HAV Ig G titers were measured and compared between two groups.

**Results:** Anti-HAV Ig G concentrations were measured after vaccination in 30 patients with juvenile SLE and 39 control subjects. Anti-HAV Ig G titer of the juvenile SLE patients was significantly lower than that of the healthy controls (median 4.6 *versus* 11.9 IU/L, p = 0.02). Although the rate of seropositivity was lower in juvenile SLE patients (n = 24/30, 80%) compared to healthy controls (n = 33/39, 84.6%); this was not statistically significant (p = 0.6). No adverse reaction was reported after vaccination.

**Conclusion:** Although anti-HAV Ig G antibody titers after vaccination have found to be somewhat lower than that of the healthy subjects, significant portion of juvenile SLE patients were seropositive. According to these results, we conclude that hepatitis A vaccine is adequately immunogenic and quite safe in juvenile SLE patients.

References:

1. Borgia RE, Silverman ED. Childhood-onset systemic lupus erythematosus: an update. Curr Opin Rheumatol 2015; 27: 483-92.

2. Zandman-Goddard G, Shoenfeld Y. Infections and SLE. Autoimmunity 2005; 38: 473–85.

3. Wucherpfennig KW. Mechanisms for the induction of autoimmunity by infectious agents. J Clin Invest 2001; 108:1097–104.

4. Melhem NM, Talhouk R, Rachidi H, Ramia S. Hepatitis A virus in the Middle East and North Africa region: a new challenge. Journal of viral hepatitis 2014; 21(9): 605-615.

5. Aytac MB, Kasapcopur O, Aslan M, Erener-Ercan T, Cullu-Cokugras F, Arisoy N. Hepatitis B vaccination in juvenile systemic lupus erythematosus. Clin Exp Rheumatol 2011; 29: 882-6.

**Disclosure of Interest:** None Declared

### P135 Microangiopathy in childhood-onset systemic lupus erythematosus, a detailed further quantitative analysis of capillaroscopy abnormalities

#### Dieneke Schonenberg-Meinema^1,2^, J.M. vd Berg^1^, Amara Nassar-Sheikh-Rashid^1^, Godelieve de Bree^3^, A.E. Hak^4^, Marieke van Onna^4^, Karin Melsens^5^, Maurizio Cutolo^6^, T.W. Kuijpers^1^, Vanessa Smith^5,7^ and EULAR study group on microcirculation in rheumatic diseases

##### ^1^Department of Pediatric Hematology, Immunology, Rheumatology and Infectious Diseases, Emma Children’s Hospital, Academical Medical Center, Amsterdam, Netherlands ^2^Department of Pediatric Hematology, Immunology, Rheumatology and Infectious Diseases, Emma Children’s Hospital, Academical Medical Center (AMC), Amsterdam, Netherlands, ^3^Department of Infectious Diseases, ^4^Department of Clinical Immunology and Rheumatology, Academical medical center, Amsterdam, Netherlands, ^5^Department of Rheumatology, Ghent University Hospital, Ghent, Belgium, ^6^Research Laboratory and Academic Unit of Clinical Rheumatology, University of Genova, Genova, Italy, ^7^Faculty of Internal Medicine, Ghent University, Ghent, Belgium

###### **Correspondence:** Dieneke Schonenberg-Meinema

**Introduction:** Capillaroscopy findings can be qualitatively described as: normal, microangiopathy (non-specific abnormalities) or scleroderma pattern. Capillary abnormalities, described in varying prevalence in patients with systemic lupus erythematosus (SLE), are mainly described as microangiopathy with nonspecific capillary morphologic changes

**Objectives:** To describe the capillary morphologic abnormalities in a cross-sectional tertiary cohort of patients with childhood-onset SLE (cSLE) by a detailed quantitative assessment

**Methods:** Nailfold videocapillaroscopy (NVC) was performed in cSLE-patients (onset < 18 years) with a x200 magnification lens (Optilia). All fingers except the thumb were examined with four images per finger. The following capillaroscopic characteristics were evaluated per millimeter: density (compared to mean density known for age, sex and ethnicity), number of abnormal shapes (as defined by the EULAR study group on microcirculation in Rheumatic Diseases), giant capillaries (defined as apical diameter >50 mcm), maximum apical diameter (dilatations defined as apical diameter 20-50mcm) and microbleedings (categorized by large hemorrhages and small multiple point-shaped hemorrhages surrounding the capillary loop)

**Results:** This cohort of cSLE-patients was predominantly female (n = 19/22, 86.4%), with a median age of 14 years at onset of disease. At time of capillaroscopy, the median age was 17 years with a median disease duration of 29.3 months (IQR* 25-75: 12.7-56.5 months). Thirteen patients (59.1%) had an African/Afro-Caribbean ethnic background and seven (31.8%) a Caucasian. Median SLEDAI-score** at diagnosis was 11, and median SLEDAI-score at moment of capillaroscopy was 4.

In total, 4607 capillaries from 22 patients were analyzed. By qualitative analysis, 81.8% (n = 18) showed a pattern of microangiopathy. Quantitatively, all of these patients showed a specific combination (observed per finger) of three specific morphological capillary abnormalities: apical dilatations, small point-shaped hemorrhages and abnormal shapes. Looking at frequency and localization of these combined capillary abnormalities, three patients (3/22, 13.6%) showed them in all eight examined fingers, 54.5% (12/22) in four or more fingers and 72.7% (16/22) in three or more fingers. The fingers that were most affected were fourth finger left (n = 14), fourth finger right (n = 13) and fifth finger left (n = 13 patients).

*IQR = InterQuartile Range

**SLEDAI = Systemic Lupus Erythematosus Disease Activity Index

**Conclusion:** In this pilot (n = 22) of cSLE patients, 81.8% (n = 18) showed a combination of *specific* capillary morphological abnormalities: apical dilatations, small point-shaped hemorrhages and abnormal shapes. Up to 72.7% showed this combination in three or more fingers and 13.6% in all eight examined fingers. More studies in (c)SLE-patients are needed to assess if these capillary abnormalities are specific for SLE

**Disclosure of Interest:** None Declared

### P136 Timing of intravenous cyclophosphamide and long-term outcome in children with proliferative lupus nephritis

#### Tetsuya Tsuchida^1^, Asami Ohara^1^, Kenichi Nishimura^1^, Tomo Nozawa^1^, Ryoki Hara^1^, Shuichi Ito^1^

##### ^1^Department of Pediatrics, Yokohama City University Hospital, Yokohama, Japan

###### **Correspondence:** Tetsuya Tsuchida

**Introduction:** Induction therapy combined with corticosteroid and immunosuppressant from the acute phase improves long-term outcome in people with systemic lupus erythematosus (SLE). The prognosis of SLE is significantly improved by intravenous cyclophosphamide (IVCY) and mycophenolate mofetil (MMF). According to major guidelines for lupus nephritis (LN), IVCY and MMF are recommended as the first line remission induction therapy for adult proliferative LN (type III or IV), but the use of this strategy in childhood LN is not well established.

**Objectives:** To investigate if early remission induction therapy using IVCY can improve long-term outcome in childhood proliferative LN (type III or IV), by comparing patients treated with IVCY at onset versus later at disease flare.

**Methods:** Thirty-four children with SLE who were admitted to our institute from April 1997 to April 2016 were enrolled. Diagnosis of SLE was based on 1987 ACR criteria and diagnosis of LN was based on WHO classification or 2003 ISN/RPS classification. Patients were divided into two groups; group A: IVCY was used at the time of onset (n = 22), and group B: IVCY was used at the first recurrence (serological and/or clinical flare of SLE/LN) (n = 12). All patients successfully achieved remission once. We retrospectively evaluated parameters before and after IVCY including renal pathology, period to the first recurrence after IVCY, dose of prednisolone at the last observation, SLE disease activity index (SLEDAI), anti-double-strand DNA antibody (anti-dsDNAab), complement, urinary protein, estimated glomerular filtration rate (eGFR) and immunosuppressant as remission maintenance therapy.

**Results:** There was no significant difference in patient’s characteristics between the two groups. Type IV LN was more common than type III in both groups. The median number of IVCY was 8.5 (6–12) in group A and 8.5 (6–9) in group B (*p* = 0.83). Eleven of 22 patients had recurrence in group A (50%) and 8 of 12 patients had recurrence in group B (67%) after IVCY. The median period to the first recurrence after IVCY was 74 months in group A and 47.5 months in group B (*p* = 0.34, log-rank test). At the last observation, daily dose of prednisolone did not differ between groups A and B (0.100 vs. 0.155 mg/kg, *p* = 0.15). Additionally, there was no significant difference in titer of anti-dsDNAab, complement, SLEDAI, urinary protein, or eGFR. Regarding remission maintenance therapy after IVCY, 26 patients were treated with MMF (16 group A, 10 group B), and 8 patients were treated with other immunosuppressants such as azathioprine and mizoribine (6 group A, 2 group B). Of note, 12 of 26 patients treated with MMF (46%) and 7 of 8 patients on non-MMF immunosuppressants (88%) experienced recurrence (*p* = 0.039, log-rank test). Six of 16 patients on MMF in group A and 6 of 10 on MMF in group B experienced recurrence (*p* = 0.47). There were no deaths during the observation period.

**Conclusion:** In this study, timing of IVCY did not affect either prevention of recurrence or recurrence-free survival. Despite IVCY as induction therapy at onset, half the patients later had recurrence. Surprisingly, choice of MMF as maintenance therapy after IVCY is one factor to achieve longer remission. A recent meta-analysis showed that MMF was the most favorable maintenance therapy for adult SLE/LN^1)^. Our results were consistent with this finding and IVCY followed by MMF could be a favorable treatment for childhood proliferative LN. However, to avoid gonadal toxicity and malignancy due to IVCY, remission induction therapy with MMF for childhood proliferative LN should be evaluated by a prospective randomized-control trial in the future.

Reference

1)Palmer SC, Tunnicliffe DJ, Singh-Grewal D, Mavridis D, Tonelli M, Johnson DW, et al. Induction and Maintenance Immunosuppression Treatment of Proliferative Lupus Nephritis : A Network Meta-analysis of Randomized Trials. Am J Kidney Dis. 2017 Feb 20. pii: Cited in PubMed ; S0272-6386(17)30036-7. doi: 10.1053/j.ajkd.2016.12.008. [Epub ahead of print]

**Disclosure of Interest:** None Declared

### P137 Sexual differences in TLR7 driven interferon alpha production may explain the increased prevalence of JSLE in females after puberty

#### Kate Webb^1^, Gary Butler^2^, Madhvi Menon^3^, Hannah Peckham^1^, Ania Radziszewska^1^, Lucy R. Wedderburn^1^, Yiannis Ioannou^1^

##### ^1^Centre for Adolescent Rheumatology, UCL, London, United Kingdom ^2^Paediatric endocrinology, UCL, London, United Kingdom ^3^Medicine, UCL, London, United Kingdom

###### **Correspondence:** Kate Webb

**Introduction:** Juvenile onset systemic lupus erythematosus (jSLE) is an interferon alpha (IFNα) driven disease with a higher prevalence in females after puberty. The median ages of onset for puberty and jSLE are very similar, implying that puberty acts as a biological switch in the development of jSLE.

**Objectives:** 1.To investigate whether transition through puberty is associated with increased production of IFNα by plasmacytoid dendritic cells (pDC) in females compared to males. 2.To investigate whether interferon regulatory factor 5 (IRF5) and interferon stimulated gene expression (as measured by tetherin expression) change with puberty or sex.

**Methods:** Blood from heathy volunteers pre, during and post puberty, was collected and peripheral blood monocytes separated by ficoll gradient centrifugation. Ex vivo phenotype including cell type percentage, IRF5 expression and tetherin expression was assessed by flow cytometry. Cells were also separately stimulated with toll like receptor (TLR) 7 agonist R848 or TLR 9 agonist CPGODN2216, before assessing for the production of IFNα by pDC by flow cytometry.

**Results:** 60 young healthy volunteers between ages 6-18 years were recruited. pDCs from female volunteers produced significantly more IFNα than males upon TLR7 stimulation, overall (p = 0.01) and after puberty (p = 0.03) but not before puberty (p = 0.47).There was no difference in TLR9 stimulated pDCs between sexes across the age groups. In females, IRF5 expression after puberty was significantly reduced in pDCs (p = 0.02), monocytes (p = 0.04) and B cells (p = 0.03) when compared to males. After puberty, female pDCs (p = 0.04) and monocytes (p = 0.04) expressed significantly more tetherin (an interferon inducible gene protein product) than males.

**Conclusion:** Females produce more IFNα than males upon TLR7 stimulation after puberty, but not before. In addition, females express less IRF5 and may have a higher IFNα signature after puberty. These unique IFNα-related changes that occur in the immune system between sexes over puberty may account for the increased incidence of jSLE in females after sexual maturity and provide insights into the pathogenesis of jSLE.

**Disclosure of Interest:** None Declared

## Systemic lupus erythematosus and antiphospholipid syndrome

### P138 Similarities and differences between childhood-onset and adult-onset systemic lupus erythematosus in sultanate of Oman

#### Asma Al Rasbi^1^, Rabab Sultan^2^, Nisreen Abdallah^3^, Juma Al Kaabi^3^, Reem Abdwani^2^

##### ^1^SQU, Muscat, Oman ^2^Child Health, SQUH, Muscat, Oman ^3^Medicine, SQU, Muscat, Oman

###### **Correspondence:** Reem Abdwani

**Introduction:** SLE is a disease that mainly affects women of childbearing age, however, its prevalence is not confined within this population. A total of 15–20% of cases present in children under 16 years of age. Although adult onset SLE (aSLE) and childhood onset SLE (cSLE) share the same clinical features and diagnostic criteria, differences between the two groups have been identified.

**Objectives:** The aim of this study is to compare the similarities and differences in demographics, clinical presentation, serology, outcomes, treatment, disease severity and damage between aSLE and cSLE in an Omani cohort. This will be first study of this nature to be conducted from an Arab population from the Middle East region.

**Methods:** We evaluated 225 SLE patients, 139 adults and 86 children, followed at the pediatric and adult rheumatology department from January 2006 to July 2016 in Sultan Qaboos University Hospital, a tertiary and academic center in Oman. We included all patients with available clinical data at the disease onset in the hospital information system. cSLE was defined as children diagnosed at the age of 16 or below, while those diagnosed above the age of 16 were classified aSLE. All patients that were included in the study satisfied the 1997 American College of Rheumatology (ACR) Revised Criteria.

**Results:** The mean age at diagnosis of aSLE 28.1 years (SD **±** 8.8 yrs) and cSLE 9,4 years (SD **±** 4.2 yrs.). While the mean disease duration in aSLE 5.9 years (SD **±**4.4) and cSLE 8.9 years (SD **±** 5.7). There was greater female predominance of the disease with increasing age in cSLE. The F:M ratio in different age groups; less than 5 yrs, 5-12 year and 13-16 year were 2.5:1, 3.5:1, 6.7:1 respectively. While the female predominance in aSLE decreased with increasing age. The F:M ratio in different age groups; 17-25 years, 25-50 year and greater than 50 years were 11.1:1, 6.5:1 and 3:1 respectively. The distribution of SLE from various regions in the sultanate was proportionate to population density in aSLE as would be expected. However, there was higher prevalence of cSLE in A’Sharqiya, a region in the Sultanate of Oman with a relatively low population density. The clinical features in cSLE showed higher frequency of renal, musculoskeletal and pulmonary involvement; while aSLE showed higher frequency of hematological and mucocuatnous involvement as shown in Table 38. Serological differences demonstrated significantly higher frequency of antidsDNA antibody in cSLE, with higher frequency of anti-smith antibody in aSLE. The mean disease activity sindex (SLEDAI) at disease onset and over disease course was also higher in cSLE than aSLE (Table 38). cSLE were more likely to be treated with immunosuppressants such as cyclophosphamide and MMF than aSLE (54%, 21.5% vs 69.8%, 51.2%).

**Table 38:** **(Abstract P138).** Demographics clinical and laboratory characteristics of 225 SLE patients included in analysis

	aSLE 139(62%)	cSLE 86 (38%)	P Value
Cutanous	17 (12.2%)	0 (0.0%)	0.004
Arthritis	74 (53.2%)	58 (67.4%)	0.050
Renal	27 (19.4%)	42 (48.8%)	P < 0.0005
Hematologic	62 (44.6%)	19 (22.1%)	0.001
Neurologic	12 (8.6%)	15 (17.4%)	0.078
Pulmonary	4 (2.9%)	11 (12.8%)	0.009
SLEDAI at disease onset (Mean ± SD)	8.47	13.27	P < 0.05
SLEDAI over disease course (Mean ± SD)	11.77	16.34	P < 0.05

**Conclusion:** Differences between aSLE and cSLE in an Arabic cohort from Oman were different than the difference noted in other Caucasian cohort. It appears that individual races and ethnicities may exhibit differences in disease susceptibility and disease manifestations

**Disclosure of Interest:** None Declared

### P139 Neonatal lupus erythematosus: a 12-year retrospective study in Korea

#### Jong Gyun Ahn^1^, Dong Soo Kim^1^, Young Dae Kim^2^, Kwang Nam Kim^3^

##### ^1^Department of Pediatrics, Severance Children's Hospital, Yonsei University College of Medicine, Seoul, Korea, Republic Of ^2^Department of Pediatrics, Inje University School of Medicine, Seoul, Korea, Republic Of ^3^Department of Pediatrics, Hallym University Sacred Heart Hospital, Hallym University College of Medicine, Anyang, Korea, Republic Of

###### **Correspondence:** Jong Gyun Ahn

**Introduction:** Neonatal lupus erythematosus (NLE) is a rare autoimmune disease manifested commonly by cutaneous erythema and congenital heart block. NLE is presumed to result from transplacental passage of maternal autoantibodies of the Ro/La family.

**Objectives:** The aim of this study is to investigate clinical manifestations, treatment and outcomes of NLE.

**Methods:** Medical records of patients with NLE and their mothers from Severance children’s hospital in Korea between January 2015 and April 2017 were reviewed. A diagnosis of NLE was made if the infant had the presence of clinical symptoms plus positive anti-Ro/SSA or anti-La/SSB or both.

**Results:** There were 20 cases (male:female ratio of 8:12). Age of onset of clinical features was from birth to 68 days (median age of 7 days). Cutaneous lesions were seen in more than 90% of patients, while hepatobiliary, hematologogic, and cardiac abnormalities were present in 45%, 20% and 10% of cases, respectively. Anti-Ro/SSA and anti-La/SSB were positive in 100% and 65% of cases. There was no mortality during the study period. Most neonatal lupus erythematosus mothers (16 cases, 74.2%) had underlying autoimmune diseases (systemic lupus erythematosus in 11 cases and other autoimmune diseases in 5 cases). However, two of the mothers without pre-pregnancy autoimmune disease were found to have autoimmune antibodies in tests after the baby’s diagnosis.

**Conclusion:** NLE should be screened if infant have congenital heart block or skin rash with multi-system involvement despite the absence of history of maternal autoimmune disease. Most NLE patients without congenital heart block have relatively excellent outcome.

**Disclosure of Interest:** None Declared

### P140 Analysis of MEFV gene sequence variants and association with clinical features and disease activity in juvenile systemic lupus erythematosus patients

#### Ayşe Zopçuk^1^, Nuray Aktay Ayaz^1^, Betül Sözeri^2^, Mustafa Çakan^1^, Şerife Gül Karadağ^1^, Ayşenur Paç Kısaarslan^3^, Zübeyde Gündüz^3^

##### ^1^Pediatric Rheumatology, Kanuni Sultan Süleyman Research And Training Hospital, İstanbul, Turkey ^2^Pediatric Rheumatology, Ümraniye Research And Training Hospital, İstanbul, Turkey ^3^Pediatric Rheumatology, Erciyes University, Kayseri, Turkey

###### **Correspondence:** Nuray Aktay Ayaz

**Introduction:** FMF is a hereditary autoinflammatory periodic fever syndrome that is caused by mutations in the MEFV gene. It is common in Mediterranean countries and the carrier rate in MEFV gene in Turkey is reported to be around 20%. It has also been reported that in many chronic inflammatory diseases, the frequency of MEFV mutations is increased and the course of the disease may be altered.

**Objectives:** The aim of this study was to assess the frequency of MEFV gene sequence variants in patients with juvenile SLE and compare the clinical features, disease severity and course of the patients with or without MEFV sequence variants.

**Methods:** MEFV gene analysis was studied in 40 jSLE patients that were being followed in 2 pediatric rheumatology centers in Istanbul and Kayseri. None of the patients in the cohort had diagnosis of FMF.

**Results:** The frequency of MEFV polymorphisms and mutations in juvenile SLE patients was found to be 35% (Table 39). The most common of these was R202Q polymorphism (50%). The significance of the R202Q and E148Q sequence variants is controversial. After the exclusion of these variants, re-analysis revealed that the carrier rate was 12.5%.

**Table 39:** **(Abstract P140).** MEFV mutation analysis of SLE patients

	Mutation	n	Min	Max	Mean ± SD	Median	p
Age of Diagnosis (Year)	No	26	3	17	12,6 ± 3	13	0,79
	Yes	14	8	17	12,9 ± 2,8	13,5	
Duration of disease (month)	No	26	10	100	34,9 ± 19,8	31	0,69
	Yes	14	11	71	37,4 ± 16,8	42	
SLICC	No	26	0	3	0,4 ± 0,8	0	0,59
	Yes	14	0	2	0,5 ± 0,7	0	
SLEDAI	No	26	0	10	3,1 ± 3,2	2	0,62
	Yes	14	0	8	1,8 ± 2,5	1	

Age at onset of the disease, duration of the disease, SLICC and SLEDAI scores did not differ between the carrier and non-carrier groups. Hepatic involvement was found to be more frequent in MEFV mutation carriers (p = 0.037). There was no difference regarding fever, hematologic involvement, renal involvement, serositis, neuropsychiatric involvement, mucocutaneous findings, and vascular involvement in between the two groups.

**Conclusion:** In conclusion, we have seen that MEFV carrier rate was high in SLE patients. But we did not observe any differences in both clinical findings and disease damage in SLE patients with MEFV variant carriers except for hepatic involvement.

**Disclosure of Interest:** None Declared

### P141 Epidemiology, clinical characteristics and therapy approaches of a retrospective cohort of pediatric systemic lupus erythematosus in a tertiary centre

#### Rosa M. Alcobendas, Sara Murias, Agustin Remesal, Amelia Munoz Calongue

##### Pediatric Rheumatology, university hospital La Paz, Madrid, Spain

###### **Correspondence:** Rosa M. Alcobendas

**Introduction:** Systemic lupus erythematosus (SLE) is a chronic autoimmune disease, potentially severe, with broad clinical spectrum, which can affect multiple organs and systems.

**Objectives:** To analyze the initial manifestations, laboratory examinations and therapeutic approaches of patients diagnosed with childhood onset systemic lupus erythematosus (c-SLE) followed in a tertiary hospital in the last 14 years.

**Methods:** Retrospective chart review. Inclusion criteria were: children under 18 years diagnosed with c-SLE between January 2003 and January 2017.

**Results:** During the study period, 38 patients were identified (ratio female/male: 3/1). The mean age at the onset of disease was 11.5 years (range 6-17). All had caucasic origin, except 5 coming from South America, 2 from India and 1 from Africa. Onset of the disease took place in spring in 22 (58%) patients, while in 10 (26%) patients onset was in summer, 5 (13%) in autumn and only one (3%) in winter season.

The most frequently clinical manifestations found at the debut were cutaneous involvement (66%, predominantly in the form of a malar rash), renal (65%) and joint (60%, 48% arthralgia and 52% polyarthritis). Other manifestations were fever (50%), cytopenias (39%), asthenia (36%), serositis and neurological clinic (both 26%) and oral aphthosis (23%). Among the neurological manifestations, 4 patients showed bradypsychia, 3 headache, 2 seizures and 1 manifested a paralysis of the sixth cranial nerve. Only 1 patient presented macrophage activation syndrome after primoinfection by *Ebstein Barr Virus*. During the study period, no deaths ocurred.

Ten patients also had some other associated autoimmune disease: 6 hypothyroidisms, 2 IgA deficiency, 1 vitiligo and 1 celiac disease. No case of diabetes mellitus was identified.

Regarding immunology, in all cases, positive antinuclear antibodies (ANA) were detected, although with variable titers (1/80 - 1/5120). Of the 25 patients who presented renal disease at the onset, 19 associated Anti-dsDNA antibodies positive at the initial time of the determination. Although antibodies related to antiphospholipid syndrome (APS) (anticardiolipin, anti-2glycoprotein and lupus anticoagulant) were detected in 12 patients (32%), only two developed associated clinical manifestations (both deep vein thrombosis in the lower limbs).

Regarding treatment, all patients required corticosteroids. Therapy with acetylsalicylic acid was indicated in all patients with APS-associated immunology. Only 6 patients received treatment with corticosteroids and hydroxychloroquine exclusively. Nineteen (50%) patients initially received azathioprine therapy, being necessary to switch to mycophenolate mofetil for lack of response in eleven, receiving the last treatment 20 patients finally (52%). Eighteen patients (47%) received cyclophosphamide therapy, 16 of them as a consequence of their renal involvement. In addition, biologic therapy (rituximab and belimumab, respectively) was used in two multirefractory patients.

**Conclusion:** As widely already reported, SLE is a disease that affects predominantly women. Moreover, as it has been previously described in the literature the most frequently initial manifestations found in c-SLE are cutaneous, renal and articular. However, a large variability of onset symptoms exists, thus c-SLE should be ruled out in patients with multisystemic involvement.

**Disclosure of Interest:** None Declared

### P142 Histological grading of lupus nephritis is related to early change in growth parameters.

#### Yuri A. Arguello^1^, Giovanni Filocamo^1^, Sofia Torreggiani^1^, Valentina Litta Modigliani^1^, Giani Marisa^1^, Giovanni Montini^1^

##### ^1^Medicine and Surgery Faculty, Università degli Studi di Milano, Milan, Italy

###### **Correspondence:** Yuri A. Arguello

**Introduction:** Childhood Systemic Lupus Erythematosus (cSLE) is a multisystemic chronic autoimmune disease characterized by a wide spectrum of clinical manifestations. Renal involvement is one of the most common manifestations of cSLE, reported in 40-80% of patients. Growth failure and delayed puberty are features of cSLE, caused by long term disease activity, and side effects of drugs, especially corticosteroids.

Objectives:

To assess the early influence of SLE Lupus Nephritis disease and treatment on growth parameters in children.

**Methods:** Patients diagnosed with cSLE in a tertiary care center in the past 22 years, with kidney biopsy-proven Lupus Nephritis were included in our study. Patients were excluded if incomplete data were available. The WHO classification was considered to evaluate histological findings. Patients were divided by histological class in two groups: the mild group (Class II-III) and the severe group (Class IV-V).

Height (cm) and Weight (kg) were measured with type of stadiometer specified (Harpenden, wall-mounted). BMI (Body Mass Index) was calculated as weight (kg) divided by height (meter) squared. We assessed the anthropometric parameters using Atlanta 2000 curves. Student t test has been calculated for continuous variables to compare the two groups. In all analyses, P < 0.05 was taken to indicate statistical significance.

**Results:** From 1994 to 2016, 45 children with lupus nephritis (LN) were biopsied. 15 of them presented incomplete clinical data and were excluded. Female-to-male ratio was 3:1., the mean age at onset was 11.5 ± 3.1 years. The most common class of Lupus Nephritis was considering WHO classification class IV (40%), followed by class III (36.7%), II (16.7%) and V (6.7%). We compared all parameters (height, weight, BMI percentiles) when first renal biopsy was performed and after 12 months.

**Conclusion:** This study compared patients with mild and severe histological assessed LN in the first year of disease. Considering height percentile, mild LN patients didn’t have a substantial growth speed deflexion, while severe LN patients had a mild decrease even in the first year of disease. Comparing weight percentiles, we observed in both groups an increase of weight, more accentuated in severe LN patients.

The BMI percentile of patients with mild LN didn’t register a significant mean gain, whereas severe LN patients had a statistical relevant gain of BMI percentile after 1 year (p value 0.027)

A higher kidney-biopsy grade seems to be related to early change in growth parameters, even if statistical significativity was reached only for BMI.

Data collection is still ongoing in order to define the influence of the treatment performed and to define if early change in growth parameters is a related to the long term growth disturbance.

Trial registration identifying number:

**Disclosure of Interest:** None Declared

### P143 Is abnormality of lipid profile associated with more severe histological findings at renal biopsy in children with lupus nephritis ?

#### Francesco Baldo^1^, Valentina Litta-Modignani^1^, Sofia Torreggiani^1^, Carlo Virginio Agostoni^1^, Marisa Giani^1^, Giovanni Montini^1^, Giovanni Filocamo^1^

##### ^1^Fondazione IRCCS Ca' Granda Ospedale Maggiore Policlinico, Milan, Italy

###### **Correspondence:** Francesco Baldo

**Introduction:** Childhood Systemic Erythematosus Lupus (cSLE) is a severe multisystem autoimmune disease. Renal involvement is a major cause of morbidity and mortality in cSLE. Serum triglyceride, total cholesterol, low density lipoprotein (LDL) and apolipoprotein B (apoB) concentrations were reported significantly higher in patients with lupus nephritis (LN). It is unclear if abnormality in lipid profile is related to the severity of renal function, the steroid therapy or both.

**Objectives:** To evaluate the lipid profile in children with Lupus Nephritis (LN) at onset and after one year of treatment and to investigate if different lipid profile can be related to the severity of renal involvement.

**Methods:** We performed a retrospective analysis of children with cSLE and nephritis confirmed at renal biopsy, followed since 1994 to 2016 in a single tertiary care center.

Lipid profile (total cholesterol, LDL, HDL and triglycerides), serum albumin level and proteinuria were researched at disease onset (T1) and after one year of disease duration (T2).

Proteinuria was measured as the quantity of protein in a 24-hour urine collection test, alternatively, the concentration of protein in the urine was reported as protein/creatinine ratio.

The WHO classification of LN was used to grade histological findings.

Renal biopsy findings were grouped into two categories: mild (for class I-III) and severe (class IV-VI).

Dependent T test was performed to evaluate the differences between the disease onset and the second evaluation. Independent T test was used to compare the results between mild and severe bioptic classes. P < 0.05 was taken to indicate statistical significance.

**Results:** From 1994 to 2016, 45 children with lupus nephritis (LN) were biopsied. 15 of them presented incomplete clinical data and were excluded. In the 30 patients enrolled in the study, the female-to-male ratio was 3:1, the mean age at onset was 11.5 ± 3.1 years. The mean disease duration since cSLE onset until renal biopsy was 10 and 19 months for mild and severe involvement respectively.

Patients were distributed among the histological classes as follows: Class II: 5 patients (16.7%), Class III: 11 patients (36.7%), Class IV: 12 patients (40%) and Class V: 2 patients (6.7%).

Daily proteinuria resulted 672 ± 550 mg/die for mild class and 2475 ± 3737,5 g/die for severe class. The urine protein/creatinine ratio resulted 1,08 ± 1,39 and 1,34 ± 1,19 for mild and severe class respectively, the difference was not significant.

Results are displayed in Table 40.

**Table 40:** **(Abstract P143).** See text for description

	T1		T2	
	Mean ± (standard deviation)			
	Mild	Severe	Mild	Severe
Albumin (mg/dl)	3,4 (± 0,8)	3,7 ± (0,5)	4 ± (0,9)	3,7 ± (0,6)
Total cholesterol (mg/dl)	203,5 (± 68,5)	187,7 ± (55,5)	197,2 ± (51,4)	222,5 ± (52)
HDL-cholesterol (mg/dl)	57,4 (± 37,4)	39,9 ± (16,2)	69 ± (28,3)	52,5 ± (11,4)
LDL-cholesterol (mg/dl)	102,6 (± 30,1)	130,2 ± (32,5)	127,5 ± (40,3)	124,8 ± (33,5)
Triglycerides (mg/dl)	164,7 (± 116,2)	178,5 ± (163,4)	129 ± (39,3)	172,1 ± (99)

**Conclusion:** Total cholesterol level at onset resulted higher in patients with milder renal disease, even if dyslipoproteinemia resulted more significant in the severe group because of an higher level of LDL and triglycerides. After one year of disease duration the differences in lipid profile between the two groups became less relevant.

**Disclosure of Interest:** None Declared

### P144 Clinical and immunological characteristics of childhood-onset systemic lupus erythematosus patients treated with rituximab

#### Alina Lucica Boteanu, Maria Angeles Blazquez Cañamero, Adela Alia Jimenez, Sandra Garrote Corral, Maria Jesus García Villanueva, Mariluz Gamir Gamir

##### Rheumatology, University Hospital Ramon Y Cajal, Madrid, Spain

###### **Correspondence:** Alina Lucica Boteanu

**Introduction:** Systemic lupus erythematosus (SLE) is an autoimmune disease that is more severe in pediatric population than in adults. Biological therapy with anti-CD20 (rituximab) is an option in patient that do not respond to conventional therapy.

**Objectives:** The aim of this study is to determine the clinical and immunological response in 9 patients with childhood-onset systemic lupus erythematosus (cSLE) that received treatment with rituximab in a third level hospital.

**Methods:** This is a retrospective observational study. 9 patients treated with Rituximab between November 2007 and October 2016 were included and their medical records were reviewed. The response to treatment at 6 months and one year after the first infusion of Rituximab were assessed. Patients with overlap syndromes were excluded. All patients fulfilled four or more of the 1982 revised American College of Rheumatology criteria for the diagnosis of SLE (< 16 years).

**Results:** Nine pediatric patients with SLE treated with rituximab were included, all of them were female. The age at diagnosis of SLE was a mean of 15,22 years. The mean time duration of disease was 87,55 months (5-255 m). 7 patients were caucasians. Rituximab was indicated in 6 patients with class IV of lupus nephritis (LN) 1/9 with class III LN, 1/9 with severe cutaneous lupus, and with severe hematological manifestations in 1 case (haemolytic anemia). In addition, 6/9 patients had mucocutaneous and articular manifestations. The disease activity of all patients was assessed using SELENA-SLEDAI index pre rituximab infusion, the mean was 17,11 (8-33). All patients had low level of complement C3 and C4 and 8/9 increased anti-DNA. In 8/9 patients Rituximab was used as a rescue treatment and in a single case as a first line. 3/6 patients with renal involvement were previously treated with cyclophosphamide (CF) iv and mycophenolate, 2/6 CF. In case of cutaneous involvement the previous treatment was methotrexate, azathioprine (AZA) and dapsone and in case of hemolytic anemia was AZA.The treatment protocol was 1 gram x 2 (1 cycle) in 7/9 patients, 375 mg/m2x 4 in 1/9 cases and 600 mg monthly for 5 months in the case of hemolytic anemia. Five patients received more than 1 cycle. After the administration of Rituximab, the SELENA-SLEDAI activity index was 4.5 points. At 6 months a complete response was obtained in the case of hematological and cutaneous manifestations, in 2 cases of lupus nephritis (proteinuria <0.5 g/day) and partial response was obtained in 2 cases. Data were not analyzed in 2 patients (death and less than 6 months of the first dose of rituximab). Patients with partial response and lack of response achieved complete response at 12 months. 2/9 patients had side effects (Rituximab pneumonitis in 1 case and infections in 2 cases). Mortality was 11.11% (1/9 patients, per infection and lupus activity, SLEDAI pre rituximab = 33)

**Conclusion:** In our study, although it consisted of few patients, it was objected that Rituximab therapy in patients with cSLE is effective, reduces lupus activity index, especially in cases of renal, cutaneous and hematologic involvement, that don’t respond to conventional therapy. It may be consider in the future as an effective alternative treatment at first line treatment.

**Disclosure of Interest:** None Declared

### P145 Panniculitis in childhood-onset systemic lupus erythematosus: a multicentric cohort study

#### Lucia M. Campos^1^, Mônica Verdier ^2^, Pedro Anuardo ^2^, Natali Gormezano ^1^, Ricardo Romiti ^3^, Nadia Aikawa ^2^, Rosa Pereira ^2^, Maria T. Terreri ^4^, Claudia Magalhães ^5^, Juliana Ferreira ^1^, Marco Silva ^1^, Mariana Ferriani ^1^, Ana P. Sakamoto ^4^, Virginia Ferriani ^6^, Maraísa Centeville ^7^, Juliana Sato ^5^, Maria C. Santos ^8^, Eloisa Bonfá ^2^, Clovis Silva ^1^

##### ^1^Paediatric Rheumatology Unit, Children’s Institute, University of São Paulo, São Paulo, Brazil ^2^Rheumatology Department, University of São Paulo, São Paulo, Brazil ^3^Dermatology Department, University of São Paulo, São Paulo, Brazil ^4^Paediatric Rheumatology Unit, Universidade Federal de São Paulo, São Paulo, Brazil ^5^Paediatric Rheumatology Unit, Faculdade de Medicina de Botucatu, Botucatu, Brazil ^6^Paediatric Rheumatology Unit, Ribeirão Preto Medical School – University of São Paulo, São Paulo, Brazil ^7^Paediatric Rheumatology Unit, University of Campinas, Campinas, Brazil ^8^Paediatric Rheumatology Unit, Santa Casa de Misericórdia de São Paulo, São Paulo, Brazil

###### **Correspondence:** Lucia M. Campos

**Introduction:** Lupus erythematosus panniculitis (LEP) is a rare form of chronic cutaneous lupus erythematosus described from 2% to 5% of adult SLE. In cSLE, LEP data are limited to few case reports

**Objectives:** To evaluate prevalence, clinical manifestations, laboratory abnormalities, treatment and outcome in a multicenter cohort of childhood-onset systemic lupus erythematosus(cSLE) patients with and without panniculitis.

**Methods:** Panniculitis was diagnosed due to painful subcutaneous nodules and/or plaques in deep dermis/subcutaneous tissues and lobular/mixed panniculitis with lymphocytic lobular inflammatory infiltrate in skin biopsy. Statistical analysis was performed using Bonferroni correction(p < 0.004).

**Results:** Panniculitis was observed in 6/847(0.7%) cSLE. Painful subcutaneous erythematosus and indurated nodules were observed in 6/6 panniculitis patients and painful subcutaneous plaques in 4/6. Generalized distribution was evidenced in 3/6 and localized in upper limbs in 2/6 and face in 1/6. Histopathology features showed lobular panniculitis without vasculitis in 5/6(one of them had concomitant obliterative vasculopathy due to antiphospholipid syndrome) and panniculitis with vasculitis in 1/6. Comparison between cSLE with panniculitis and 60 cSLE without panniculitis with same disease duration [2.75(0-11.4) vs. 2.83(0-11.8) years,p = 0.297], showed higher frequencies of constitutional involvement (67% vs. 10%,p = 0.003), leukopenia (67% vs. 7%,p = 0.002) and median C-reactive protein (10.5 vs. 0.5 mg/L,p = 0.001). Cutaneous atrophy and hyperpigmentation occurred in 83% of patients.

**Conclusion:** Panniculitis is a rare skin manifestation of cSLE occurring in the first three years of disease with considerable sequelae. The majority of patients have concomitant mild lupus manifestations.

**Disclosure of Interest:** None Declared

### P146 Prevalence of ena and anti cardiolipin antibodies in different classes of lupus nephritis.

#### Giancarla Di Landro, Francesco Baldo, Valentina Litta Modignani, Marisa Giani, Giovanni Montini, Carlo Virginio Agostoni, Giovanni Filocamo

##### Fondazione IRCCS Ca' Granda Ospedale Maggiore Policlinico, Milan, Italy

###### **Correspondence:** Giancarla Di Landro

**Introduction:** Childhood Systemic Lupus Erythematosus (cSLE) is a rare autoimmune disease. Lupus nephritis (LN) is one of the most serious manifestations of cSLE, associated with poor prognosis and reported in 40-80% of patients at onset disease. Extractable nuclear antigen (ENA) antibody can be detected in SLE and some of them are related to a distinct clinical subset of disease, independently of their frequency. Autoantibodies against Smith antigen (Sm) are reported in high prevalence in patients with active LN. It is unclear if the prevalence of ENA autoantibodies is related to the severity of renal involvement.

**Objectives:** To evaluate if ENA antibodies positivity in early stage of SLE disease is associated with the development of more severe disease, assessed by histological finding.

**Methods:** We performed a retrospective analysis of children with cSLE and nephritis confirmed by renal biopsy, followed since 1994 to 2016 in a single tertiary care center.

Clinical features, renal involvement and biopsy findings were collected from patients files as well as the positivity of ENA antibodies (Anti-RNP, Anti-Sm, anti-Ro/SS-A, anti-La/SS-B, anti-Scl-70, anti-Jo1, anti-nucleosome, anti-histone), Lupus Anti Coagulant (LAC) test and Anti- cardiolipin antibodies (ACL).

The WHO classification of LN was used to grade histological findings.

Renal biopsy findings were grouped into two categories: mild (for class I-III) and severe (class IV-VI).

Chi-Squared Test was used to to determine significant relationship between categorical variables. P < 0.05 was taken to indicate statistical significance.

**Results:** From 1994 to 2016, 45 children with LN were biopsied. In 22 of them ENA screening was not reported in clinical records and they were excluded from the analysis. Between the 23 remaining patients, 12 were included in the mild group, the remaining patients in the severe group; 1 from the severe class had record of ENA positivity, but not of the specif pattern.

In the 23 patients enrolled, the female-to-male ratio was 2,6:1, the mean age at onset was 11.75 ± 3.2 years. The mean disease duration up to renal biopsy was 15 and 19 months for mild and severe involvement respectively.

ACL and LAC screening record were found for 21 and 20 patients respectively. LAC was present in 19,1% of patients and IgM and IgG anti-cardiolipin were present in 40% of patients. ENA were positive in 13 out 23 patients (56.5%). Overall the patients enrolled, the most common positive ENA subtypes were, anti-Ro/SS-A (38%), followed by anti-Sm and anti-RNP(22,7%). The prevalence of ENA in patients with mild and severe renal involvement are reported in Table 41.

**Table 41:** **(Abstract P146).** See text for description

AUTOANTIBODIES	MILD		SEVERE		p value
ENA:	7	63,63%	6	50,00%	0,509
anti-RNP	3	27,27%	2	18,18%	0,611
anti-Sm	2	18,18%	3	27,27%	0,611
anti-Ro/SS-A	5	45,45%	4	36,36%	0,665
anti-La/SS-B	1	9,09%	3	27,27%	0,269
Scl-70	1	9,09%	1	9,09%	1
Anti-nucleosome and anti-histone	1	9,09%	0	0	NS
LAC	2	20,00%	2	18,18%	0,91
IgM and IgG aCL > 10	4	40,00%	4	40,00%	1

**Conclusion:** ENA autoantibodies are present in about half of the patients with LN. The prevalence of ENA, ACL and LAC does not differ significantly in patients with different grade of renal involvement.

**Disclosure of Interest:** None Declared

### P147 Juvenile systemic lupus erythematosus: clinical and immunological patterns of disease expression in a cohort of Mexican children.

#### Sofia Osorio, Andrés Rodríguez, Rocio Maldonado, Enrique Faugier, Talia Diaz, Yuridiana Ramirez, Luis Aparicio, Maria Braña

##### Pediatric Rheumatology, Hospital Infantil de Mexico Federico Gomez, Ciudad de mexico, Mexico

###### **Correspondence:** Talia Diaz

**Introduction:** Juvenile systemic lupus erythematosus is a chronic multisystem autoimmune disease of unpredictable course and prognosis. It manifests with a wide spectrum of clinical and immunological abnormalities. To date, no literature has been described about the most frequent clinical manifestations in mexican children with this disease.

**Objectives:** To define the pattern of disease expression in subjects with juvenile systemic lupus erythematosus in Mexico and compare it with what has been reported in other series, in addition to gain a better understanding of juvenile systemic lupus erythematosus in mexican children.

**Methods:** The features of 150 patients with juvenile systemic lupus erythematosus who had disease onset before the age of 18 years in the Children’s Hospital of Mexico < <Federico Gómez>>, were retrospectively analysed. Demographic, clinical and laboratory manifestations, therapy were assessed.

**Results:** A cohort of 150 patients with a mean age at diagnosis of 11.3 ± 3.13 years and a mean period of follow-up of 3.34 ± 2.14 years were analyzed. One hundred thirty-one (87.3%) patients were female. The most common manifestations were mucocutaneous (74.7%), hematological (56%) and musculoskeletal (39.3%) abnormalities. Upon diagnosis, renal damage was found in 56 patients (37.3%), of which 28 (18.3%) were diagnosed with lupus nephritis. Antinuclear and double-stranded anti-DNA antibodies were positive in most patients, 96.7% and 77.3%, respectively; antiphospholipid antibody positivity was observed in 40.7%. During follow-up, immunosuppressive management consisted of azathioprine in 54%, cyclophosphamide in 66.6%, mycophenolate mofetil in 31%, a combination of more than one of the above in 56.7%, being the most frequent azathioprine and cyclophosphamide; 22 individuals (14%) were only treated with hydroxychloroquine as an immunomodulator.

**Conclusion:** This study suggests that in our patients the clinical and laboratory features observed were similar to juvenile systemic lupus erythematosus patients from other series.

**Disclosure of Interest:** None Declared

### P148 Neonatal lupus - case series of a tertiary hospital

#### Ana R. Teixeira ^1^, Raquel Ferreira^1, 2^, Mariana Rodrigues ^3, 4^, Francisca Aguiar ^1, 2^, Iva Brito ^2, 5^

##### ^1^Faculty of Medicine, University of Porto, Porto, Portugal ^2^Rheumatology, Centro Hospitalar de São João, Porto, Portugal ^3^Pediatrics, Centro Hospitalar de São João, Porto, Portugal ^4^Pediatrics, Faculty of Medicine, University of Porto, Porto, Portugal ^5^Medicine, Faculty of Medicine, University of Porto, Porto, Portugal

###### **Correspondence:** Raquel Ferreira

**Introduction:** Neonatal Lupus (NL) is a rare condition caused by the transplacental passage of autoantibodies anti-Ro/SSA, anti-Sa/SSB and/or anti-U1 RNP antibodies into the fetal circulation which affects newborns. The mother may be asymptomatic or have a known autoimmune disorder, like Sjögren syndrome or Systemic Lupus Erythematosus. Clinical manifestations are diverse and have varying severity, the most common being cutaneous and cardiac.

**Objectives:** Identify NL cases, describe the clinical manifestations and its course, evaluate the presence of the most important antibodies and the history of mother’s disease.

**Methods:** Retrospective review of case files of all patients diagnosed with NL in the last eight years, admitted to the Neonatal Unit and/or followed at the outpatients department of a tertiary teaching hospital.

Results

The authors present a case series which includes eight cases diagnosed with NL and a brief review of the literature (Table 42).

**Table 42:** **(Abstract P148).** Clinical and Laboratory Characteristics; F, Female; M, Male; SS, Sjögren Syndrome; SLE, Systemic Lupus Erythematosus; NE, Not Evaluated

	Case 1	Case 2	Case 3	Case 4	Case 5	Case 6	Case 7	Case 8
Gender	F	F	M	F	F	F	M	M
Maternal history	SS	LES	LES	LES	SS	SS	SS, LES	SS
Dermatological involvement	No	No	No	No	No	No	Yes	Yes
Cardiac involvement	Yes	Yes	Yes	Yes	Yes	Yes	Yes	No
Other organs involvement	No	Yes	Yes	No	No	No	No	No
Child's autoantibodies	NE	Anti-SSA and SSB	Anti-SSA	NE	NE	Anti-SSB	Anti-SSA and SSB	NE
Maternal autoantibodies	Anti-SSA	Anti-SSA and SSB	Anti-SSA and SSB	Anti-SSA	Anti-SSA and SSB	Anti-SSA and SSB	Anti-SSA and SSB	Anti-SSA and SSB

**Conclusion:** Underdiagnosis might explain the reduced number of patients identified. The positivity of anti-Ro/SSA and anti-Sa/SSB seems to be associated with higher prevalence of neonatal cardiac manifestations. Early detection of this condition is paramount since treatment may reverse lower grade HB. Also, adequate maternal treatment can reduce the likelihood of NL.

**Disclosure of Interest:** None Declared

### P149 Gender differences in systemic lupus erythematosus presentation and treatment at paediatric rheumatology department, Sofia, Bulgaria for a period of 4 years (2013-2017)

#### Margarita Ganeva, Stefan Stefanov, Albena Telcharova, Dimitrina Mihaylova, Vanya Kostova, Katya Temelkova, Tanya Andreeva

##### Paediatric Rheumatology, University Children's Hospital, Medical University Sofia, Sofia, Bulgaria

###### **Correspondence:** Margarita Ganeva

**Introduction:** The prevalence of systemic lupus erythematosus (SLE) is known to be lower in boys than in girls. The female-to-male ratio of paediatric SLE (pSLE) is 4.5-5:1. Much controversy surrounds the differences in disease manifestation in both sexes.

**Objectives:** The aim of this retrospective study is to compare the gender differences in the age of onset, time to diagnosis, clinical and laboratory manifestations and medication use in pSLE at presentation.

**Methods:** Seventeen patients (11 boys and 6 girls) with newly diagnosed pSLE admitted to the Paediatric Rheumatology Department, Sofia, Bulgaria for a period of 4 years (2013-2017) were included in the study. The medical records of all patients were retrospectively reviewed. Patients fulfilled the American College of Rheumatology (ACR) SLE classification criteria.

**Results:** The female-to-male ratio in the described cohort of patients is reversed - 3.6:6.4. The mean age of onset in male pSLE patients was 12.59 yrs (5.83-17.83 yrs) with mean time to diagnosis of 2 ± 1.3 months. The mean age of onset in female patients was 14.06 yrs (7.5-16.33 yrs) with mean time to diagnosis – 3.5 ± 3.99 months. No statistically significant differences were found between boys and girls with regard to mean age of onset, mean time to diagnosis and clinical and laboratory manifestations of pSLE. The most common clinical sign at presentation in boys was arthritis (9/11; 81.8%), followed by renal involvement (7/11; 63.6%). Girls presented most frequently with malar rash (5/6; 83.3%), followed by arthritis (3/6; 50%). Renal involvement at presentation was detected in only one of the girls (1/6; 16.6%). Serositis was noted as presenting manifestation in one girl (1/6; 16.6%) and was not observed in any of the boys. Vascular thrombosis was seen in one boy (1/11; 9.1%). No neurological manifestation was observed in both groups. The most frequently detected hematological manifestation in the male group was lymphopenia (7/11; 63.6%) with leucopenia being the most common presenting laboratory sign in the female group (5/6; 83.3%). The least commonly observed laboratory sign in both groups was Coombs-positive autoimmune hemolytic anemia. Positive anti-dsDNA antibodies were detected in 90.9% of the boys and 100% of the girls. Antinuclear antibodies were positive in 100% of the boys and 83.8% of the girls. Low complement levels were seen in 81.8% of the males and 83.8% of the girls. All patients received corticosteroid therapy with intravenous pulse therapy performed in one girl and three boys. Cyclophosphamide for the initial 6-month induction period was used in 50% of the girls and 63.6% of the boys. Azathioprine was given to one girl and two boys. Chloroquine was used in 33.3% of the girls and 18.1% of the boys.

**Conclusion:** A male predominance of SLE has been observed. The mean age at disease onset and mean time interval from disease onset to diagnosis did not differ in male and female patients. The observed clinical and laboratory manifestations were also comparable between the two groups. Arthritis and renal involvement were found to be the commonest clinical manifestation among boys. Renal involvement favored males, however the result was not statistically significant.

**Disclosure of Interest:** None Declared

### P150 Differences of the metabolome of autoimmune diseases

#### Anna E. A. Glaser^1^, Angela Midgley^1^, Helen L. Wright^2^, Marie M. Phelan^3,4^, Matthew Peak^5^, Michael W. Beresford^1,5^

##### ^1^Department of Women’s and Children’s Health, University of Liverpool, Liverpool, United Kingdom ^2^Department of Biochemistry, University of Liverpool, Liverpool, United Kingdom ^3^Institute of Integrative Biology, University of Liverpool, Liverpool, United Kingdom ^4^HLS Technology Directorate, University of Liverpool, Liverpool, United Kingdom ^5^Department of Paediatric Rheumatology, Alder Hey Children's NHS Foundation Trust, Liverpool, United Kingdom

###### **Correspondence:** Anna E. A. Glaser

**Introduction:** Both juvenile-onset systemic lupus erythematosus (JSLE) and juvenile idiopathic arthritis (JIA) are autoimmune diseases which can affect multiple targets in different organs of the body. Metabolomics studies are a comprehensive way to investigate reactions and interactions of different, potentially important cells contributing to the immunopathogenesis of these disorders.

**Objectives:** To compare the metabolite profiles of serum and urine of patients with JIA and JSLE as well as non-inflammatory paediatric control patients.

**Methods:** Serum and urine samples were obtained from children (diagnosed <17 years of age) with JIA (n = 5 for serum, n = 4 for urine), with JSLE (n = 10, n = 8) and from paediatric healthy controls (n = 9, n = 4). The samples were run at 310 K for serum and at 300 K for urine on a Bruker 600 MHz AvanceIII with CryoProbe. Resulting 1H NMR spectra were analysed with Topspin, Chenomx NMR Suite and R.

**Results:** In serum global changes in spectra analysed via Partial Least Squares Discriminant Analysis (PLSDA) revealed variation between JIA, JSLE and control patients. While JSLE and control patients were separated, the JIA patients fell in-between and a clear clustering was therefore not possible.

Cross-validation accuracy for this PLSDA was 0.67 with a robustness (R^2^) 0.88 and a prediction (Q^2^) of 0.25 with optimal model using four linear discriminants. Univariate analysis using ANOVA showed that Acetoacetate was significantly higher (p < 0.05) in JIA patients compared to healthy controls (1.8-fold) and JSLE patients (1.8-fold). Alanine on the other hand was significantly lower (p < 0.05) with a 0.8-fold change in JIA patients compared to healthy controls and JSLE patients. Pyruvate on the other hand was significantly increased (p < 0.05) in JSLE patients compared to JIA patients (1.4-fold) and healthy controls (1.3-fold).

These differences in the serum indicate that these diseases utilize different pathways to generate energy.

In urine, PLSDA of the spectra resulted in a clear separation of the three groups with a cross-validation accuracy of 0.75 an R^2^-value of 0.95 and Q^2^–value of 0.38 with the optimal model using three linear discriminants. Metabolites contributing to this separation included hippurate, taurine, citrate and 4-hydroxybenzoate.

**Conclusion:** Variation in metabolic profiles was observed in JIA and JSLE patients compared to paediatric healthy controls. Analysis of serum and urine indicate urine to be a better biofluid sample to distinguish between diseases. Investigating changes of metabolites in serum samples gave us indications of which pathways are differentially regulated between the three conditions. Further analyses are being undertaken to investigate which pathways are regulated differently between the two diseases.

**Disclosure of Interest:** None Declared

### P151 Primary antiphospholipid syndrome in children – case series from Chennai, South India

Abstract withdrawn

### P152 Novel urine biomarkers for the assessment of pediatric systemic lupus erythematosus nephritis

#### Artemis Koutsonikoli^1^, Maria Trachana^1^, Evangelia Farmaki^1^, Vasiliki Tzimouli^1^, Polyxeni Pratsidou-Gertsi^1^, Nikoleta Printza^1^, Alexandros Garyphallos^2^, Vasiliki Galanopoulou^3^, Florence Kanakoudi-Tsakalidou^1^, Fotios Papachristou^1^

##### ^1^First Department of Pediatrics, Aristotle University of Thessaloniki, Hippokration Hospital, Thessaloniki, Greece ^2^Fourth Department of Internal Medicine, Aristotle University of Thessaloniki, Hippokration Hospital, Thessaloniki, Greece ^3^Department of Rheumatology, Papageorgiou Hospital, Thessaloniki, Greece

###### **Correspondence:** Artemis Koutsonikoli

**Introduction:** Novel urine biomarkers, with a proven specificity for pediatric Lupus Nephritis (pLN), will facilitate the non-invasive and reliable assessment of the disease course and the subsequent choice of targeted treatment.

**Objectives:** To explore the relation of urine Neutrophil Gelatinase-Associated Lipocalin (NGAL) and High-Mobility Group Box 1 (HMGB1) protein to: (a) the presence of pLN and (b) the pLN activity, in a homogeneous Caucasian pediatric Systemic Lupus Erythematosus (pSLE) population from Northern Greece.

**Methods:** Thirty-three urine samples were collected from 17 pLN patients and 12 urine samples from 12 pSLE patients without pLN. The pLN activity was assessed using the renal domain of the Systemic Lupus Erythematosus Disease Activity Index 2000 (SLEDAI-2 K). The biomarkers’ levels were determined by ELISA.

**Results:** The levels of the urine NGAL were higher in the pLN patients [median (IQR): 33.46 (19.37-84.7) pg/ml] as compared to the pSLE patients without pLN [14.1 (10.24-21.45) pg/ml] (p = 0.001) and were correlated with the pLN activity (rho = 0.457, p = 0.008). The levels of the urine HMGB1 were higher in the pLN patients [5.55 (4.17- 6.54) ng/ml] as compared to the pSLE patients without pLN [3.81 (2.46-5.1) ng/ml] (p = 0.034) and were correlated with the pLN activity (rho = 0.685, p < 0.001).

**Conclusion:** These preliminary findings indicate that the combination of the urine NGAL and HMGB1 levels may serve as biomarkers of pLN presence in pSLE patients, and may be considered as another non-invasive tool for the assessment of pLN activity. Further studies in Caucasian patients are needed to verify these results.

**Disclosure of Interest:** None Declared

## Treatment

### P153 Smart technologies to improve health outcomes in juvenile idiopathic arthritis

#### Andrea Coda^1^, Dean Sculley^1^, Derek Santos^2^, Xavier Girones^3^, Derek Smith^4^, Joshua Burns^5^, Keith Rome^6^, Jane Munro^7^, Davinder Singh-Grewal^8^

##### ^1^School of Health Sciences, The University of Newcastle, Ourimbah, Australia, ^2^School of Health Sciences, Queen Margaret University, Edinburgh, United Kingdom, ^3^Faculty of Health Sciences, Manresa University, University of Vic-Central University of Catalonia, University of Barcelona), Manresa, Spain, ^4^School of Health Sciences, James Cook University, Townsville, Australia ^5^ Allied Health (Paediatrics), The Children’s Hospital at Westmead & the University of Sydney, Sydney, Australia, ^6^ Acting Director of Health & Research Rehabilitation Institute, AUT University, Auckland, New Zealand, ^7^ Head of Rheumatology Unit, Dept of General Medicine, Royal Children’s Hospital, Parkville - Victoria, Australia ^8^Paediatric Rheumatologists & Paediatrician Consultant, Sydney Children Hospitals Network & Clinical A/Prof- The University of Sydney, Sydney, Australia

###### **Correspondence:** Andrea Coda

**Introduction:** Children and adolescents diagnosed with Juvenile Idiopathic Arthritis (JIA) often exhibit lower physical activity level and poorer aerobic and anaerobic exercise capacity when compared to their non-JIA counterparts. Mild intensity exercise regimen has been proven to be safe in children with JIA and may produce significant improvements in overall physical function. Inadequate adherence to the treatment prescribed by paediatric rheumatologists, could also have a detrimental impact towards different clinical outcomes and possibly increased disease activity. This includes symptoms such as pain, fatigue, quality of life, longer term outcomes including joint damage, as well as increase of healthcare associated costs. Low adherence to medications such as methotrexate and biological-drugs remains a significant issue in paediatric rheumatology, with evidence that less than half of the children with JIA are actually compliant to their drug-therapy. The recent advances in smart technology resulting in a variety of wearable user-friendly interactive devices may become a key solution to tackle important challenges in JIA clinical management.

**Objectives:** The aim of this review was to explore the current use of modern interactive technologies in the provision of health care

**Methods:** A litterature review was performed using MEDLINE, PUBMED and CINAHL. This review focused in 4 main topics: monitoring symptoms and disease progression using interactive technologies, adherence to prescribed medications using smart devices, available app to encourage physical activity and data protection.

**Results:** The recent advances in smart technology resulting in a variety of wearable user-friendly interactive devices may become a key solution to tackle important challenges in JIA clinical management. Fully understanding the impact that JIA and treatment complications have upon patients and their families has long been a challenge for clinicians. Modern interactive technologies can be adapted to the individual requirements and accessed directly in the hands or wrists of children with JIA. These secured networks could be accessible 'live' at anytime and anywhere by the child, parents and clinicians.

Multidisciplinary teams in paediatric rheumatology may benefit from adopting these smart devices to enhance the understanding in different aspects, such as: patient’s biological parameters, symptoms progression, and adherence to drug-therapy, quality of life, and participation in physical activities. Most importantly the use of interactive technologies may also promote more timely clinical decisions, improve self-management and parents active involvement with their child's disease. Paediatric rheumatology research could also further advance from the use of these smart devices, as they would enable real-time access to meaningful data to thoroughly analyse the disease-patterns of JIA, such as pain and physical activity outcomes. Data collection that typically occurs once every 1 or 3 months in the clinical setting could instead be gathered every week, day, minute or virtually live online. Many limitations in wearing such interactive technologies still exist and require further developments and investments.

**Conclusion:** Further studies in paediatric rheumatology are required to critically evaluate the effectiveness and acceptability of already available apps in large number of patients and to develop new devices utilizing valuable input and feedback from patients, carers and clinicians. Finally, by embracing and adapting these new and now highly accessible interactive technologies, clinical management and research progress in paediatric rheumatology could be greatly advanced.

Trial registration identifying number: NA

**Disclosure of Interest:** None Declared

### P154 Reduced-dose rituximab in treatment of pediatric rheumatic diseases

#### Nadina E. Rubio-Perez, Fernando Garcia-Rodriguez, Marcia D. Torres-Made, Manuel E. de la O-Cavazos

##### Pediatric Rheumatology, Departamento de Pediatría, Hospital Universitario "Dr. José E. González", UANL, Monterrey, Mexico

###### **Correspondence:** Fernando Garcia-Rodriguez

**Introduction:** Rituximab (RTX) response does not depend on its serum concentration, so reduced doses (miniRTX) could be an alternative treatment in pediatric rheumatic diseases (PRD), especially in low-income countries.

**Objectives:** To describe the outcome of a longitudinal series of PRD patients treated with miniRTX.

**Methods:** A longitudinal, open-label, non-controlled, case series was conducted. We enrolled all consecutive patients diagnosed with PRD in whom failure to standard therapy was stablished. Treatment with miniRTX, defined as either 500 mg in a single dose infusion or 100 mg a week during 4 weeks, were added to patients’ therapy. A 200 mg dose were administered as maintenance treatment at 6 and 12 months after first dose to those patients whom achieved a significant clinical response. Clinical outcome were registered in every patient during a follow up of at least 18 months.

**Results:** Eight patients were included in this series, seven female, median age 14 (2 – 17) years old at the time of diagnosis and 18 (11 – 82) months of disease duration. Most of the patients (seven) has juvenile systemic lupus erythematosus (JSLE) and one patient presented with juvenile dermatomyositis (JDM).

Among JSLE patients, previous used treatments were azathioprine (3 patients), mofetil mycophenolate (7 patients), and cyclophosphamide (5 patients) in different periods. All those patients received corticosteroids (both orally and/or IV) and hydroxychloroquine. Patient with JDM received corticosteroids, methotrexate, azathioprine, cyclosporine A, and hydroxychloroquine prior to miniRTX.

Clinical manifestations presented in JSLE patients at the time of miniRTX administration were hematological (5 patients), arthritis (4 patients), nephritis (4 patients), vasculitis (2 patients), serositis (1 patient), secondary antiphospholipid syndrome (APL) and neuropsychiatric (1 patient).

Most of the patients received 100 mg/week miniRTX scheme, while two received a single dose (500 mg).

Two patients presented primary failure to therapy (one JSLE and JDM patient) so no maintenance doses were administered. One patient with JSLE and APL presented initial good response but a relapse were evident 12 months after initial administration. Five patients completed the 18 months follow up with absence of JSLE signs and symptoms; however, none of the patients completed an off-treatment remission criterion.

One patient presented recurrent ear infections and secondary immunodeficiency were diagnosed attributable to miniRTX administration. Patient received complementary IVIG with resolution and no other complications were presented in this series.

**Conclusion:** Comparative studies are needed to establish the usefulness of miniRTX in PRD; however, the response in most of the patients of this small series was favorable.

**Disclosure of Interest:** None Declared

### P155 Etanercept (ENBREL®) treatment retention in the sub-population of pediatric patients from a retrospective cohort study using Canadian claims-level data

#### Majed Khraishi^1^, Brad Millson^2^, John Woolcott^3^, Heather Jones^4^, Lisa Marshall^4^

##### ^1^Faculty of Medicine, Memorial University of Newfoundland, St. Johns, Canada; ^2^Health Access and Outcomes, QuintilesIMS, Kanata, Canada; ^3^Inflammation and Immunology, Global Outcomes and Evidence, Pfizer, Collegeville, PA, United States; ^4^Inflammation and Immunology, Global Medical Affairs, Pfizer, Collegeville, PA, United States

###### **Correspondence:** Majed Khraishi

**Introduction:** Since its initial approval for the treatment of patients with moderate to severe refractory rheumatoid arthritis (RA), etanercept (ETN; Enbrel®), a recombinant fusion protein, has led the expansion of therapeutic options available for patients with other inflammatory diseases.^1^ ETN was the first biologic approved for use in the treatment of patients with polyarticular-course juvenile idiopathic arthritis (JIA),^2^ and is now indicated in the JIA categories, extended oligoarthritis, enthesitis-related arthritis, and psoriatic arthritis.^3^ Use of ETN in patients with JIA has seen significant benefit in reducing disease symptoms^4^ and radiographic progression.^5^ There is evidence from registry studies and real-world data sources that ETN is favored as a first-line biologic therapy in clinical practice in patients with JIA;^6,7^ however, the factors associated with long-term retention of ETN in this patient population have been little explored.

**Objectives:** To evaluate retention rates up to 6 years in ETN-treated pediatric patients in Canada.

**Methods:** A retrospective cohort study was conducted using longitudinal prescription drug claims data from QuintilesIMS Private Drug Plan database (PDP), Ontario Public Drug Plan database (OPDP), and Quebec Public Drug Plan database (RAMQ). Between 07/2008 and 06/2010, biologic-naïve patients (ie, patients with no biologic treatment in the preceding 12 months) who initiated ETN, were identified and followed for 72 months. Disease indications were inferred through patient drug history. 12-month retention rates were evaluated in 1-year increments for all patients retained on therapy at years 1, 2, 3, 4, 5, and 6 post initiation, and comparisons made to retention rates in the first year with *P*-values reported. Two-proportion z-tests were made with reference to year-1 retention; the Bonferroni method was used to counteract the problem of multiple comparisons.

**Results:** The study identified 4528 ETN-treated patients (61% female, 85% rheumatic diseases, and 15% psoriasis) across Canada, who initiated therapy during the selection period. 65 (1.4%) were identified as pediatric patients (ie, age 2-12 years; 94% with JIA) at the time of therapy initiation. The majority of patients were from Ontario (48%) and insured by private drug plan. 12-month ETN retention rates for the pediatric patients increased following their first year on therapy. 68% of patients were retained at year 1; 12-month retention rates through years 2-6 are shown in the Table. Retention rates for the corresponding periods in the adult population (>18 years) were: 66%, 79%, 82%, 84%, 83%, and 79%. A total of 23.1% (n = 15) of pediatric patients remained on ETN treatment for the entire 72 months of the study.

**Conclusion:** Pediatric patients who were treated with ETN demonstrated higher retention rates after the first year, particularly if they were maintained on ETN for more than 2 years. The sample size of pediatric patients in this study is relatively small. Further analysis of the reasons for ETN treatment discontinuation in a larger sample of pediatric patients may assist in identifying measures to support patients in maintaining treatment to achieve sustained clinical benefit^4^ and quality of life.^8^

**References:** 1. Scott LJ. *Drugs* 2014;74:1379-1410; 2. Hinze C, et al. *Nat Rev Rheumatol* 2015;11:290-300; 3. Windschall D, et al. *Clin Rheumatol* 2015;34:61-9; 4. Giannini EH, et al. *Arthritis Rheum* 2009;60:2794-804; 5. Nielsen S, et al. *Clin Exp Rheumatol* 2008;26:688-92; 6. Horneff G, et al. *Arthritis Res Ther* 2016;18:272; 7. Verazza S, et al. *Pediatr Rheumatol Online J* 2016;14:68; 8. Klotsche J, et al. *Arthritis Care Res (Hoboken)* 2014;66:253-62.

**Disclosure of Interest**: M. Khraishi Consultant for: Pfizer, Canada and Amgen, Canada, B. Millson Employee of: Quintiles, J. Woolcott Shareholder of: Pfizer, Employee of: Pfizer, H. Jones Shareholder of: Pfizer, Employee of: Pfizer, L. Marshall Shareholder of: Pfizer, Employee of: Pfizer

**Table 43:** **(Abstract P155).** ETN treatment retention in pediatric patients (aged 2-12 years)

Year	Tracked Patients, n	Retained Patients, n	Retention Rate, %	*P*-Value
1	65	44	68	
2	41	33	80	0.1501
3	31	28	90	0.0166
4	28	25	89	0.0290
5	25	19	76	0.4411
6	18	15	83	0.1952

### P156 A case report of a pediatric patient with orbital IGG4-related disease

#### Anna Kozlova, Vasiliy Burlakov, Dmitriy Abramov, Garik Sagoyan, Anna Shcherbina

##### Immunology, Federal State Budgetary Institution "National Scientific and Practical Center of Pediatric Hematology, Oncology and Immunology named after Dmitry Rogachev" of the Ministry of Healthcare of the Russian Federation, Moscow, Russian Federation

###### **Correspondence:** Anna Kozlova

**Introduction:** IgG4-related disease (IgG4-RD) is a fibroinflammatory condition that can affect essentially any organ.

**Objectives:** The disease shows similar histopathological findings across organs and systems, and consist of a lymphoplasmacytic infiltrate enriched in IgG4-positive plasma cells. Most patients with IgG4-RD respond at least partially to glucocorticoids, but is it toxic therapy. Treatment protocols of IgG4-RD in children are not established.

**Methods:** We report a case of pediatric patient with orbital IgG4-related disease and successful treatment JAK inhibitor – ruxolitinib.

**Results:** Thirteen years old boy has been suffering from hyperemia of the left eye and edema of the eyelid. The MRI showed a lesion adjacent to the orbita. The child underwent biopsy of the tumor, and the histologic examination revealed lymphoplasmacytic iniltrates with fibrosis and vasculitis correlating with IgG4-related disease. Serum IgG4 level was not elevated. Blood tests and acute phase reactants were normal. He was started on JAK inhibitor therapy (ruxolitinib) with good effect. His tumor mass of eyes changed from 25 to10mm for 2 months. The condition and quality of life of the child became better. Adverse events were not noted.

**Conclusion:** In our case of IgG4-RD patient JAK inhibitor – ruxolitinib therapy was highly effective and is a safe treatment modality but indications for therapy require further investigation.

**Disclosure of Interest:** None Declared

### P157 Optimum serum adalimumab levels in juvenile idiopathic arthritis according to the response criteria

#### Berta Lopez^1^, M. Isabel Gonzalez^1^, Miguel Marti^1^, Lorena Martinez^2^, Silvia Gabriela Ceberio^2^, Inmaculada Calvo^1^

##### ^1^Pediatric Rheumatology, Huip La Fe, Valencia, Spain; ^2^Pharmacology, Huip La Fe, Valencia, Spain

###### **Correspondence:** BERTA LOPEZ

**Introduction:** Serum adalimumab (ADA) levels have been related to treatment response in juvenile idiopathic arthritis (JIA).

**Objectives:** The aim of this study is to determine the optimal cutoff point of adalimumab in our cohort of JIA patients in function of the variable chosen for remission: inactive JIA.

**Methods:** Retrospective observational study. Patients with JIA and pharmacokinetic monitoring of adalimumab, between September 2014 and February 2017, were included. The JIA improvement criteria were used: visual analogue physician scale (EVA physician), visual analogue patient scale (EVAp), number of active joints, number of limited joints, CHAQ (quality of life questionnaire) and ESR. Inactive JIA was defined according to Wallace criteria as the absence of active joints, systemic symptoms and uveitis; normal ESR or PCR values; EVA medical negative and duration of stiffness less than 15 minutes. The 7 subtypes of JIA were oligorticular, polyarticular, psoriatic and enthesitis related arthritis.

**Results:** Thirty-four patients (58.82% female) were included in the study, with a total of 50 determinations. The mean age was 12.35 years (95% CI: 10.45-14.26). The forms of JIA were: oligoarticular 35.29% (n = 12), polyarticular 29.41% (n = 10), enthesitis related arthritis 23.53% (n = 8) and psoriatic arthritis 11.77% (n = 4). All of patients were given methotrexate concomitantly. The mean ADA levels according JIA forms were: oligoarticular 9.21 mcg/mL, polyarticular 8.36 mcg/mL, enthesitis related arthritis 7.73 mcg/mL and psoriatic arthritis 13.44 mcg/mL. In the group with uveítis, ten of them had active uveitis, predominantly oligoarticular and polyarticular (90%). The mean ADA levels were 9.80 and 5.88 mcg/mL (p = 0.019) inactive and active uveitis respectively. Mean levels were higher in patients with inactive JIA compared to patients with active JIA (10.76 mcg/mL vs. 7.33 mcg/mL, p = 0.024). AUC (IC95%) according to the ROC curve was 0.70 (0.53-0.86) with an optimal cutoff of 11 mcg/mL (Sensitivity (Se) = 52.6%; Specificity (Ep) = 85.5%).

**Conclusion:** In our cohort of patients, it has been observed that inactive JIA requires ADA levels higher than those of active JIA. On the other hand, despite the predominance of oligoarticular and polyarticular forms in active uveitis, psoriatic arthritis requires higher levels of ADA. Finally, the optimal ADA cut-off point indicates that the levels of ADA for JIA are higher than those generally established for adult rheumatology patients.

**Disclosure of Interest:** None Declared

### P158 Efficacy of omalizumab treatment in a girl with autoinflammatory desease and chronic urticaria

#### Maria Cristina Maggio^1^, Anna Lucania^2^, Giovanni Corsello^1^

##### ^1^University Department Pro.Sa.M.I. “G. D’Alessandro”, University of Palermo, Palermo, Italy; ^2^II Pediatric Unit,, “G. Di Cristina” Children Hospital, ARNAS, Palermo, Palermo, Italy

###### **Correspondence:** Maria Cristina Maggio

**Introduction:** Chronic Idiopathic Urticaria (CIU) is associated to angioedema in 40% of patients, with a prevalence of 0.1-0.3%, a medium age at the diagnosis of 6-11 years. Furthermore, the 25-85% of cases remain with a diagnosis of an “idiopathic” disease, for the negativity of all the diagnostic tests. 30-50% of patients have an autoimmune origin, although confirmation of the diagnosis in these cases is not easy. Some patients, in fact, have autoantibodies against the high-affinity IgE receptor FcεR1 or the IgE. These patients, show an increased incidence of anti-thyroid autoantibodies, and represent 30-50% of the patients designated as having CIU.

CAPS are Autoinflammatory diseases (AID) classically characterized by recurrent episodes of fever, rash, significant increase of inflammatory markers (SAA, CRP, ESR, neutrophil leukocytes), arthralgia, myalgia, abdominal and chest pain; these patients can have severe clinical manifestations, which require to be treated with biological drugs anti-IL-1.

**Objectives:** However, in some patients genetic analysis of the candidate genes (NLRP3, NLRP12…) are negative and the best treatment is hard to decide.

**Methods:** We describe the clinical case of a 9-year-olf-female with recurrent monthly episodes of fever (> 38,5 ° C for about 5 days), arthralgia, abdominal pain, urticaria-angioedema, without itch, significant increase of SAA, CRP, ESR, leucocytosis. ANA, ENA, ASCA, ANCA, LAC, RAST, IgE, thyroiditis and coeliac disease markers, C3 and C4 anti-C1q inhibitor e C1q inhibitor gene mutations were negative. The genetic study of AID (FMF, TRAPS, MVK, NLRP3, NLRP12) was negative.

**Results:** The attacks only partially responded to high doses of steroids and antihistaminic drugs. She was tested with omalizumab, a monoclonal humanized murine antibody direct against IgE. The treatment, off-label for age, induced a prompt and persistent resolution of the clinical manifestations and the normalization of inflammatory markers. Fever and arthralgia resolved and- after 5 months of treatment- she did not present more attacks.

**Conclusion:** Omalizumab induced an on-going positive response and was well tolerated without side effects. The omalizumab effect was extended on inflammatory markers and symptoms, suggesting the possible employ of the drug in severely symptomatic patients in whom urticaria is the dominant sign and genetic analysis does not support the diagnosis of CAPS.

**Disclosure of Interest:** None Declared

### P159 The use of consequential biologic DMARDs in paediatric rheumatology

#### Joseph Mcallister^1^, Adam Clough^2^, Phil Riley^1^, Alice Chieng^1^

##### ^1^Paediatric Rheumatology, Royal Manchester Childrens Hospital, Central Manchester University Hospitals, Manchester, United Kingdom; ^2^Pharmacy, Royal Manchester Childrens Hospital, Central Manchester University Hospitals, Manchester, United Kingdom

###### **Correspondence:** Joseph Mcallister

**Introduction:** Biologic DMARDs (bDMARDs) are integral to the management of paediatric rheumatological disease. There is an increasing cohort of patients who have tried more than one bDMARD and the indications and side effects are not well studied. In our department some have received up to four different bDMARDs. Despite their widespread use, there is limited evidence about the consequential use of different bDMARDs in paediatric rheumatology.

**Objectives:** The aim of this study was to investigate the consequential use of bDMARDs in the paediatric rheumatology. The outcomes were to define local practice and the group of patients who clinically need multiple bDMARDs. We also wanted to look at the adverse effects of receiving consequential bDMARDs.

**Methods:** Using hospital pharmacy records, a database of paediatric rheumatology patients who received two or more bDMARDs was created. Patient records were scrutinised for demographics and diagnoses. Data collection was completed by documenting bDMARDs, and recording information pertinent to each course of treatment including the duration, side effects, reported infections and reason for changing to another bDMARD.

**Results:** From February 2015 to March 2017 there were 34 patients who received consequential bDMARDs who collectively underwent 48 medication changes. Their diagnoses included juvenile idiopathic arthritis (JIA) (poly-JIA - 14%; oligo-JIA alone – 8.2%; systemic JIA – 4.1%; oligo JIA and uveitis - 18.8%). Enthesitis-related arthritis, uveitis and sarcoidosis accounted for the remainder. 25 patients received two bDMARDs, 10 patients received three bDMARDs and 4 patients received four bDMARDs. The mean duration of treatment was 49.4 months (range 15 - 132 months).

Etanercept was prescribed 22 times, usually first line (90%). Adalimumab was prescribed 31 times, mostly second line (77%). Infliximab was prescribed 16 times mostly first line (56%). Tocilizumab was prescribed for 13 patients mostly as third (46%) and fourth line (23%) therapy and never as first line. Abatacept was prescribed once as fourth line. Anakinra was used twice. Practice was mostly in keeping with BSPAR guidance for bDMARD usage.

Many factors contributed to switching. Most common were poor control of JIA (41.6%), uveitis (18.8%), and infections (5%). Intolerance to administration for adalimumab and etanercept was common (22%). Adverse reactions to infliximab accounted for 8%.

Patients requiring three or more bDMARDs had diagnoses of antibody negative oligo-JIA (7/10) six of whom also had active uveitis. The three remaining patients had poly-JIA and two of these were Rheumatoid Factor positive (RF+).

158 infections were reported, most of which were mild. Eight infections required hospitalisation. The most common infections were upper respiratory tract infections (40.5%), urinary tract infections (12%), ear infections, cellulitis and pneumonia (7% each). Other serious infections reported were appendicitis, central line sepsis and sialitis. Minor infections includeded thrush, gastroenteritis, threadworms, tinea, dental infections, herpes simplex, varicella zoster, paronychia and infected ingrown toenails (Table 44).

**Table 44:** **(Abstract P159).** See text for description

Results table							
bDMARD	No. patients	No. treatment years	Observed no. infections	Infections per 100 patient years	Observed no. adverse events	Adverse events per 100 patient years	% changed for ongoing JIA or uveitis
1st agent	34	51.1	64	3.68	23	3.68	58.8%
2nd agent	34	77.8	76	2.87	12	0.45	70%
3rd agent	10	5.8	10	17.39	2	3.48	25%
4th agent	4	5.3	8	37.5	0	0	-
Total		1680	158				

**Conclusion:** This investigation highlights practice at a tertiary centre in which bDMARD use is frequent. The results show factors contributing to bDMARD switching and this can aid decision making when commencing therapy. This needs further studying as 41.6% reported poor efficacy with their first bDMARD. It appears that patients with antibody negative oligo-JIA and uveitis, and possibly RF + ve poly-JIA are at risk of poor response. Infection rates increased during 3rd and 4th bDMARD courses, and although reducing patient numbers are confounding, this is an interesting area for future research given the impact on quality of life. Future research should follow patients into adulthood to demonstrate long term effects of these medications.

**Disclosure of Interest:** None Declared

## Uveitis

### P160 The persistence of ana positive in JIA patients and the risk of developing chronic anterior uveitis: a retrospective study

#### Alina Lucica Boteanu, Maria Llop Vilaltella, Maria Andreina Terán Tinedo, Maria Angeles Blazquez Cañamero, Mariluz Gamir Gamir

##### Rheumatology, University Hospital Ramon Y Cajal, Madrid, Spain

###### **Correspondence:** Alina Lucica Boteanu

**Introduction:** Juvenile idiopathic arthritis (JIA) is one of the most frequent rheumathologic conditions in childhood. The most common extra-articular manifestation of JIA is chronic anterior uveitis (CAU). It usually has an insidious, asymptomatic onset, with a chronic and recurrent course, being blindness its major complication. Several risk factors to develop CAU has been reported, including early age at the onset of arthritis (before 6 years), short disease duration and some JIA subtypes (oligoarticular-persistent and extended-oligoarticular JIA, psoriatic arthritis, undifferentiated arthritis). The presence of positive antinuclear antibodies (ANA) tests is one of the most important risk factors. In some cases ANA are present at the onset of the disease, subsequently becoming negative.

**Objectives:** To determine if the presence of steadily ANA positivity (>2 determinations) throughout disease course is associated with an increased risk of developing chronic anterior uveitis

**Methods:** We performed a retrospective study including JIA patients with high-risk to develop CAU and at least one positive ANA determination during the follow-up at the Pediatric Rheumatology Unit of our center. A cut-off point titer of 1/80 was considered for ANA positivity. The χ^2^ test was performed with Fisher's adjustment. The level of significance was set by the 95% confidence interval (CI) and p <0.05.

**Results:** A total of 53 JIA patients were included, 48 (90.5%) of them are girls, with a mean age at diagnosis of 3 years. 16 patients developed CAU (31.3% of the girls and 20% of the boys), without a statistically significant difference in the percentage of patients who developed CAU between the genders or age. When dividing into groups, 52% of the girls had 1or 2 positive ANA determinations and 48% had ≥3 positive determinations; 40% of the boys had 1or 2 positive ANA determinations and 60% had ≥3 positive determinations. Analysing by groups, patients with 3 or more determinations an OR: 4.93 (95% CI: 1.32-18.31) to develop CAU compared to those with 1or 2 positive determinations.

**Conclusion:** Our results are similar to others studies previously published regarding the absence of association of chronic anterior uveitis with the female sex, based on the frequency difference in the largest number of girls with JIA who debut at an early age. Traditionally, 2 positive ANA determinations are considered to be associated with a high risk of CAU. In addition, the results of our study suggest that the persistence of ANA is associated with an even higher risk of developing chronic anterior uveitis. Further studies with a larger cohort are needed to confirm these findings.

**Disclosure of Interest:** None Declared

### P161 Uveitis associated with juvenile idiopathic arthritis: lower prevalence and unique clinical characteristics in Asian patients

#### Pauline Chan Ng, Lee Kean Lim, Elizabeth Youning Ang, Pei Ling Ooi

##### Khoo Teck Puat-National University Children’s Medical Institute, National University Hospital, Singapore, Singapore

###### **Correspondence:** Pauline Chan Ng

**Introduction:** Uveitis is the most frequent extra-articular manifestation in Juvenile Idiopathic Arthritis (JIA) and results in significant morbidities including vision loss. Paucity of data on JIA-associated uveitis (JIA-U) in Asia makes it challenging to develop well-informed clinical practice guidelines for screening of JIA-U in this population.

**Objectives:** We aim to describe the prevalence, clinical characteristics and risk factors of JIA-U in patients treated in a Paediatric tertiary centre in Singapore.

**Methods:** 103 patients were diagnosed with JIA in our hospital from Jan 2007 to Feb 2017. We analysed the electronic medical records (EMRs) of 83 of these patients. Twenty patients were excluded from our study: 15 did not have available EMRs and 5 made only one visit to the Paediatric Rheumatology clinic.

**Results:** Among 83 patients with JIA, 29 (34.9%) were girls and 54 (65.1%) were boys. Most of the patients were Chinese (n = 39, 47.0%), 22 (26.5%) Indonesian, 10 (12.0%) Indian and 2 (2.4%) Malays. Ten patients were from Bangladesh, Cambodia, Korea, Vietnam, Sri Lanka and East Timor. The subtypes of JIA were: enthesitis related arthritis (ERA) (35.7%), oligoarthritis (22.6%), systemic JIA (15.5%), seronegative polyarthritis (15.5%), seropositive polyarthritis (7.1%), psoriatic arthritis (2.4%) and undifferentiated (1.2%). Twenty five of 72 patients (34.7%) were positive for ANA.

Four children (4.8%) were diagnosed with uveitis: 3 had ERA and developed anterior uveitis, while 1 had oligoarticular JIA and had panuveitis. All 4 affected patients were negative for ANA and Rheumatoid factor. All 3 patients with ERA were symptomatic for uveitis and had bilateral involvement. The patient with oligoarticular JIA presented with unilateral ptosis only. Mean age at diagnosis of uveitis was 10.3 years (CI 2.34–18.26). Clinical characteristics of the 4 patients with JIA-U are shown in Table 45.

**Table 45:** **(Abstract P161).** Clinical characteristics of patients with JIA-U. (Abbreviations: MTX, methotrexate)

Patient	JIA subtype	Gender; Ethnicity	Age of uveitis diagnosis (years)	Uveitis diagnosis from JIA diagnosis (years)	Symptom onset to diagnosis (months)	Symptoms	Disease extent	Complication	Treatment
1	ERA	Male; Indonesian	9.2	– 3.8	0.6	Pain and redness	Bilateral; anterior	–	Topical steroids, Adalimumab
2	ERA	Male; Indonesian	13.8	1.3	1.1	Redness, blurring of vision, photophobia	Bilateral; anterior	Macular oedema	Topical and systemic steroids, MTX, Adalimumab
3	ERA	Male; Indian	13.0	0	1.6	Redness	Bilateral; anterior	–	Topical and systemic steroids, MTX, Adalimumab
4	Oligoarticular-extended	Male; Indian	5.1	1.5	5.2	Left eye ptosis	Left; panuveitis	Macular oedema, glaucoma, cataract	Topical and systemic steroids, MTX, Infliximab; cataract surgery; YAG laser

**Conclusion:** Our Asian population demonstrates distinct differences to our Western counterparts:

Our patients have a much lower prevalence at 4.8%. Literature from Western populations cite prevalence of JIA-U between 11 and 30%. Even though our Asian patient population is fairly heterogeneous, our prevalence is consistent with the 4.7% prevalence demonstrated in a study done in Taiwan, a more homogeneous Asian population.

Our Asian population did not demonstrate the same risk factors for JIA-U as in previous studies. There was a much higher association of uveitis with ERA in both our and the Taiwanese study. Frequently reported risk factors of ANA-positivity, young age of disease onset, female gender were not evident in our population.

Lastly, majority (3 of 4) of our patients were symptomatic for uveitis.

These preliminary findings have implications for screening guidelines for uveitis in Asian patients with JIA. Larger population studies are required to characterize the risk factors for JIA-U to guide development of such guidelines.

**Disclosure of Interest:** None Declared

### P162 Our modest experience with adalimumab in the treatment of juvenile idiopathic arthritis associated uveitis

#### Almira Ćosićkić^1^, Fahrija Skokić^2^, Sanimir Suljendić^3^, Meliha Halilbašić^4^, Amela Selimović^5^

##### ^1^Department of Allergy, Rheumatology and Immunology, Children's Hospital, Tuzla, Bosnia and Herzegovina; ^2^Department of Neonatology, Children's Hospital, Tuzla, Bosnia and Herzegovina; ^3^Department of Children's Surgery, Children's Hospital, Tuzla, Bosnia and Herzegovina; ^4^Ophthalmology Clinic, Tuzla, Bosnia and Herzegovina; ^5^Department of Intensive Care and Therapy, University Clinical Center, Tuzla, Bosnia and Herzegovina

###### **Correspondence:** Almira Ćosićkić

**Introduction:** Juvenile idiopathic arthritis (JIA) is commonly complicated by chronic uveitis. Therapy of JIA-associated uveitis is guided by the severity of inflammation and complications. When the standard anthireumatic drugs and the local eye therapy are insufficient we need to consider immunosuppressive or biological agents.

**Objectives:** Observation was aimed at assessing the effectiveness of adalimumab (ADA) in the treatment of juvenile idiopathic arthritis associated uveitis in children with disease resistance to standard antirheumatic therapy.

**Methods:** 7 patients with JIA (5 oligoarticular, 2 polyarticular) and who experienced eye problems were included in the study, 6 girls and 1 boy. Mean age was 6.3 years; the age of disease onset was 4.4 ± 1.2 years. Prior to ADA administration all children received the topical treatment of uveitis and methotrexate and 4/7 children received oral corticosteroids. 4 children had bilateral eye involvement and 3 children had unilateral; 5 children had active uveitis; most children had the moderate activity of arthritis; mean ESR was 23.4 ± 15.3 mm/h; CRP 72 ± 18.1 mg/dl (ref <3.0 mg/dl).

**Results:** After 6 months of ADA administration, uveitis remission was achived in 27.3% eyes, 18.2% eyes showed a reduction in inflammatory activity, flares was observed in 2/7 children; after 12 months of ADA 42.8% eyes had uveitis remission, 14.3% eyes had sub active uveitis. Monitoring arthritis for 6 months of ADA administration, 95% children achieved ACR ped-30, 57.1% ACR ped 50 with substantially decreased ESR, CRP also; after 12 months of ADA, ACR ped-30 and ACR ped-50 was achieved in all children, while ACR ped-70 in 4 children; mean ESR decreased to 11.4 ± 2.3 mm/h and also CRP from 72 ± 18.1 mg/dl to 25.2 ± 2.1 mg/dl.

**Conclusion:** Our modest experience has confirmed that adalimumab in the treatment of JIA associated with uveitis has the significant efficacy in the majority of children.

**Disclosure of Interest:** None Declared

### P163 A rare case of recurrent bilateral optic disc edema in tubulointerstitial nephritis and uveitis syndrome treated with infliximab

#### Siska E. Dhaese^1^, Joke Dehoorne^2^, Jean-Baptiste Willemot^3^, Johan Vande Walle^2^, Ilse De Schryver^3^

##### ^1^Medical School, Ghent University, Ghent, Belgium; ^2^Pediatric Nephrology and Rheumatology Unit, Ghent University Hospital, Ghent, Belgium; ^3^Ophthalmology, Ghent University Hospital, Ghent, Belgium

###### **Correspondence:** Siska E. Dhaese

**Introduction:** Tubulointerstitial Nephritis and Uveitis syndrome (TINU) is a rare disorder characterized by inflammation of the tubulointerstitium associated with recurrent unilateral or bilateral uveitis. Since TINU syndrome may not be obvious at initial presentation, it remains often underdiagnosed.

**Objectives:** To report a case of recurrent bilateral optic disc edema in a definite TINU syndrome with limited responsiveness to corticosteroid therapy and immunosuppressants. An anti-TNF-α blocking agent was added in order to control the inflammation.

**Methods:** Observational report about a 13-year-old Caucasian boy diagnosed with TINU syndrome and bilateral papilledema. An extensive general and ophthalmological workup confirmed the diagnosis of TINU.

**Results:** A 13-year-old boy diagnosed with TINU syndrome, was referred to our department with a bilateral papilledema. In a 25-months follow-up 4 episodes of bilateral anterior uveitis were observed. At the 2nd episode the anterior chamber inflammation was accompanied by bilateral optic disc edema. The ocular inflammation responded initially well to systemic corticosteroids but after a recurrence-free period of 1-year, bilateral papilledema recurred. At referral ocular examination showed mild anterior chamber reaction with bilateral optic disc edema. Visual field testing was normal and optical coherence tomography showed a thicker peripapillary retinal nerve fiber layer (PPRNFL). Immunosuppressive therapy with mycophenolate mofetil and methotrexate failed to control the inflammation. A treatment with infliximab, an anti-TNF-α blocking agent, was initiated. Initially remission with a recurrence-free period of 7 months was achieved but papilledema recurred.

**Conclusion:** TINU syndrome may rarely manifest with optic disc edema. To our knowledge, this is the first case of TINU syndrome with bilateral papilledema treated with infliximab. Despite a recurrence-free period of 7 months, the anti-TNF-α blocking agent failed to control the papilledema. Since the limited responsiveness to anti-TNF-α blocking agents, alternative treatment options need to be explored.

**Disclosure of Interest:** None Declared

