# Correction to: P92 chronic arthritis as the only manifestation of FMF in Armenian children

**DOI:** 10.1186/s12969-017-0211-z

**Published:** 2017-11-27

**Authors:** Gayane Khloyan, Gayane Amaryan, Rotraud Saurenmann

**Affiliations:** 1 ARABKIR JMS ICAH, Yerevan, Armenia; 2 Yerevan State Medical University, Yerevan, Armenia; 3 Cantonal Hospital, Winterthur, Switzerland

## Correction

After publication of the below abstract in supplement [1], it was brought to our attention that in abstract P92, two of the authors were accidentally omitted from the author list. The second author should have been Gayane Amaryan and the third author should have been Rotraud Saurenmann.

## P92 Chronic arthritis as the only manifestation of FMF in Armenian children

### Gayane Khloyan^1^, Gayane Amaryan^1,2^, Rotraud Saurenmann^3^.

#### ^1^ARABKIR JMS ICAH, Yerevan, Armenia. ^2^Yerevan State Medical University, Yerevan, Armenia. ^3^Cantonal Hospital, Winterthur, Switzerland.

##### **Correspondence:** Gayane Khloyan

**Introduction:** Familial Mediterranean Fever (FMF) is the most common inherited auto inflammatory disease, characterized by recurrent, self–limited attacks of fever and aseptic polyserositis.FMF is widespread in Armenia and there is a higher than expected frequency of FMF-associated arthritis in children. Chronic arthritis can be the first, and sometimes the only manifestation of FMF.

**Objectives:** To the importance of genetic investigation for MEFV gene mutations in patients of high-FMF-prevalence ethnicities with chronic arthritis.

**Methods:** Case reports of two patients with chronic arthritis as their first symptom of FMF.

**Results: 1-st case:** A 6 year-old boy was admitted to the hospital with complaints of weakness and limping during the last 1.5 years. He had also self- limited episodes of knee and foot pain. The physical examination revealed pain on flexion of the left knee, painful palpation of both sacroiliac joints, but no visible or palpable changes of the joints. Initial laboratory examination showed elevation of ESR– 49 mm/h and CRP-48 mg/l. WBC, RBS, PLT were in normal range. An extensive diagnostic work up including biochemical examination, serology tests, urine test, chest X-ray, Echo-CG revealed no changes. Splenomegaly was found on abdominal sonography. MRI of the pelvic bones and hip joints indicated signs of bilateral sacroiliitis. Chronic arthritis was diagnosed. Despite of absence of clinical features of FMF, genetic analysis for MEFV gene mutations was done and homozygous genotype for M694 V mutation (M694 V/M694 V) was found. Treatment with colchicine and sulfasalazine was started and both his clinical condition and laboratory findings improved significantly. Only occasionally the patient still has gait abnormalities.

**2-nd case.** A 1.8 year-old girl was admitted to the hospital because of pain, swelling and deformity of the left wrist, which had started 5 months earlier. In her past history 2 episodes of unexplained fever of 1 day duration were noticed. The physical examination revealed swelling, pain and limitation of motions of the left wrist. ESR was elevated within 53 mm/h, but CRP was negative. Thorough diagnostic work up (CBC, biochemical examination, serology tests, urine test, chest X-ray, Echo-CG, abdominal sonography) revealed no pathologies. MRI of the left wrist indicated signs of arthritis. Chronic arthritis was diagnosed. But also genetic analysis of MEFV mutations was done and 2 mutations (M694 V/R761H) in compound-heterozygous state were found. Intraarticular injection of triamcinolone acetonide to the left wrist was done and colchicine treatment was started with significant improvement: during 4 months of follow up the girl had no complaints and no inflammatory activity with normal level of ESR.

**Conclusion:** In ethnicities with high prevalence of FMF, screening for MEFV gene mutations is recommended for patients with chronic arthritis even in the absence of FMF symptoms.

**Disclosure of Interest:** None Declared.
